# 6-(Tetrazol-5-yl)-7-aminoazolo[1,5-*a*]pyrimidines as Novel Potent CK2 Inhibitors

**DOI:** 10.3390/molecules27248697

**Published:** 2022-12-08

**Authors:** Grigoriy V. Urakov, Konstantin V. Savateev, Svetlana K. Kotovskaya, Vladimir L. Rusinov, Alexandr A. Spasov, Denis A. Babkov, Elena V. Sokolova

**Affiliations:** 1 Department of Organic and Biomolecular Chemistry, Ural Federal University Named after the First President of Russia B.N. Eltsin, Mira St. 19, 620002 Yekaterinburg, Russia; 2 Scientific Center for Innovative Drugs, Volgograd State Medical University, 400131 Volgograd, Russia

**Keywords:** azolo[1,5-a]pyrimidines, tetrazoles, regioselectivity, casein kinase 2, inhibitors, structure-activity relationship

## Abstract

In this work, we describe the design, synthesis, and structure-activity relationship of 6-(tetrazol-5-yl)-7-aminoazolo[1,5-*a*]pyrimidines as inhibitors of Casein kinase 2 (CK2). At first, we optimized the reaction conditions for the azide-nitrile cycloaddition in the series of 6-cyano-7-aminoazolopyridimines and sodium azide. The regioselectivity of this process has been shown, as the cyano group of the pyrimidine cycle was converted to tetrazole while the nitrile of the azole fragment did not react. The desired tetrazolyl-azolopyrimidines were obtained in a moderate to excellent yields (42–95%) and converted further to water soluble sodium salts by the action of sodium bicarbonate. The obtained 6-(tetrazol-5-yl)-7-aminopyrazolo[1,5-*a*]pyrimidines **2a–k** and their sodium salts **3a–c**, **3g–k** showed nano to low micromolar range of CK2 inhibition while corresponding [1,2,4]triazolopyrimidines **10a–k** were less active (IC_50_ > 10 µM). The leader compound 3-phenyl-6-(tetrazol-5-yl)-7-aminopyrazolo[1,5-*a*]pyrimidine **2i** as CK2 inhibitor showed IC_50_ 45 nM.

## 1. Introduction

Casein Kinase 2 (CK2) is a highly conserved polyfunctional serine/threonine protein kinase that plays an important role in the regulation of the processes of several cells, such as proliferation, differentiation and survival [1]. It is considered that CK2 has been implicated in the manifestation of some diseases, including multiple sclerosis [2], inflammation [3], hypertension [4], and viral infections [5]. The role of CK2 has been extensively studied in the development of malignant tumors and it was proved as a key regulator of multiple oncogenic pathways, including the PI3K/Akt, JAK/STAT, IL-6 and NF-jB signaling cascades [6]. In turn, CK2 is a key suppressor of cell apoptosis [7], which determines its role in oncogenesis of several tumors with overexpression of CK2, including breast carcinoma, adenocarcinoma of the lung, prostate carcinoma and lymphomas [8]. It can be noted that Silmitasertib has been approved by the FDA for the treatment of cholangiocarcinoma as CK2 inhibitor [9]. Thus, the development of novel CK2 inhibitors as chemotherapeutic agents against cancer and other nosologies where this type of kinases is involved is a relevant task.

**Figure 1:**
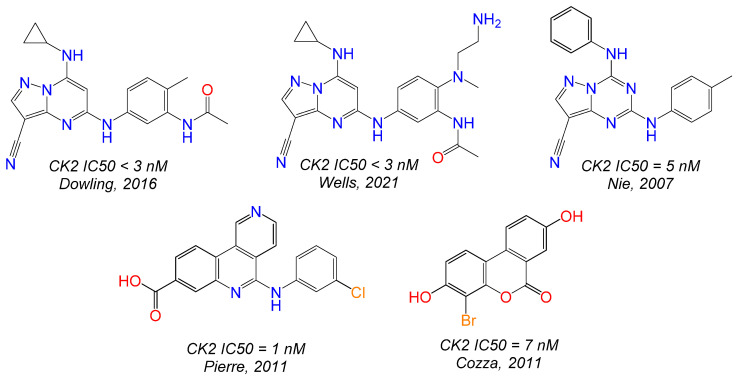
Examples of pyrazoloazines and other molecules with high affinity for CK2 [11,12,13,14,15].

Previously, a wide variety of different molecules have been described as CK2 inhibitors, including polyhalogenated benzimidazole and benzotriazole derivatives [10], nitrogen-containing heterocycles [11,12,13] and their polycondensed analogues [14], as well as condensed coumarin derivatives [15] (Figure 1). Azoloazines heterocycles with bridge nitrogen atom are of considerable interest, since many representatives of this class are known to inhibit CK2 in the low nanomolar range. However, most of the currently available CK2 inhibitors lack the potency, physiochemical, and pharmacological properties required to be successful in clinical trials.

It should be noted that related azolopyrimidines are a privileged class of heterocycles in medicinal chemistry as they demonstrate a wide range of biological activities, in particular, anticoagulant [16], anti-inflammatory [17], antidiabetic [18], hypotensive [19], antiseptic [20].

**Figure 2:**
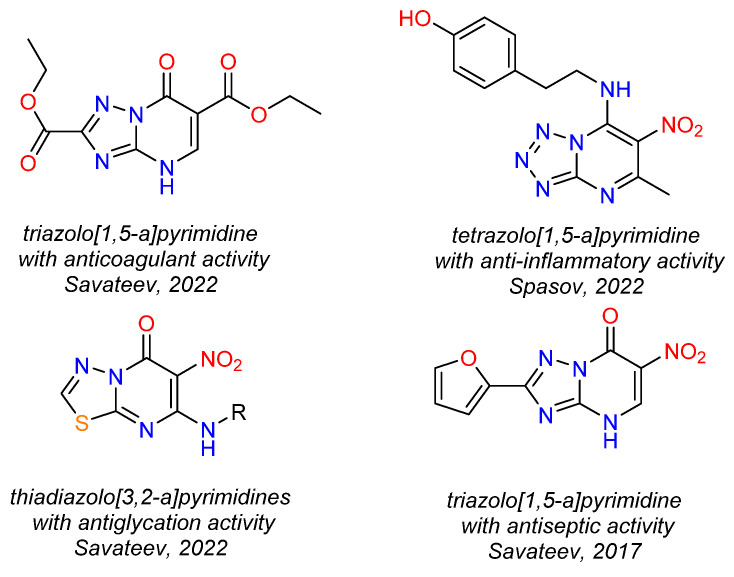
Examples of biologically active azolopyrimidines [16,17,18,20].

**Figure 3:**
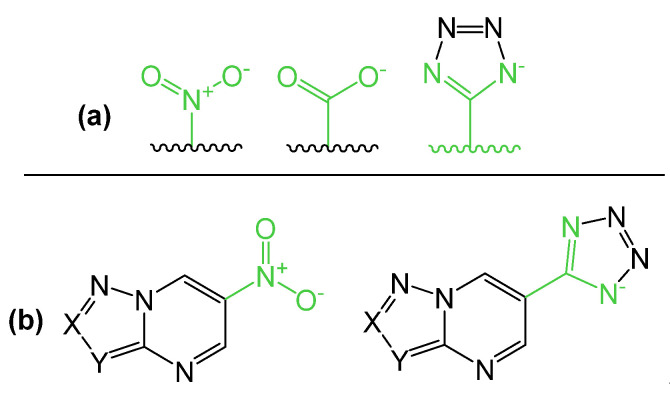
(**a**) Isosterism of tetrazole ring, carboxylic fragment and nitro group. (**b**) Potent nitro-azolopyridimidines and tetrazolyl-containing analogues as perspective alternative.

At the same time, a nitro group or carboxylic fragment should present within heterocyclic scaffold for this useful activity to be formed (Figure 2). On the other hand, the tetrazole cycle is a metabolically stable bio-isostere of the carboxyl group and the *cis*-amide fragment due to the similar electronic structure [21,22,23,24,25]. The corresponding similarity for the carboxylic anion and the nitro group can be noted and one can consider the tetrazolyl fragment as an isostere for both of them (Figure 3a). However, only one example of azoloazines containing tetrazole cycle has been published to date—2-nitro-6-(*1H*-tetrazol-5-yl)-[1,2,4]triazolo[1,5-*a*]pyrimidin-7-amine was considered as nitrogen-rich energetic material [26].

In this work we propose the introduction of tetrazolyl fragment into azolopyrimidine scaffold as promising structural modification to search for novel CK2 inhibitors (Figure 3b).

## 2. Results

### 2.1. Chemistry

**Scheme 1:**
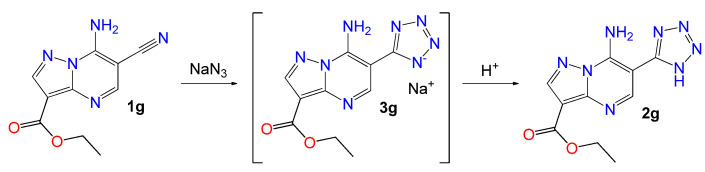
Model reaction of cyanoderivative **1g** with sodium azide for condition optimization.

We have developed a versatile approach to the synthesis of 6-cyano-7-aminoazolo[1,5-*a*]pyrimidines and obtained a library of corresponding heterocycles [27] which are good precursors for azide-nitrile cycloaddition. Herein, 3-Ethoxycarbonyl-6-cyano-7-aminopyrazolo[1,5-*a*]pyrimidine **1g** was used as model substrate to study this process and evaluate different reaction conditions while sodium azide served as the source of the azide fragment (Scheme 1).

The mechanism of this process has been studied extensively by DFT calculations and it was shown that energy barrier for the reaction of the azide anion with nitriles is considerably lower than the barrier for the attack of the neutral hydrazoic acid [28]. At the same time, experimental data revealed that the reaction is strongly accelerated by Brønsted acids such as AcOH and ammonium salts [29,30]. Lewis acids [31], specific organocatalysts [32], and ionic liquids [33] could serve as catalyst in azide-nitrile cycloaddition as well.

It was found that formation of tetrazole cycle by reaction of sodium azide with nitrile derivative **1g** proceeded smoothly in polar aprotic solvents (DMF, MeCN) in the presence of ammonium salts (entry 1–6 and 8, Table 1), AcOH (entry 7, Table 1), ZnCl_2_ (entry 10, Table 1), or 1-butyl-3-methylimidazolium chloride (entry 9, Table 1). The highest yield (78%) of the desired product **2g** was observed in the case AcOH while control experiment where **1g** reacted with sodium azide in DMF without any additive resulted in 82% yield of tetrazole **2g** (entry 11, Table 1). Optimal conditions required heating at 120 ^°^C for 8 h of a 0.25 molar solution of **1g** in DMF with 1.1 equiv. of NaN_3_ and further treatment of water suspension of **3g** with conc. HCl to provide 90% yield of **2g** (entry 13, Table 1). It is worth noting that the formation of the tetrazole cycle was not observed in protic polar solvents (H2O, MeOH) both with catalysis (entry 2 and 6, Table 1) and without (entry 18, Table 1) by TLC analysis of the reaction mixture as well as by NMR analysis of the isolated products.

**Scheme 2:**
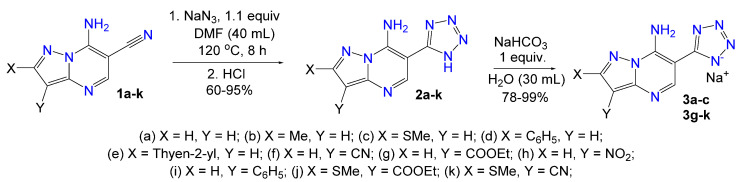
Scope of 6-(tetrazol-5-yl)-7-aminopyrazolo[1,5-*a*]pyrimidines **2a**–**k** and corresponding sodium salts **3a**–**c**, **3g**–**k**.

With the optimized reaction conditions in hand, a series of 6-(tetrazol-5-yl)pyrazolopyrimidines **2a**–**k** were synthesized in good to excellent yields (60–95%) (Scheme 2).

**Scheme 3:**
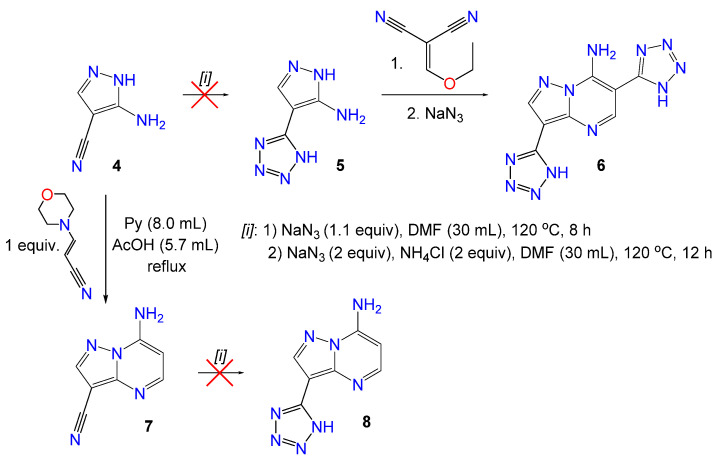
Regioselectivity of nitrile-azide cycloaddition.

The cycloaddition of sodium azide to the C6-nitrile fragment in this series **1a**–**k** proceeded without competing processes. Thus, in the case of dinitriles **1f** and **1k** it was observed that only one cyano group reacted with azide to form tetrazole cycle as there were CN characteristic absorption peaks in the region of 2217–2231 cm^−1^ in IR spectra of the obtained products **2f** and **2k**. The same results were obtained in the reaction of **1f** and **1k** with 3 equiv. of sodium azide by the analysis of reaction products with ^1^H and IR spectroscopy. We have tried to obtain 3,6-di(tetrazol-5-yl)pyrazolopyrimidine **6** by independent synthesis via two steps starting from 3-amino-4-cyanopyrazole **4** (Scheme 3). It was showed that the heating of compound **4** with sodium azide in DMF both with ammonium chloride and in the absence of it did not lead to tetrazolyl heterocycle **5**. Subsequently, pyrazole **4** has been converted to 3-cyano-7-aminopyrazolopyrimidine **7** [34], but the latter also did not react with sodium azide under different conditions and starting material **7** was isolated after workup of the reaction mixture (Scheme 3). These findings support regioselectivity of the azide-nitrile cycloaddition process in the series of 3,6-dicyanopyrazolopyrimidines as only nitrile group in the pyrimidine ring converts into tetrazole fragment.

**Scheme 4:**
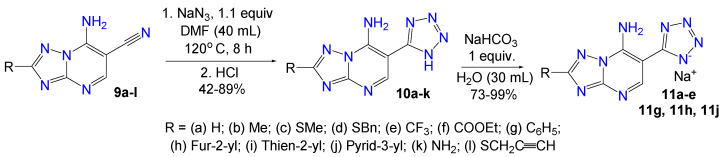
Scope of 6-(tetrazol-5-yl)-7-amino[1,2,4]triazolo[1,5-*a*]pyrimidines **10a**–**k** and corresponding sodium salts **11a**–**e**, **11g**, **11h**, **11j**.

The azide-nitrile cycloaddition proceeded smoothly in the case of [1,2,4]-triazolo[1,5-*a*]pyrimidines **9a**–**k** under optimized conditions and resulted in a series of 2-R-6-(tetrazol-5-yl)-7-amino[1,2,4]triazolo[1,5-*a*]pyrimidines **10a**–**k** with good yields (42–89%) (Scheme 4). The unidentified oily products were isolated when thiopropargyl containing derivative **9l** was introduced in the reaction, probably due to the side azide-alkyne cycloaddition to form 1,2,3-triazole.

The structure of the obtained heterocycles **2a**–**k** and **10a**–**k** was confirmed by ^1^H NMR spectroscopy (the signal of C5H proton was shifted downfield (∆δ ≈ 0.5 ppm) in comparison with starting material), ^13^C NMR technic (characteristic signal around δ ≈ 85–91 ppm, probably, it can be attributed to C6 atom, while other aromatic carbons located in the region of δ ≈ 145–160 ppm), IR spectroscopy (absence of CN absorption peak in the region of 2100–2300 cm^−1^ in comparison with starting material), mass-spectrometry (a molecular ion peaks were detected) and elemental analysis (see Supporting Information).

We converted obtained 6-(*1H*-tetrazol-5-yl)-7-aminoazolo[1,5-*a*]pyrimidines **2** and **10** to the corresponding sodium salts by the reaction with sodium bicarbonate (Scheme 2 and Scheme 4) based on the NH-acidity of the tetrazole ring [35]. These sodium salts **3a**–**c**, **3g**–**k** and **11a–e**, **11g**, **11h**, **11j** possess high water solubility which is an undoubted advantage for testing its biological activity in the CK2 assay and further experiments.

### 2.2. CK2 Inhibition

Once in hand target compounds were evaluated against human recombinant CK2 using luminescent ADP-GloTM platform. Initial screening performed at 50 µM compound concentration revealed scaffold as a rich source of CK2 inhibitors. Confirmation experiments were run in a concentration-dependent manner to obtain IC_50_ values (Table 2).

**Figure 4:**
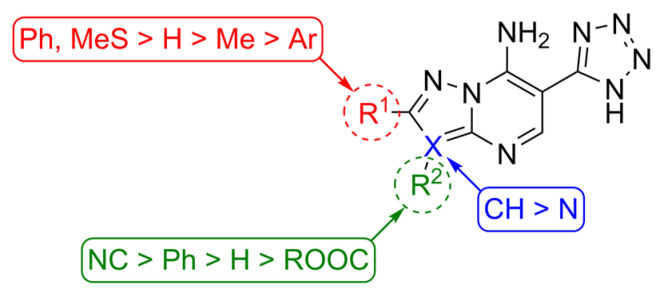
Structure-activity relationship for 6-(tetrazol-5-yl)azolo[1,5-*a*]pyrimidines as CK2 inhibitors.

Structure-activity relationship analysis (Figure 4) suggests that 6-(tetrazol-5-yl)-[1,2,4]triazolo[1,5-*a*]pyrimidines **10a–j** generally have lower activity than corresponding pyrazolopyrimidines **2a**–**k** reflecting in IC_50_ values in higher micromolar range. Notably, in the triazolopyrimidine series, compounds **10a** and **10h** are the most active (IC_50_ 23.78 and 11.81 µM, correspondingly), while any other substituents in the triazole ring resulted in the decrease of affinity towards CK2.

In turn, derivatives of 6-(tetrazol-5-yl)pyrazolo[1,5-*a*]pyrimidine series **2a**–**k** and **3a**–**c**, **3f**, **3g**, **3i** demonstrated rather improved potency. Compounds **2a** and its sodium salt **3a** are micromolar inhibitors. Introduction of SMe (**2c**) or Ph (**2d**) group in the C2-position of heterocyclic scaffold is beneficial, while the smaller Me-substituent at this position led to less active compound **2b**. Evaluation of substituents in position C3 of the pyrazolopyrimidine system indicates non-additive SAR. Thus, both electron-withdrawing and electron-donating groups resulted in low micromolar inhibitors **2f–i** with leader compound **2i** demonstrated IC_50_ = 45 nM. At the same time, the combination of 2-methylsulfanyl group with 3-ethoxycarbonyl or 3-nitrile substituents also revealed compounds **2j** and **2k** with good affinity to CK2. It is worth noting that in most cases sodium salts **3** were surprisingly less active than NH-form of tetrazolyl containing heterocycles **2** excluding potent sodium 5-(7-amino-3-cyanopyrazolo[1,5-*a*]pyrimidin-6-yl)tetrazol-1-ide **3f** with IC_50_ = 65 nM.

## 3. Materials and Methods

### 3.1. Chemistry

Commercial reagents were obtained from Sigma-Aldrich, Acros Organics, or Alfa Aesar and used without any further purification. All workup and purification procedures were carried out using analytical grade solvents. One-dimensional ^1^H, ^19^F, and ^13^C NMR spectra were acquired on a Bruker DRX-400 instrument (400, 376, and 101 MHz, respectively), utilizing DMSO-d_6_ as solvent and as an external reference. The following abbreviations are used for multiplicity of NMR signals: s—singlet, d—doublet, t—triplet, q—quartet, dd—doublet of doublets, dt–doublet of triplets, m—multiplet, br—broaded. Mass spectroscopy studies were performed on a Shimadzu GCMS-QP2010 Ultra (EI, 70 eV). IR spectra were recorded on a Bruker Alpha spectrometer equipped with a ZnSe ATR accessory. Elemental analysis was performed on a PerkinElmer PE 2400 elemental analyzer. Melting points were determined on a Stuart SMP3 and are uncorrected. The monitoring of the reaction progress was performed by using TLC on Silufol UV254 plates (eluent is AcOEt). All synthesized compounds are >95% pure by elemental analysis.

General procedure for the synthesis of 6-(tetrazol-5-yl)-7-aminoazolo[1,5-a]pyrimidines (**2a**–**k, 10a**–**k**).

A suspension of 0.01 mol (1 equiv.) of the corresponding 6-cyano-7-aminoazolo[1,5-a]pyrimidine (1a-k, 9a-k) and 0.011 mol (1.1 equiv.) of sodium azide in 40 mL of DMF was stirred at 120 °C for 8 h under air atmosphere (TLC control, AcOEt as eluent, starting material Rf ≈ 0.6–0.7, tetrazole products Rf ≈ 0.0). The reaction mixture was cooled to 25 °C, evaporated at reduced pressure, residue was dissolved in 30 mL of H_2_O and acidified with conc. HCl to pH≈1. The obtained precipitate was filtered off and washed with 100 mL of H_2_O to give the corresponding product.

6-(1H-tetrazolTetrazol-5-yl)-7-aminopyrazolo[1,5-a]pyrimidine (2a).

Brown solid. Yield 1.67 g, 83%. Mp > 300 °C. 1H NMR (400 MHz, DMSO-d6), δ, ppm. (*J*, Hz): 6.58 (H, d, C3H, *J* = 2.2), 8.24 (H, d, C2H, *J* = 2.2), 8.70 (2H, s, NH2), 8.76 (H, s, C5H). 13C NMR (100 MHz, DMSO-d6), δ, ppm.: 152.7, 148.4, 147.6, 146.0, 145.4, 96.4, 85.7. IR, ν, cm^−1^: 3259 (NH2). MS (EI, 70 eV), *m/z*, (Irel), %: 52 (100), 77 (18), 105 (15), 145 (32), 159 (15), 174 (50), 202 (73), [M]+). Anal. Calcd. for C7H6N8: C 41.59, H 2.99, N 55.42; found: C 41.65, H 3.06, N 55.33.

2-methylMethyl-6-(1H-tetrazol-5-yl)-7-aminopyrazolo[1,5-a]pyrimidine (2b).

Beige solid. Yield 1.84 g, 85%. Mp > 300 °C. 1H NMR (400 MHz, DMSO-d6), δ, ppm. (*J*, Hz): 2.44 (3H, s, CH3), 6.39 (H, s, C3H), 8.61 (2H, s, NH2), 8.69 (H, s, C5H). 13C NMR (100 MHz, DMSO-d6), δ, ppm.: 155.0, 152.6, 148.8, 147.3, 145.5, 96.0, 85.2, 14.4. IR, ν, cm^−1^: 3173 (NH2). MS (EI, 70 eV), *m/z*, (Irel), %: 52 (100), 81 (19), 159 (28), 108 (50), 173 (18), 188 (53), 216 (85), [M]+). Anal. Calcd. for C8H8N8: C 44.44, H 3.73, N 51.83; found: C 44.39, H 3.76, N 51.83.

2-(methylthioMethylthio)-6-(1H-tetrazol-5-yl)-7-aminopyrazolo[1,5-a]pyrimidine (2c).

Brown solid. Yield 1.95 g, 79%. Mp = 273–275 °C. 1H NMR (400 MHz, DMSO-d6), δ, ppm. (*J*, Hz): 2.64 (3H, s, CH3), 6.41 (H, s, C3H), 8.56 (2H, s, NH2), 8.69 (H, s, C5H). 13C NMR (100 MHz, DMSO-d6), δ, ppm.: 156.0, 152.4, 149.1, 147.8, 144.9, 94.5, 85.5, 13.9. IR, ν, cm^−1^: 3238 (NH2). MS (EI, 70 eV), *m/z*, (Irel), %: 52 (70), 131 (20), 159 (16), 173 (87), 220 (42), 248 (100), [M]+). Anal. Calcd. for C8H8N8S: C 38.70, H 3.25, N 45.14; found: C 38.79, H 3.11, N 45.23.

2-phenylPhenyl-6-(1H-tetrazol-5-yl)-7-aminopyrazolo[1,5-a]pyrimidine (2d). 

Yellow solid. Yield 2.64 g, 95%. Mp = 291–293 °C. 1H NMR (400 MHz, DMSO-d6), δ, ppm. (*J*, Hz): 6.95 (H, s, C2H), 7.41 (H, t, C4’H, *J* = 7.6), 7.48 (2H, t, C3’H, C5’H, *J* = 7.6), 8.07 (2H, d, C2’H, C6’H, *J* = 7.6), 8.64 (2H, s, NH2), 8.76 (H, s, C5H). 13C NMR (100 MHz, DMSO-d6), δ, ppm.: 155.6, 152.4, 149.1, 147.6, 145.9, 132.2, 129.3, 128.8, 126.4, 93.4, 85.9. IR, ν, cm^−1^: 3280 (NH2). MS (EI, 70 eV), *m/z*, (Irel), %: 52 (38), 77 (100), 116 (32), 170 (10), 208 (8), 221 (23), 250 (65), 278 (92), [M]+). Anal. Calcd. for C13H10N8: C 56.11, H 3.62, N 40.27; found: C 56.03, H 3.62, N 40.23.

2-(thiophenThiophen-2-yl)-6-(1H-tetrazol-5-yl)-7-aminopyrazolo[1,5-a]pyrimidine (2e). 

Brown solid. Yield 2.56 g, 90%. Mp = 297–298 °C. 1H NMR (400 MHz, DMSO-d6), δ, ppm. (*J*, Hz): 6.83 (H, s, C3H), 7.15 (H, dd, C4’H, *J*_1_ = 5.0, *J*_2_ = 3.5), 7.54 (H, d, C3’H, *J* = 3.5), 7.68 (H, d, C5’H, *J* = 3.5), 8.56 (2H, s, NH2), 8.74 (H, s, C5H). 13C NMR (100 MHz, DMSO-d6), δ, ppm.: 152.4, 151.3, 149.3, 148.0, 145.6, 135.2, 128.1, 127.5, 127.2, 93.3, 86.0. IR, ν, cm^−1^: 3348 (NH2). MS (EI, 70 eV), *m/z*, (Irel), %: 52(55), 93 (25), 148 (29), 227 (23), 241 (17), 256(70), 284 (100), [M]+). Anal. Calcd. for C11H8N8S: C 46.47, H 2.84, N 39.41; found: C 46.47, H 2.90, N 39.41.

3-carbonitrileCarbonitrile-6-(1H-tetrazol-5-yl)-7-aminopyrazolo[1,5-a]pyrimidine (2f).

Brown solid. Yield 1.93 g, 82%. Mp > 300 °C. 1H NMR (400 MHz, DMSO-d6), δ, ppm. (*J*, Hz): 8.64 (H, s, C2H), 8.83 (H, s, NH), 8.97 (H, s, C5H), 9.35 (H, s, NH). 13C NMR (100 MHz, DMSO-d6), δ, ppm.: 152.2, 150.7, 150.6, 147.6, 146.6, 113.5, 89.4, 80.8. IR, ν, cm^−1^: 2231 (CN); 3321 (NH2). MS (EI, 70 eV), *m/z*, (Irel), %: 52 (100), 144 (92), 170 (18), 184 (15), 199 (52), 227 (63), [M]+). Anal. Calcd. for C8H5N9x1/2H2O: C 40.67, H 2.54, N 53.39; found: C 40.75, H 2.52, N 53.38.

3-ethoxycarbonylEthoxycarbonyl-6-(1H-tetrazol-5-yl)-7-aminopyrazolo[1,5-a]pyrimidine (2g). 

Beige solid. Yield 2.54 g, 90%. Mp = 288–290 °C. 1H NMR (400 MHz, DMSO-d6), δ, ppm. (*J*, Hz): 1.36 (3H, t, CH3, *J* = 7.2), 4.30 (2H, q, CH2, *J* = 7.2), 8.44 (H, s, C2H), 8.70 (H, s, NH), 9.06 (H, s, C5H), 9.12 (H, s, NH). 13C NMR (100 MHz, DMSO-d6), δ, ppm.: 161.9, 153.8, 150.1, 147.5, 147.0, 146.0, 101.2, 91.1, 59.4, 14.5. IR, ν, cm^−1^: 1683 (COOEt); 3315 (NH2). MS (EI, 70 eV), *m/z*, (Irel), %: 52 (100), 94 (70), 159 (100), 202 (46), 230 (27), 246 (11), 274 (50), [M]+). Anal. Calcd. for C10H10N8O2xH2O: C 41.10, H 4.11, N 38.36; found: C 41.00, H 4.15, N 38.37.

3-nitroNitro-6-(1H-tetrazol-5-yl)-7-aminopyrazolo[1,5-a]pyrimidine (2h). 

Yellow solid. Yield 1.48 g, 60%. Mp > 300 °C. 1H NMR (400 MHz, DMSO-d6), δ, ppm. (*J*, Hz): 8.93 (H, s, C2H), 8.93 (H, s, NH), 9.08 (H, s, C5H), 9.52 (H, s, NH). 13C NMR (100 MHz, DMSO-d6), δ, ppm.: 152.4, 152.1, 146.6, 143.3, 142.9, 122.4, 91.6. IR, ν, cm^−1^: 1557 (NO2); 1267 (NO2); 3325 (NH2). MS (EI, 70 eV), *m/z*, (Irel), %: 52 (100), 92 (18), 144 (14), 204 (5), 219 (63), 247 (50), [M]+). Anal. Calcd. for C7H5N9O2: C 34.01, H 2.04, N 51.00; found: C 33.98, H 1.89, N 51.13.

3-phenylPhenyl-6-(1H-tetrazol-5-yl)pyrazolo[1,5-a]pyrimidin-7-amine (2i). 

Yellow solid. Yield 1.92 g, 69%. Mp = 277–279 °C. 1H NMR (400 MHz, DMSO-d6), δ, ppm. (*J*, Hz): 7.20 (H, t, C4’H, *J* = 7.8), 7.39 (2H, t, C3’H, C5’H, *J* = 7.8), 8.11 (2H, dd, C2’H, C6’H, *J*_1_ = 7.8, *J*_2_ = 1.2), 8.63 (H, s, C2H), 8.74 (2H, s, NH2), 8.85 (H, s, C5H). 13C NMR (100 MHz, DMSO-d6), δ, ppm.: 152.40, 147.88, 146.17, 144.43, 143.39, 131.98, 128.59, 125.88, 125.60, 109.31, 86.07. IR, ν, cm^−1^: 3292 (NH2). MS (EI, 70 eV), *m/z*, (Irel), %: 52 (50), 142 (25), 235 (11), 250 (42), 278 (100), [M]+). Anal. Calcd. for C13H10N8: C 56.11, H 3.62, N 40.27; found: C 56.02, H 3.66, N 40.26.

2-(methylthioMethylthio)-3-ethoxycarbonlyl-6-(1H-tetrazol-5-yl)-7-aminopyrazolo[1,5-a]pyrimidine (2j).

Yellow solid. Yield 2.91 g, 86%. Mp = 281–284 °C. 1H NMR (400 MHz, DMSO-d6), δ, ppm. (*J*, Hz): 1.30 (3H, t, CH3, *J* = 7.2), 2.64 (3H, s, SCH3), 4.27 (2H, q, CH2, *J* = 7.2), 8.72 (2H, s, NH2), 8.84 (H, s, C5H). 13C NMR (100 MHz, DMSO-d6), δ, ppm.: 162.0, 158.7, 152.2, 149.9, 148.9, 144.9, 98.8, 88.8, 59.5, 14.5, 12.8. IR, ν, cm^−1^: 1639 (COOEt); 3309 (NH2). MS (EI, 70 eV), *m/z*, (Irel), %: 52 (100), 144 (23), 292 (30), 320 (83), [M]+). Anal. Calcd. for C11H12N8O2SxH2O: C 39.05, H 4.14, N 33.14; found: C 38.99, H 4.15, N 33.10.

2-(methylthioMethylthio)-3-carbonitrile-6-(1H-tetrazol-5-yl)-7-aminopyrazolo[1,5-a]pyrimidine (2k).

Orange solid. Yield 2.02 g, 74%. Mp > 300 °C. 1H NMR (400 MHz, DMSO-d6), δ, ppm. (*J*, Hz): 2.75 (3H, s, CH3), 8.85 (H, s, NH), 8.90 (H, s, C5H), 9.01 (H, s, NH). 13C NMR (100 MHz, DMSO-d6), δ, ppm.: 157.4, 153.2, 151.4, 150.3, 145.3, 113.1, 91.6, 78.9, 13.2. IR, ν, cm^−1^: 2217 (CN); 3364 (NH2). MS (EI, 70 eV), *m/z*, (Irel), %: 52 (100), 77 (37), 92 (49), 230 (31), 245 (43), 273 (52), [M]+). Anal. Calcd. for C9H7N9S: C 39.56, H 2.58, N 46.13; found: C 39.56, H 2.60, N 46.23.

6-(1H-tetrazolTetrazol-5-yl)-7-amino-[1,2,4]triazolo[1,5-a]pyrimidine (10a).

Pale yellow solid. Yield 1.50 g, 74%. Mp > 300 °C. 1H NMR (400 MHz, DMSO-d6), δ, ppm. (*J*, Hz): 8.47 (H, s, C2H), 8.82 (s, NH), 9.02 (H, s, C5H), 9.05 (s, NH). 13C NMR (150 MHz, DMSO-d6), δ, ppm.: 155.5, 155.4, 152.4, 146.8, 89.4. IR, ν, cm^−1^: 3322 (NH2). MS (EI, 70 eV), *m/z*, (Irel), %: 52 (98), 146 (32), 160 (10), 175 (100), 203 (60), [M]+). Anal. Calcd. for C6H5N9: C 35.47, H 2.48, N 62.05; found: C 35.55, H 2.38, N 62.01.

2-methylMethyl-6-(1H-tetrazol-5-yl)-7-amino-[1,2,4]triazolo[1,5-a]pyrimidine (10b).

Beige solid. Yield 1.45 g, 64%. Mp > 300 °C. 1H NMR (400 MHz, DMSO-d6), δ, ppm. (*J*, Hz): 3.08 (3H, s, CH3), 9.47 (2H, s, NH2), 9.54 (H, s, C5H). 13C NMR (100 MHz, DMSO-d6), δ, ppm.: 164.5, 155.6, 153.7, 151.6, 145.9, 91.1, 14.8. IR, ν, cm^−1^: 3399 (NH2). MS (EI, 70 eV), *m/z*, (Irel), %: 52 (55), 83 (15), 174 (6), 189 (50), 217 (33), [M]+). Anal. Calcd. for C6H5N9x1/2H2O: C 37.17, H 3.54, N 55.75; found: C 37.17, H 3.55, N 55.71.

2-(methylthioMethylthio)-6-(1H-tetrazol-5-yl)-7-amino-[1,2,4]triazolo[1,5-a]pyrimidine (10c).

Beige solid. Yield 1.94 g, 78%. Mp = 275–280 °C. 1H NMR (600 MHz, DMSO-d6), δ, ppm. (*J*, Hz): 2.7 (3H, s, CH3), 8.7 (H, s, NH), 8.91 (H, s, C5H), 8.96 (H, s, NH). 13C NMR (100 MHz, DMSO-d6), δ, ppm.: 166.9, 155.7, 152.0, 152.0, 145.6, 89.2, 13.2. IR, ν, cm^−1^: 3404 (NH2). MS (EI, 70 eV), *m/z*, (Irel), %: 52 (95), 176 (20), 192 (8), 221 (66), 249 (100), [M]+). Anal. Calcd. for C7H7N9S: C 33.73, H 2.83, N 50.58; found: C 33.77, H 2.81, N 50.53.

2-(benzylthioBenzylthio)-6-(1H-tetrazol-5-yl)-7-amino-[1,2,4]triazolo[1,5-a]pyrimidine (10d).

Yellow solid. Yield 2.76 g, 85%. Mp = 263–266 °C. 1H NMR (400 MHz, DMSO-d6), δ, ppm. (*J*, Hz): 4.55 (2H, s, CH2), 7.25 (H, t, C4’H, *J* = 6.4), 7.32 (2H, t, C3’H, C5H, *J* = 6.4), 7.51 (2H, d, C2’H, C6’H, *J* = 7.2), 8.86 (2H, s, NH2), 8.90 (H, s, C5H). 13C NMR (100 MHz, DMSO-d6), δ, ppm.: 165.9, 155.8, 152.4, 152.1, 145.7, 137.7, 129.0, 128.5, 127.3, 89.9, 34.6. IR, ν, cm^−1^: 3323 (NH2). MS (EI, 70 eV), *m/z*, (Irel), %: 52 (10), 91 (100), 282 (3), 325 (20), [M]+). Anal. Calcd. for C13H11N9S: C 47.99, H 3.41, N 38.75; found: C 47.98, H 3.40, N 38.83.

6-(1H-tetrazolTetrazol-5-yl)-2-(trifluoromethyl)-7-amino-[1,2,4]triazolo[1,5-a]pyrimidine (10e). 

Orange solid. Yield 1.14 g, 42%. Mp > 300 °C. 1H NMR (400 MHz, DMSO-d6), δ, ppm. (*J*, Hz): 8.89 (H, s, NH), 9.12 (H, s, C5H), 9.67 (H, s, NH). 19F NMR (376 MHz, DMSO-d6), δ, ppm.: −64.96. 13C NMR (100 MHz, DMSO-d6), δ, ppm. (*J*, Hz): 155.9, 155.0, 154.0 (q, *J* = 38.6), 152.2, 147.5, 119.3 (q, *J* = 269.6), 91.1. IR, ν, cm^−1^: 3383 (NH2). MS (EI, 70 eV), *m/z*, (Irel), %: 52 (100), 69 (50), 214 (12), 243 (80), 271 (30), [M]+). Anal. Calcd. for C7H4F3N9S: C 31.01, H 1.49, N 21.02; found: C 30.90, H 1.33, N 21.19.

2-ethoxycarbonylEthoxycarbonyl-6-(1H-tetrazol-5-yl)-7-amino-[1,2,4]triazolo[1,5-a]pyrimidine (10f). 

Yellow solid. Yield 1.70 g, 58%. Mp = 226–228 °C. 1H NMR (400 MHz, DMSO-d6), δ, ppm. (*J*, Hz): 1.44 (3H, t, CH3, *J* = 7.2), 4.46 (2H, q, CH2, *J* = 7.2), 8.86 (H, s, NH), 9.08 (H, s, C5H), 9.59 (H, s, NH). 13C NMR (100 MHz, DMSO-d6), δ, ppm.: 159.7, 156.2, 155.6, 153.5, 152.3, 147.2, 90.3, 61.8, 14.1. IR, ν, cm^−1^: 1650 (COOEt); 3255 (NH2). MS (EI, 70 eV), *m/z*, (Irel), %: 160 (45), 232 (12), 247 (8), 275 (32), [M]+). Anal. Calcd. for C9H9N9O2xH2O: C 36.86, H 3.75, N 43.00; found: C 36.99, H 3.73, N 43.20.

2-phenylPhenyl-6-(1H-tetrazol-5-yl)-7-amino-[1,2,4]triazolo[1,5-a]pyrimidine (10g).

Yellow solid. Yield 1.73 g, 62%. Mp > 300 °C. 1H NMR (400 MHz, DMSO-d6), δ, ppm. (*J*, Hz): 7.5 (2H, d, *J* = 6.8, C3’ H, C5’H), 7.52 (H, s, C4’H), 8.26 (2H, dd, *J*_1_ = 7.6, *J*_2_ = 2.8, C2’H, C6’H), 8.88 (2H, s, NH2), 8.99 (H, s, C5H). 13C NMR (100 MHz, DMSO-d6), δ, ppm.: 164.1, 156.1, 152.4, 152.2, 146.6, 130.6, 130.3, 128.9, 126.9, 89.2. IR, ν, cm^−1^: 3361 (NH2). MS (EI, 70 eV), *m/z*, (Irel), %: 52 (39), 77 (65), 251 (55), 279 (32), [M]+). Anal. Calcd. for C12H9N9: C 51.61, H 3.25, N 45.14; found: C 51.61, H 3.23, N 45.28.

2-(furanFuran-2-yl)-6-(1H-tetrazol-5-yl)-7-amino-[1,2,4]triazolo[1,5-a]pyrimidine (10h).

Beige solid. Yield 1.67 g, 60%. Mp > 300 °C. 1H NMR (400 MHz, DMSO-d6), δ, ppm. (*J*, Hz): 6.65 (H, dd, C4’H, *J* = 2.4), 7.18 (H, d, C3’H, *J* = 3.2), 7.82 (H, s, C5’H), 8.99 (2H, s, NH2), 9.04 (H, s, C5H). 13C NMR (100 MHz, DMSO-d6), δ, ppm.: 157.1, 155.8, 152.6, 152.2, 146.6, 145.6, 145.3, 112.56, 112.2, 89.5. IR, ν, cm^−1^: 3255 (NH2). MS (EI, 70 eV), *m/z*, (Irel), %: 52 (41), 94 (100), 160 (23), 212 (20), 226 (18), 241(65), 269 (65), [M]+). Anal. Calcd. for C10H7N9O2x1/2H2O: C 43.16, H 2.88, N 45.32; found: C 43.15, H 2.92, N 45.31.

2-(thiophenThiophen-2-yl)-6-(1H-tetrazol-5-yl)-7-amino-[1,2,4]triazolo[1,5-a]pyrimidine (10i).

Beige solid. Yield 2.39 g, 83%. Mp > 300 °C. 1H NMR (400 MHz, DMSO-d6), δ, ppm. (*J*, Hz): 7.24 (H, t, C4’H, *J* = 4.4), 7.78 (H, d, C3’H, *J* = 4.8), 7.86 (H, d, C5’H, *J* = 3.6), 8.89 (2H, s, NH2), 8.92 (H, s, C5H). 13C NMR (100 MHz, DMSO-d6), δ, ppm 160.4, 155.9, 152.5, 152.2, 146.4, 133.0, 129.5, 128.4, 128.3, 89.5. IR, ν, cm^−1^: 3250 (NH2). MS (EI, 70 eV), *m/z*, (Irel), %: 52 (53), 110 (100), 228 (15), 242 (33), 257 (48), 285 (52), [M]+). Anal. Calcd. for C10H7N9S: C 42.10, H 2.47, N 44.19; found: C 42.01, H 2.55, N 44.20.

2-(pyridinPyridin-3-yl)-6-(1H-tetrazol-5-yl)-7-amino-[1,2,4]triazolo[1,5-a]pyrimidine (10j). 

Beige solid. Yield 2.49 g, 83%. Mp > 300 °C. 1H NMR (400 MHz, DMSO-d6), δ, ppm. (*J*, Hz): 7.56 (H, t, C5’H, *J* = 6.4), 8.55 (H, d, C6’H, *J* = 7.6), 8.69 (H, s, C4’H), 8.96 (2H, s, NH2), 9.03 (H, s, C5H), 9.41 (H, s, C2’H). 13C NMR (100 MHz, DMSO-d6), δ, ppm.: 161.9, 156.0, 152.6, 152.3, 150.9, 147.5, 146.5, 134.3, 126.2, 123.9, 89.6. IR, υ, cm^−1^: 3240 (NH2). MS (EI, 70 eV), *m/z*, (Irel), %: 52 (63), 105 (100), 223 (12), 237 (74), 252 (53), 280 (35), [M]+). Anal. Calcd. for C11H8N10: C 47.14, H 2.88, N 49.98; found: C 47.16, H 2.87, N 50.00.

6-(1H-tetrazolTetrazol-5-yl)-2,7-diamino-[1,2,4]triazolo[1,5-a]pyrimidine (10k).

Yellow solid. Yield 1.44 g, 66%. Mp > 300 °C. 1H NMR (400 MHz, DMSO-d6), δ, ppm. (*J*, Hz): 6.10 (2H, s, C2-NH2), 8.34 (2H, s, C7-NH2), 8.72 (H, s, C5H). 13C NMR (100 MHz, DMSO-d6), δ, ppm.: 166.5, 155.3, 152.3, 150.3, 144.7, 88.6. IR, ν, cm^−1^: 3331 (NH2); 3425 (NH2). MS (EI, 70 eV), *m/z*, (Irel), %: 52 (35), 120 (40), 161 (7), 175 (13), 190 (21), 218 (18), [M]+). Anal. Calcd. for C6H6N10: C 33.03, H 2.77, N 64.20; found: C 32.90, H 2.77, N 64.38.

General procedure for the synthesis of sodium 5-(7-aminoazolo[1,5-a]pyrimidin-6-yl)tetrazol-1-ides (**3**,**11**).

A suspension of 0.005 mol (1 equiv.) of the corresponding 6-(tetrazol-5-yl)azolo[1,5-a]pyrimidin-7-amines and 0.005 mol (0.42 g, 1 equiv.) of sodium bicarbonate in 30 mL of deionized H_2_O was refluxed for 5 min under air atmosphere. The resulting solution was cooled to 25 °C, evaporated at reduced pressure to dryness to give the corresponding product.

Sodium 5-(7-aminopyrazolo[1,5-*a*]pyrimidin-6-yl)tetrazol-1-ide (**3a**).

Beige solid. Yield 1.19 g, 99%. Mp > 300 °C. ^1^H NMR (400 MHz, DMSO-*d*_6_), δ, ppm. (*J*, Hz): 6.34 (H, d, *J* = 2.0, C3H), 7.76 (H, s, NH), 7.98 (H, d, *J* = 2.0, C2H), 8.91 (H, s, C5H), 9.20 (H, s, NH). ^13^C NMR (100 MHz, DMSO-*d*_6_), δ, ppm.: 157.2, 148.3, 147.8, 144.9, 144.1, 94.7, 92.5. IR, ν, cm^–1^: 3333 (NH_2_). Anal. Calcd. for C_7_H_5_N_8_NaxH2O: C 34.72, H 2.91, N 46.27, found: C 34.72, H 3.00, N 46.39.

Sodium 5-(2-methyl-7-aminopyrazolo[1,5-*a*]pyrimidin-6-yl)tetrazol-1-ide (**3b**).

Brown solid. Yield 1.21 g, 95%. Mp > 300 °C. ^1^H NMR (400 MHz, DMSO-*d*_6_), δ, ppm. (*J*, Hz): 2.44 (3H, s, CH_3_), 6.11 (H, s, C3H), 7.52 (H, s, NH), 8.85 (H, s, C5H), 9.13 (H, s, NH). ^13^C NMR (100 MHz, DMSO-*d*_6_), δ, ppm.: 157.3, 153.3, 148.9, 147.4, 144.4, 94.1, 92.3, 14.4. IR, ν, cm^–1^: 3397 (NH_2_). Anal. Calcd. for C_8_H_7_N_8_NaxH2O: C 37.50, H 3.54, N 43.74, found: C 37.55, H 3.59, N 43.66.

Sodium 5-(2-(methylthio)-7-aminopyrazolo[1,5-*a*]pyrimidin-6-yl)tetrazol-1-ide (**3c**).

Brown solid. Yield 1.38 g, 96%. Mp > 300 °C. ^1^H NMR (400 MHz, DMSO-*d*_6_), δ, ppm. (*J*, Hz): 2.62 (3H, s, CH_3_), 6.24 (H, s, C3H), 7.68 (H, s, NH), 8.84 (H, s, C5H), 9.20 (H, s, NH). ^13^C NMR (100 MHz, DMSO-*d*_6_), δ, ppm.: 157.1, 154.0, 149.1, 147.7, 144.0, 92.8, 92.7, 14.1. IR, ν, cm^–1^: 3397 (NH_2_). Anal. Calcd. for C_8_H_7_N_8_NaSxH2O: C 33.33, H 3.15, N 38.87, found: C 33.21, H 3.10, N 38.86.

Sodium 5-(3-cyano-7-aminopyrazolo[1,5-*a*]pyrimidin-6-yl)tetrazol-1-ide (**3f**).

Brown solid. Yield 1.32 g, 99%. Mp > 300 °C. ^1^H NMR (400 MHz, DMSO-*d*_6_), δ, ppm. (*J*, Hz): 8.44 (H, s, C3H), 8.44 (H, s, NH), 9.08 (H, s, C5H), 9.64 (H, s, NH). ^13^C NMR (100 MHz, DMSO-*d*_6_), δ, ppm.: 156.25, 149.81, 149.78, 147.02, 145.81, 114.33, 96.28, 78.88. IR, ν, cm^–1^: 2241 (CN). Anal. Calcd. for C_8_H_4_N_9_NaxH2O: C 35.96, H 2.26, N 47.18, found: C 36.02, H 2.26, N 47.11.

Sodium 5-(3-(ethoxycarbonyl)-7-aminopyrazolo[1,5-*a*]pyrimidin-6-yl)tetrazol-1-ide (**3g**).

Beige solid. Yield 1.55 g, 99%. Mp > 300 °C. ^1^H NMR (400 MHz, DMSO-*d*_6_), δ, ppm. (*J*, Hz): 1.32 (3H, t, *J* = 7.2, CH_3_), 4.28 (2H, q, *J* = 7.2, CH_2_), 8.52 (H, s, C2H), 8.53 (H, s, NH), 9.10 (H, s, C5H), 9.47 (H, s, NH). ^13^C NMR (100 MHz, DMSO-*d*_6_), δ, ppm.: 162.26, 156.43, 149.68, 147.20, 146.58, 145.42, 100.14, 95.67, 59.19, 14.53. IR, ν, cm^−1^: 1608 (COOEt), 3326 (NH_2_). Anal. Calcd. for C_10_H_9_N_8_NaO_2_xH2O: C 38.22, H 3.53, N 35.66, found: C 38.20, H 3.57, N 35.66.

Sodium 5-(3-nitro-7-aminopyrazolo[1,5-*a*]pyrimidin-6-yl)tetrazol-1-ide (**3h**).

Yellow solid. Yield 1.51 g, 99%. Mp > 300 °C. ^1^H NMR (400 MHz, DMSO-*d*_6_), δ, ppm. (*J*, Hz): 8.95 (H, s, C2H), 9.01 (H, s, NH), 9.18 (H, s, C5H), 9.76 (H, s, NH). ^13^C NMR (100 MHz, DMSO-*d*_6_), δ, ppm.: 156.0, 150.9, 145.9, 142.6, 142.5, 121.5, 98.5. IR, ν, cm^−1^: 1373 (NO_2_), 1645 (NO_2_), 3316 (NH_2_). Anal. Calcd. for C_7_H_4_N_9_NaO_2_x2H2O: C 27.55, H 2.64, N 41.31, found: C 27.69, H 2.75, N 41.49.

Sodium 5-(3-phenyl-7-aminopyrazolo[1,5-*a*]pyrimidin-6-yl)tetrazol-1-ide (**3i**).

Yellow solid. Yield 1.42 g, 95%. Mp > 300 °C. ^1^H NMR (400 MHz, DMSO-*d*_6_), δ, ppm. (*J*, Hz): 7.15 (H, t, C4’H, *J* = 7.6), 7.37 (2H, t, C3’H, C5’H, *J* = 7.6), 7.90 (H, s, NH), 8.14 (2H, d, C2’H, C6’H, *J* = 7.6), 8.47 (H, s, C2H), 9.02 (H, s, C5H), 9.32 (H, s, NH). ^13^C NMR (100 MHz, DMSO-*d*_6_), δ, ppm.: 156.94, 147.89, 145.03, 144.95, 144.47, 141.92, 132.89, 128.52, 125.23, 125.20, 107.53, 93.34, 93.30. IR, ν, cm^−1^: 3366 (NH_2_). Anal. Calcd. for C_13_H_9_N_8_Na: C 52.00, H 3.02, N 37.32, found: C 52.03, H 2.99, N 37.20.

Sodium 5-(2-(methylthio)-3-(ethoxycarbonyl)-7-aminopyrazolo[1,5-*a*]pyrimidin-6-yl)tetrazol-1-ide (**3j**).

Beige solid. Yield 1.78 g, 99%. Mp > 300 °C. ^1^H NMR (400 MHz, DMSO-*d*_6_), δ, ppm. (*J*, Hz): 1.40 (3H, t, CH_3_, *J* = 7.2), 2.63 (3H, s, CH_3_), 4.31 (2H, q, CH_2_, *J* = 7.2), 7.99 (H, s, NH_2_), 9.07 (H, s, C5H), 9.50 (H, s, NH_2_). ^13^C NMR (100 MHz, DMSO-*d*_6_), δ, ppm.: 162.5, 157.7, 156.4, 149.2, 148.4, 144.3, 97.1, 95.9, 59.2, 14.6, 12.7. IR, ν, cm^−1^: 1670 (COOEt). Anal. Calcd. for C_11_H_11_N_8_NaO_2_SxH2O: C 36.67, H 3.64, N 31.10, found: C 36.67, H 3.69, N 30.95.

Sodium 5-(2-(methyilthio)-3-cyano-7-aminopyrazolo[1,5-*a*]pyrimidin-6-yl)tetrazol-1-ide (**3k**).

Orange solid. Yield 1.55 g, 99%. Mp > 300 °C. ^1^H NMR (400 MHz, DMSO-*d*_6_), δ, ppm. (*J*, Hz): 2.75 (3H, s, CH_3_), 8.62 (H, s, NH), 8.98 (H, s, CH), 9.58 (H, s, NH). ^13^C NMR (100 MHz, DMSO-*d*_6_), δ, ppm.: 156.8, 156.2, 150.9, 149.5, 145.0, 113.7, 96.6, 77.7, 13.3. IR, ν, cm^−1^: 2224 (CN), 3382 (NH_2_). Anal. Calcd. for C_9_H_6_N_9_NaSxH2O: C 34.51, H 2.57, N 40.24, found: C 34.39, H 2.60, N 40.07.

Sodium 5-(7-amino-[1,2,4]triazolo[1,5-*a*]pyrimidin-6-yl)tetrazol-1-ide (**11a**).

Yellow solid. Yield 1.16 g, 95%. Mp > 300 °C. ^1^H NMR (400 MHz, DMSO-*d*_6_), δ, ppm. (*J*, Hz): 8.49 (H, s, C2H), 8.60 (H, s, NH), 9.13 (H, s, C5H), 9.44 (H, s, NH). ^13^C NMR (100 MHz, DMSO-*d*_6_), δ, ppm.: 156.4, 154.8, 151.3, 145.9, 95.7. IR, ν, cm^−1^: 3342 (NH_2_). Anal. Calcd. for C_6_H_4_N_9_NaxH2O: C 29.64, H 2.49, N 51.84, found: C 29.66, H 2.51, N 51.96.

Sodium 5-(2-methyl-7-amino-[1,2,4]triazolo[1,5-*a*]pyrimidin-6-yl)tetrazol-1-ide (**11b**).

Beige solid. Yield 1.22 g, 95%. Mp > 300 °C. ^1^H NMR (400 MHz, DMSO-*d*_6_), δ, ppm. (*J*, Hz): 2.48 (3H, s, CH_3_), 8.35 (H, s, NH), 9.01 (H, s, C5H), 9.36 (H, s, NH). ^13^C NMR (100 MHz, DMSO-*d*_6_), δ, ppm.: 163.9, 156.5, 155.3, 150.8, 145.4, 95.5, 14.8. IR, ν, cm^−1^: 3364 (NH_2_). Anal. Calcd. for C_7_H_6_N_9_NaxH2O: C 32.69, H 3.14, N 49.01, found: C 32.70, H 3.01, N 49.15.

Sodium 5-(2-(methylthio)-7-amino-[1,2,4]triazolo[1,5-*a*]pyrimidin-6-yl)tetrazol-1-ide (**11c**).

Orange solid. Yield 1.43 g, 99%. Mp > 300 °C. ^1^H NMR (400 MHz, DMSO-*d*_6_), δ, ppm. (*J*, Hz): 2.68 (3H, s, CH_3_), 8.36 (H, s, NH), 9.00 (H, s, C5H), 9.40 (H, s, NH). ^13^C NMR (100 MHz, DMSO-*d*_6_), δ, ppm.: 165.9, 156.3, 155.3, 150.6, 144.9, 96.0, 13.4. IR, ν, cm^−1^: 3376 (NH_2_). Anal. Calcd. for C_7_H_6_N_9_NaSxH2O: C 29.07, H 2.79, N 43.58, found: C 29.09, H 2.66, N 43.63.

Sodium 5-(2-(benzylthio)-7-amino-[1,2,4]triazolo[1,5-*a*]pyrimidin-6-yl)tetrazol-1-ide (**11d**).

Pale yellow solid. Yield 1.65 g, 95%. Mp > 300 °C. ^1^H NMR (400 MHz, DMSO-*d*_6_), δ, ppm. (*J*, Hz): 4.52 (2H, s, CH_2_), 7.22 (H, t, C4’H, *J* = 7.2), 7.29 (2H, t, C3’H, C5’H, *J* = 7.2), 7.48 (2H, d, C2’H, C6’H, *J* = 7.2), 8.23 (H, s, NH), 9.04 (H, s, C5H), 9.43 (H, s, NH). ^13^C NMR (100 MHz, DMSO-*d*_6_), δ, ppm.: 164.8, 156.3, 155.1, 150.6, 144.9, 137.9, 129.0, 128.5, 127.3, 96.2, 34.5. IR, ν, cm^−1^: 3363 (NH_2_). Anal. Calcd. for C_13_H_10_N_9_NaS: C 44.95, H 2.90, N 36.29, found: C 45.01, H 3.05, N 36.27.

Sodium 5-(2-(trifluoromethyl)-7-amino-[1,2,4]triazolo[1,5-*a*]pyrimidin-6-yl)tetrazol-1-ide (**11e**).

Orange solid. Yield 1.45 g, 93%. Mp > 300 °C. ^1^H NMR (400 MHz, DMSO-*d*_6_), δ, ppm. (*J*, Hz): 8.88 (H, s, NH), 9.22 (H, s, C5H), 9.71 (H, s, NH). ^13^C NMR (100 MHz, DMSO-*d*_6_), δ, ppm, (*J*, Hz): 156.1, 155.0, 154.5 (q, *J* = 38.6), 152.5, 146.4, 119.6 (q, *J* = 269.6), 97.6. IR, ν, cm^−1^: 3242 (NH_2_). Anal. Calcd. for C_7_H_3_F_3_N_9_NaxH2O: C 27.02, H 1.62, N 40.51, found: C 26.91, H 1.68, N 40.34.

Sodium 5-(2-phenyl-7-amino-[1,2,4]triazolo[1,5-*a*]pyrimidin-6-yl)tetrazol-1-ide (**11g**).

Yellow solid. Yield 1.43 g, 95%. Mp > 300 °C. ^1^H NMR (400 MHz, DMSO-*d*_6_), δ, ppm. (*J*, Hz): 7.50 (H, m, C4’H), 7.52 (2H, m, C3’H, C5’H), 8.21 (H, s, NH), 8.26 (2H, d, C2’H, C6’H, *J* = 6.8), 9.10 (H, s, C5H), 9.49 (H, s, NH). ^13^C NMR (100 MHz, DMSO-*d*_6_), δ, ppm.: 163.5, 156.5, 155.6, 151.4, 145.7, 130.9, 130.3, 128.9, 126.9, 96.1. IR, ν, cm^−1^: 3258 (NH_2_). Anal. Calcd. for C_12_H_8_N_9_Na: C 47.84, H 2.68, N 41.85, found: C 47.84, H 2.66, N 41.90.

Sodium 5-(2-(furan-2-yl)-7-amino-[1,2,4]triazolo[1,5-*a*]pyrimidin-6-yl)tetrazol-1-ide (**11h**).

Orange solid. Yield 1.48 g, 96%. Mp > 300 °C. ^1^H NMR (400 MHz, DMSO-*d*_6_), δ, ppm. (*J*, Hz): 6.65 (H, dd, C4’H, *J*_1_ = 2.0, *J*_2_ = 1.6), 7.15 (H, d, C3’H, *J* = 2.8), 7.80 (H, s, C5’H), 8.47 (H, s, NH), 9.10 (H, s, C5H), 9.50 (H, s, NH). ^13^C NMR (100 MHz, DMSO-*d*_6_), δ, ppm.: 156.7, 156.4, 155.2, 151.4, 146.2, 145.7, 144.9, 112.1, 111.8, 96.2. IR, ν, cm^−1^: 3228 (NH_2_). Anal. Calcd. for C_10_H_6_N_9_NaxH2O: C 38.84, H 2.61, N 40.77, found: C 38.90, H 2.66, N 40.59.

Sodium 5-(2-(pyridin-3-yl)-7-amino-[1,2,4]triazolo[1,5-*a*]pyrimidin-6-yl)tetrazol-1-ide (**11j**).

Pale yellow solid. Yield 1.49 g, 93%. Mp > 300 °C. ^1^H NMR (400 MHz, DMSO-*d*_6_), δ, ppm. (*J*, Hz): 7.53 (H, dd, C5’H, *J*_1_ = 8.0, *J*_2_ = 3.2), 8.37 (H, s, NH), 8.54 (H, dt, C4’H, *J*_1_ = 8.0, *J*_2_ = 1.6), 8.66 (H, dd, C6’H, *J*_1_ = 3.2, *J*_2_ = 1.6), 9.13 (H, s, C5H), 9.40 (H, d, C2’H, *J* = 2.4), 9.54 (H, s, NH). ^13^C NMR (100 MHz, DMSO-*d*_6_), δ, ppm.: 161.8, 156.8, 156.0, 151.9, 151.5, 148.3, 146.2, 134.6, 127.3, 124.5, 96.8. IR, ν, cm^−1^: 3348 (NH_2_). Anal. Calcd. for C_11_H_7_N_10_NaxH2O: C 41.26, H 2.83, N 43.74, found: C 41.21, H 2.71, N 43.88.

### 3.2. CK2 Assay

Kinase activity was determined using the CK2a1 enzyme system (Promega V4482, Madison, WI, USA) and the ADP-GloTM kit (Promega V9101, Madison, USA) in white 384-well plates (ThermoFisher). The assay was carried out using 10 ng/well of N-GST labeled human recombinant CK2a1 expressed in Sf9 cells, 0.1 µg/µL casein, 10 µM ATP in a 40 mM Tris buffer (pH 7.50) containing 20 mM MgCl_2_, 0.1 mg/mL BSA and 50 µM DTT. Compounds were introduced in 1.25% DMSO and preincubated with kinase at 450 rpm. within 10 min. The reaction was carried out during 60 min. at 25 °C in PST-60HL shaker (Biosan, Latvia). ATP-dependent luminescence was measured at an integration time of 1000 ms using Infinite M200 PRO microplate reader (Tecan GmbH, Grödig, Austria). The experiments were run in two replicates. The activity of CK2 in sample wells was normalized against control and enzyme-blank wells, and IC_50_ values were calculated using 3-parameter log-logistic nonlinear regression with Prism 8.0 (GraphPad Software, San Diego, CA, USA).

## 4. Conclusions

We have explored the chemical space around azolo[1,5-*a*]pyrimidines as a valuable scaffold for the design of potent CK2 inhibitors. Tetrazolyl-containing azolopyrimidines have been proposed as perspective structural analogues of nitroazoloazines with a wide range of useful biological activity. A method for the synthesis of 6-(tetrazol-5-yl)-7-aminoazolo[1,5-*a*]pyrimidines based on azide-nitrile cycloaddition was developed. Optimized conditions allowed us to obtain a library of tetrazolyl-containing azolopyrimidines and screened it for CK2 inhibitory activity. Some SAR have been revealed as azolo[1,5-*a*]pyrimidines of this series, which showed a higher affinity to CK2 then corresponding [1,2,4]triazolo[1,5-*a*]pyrimidines. We have found several low micromolar and nanomolar CK2 inhibitors and leader compound **2i** demonstrated IC_50_ = 45 nM. These findings are going to be used for further optimization of azoloazines as promising bioactive compounds.

## Figures and Tables

**Table 1:** Optimization of reaction parameters in the synthesis of tetrazolyl-pyrazolopyrimidine **2g** ^a^.

№	NaN_3_, Equiv.	Catalyst	Catalyst, Equiv.	Solvent	T, °C	Time, h	Yield of 2g, %
1	1.1	Me_3_N·HCl [29]	1	DMF	100	8	60 ^b^
2	1.1	Me_3_N·HCl	1	MeCN	81	8	15 ^b^
3	1.1	Me_3_N·HCl	1	MeOH	64	8	0
4	1.1	Me_3_N·HCl	1.5	DMF	100	8	60 ^b^
5	1.1	H_3_N·HCl [30]	1	DMF	100	8	44 ^b^
6	1.1	H_3_N·HCl	1	H2O	100	8	0
7	1.1	AcOH [29]	1.2	DMF	100	8	78 ^b^
8	1.2	Bu_4_NBr	0.1	DMF	100	8	70 ^b^
9	1.1	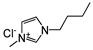 [28]	0.1	DMF	100	8	56 ^b^
10	1.1	ZnCl_2_ [31]	0.15	DMF	100	8	33 ^b^
11	1.1	-	-	DMF	100	8	82 ^b^
12	1.2	-	-	DMF	100	8	80 ^b^
13	1.1	-	-	DMF	120	8	90 ^b^
14	1.1	-	-	DMF	140	8	69 ^b^
15	1.1	-	-	DMF	120	15	89 ^b^
16	1.0	-	-	DMF	120	8	80 ^b^
17	1.1	AcOH	1.2	DMF	120	8	75 ^b^
18	1.1	-	-	H2O	110	9	0
19	1.1	-	-	DMF	120	8	77 ^c^

^a^—0.005 mol of the starting material **1g**, air atmosphere, conventional heating; ^b^—acidification of **3g** by conc. HCl; ^c^—acidification of **3g** by AcOH.

**Table 2:** CK2 inhibition of obtained tetrazolyl-azolopyrimidines **2,3** and their sodium salts **10**,**11**.

Compound	CK2 Inhibition at 50 µM (%)	IC_50_ (µM)	IC_50_ 95% C.I. (µM)
**2a**	90.24 ± 2.83	9.27	4.09–21.03
**3a**	93.69 ± 0.48	21.75	2.94–176.0
**2b**	73.92 ± 1.05	42.10	22.32–78.75
**3b**	77.73 ± 3.62	16.38	4.80–64.19
**2c**	94.04 ± 0.47	4.48	1.11–16.49
**3c**	93.04 ± 0.58	22.6	7.71–72.73
**2d**	89.83 ± 1.65	2.42	0.16–20.35
**2e**	98.19 ± 0.38	3.89	1.22–11.48
**2f**	92.78 ± 4.16	0.18	0.11–0.28
**3f**	99.54 ± 2.53	0.067	0.026–0.176
**2g**	75.51 ± 10.19	9.45	1.35–99.91
**3g**	87.3 ± 1.29	29.91	0.53–5664
**2h**	98.23 ± 1.77	2.33	0.23–26.18
**2i**	100.86 ± 0.83	0.045	0.018–0.243
**3i**	99.26 ± 0.21	0.168	0.060–0.496
**2j**	88.28 ± 1.23	0.253	0.967–7.405
**2k**	89.32 ± 2.51	n.t.	n.t.
**10a**	83.04 ± 1.52	23.78	6.33–102.6
**10b**	56.85 ± 3.69	n.t.	n.t.
**10c**	75.89 ± 2.65	30.41	5.17–203.7
**10d**	43.95 ± 1.73	n.t.	n.t.
**10e**	58.38 ± 4.88	n.t.	n.t.
**10f**	85.18 ± 2.48	182.3	1.6–319.8
**10g**	77.76 ± 1.11	114.8	49.35–302.2
**10h**	85.02 ± 0.64	11.81	0.79–172.9
**10i**	74.83 ± 2.74	44.67	1.29–254.7
**10j**	58.85 ± 10.44	n.t.	n.t.

n.t.—not tested.

## Data Availability

Data are contained within the article.

## Supplementary Materials

The following supporting information can be downloaded at: https://www.mdpi.com/article/10.3390/molecules27248697/s1, NMR, IR, mass spectra of compounds **2**, **3**, **10**, **11**.

## Sample Availability

Samples of the compounds **1**–**3**, **9**, **11** are available from the authors.
