# Supplementary material for: 6-(Tetrazol-5-yl)-7-aminoazolo[1,5-a]pyrimidines as Novel Potent CK2 Inhibitors
Source: Molecules. 2022 Dec 8;27(24):8697. doi: 10.3390/molecules27248697 (PMC9783892; doi:10.3390/molecules27248697)
Supplement: Supplementary file 1 [file molecules-27-08697-s001.zip › molecules-2071609-supplementary.pdf]

6-(1H-tetrazol-5-yl)-7-aminopyrazolo[1,5-a]pyrimidine (2a)

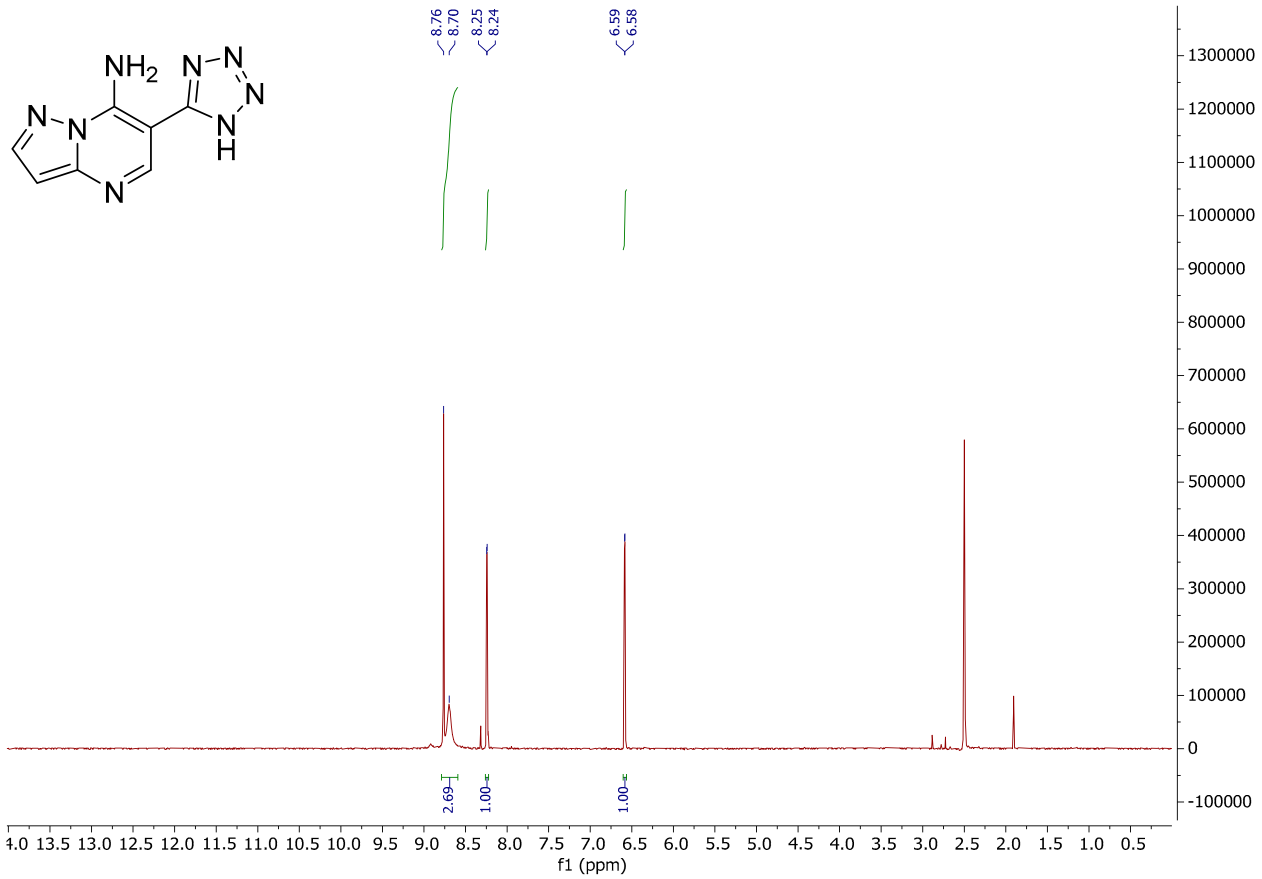

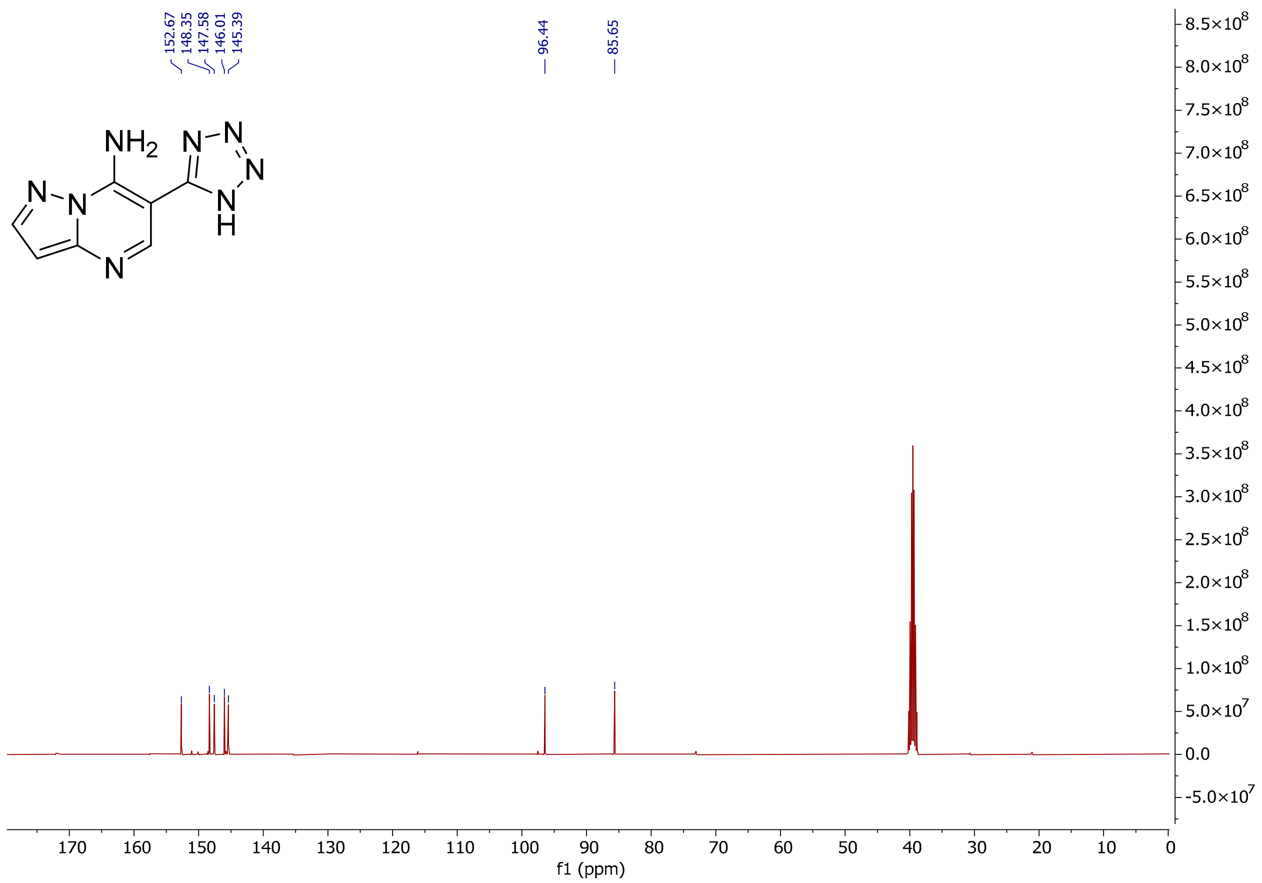

Figure S1. ^{1}H NMR (400 MHz, DMSO-d_6) and ^{13}C NMR (100 MHz, DMSO-d_6) spectra of **2a.**

6-(1H-tetrazol-5-yl)-7-aminopyrazolo[1,5-a]pyrimidine (2a)

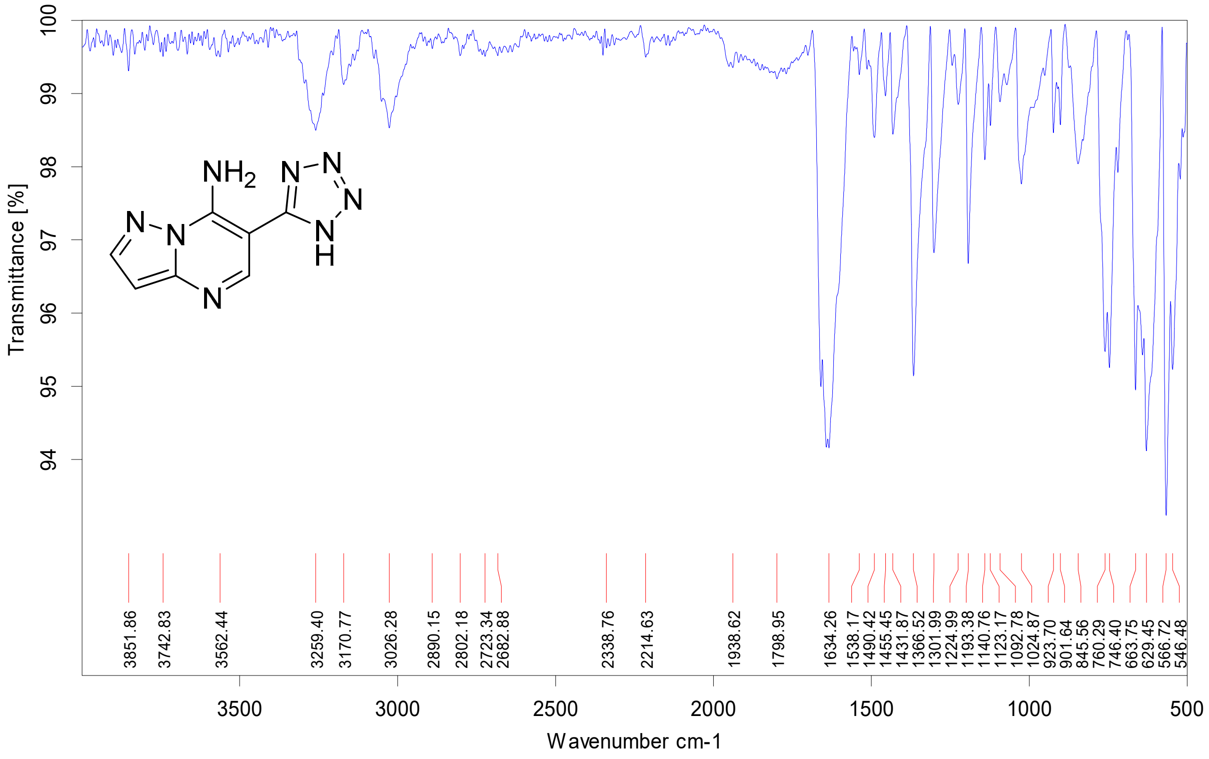

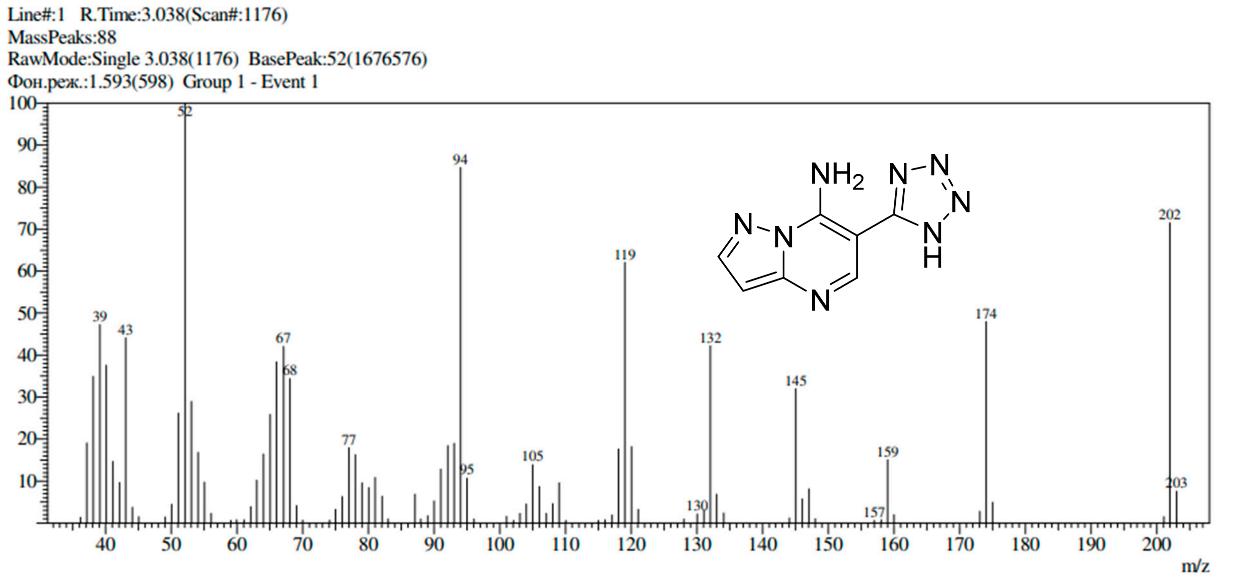

Figure S2. IR and MS (EI, 70 eV) spectra of **2a**

2-methyl-6-(1H-tetrazol-5-yl)-7-aminopyrazolo[1,5-a]pyrimidine (2b)

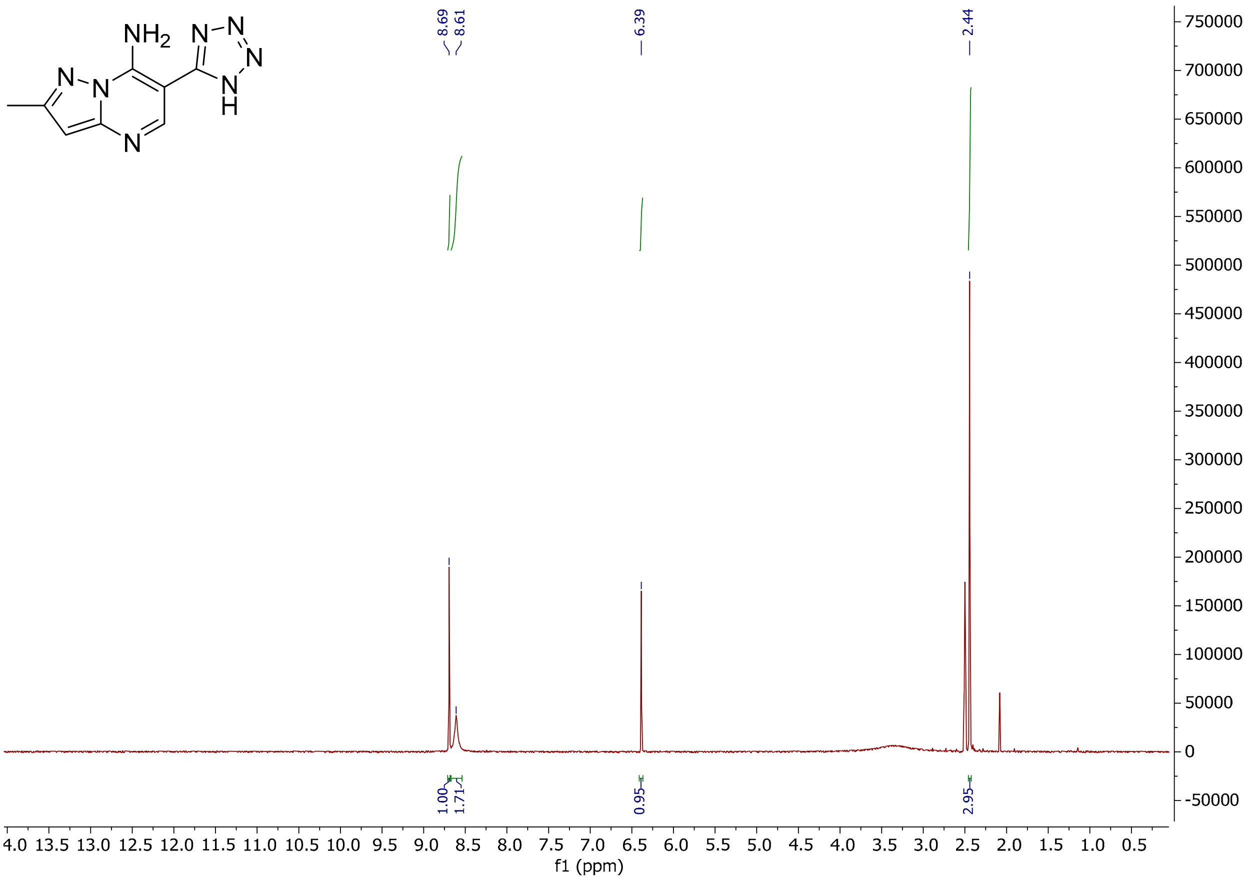

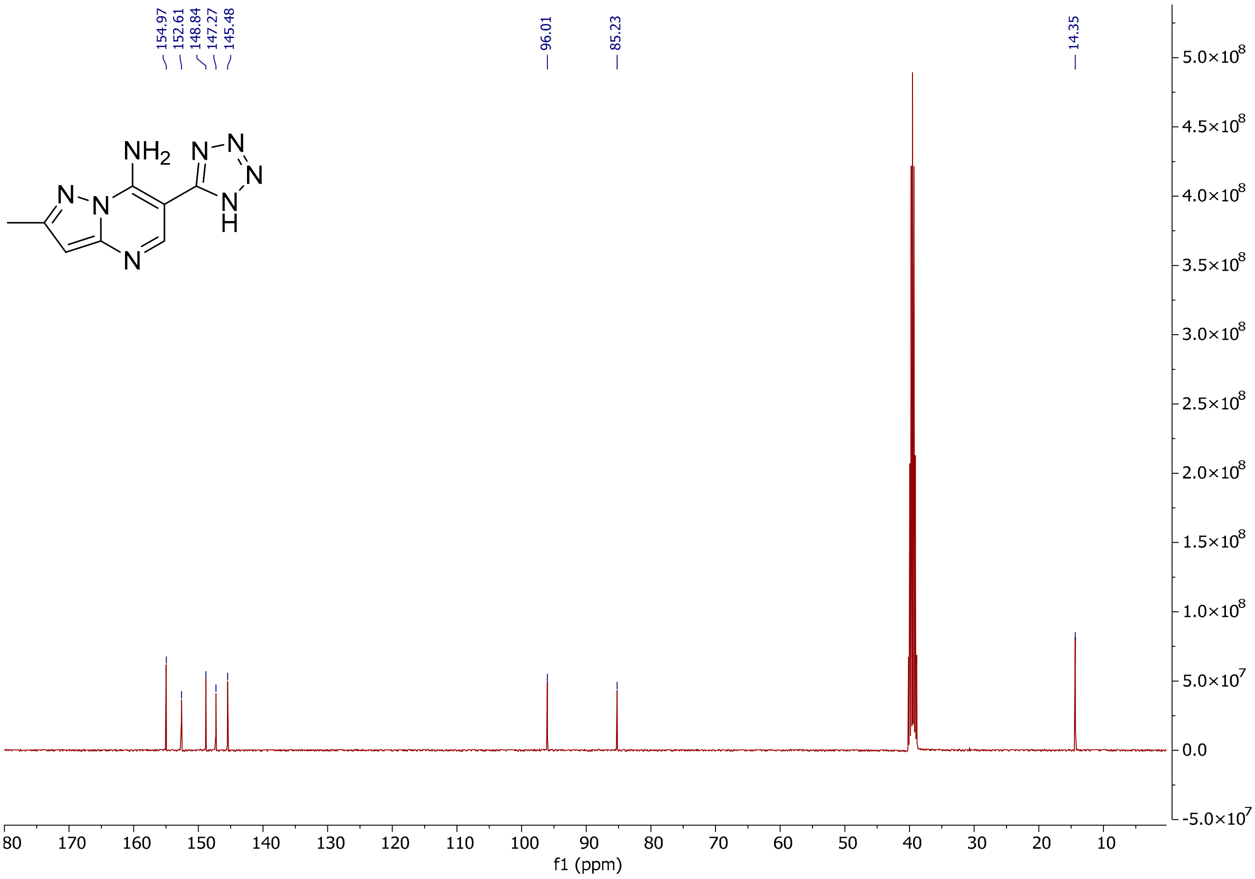

Figure S3. ^{1}H NMR (400 MHz, DMSO-d_6) and ^{13}C NMR (100 MHz, DMSO-d_6) spectra of **2b**

2-methyl-6-(1H-tetrazol-5-yl)-7-aminopyrazolo[1,5-a]pyrimidine (2b)

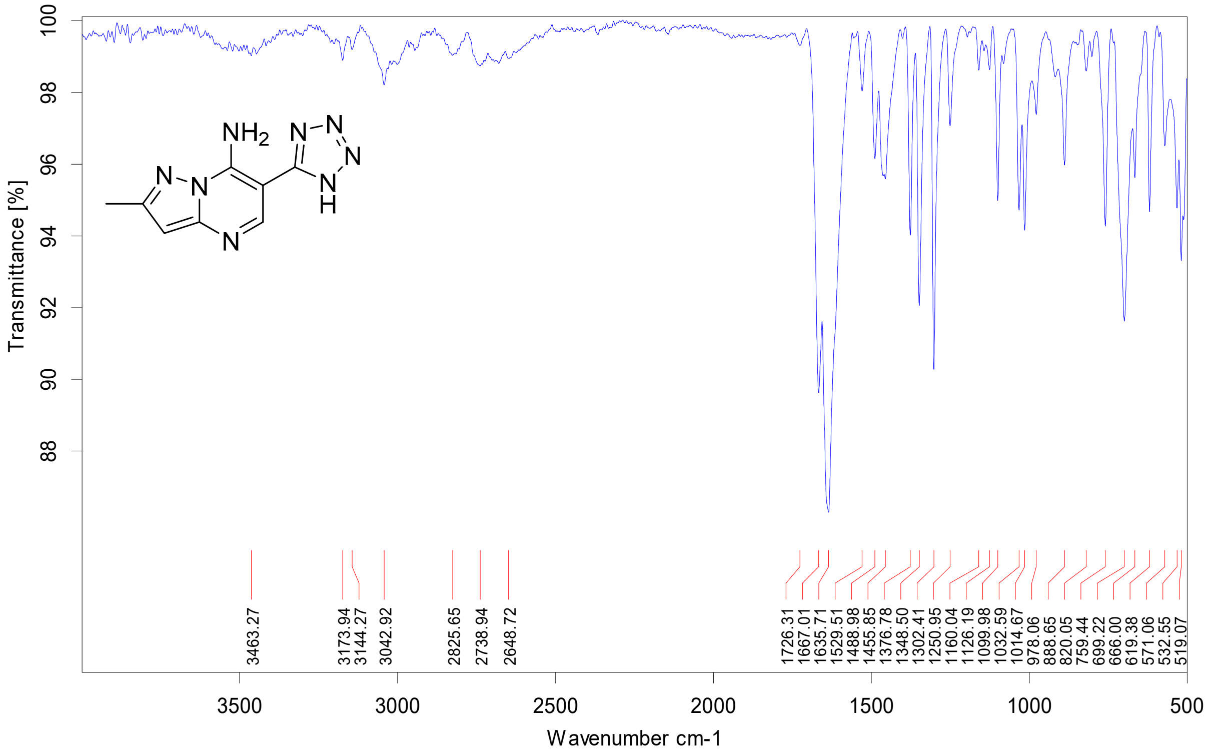

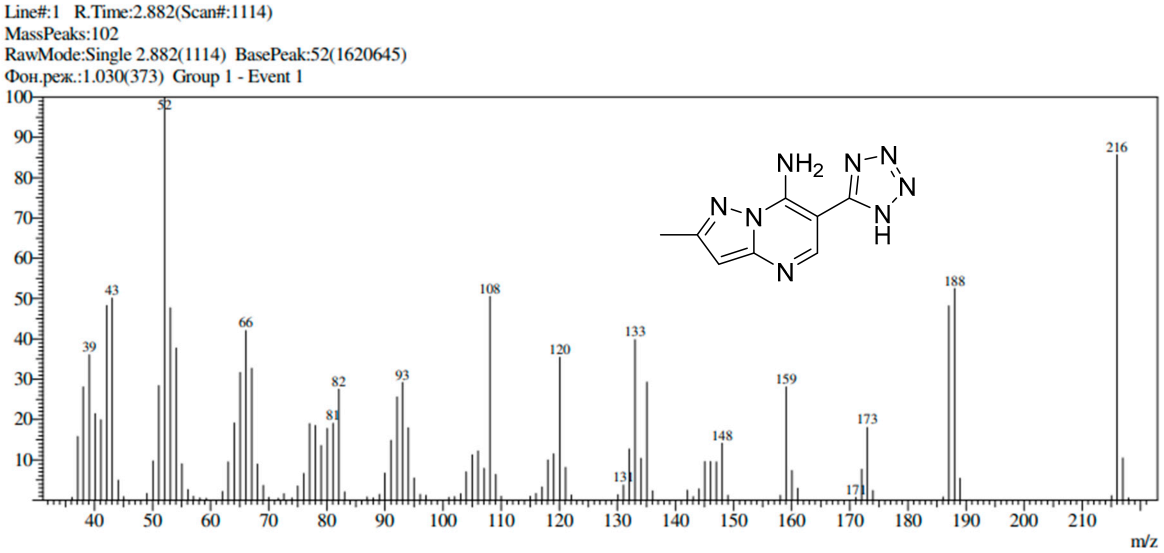

Figure S4. IR and MS (EI, 70 eV) spectra of **2b**

2-(methylthio)-6-(1H-tetrazol-5-yl)-7-aminopyrazolo[1,5-a]pyrimidine (2c)

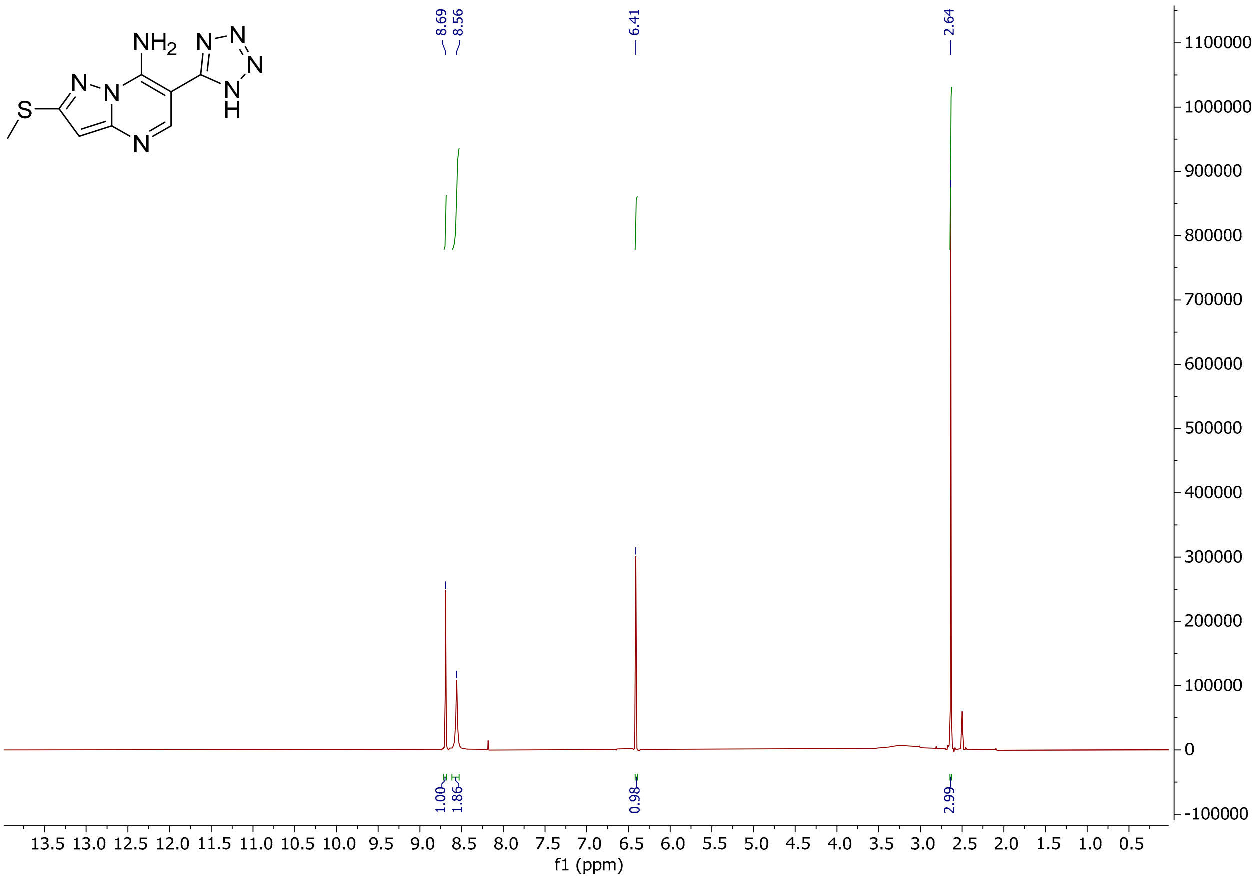

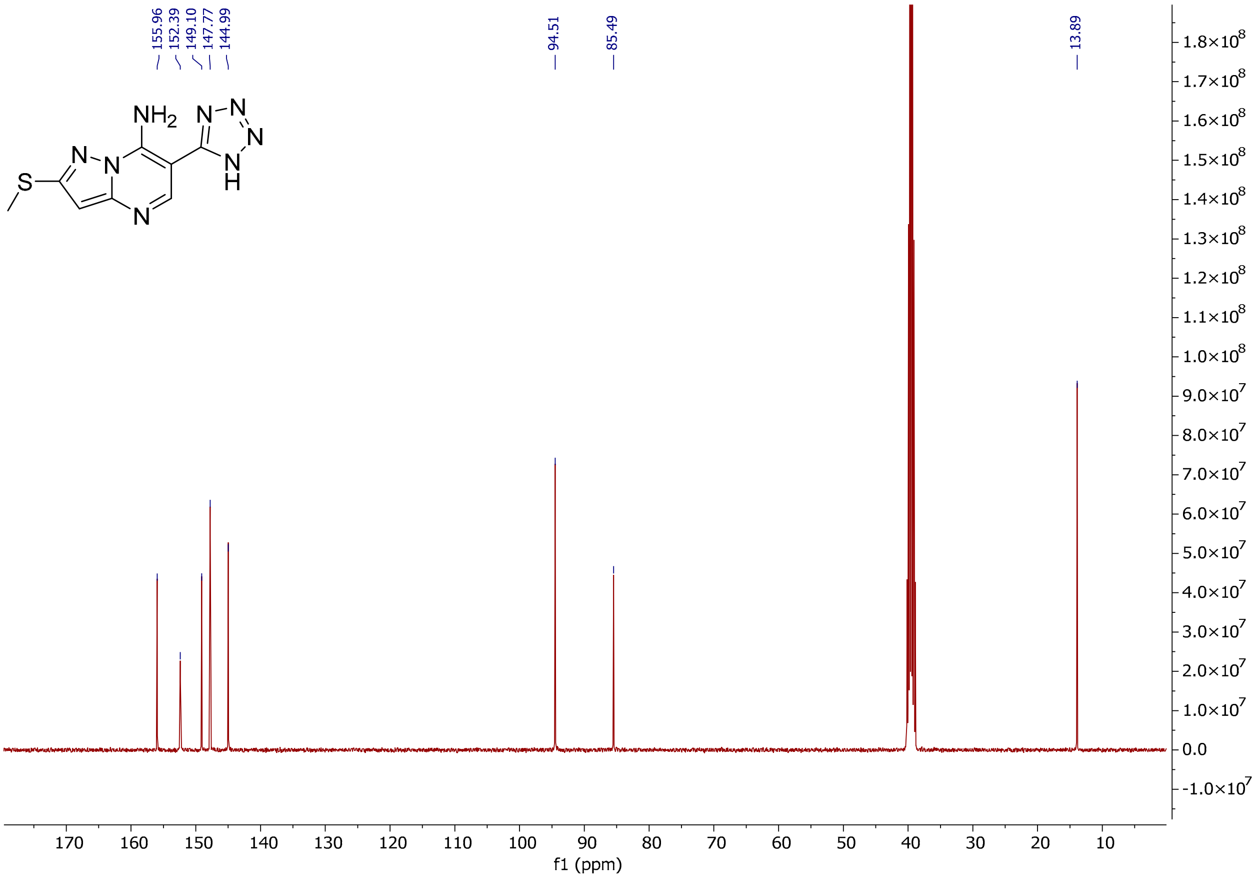

Figure S5. ^{1}H NMR (400 MHz, DMSO-d_6) and ^{13}C NMR (100 MHz, DMSO-d_6) spectra of **2c.**

2-(methylthio)-6-(1H-tetrazol-5-yl)-7-aminopyrazolo[1,5-a]pyrimidine (2c)

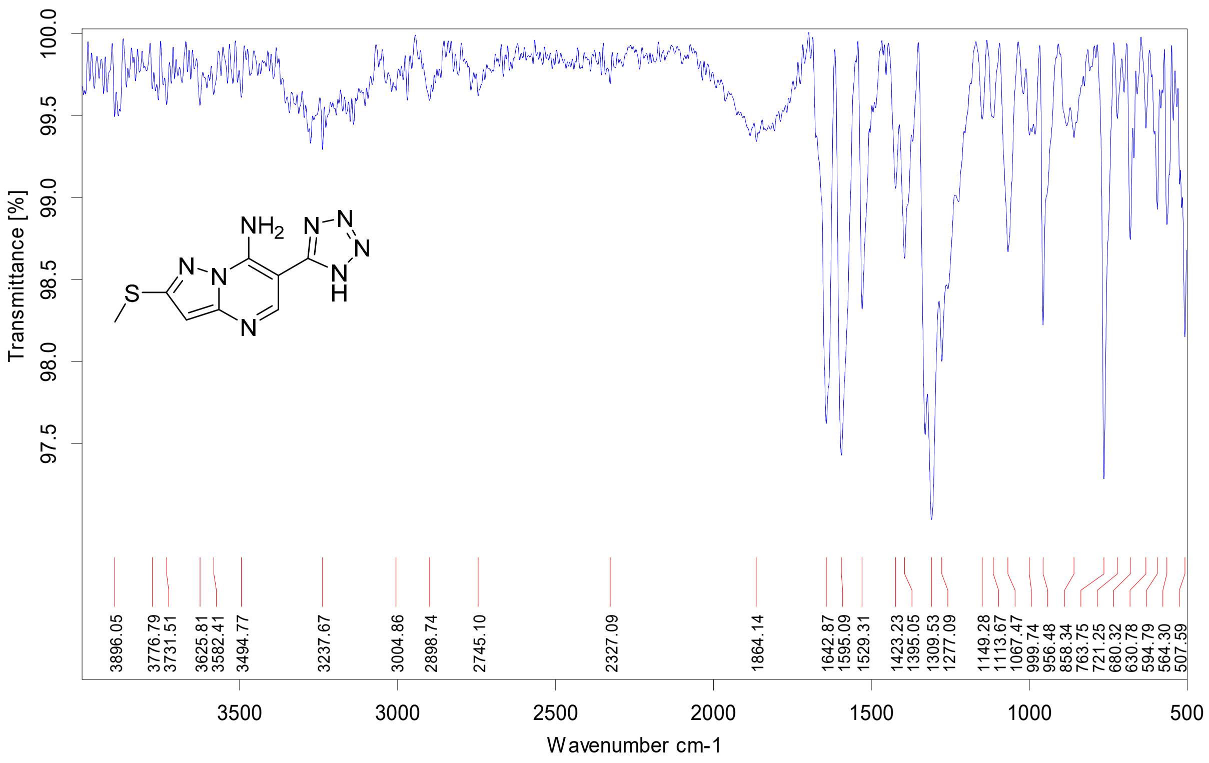

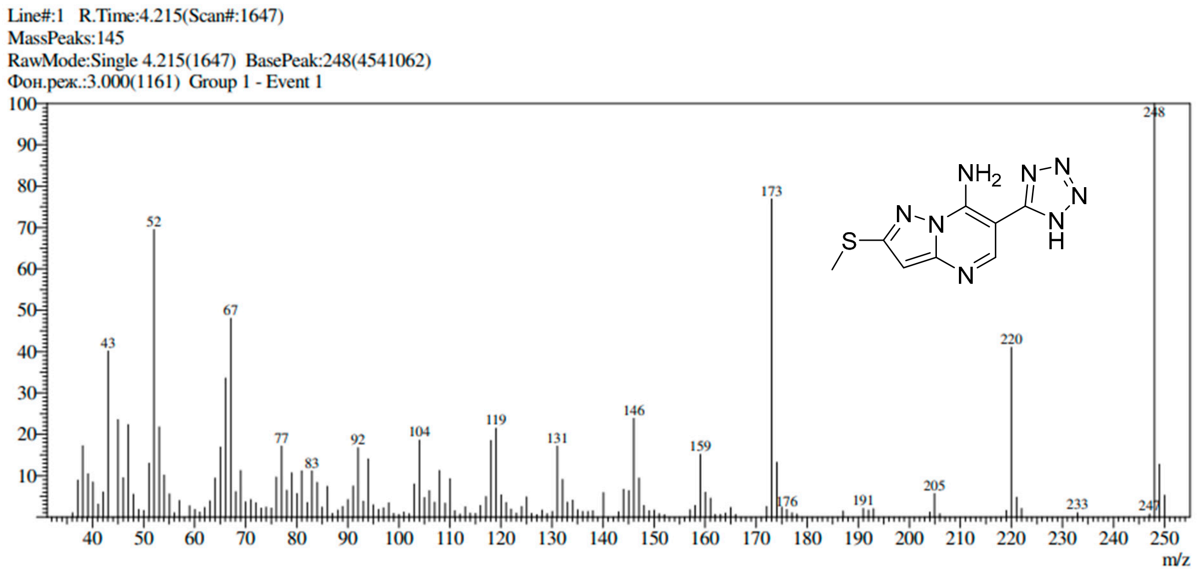

Figure S6. IR and MS (EI, 70 eV) spectra of **2c**

2-phenyl-6-(1H-tetrazol-5-yl)-7-aminopyrazolo[1,5-a]pyrimidine (2d)

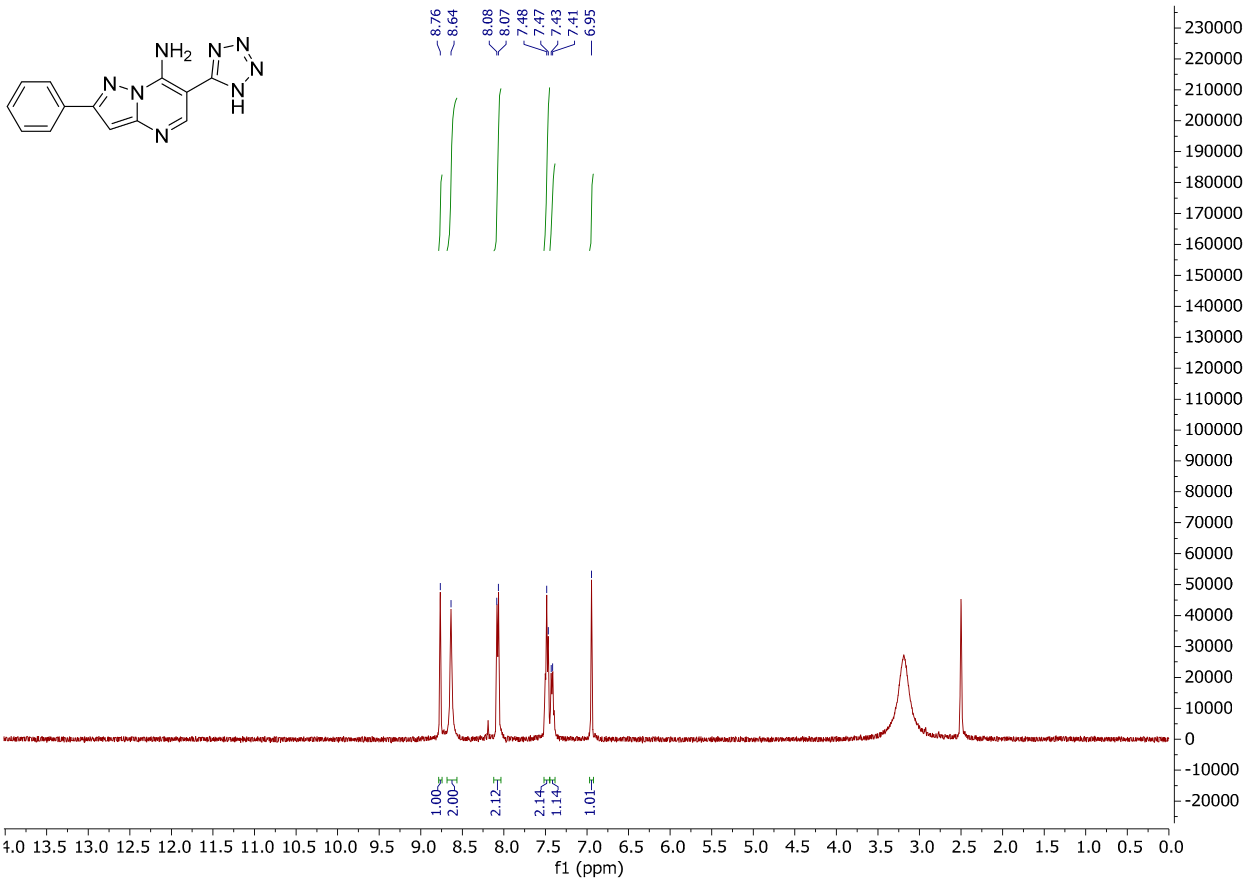

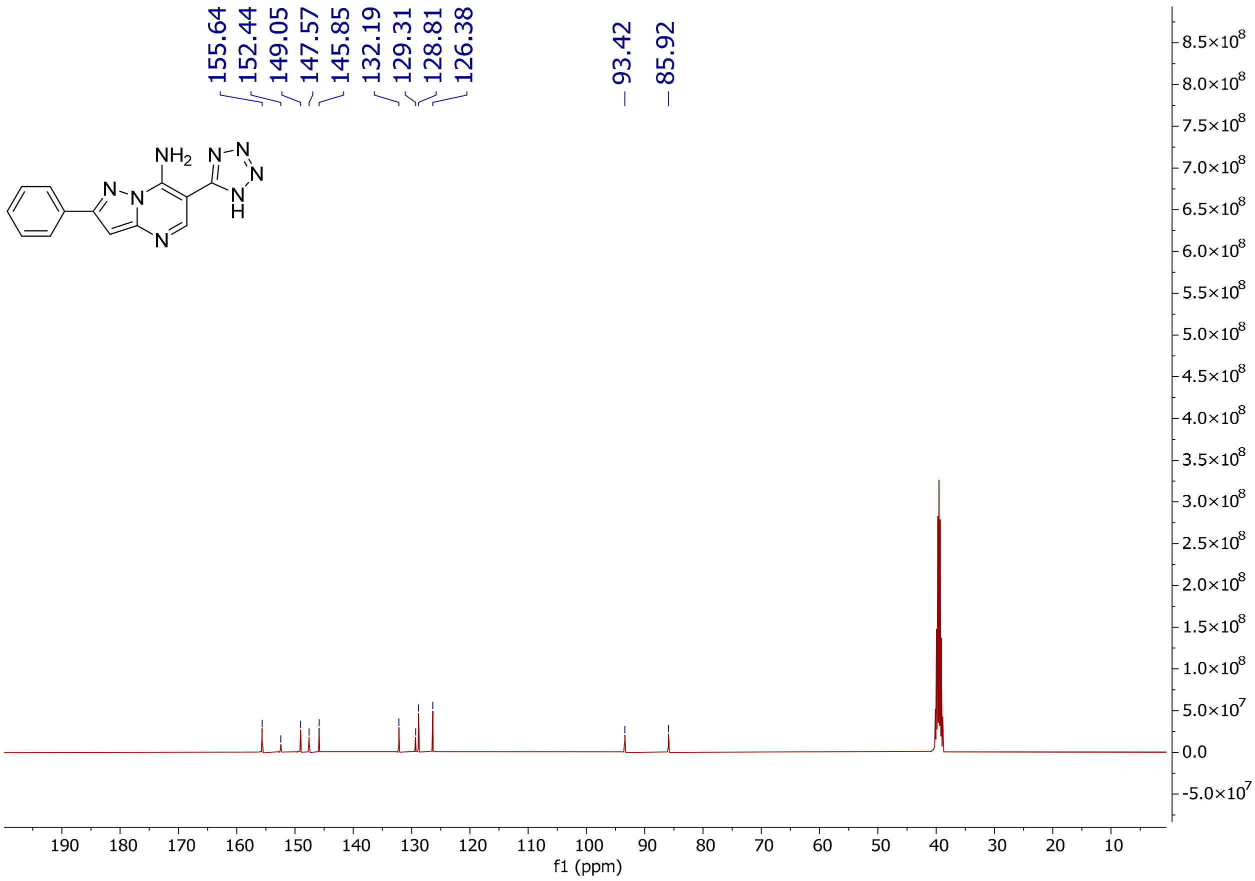

Figure S7. ^{1}H NMR (400 MHz, DMSO-d_6) and ^{13}C NMR (100 MHz, DMSO-d_6) spectra of **2d.**

2-phenyl-6-(1H-tetrazol-5-yl)-7-aminopyrazolo[1,5-a]pyrimidine (2d)

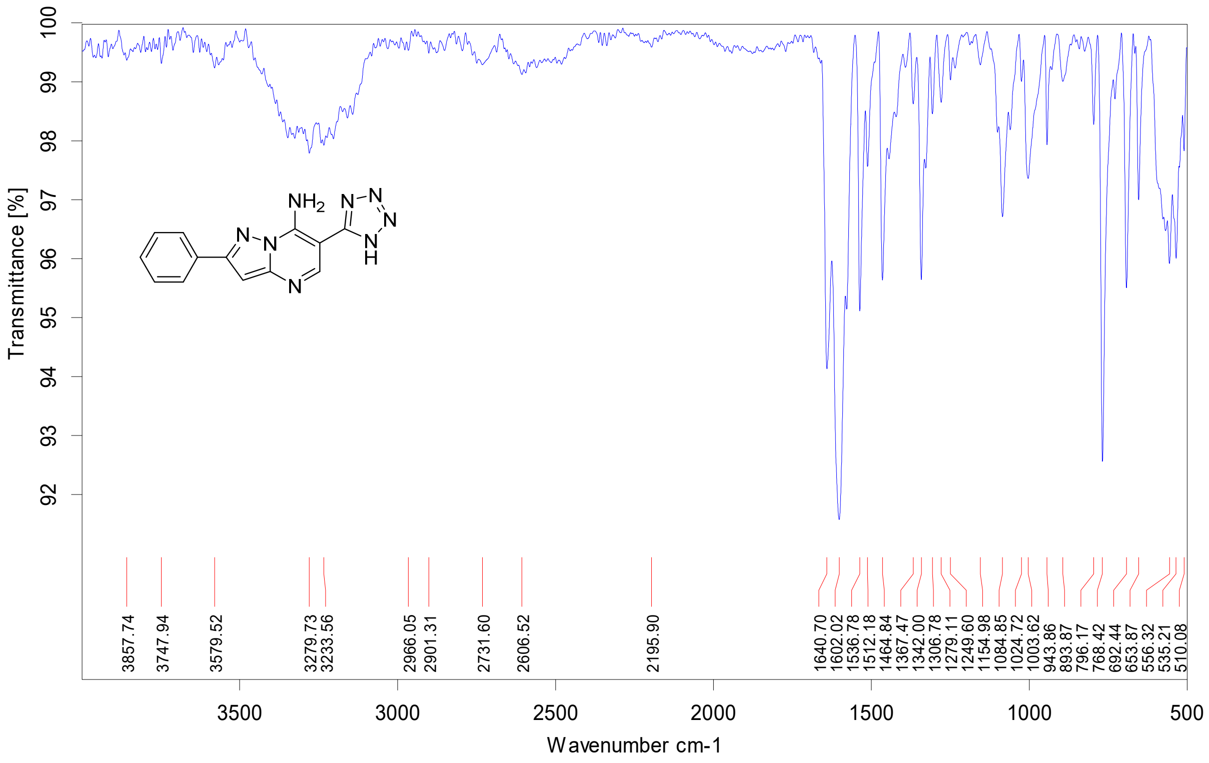

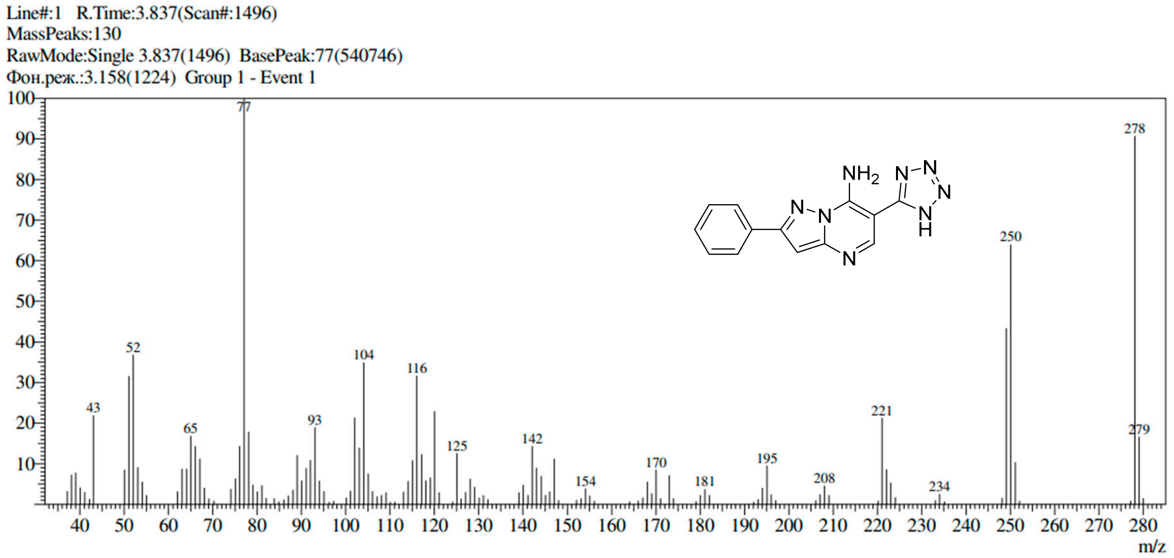

Figure S8. IR and MS (EI, 70 eV) spectra of **2d.**

2-(thiophen-2-yl)-6-(1H-tetrazol-5-yl)-7-aminopyrazolo[1,5-a]pyrimidine (2e)

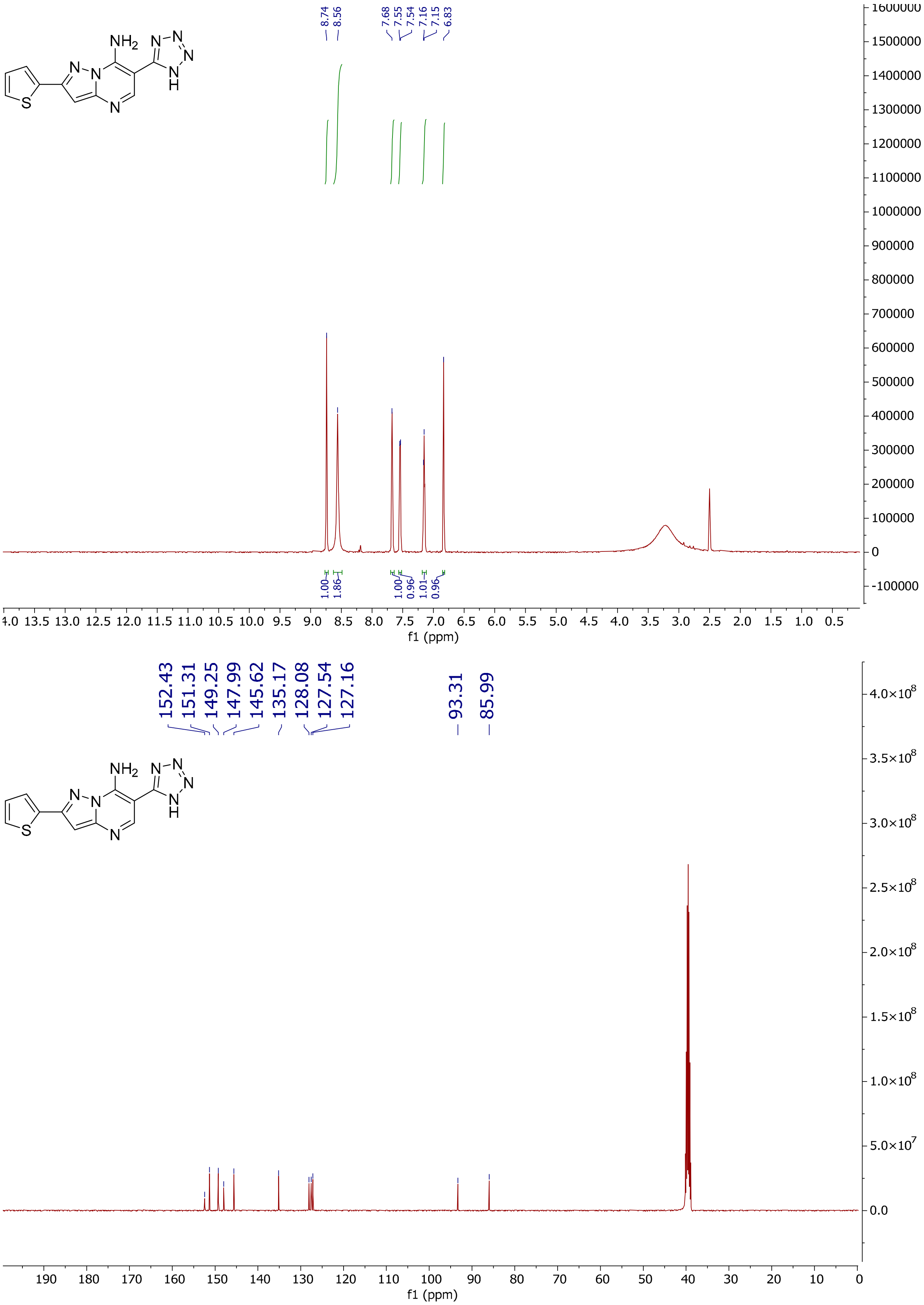

Figure S9. ^{1}H NMR (400 MHz, DMSO-d_6) and ^{13}C NMR (100 MHz, DMSO-d_6) spectra of **2e.**

2-(thiophen-2-yl)-6-(1H-tetrazol-5-yl)-7-aminopyrazolo[1,5-a]pyrimidine (2e)

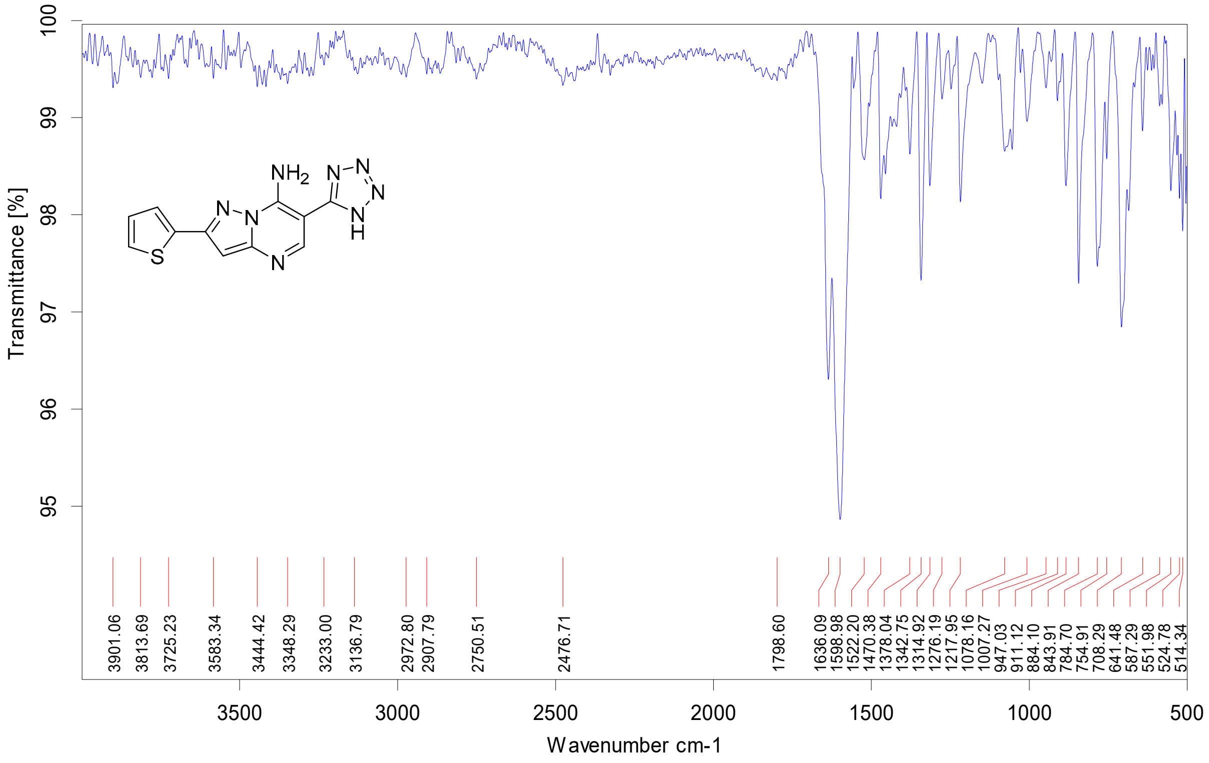

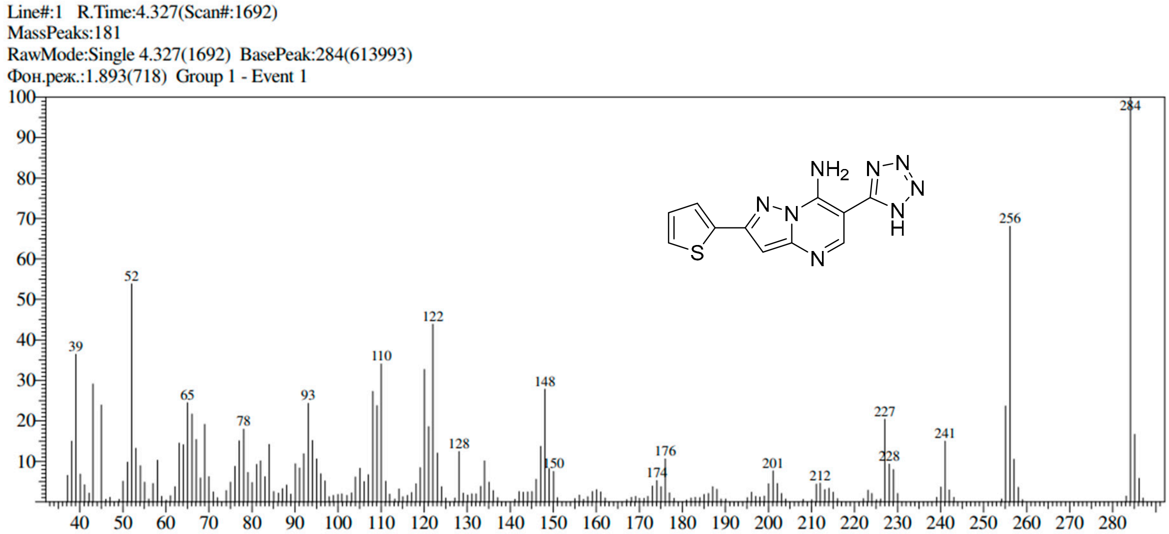

Figure S10. IR and MS (EI, 70 eV) spectra of **2e**

3-carbonitrile-6-(1H-tetrazol-5-yl)-7-aminopyrazolo[1,5-a]pyrimidine (2f)

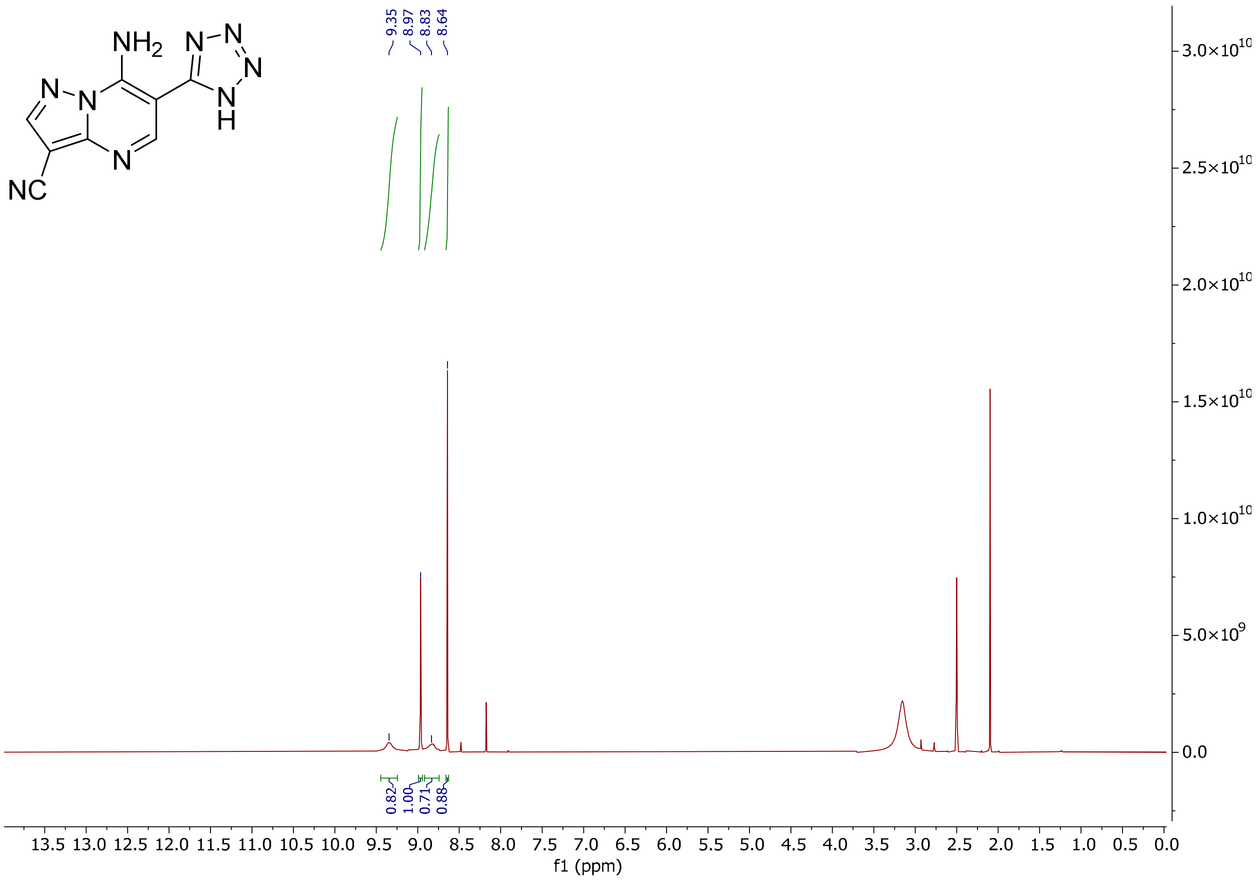

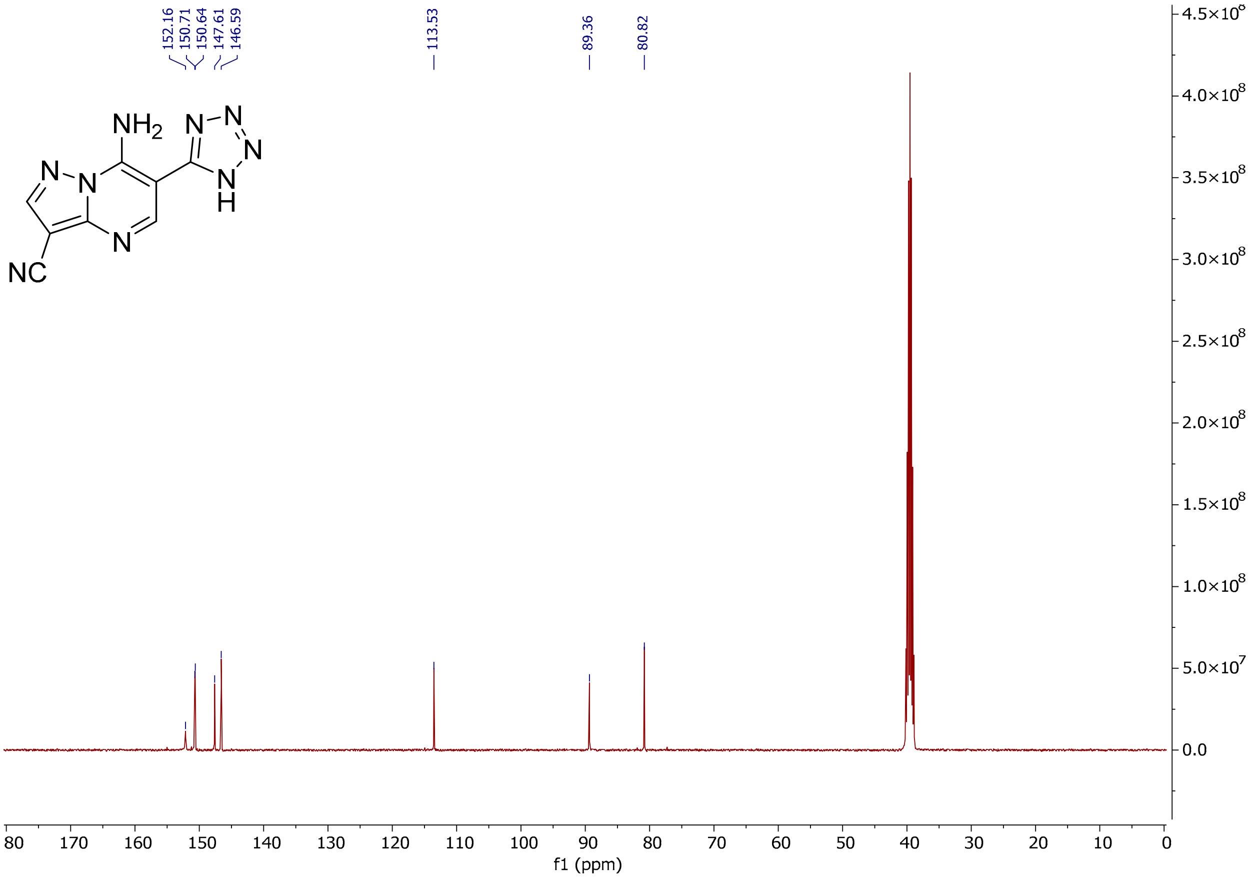

Figure S11. ^{1}H NMR (400 MHz, DMSO-d_6) and ^{13}C NMR (100 MHz, DMSO-d_6) spectra of **2f**

3-carbonitrile-6-(1H-tetrazol-5-yl)-7-aminopyrazolo[1,5-a]pyrimidine (2f)

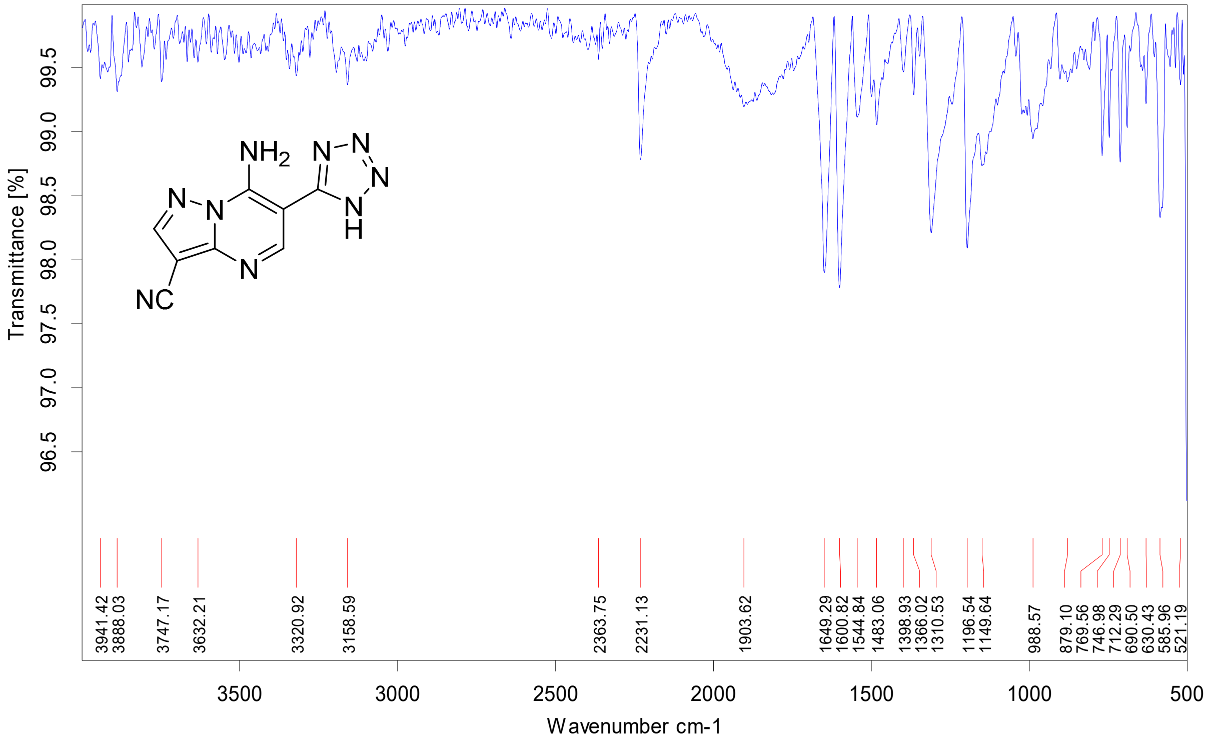

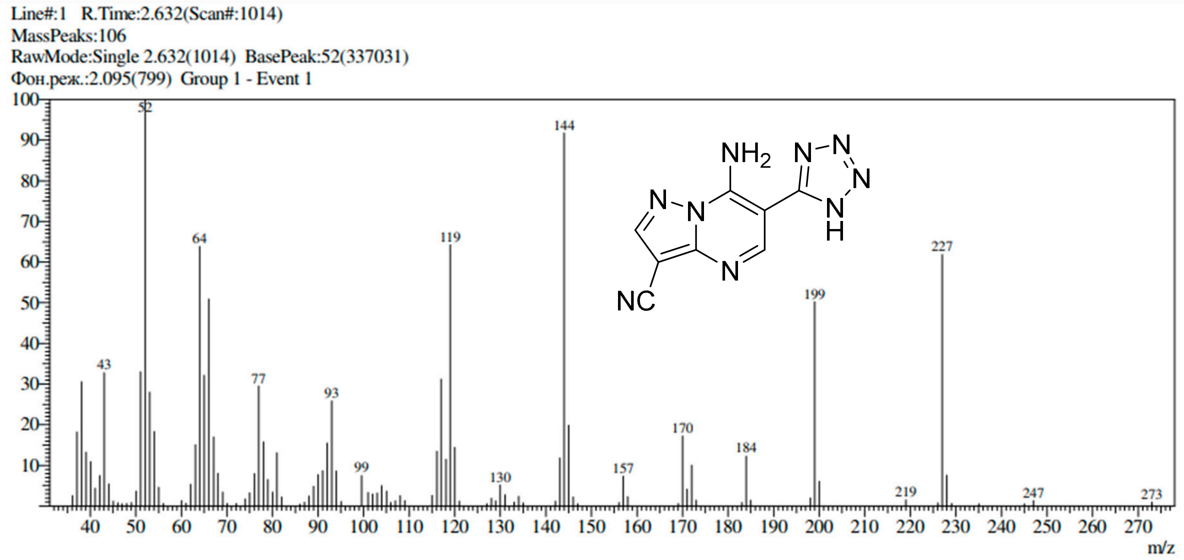

Figure S12. IR and MS (EI, 70 eV) spectra of **2f**

3-ethoxycarbonyl-6-(1H-tetrazol-5-yl)-7-aminopyrazolo[1,5-a]pyrimidine (2g)

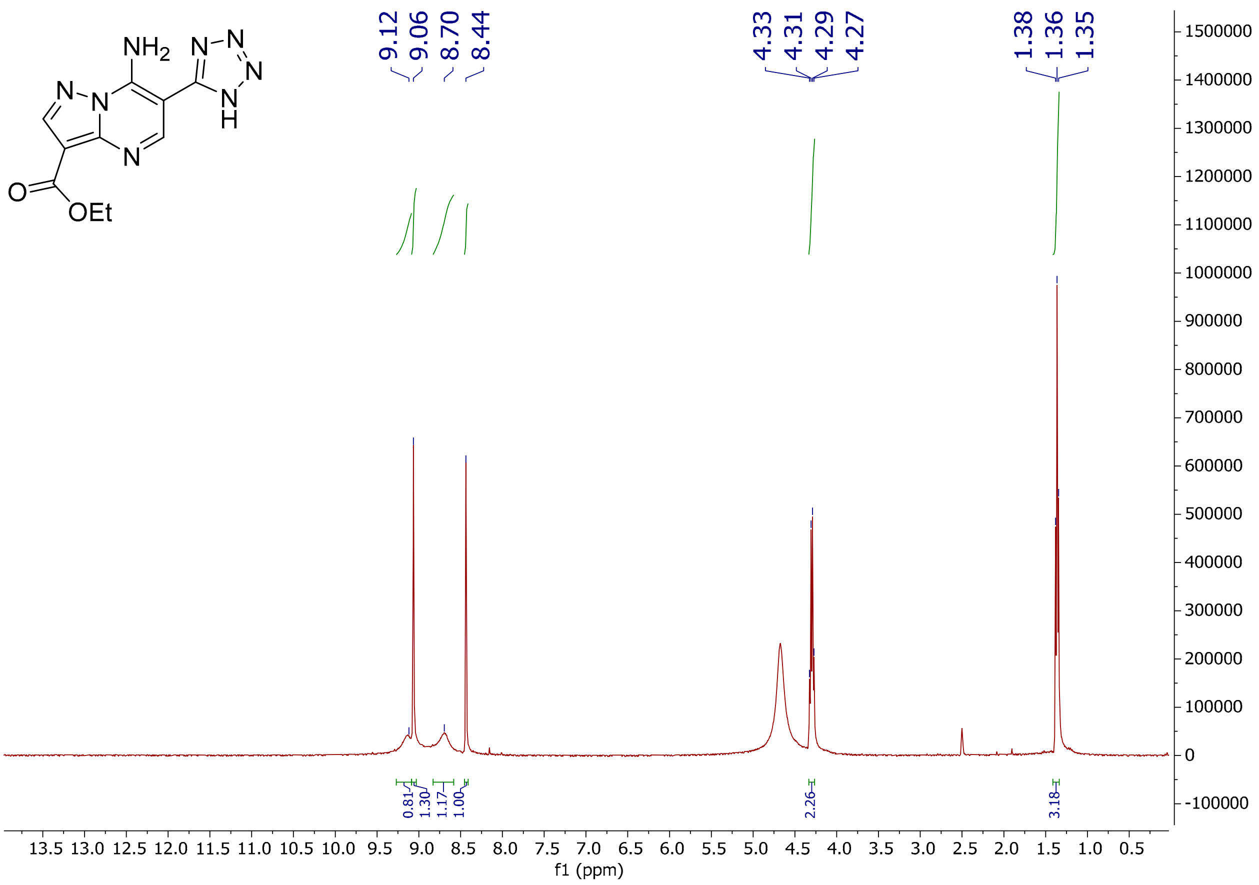

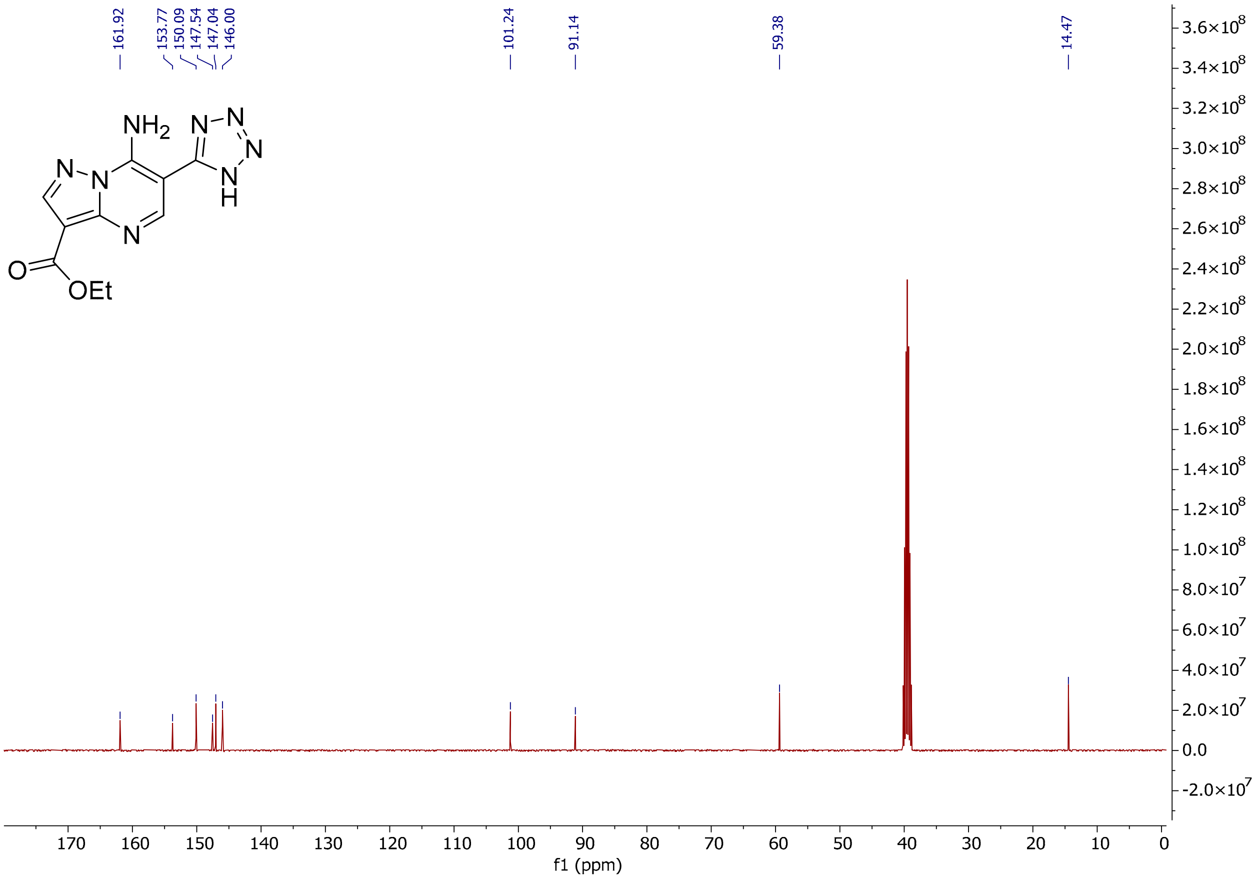

Figure S13. ^{1}H NMR (400 MHz, DMSO-d_6) and ^{13}C NMR (100 MHz, DMSO-d_6) spectra of **2g**

3-ethoxycarbonyl-6-(1H-tetrazol-5-yl)-7-aminopyrazolo[1,5-a]pyrimidine (2g)

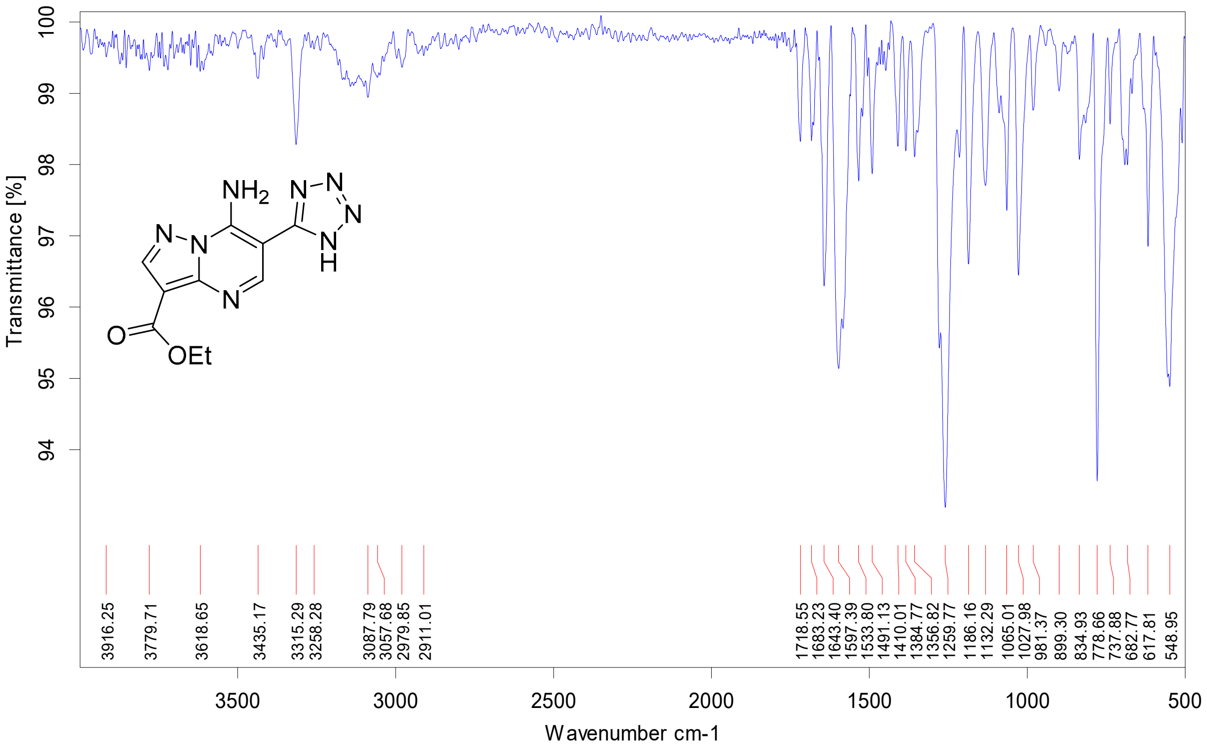

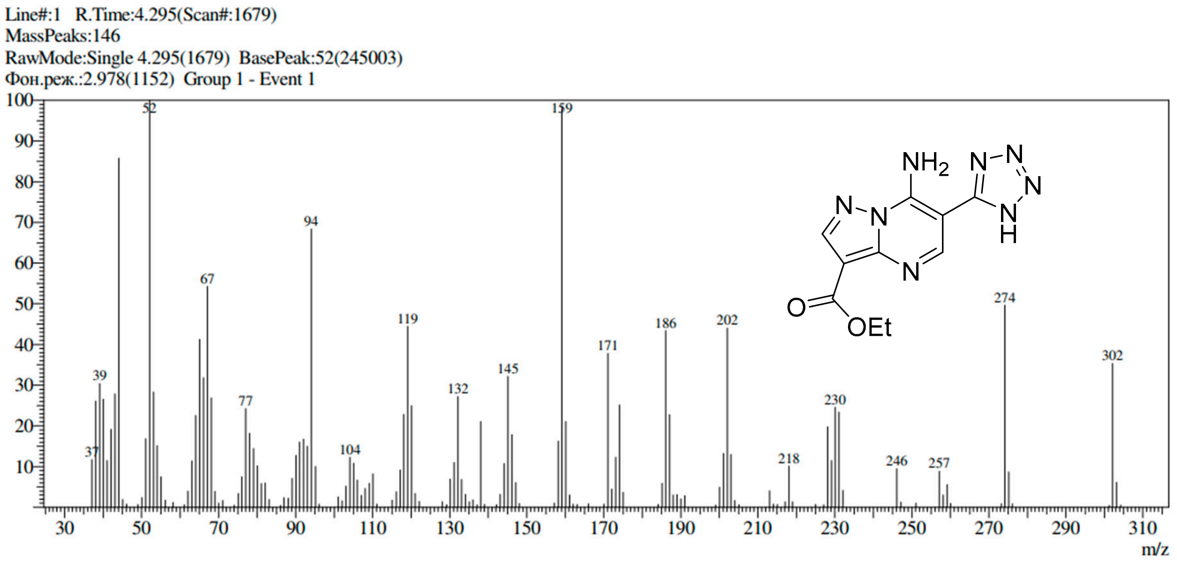

Figure S14. IR and MS (EI, 70 eV) spectra of **2g**

3-nitro-6-(1H-tetrazol-5-yl)-7-aminopyrazolo[1,5-a]pyrimidine (1H)

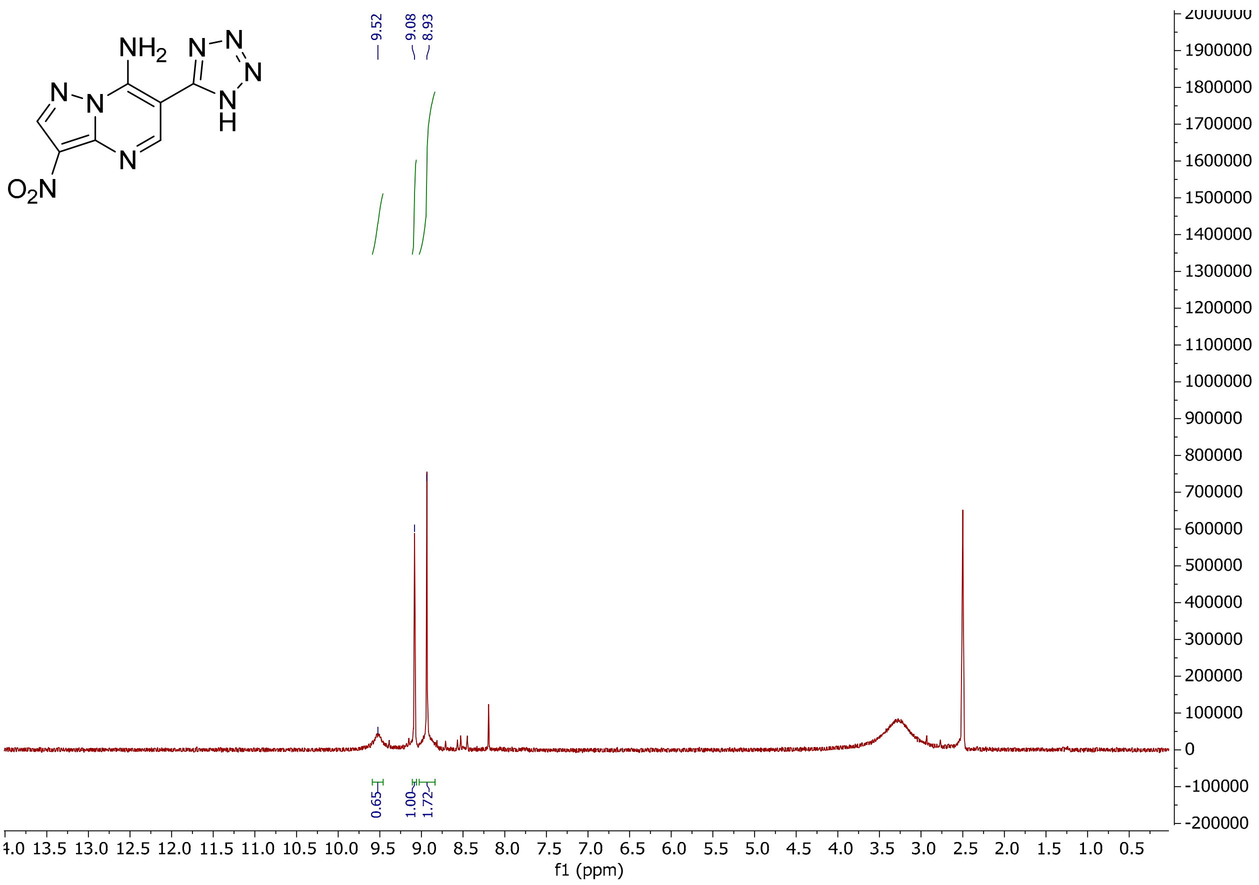

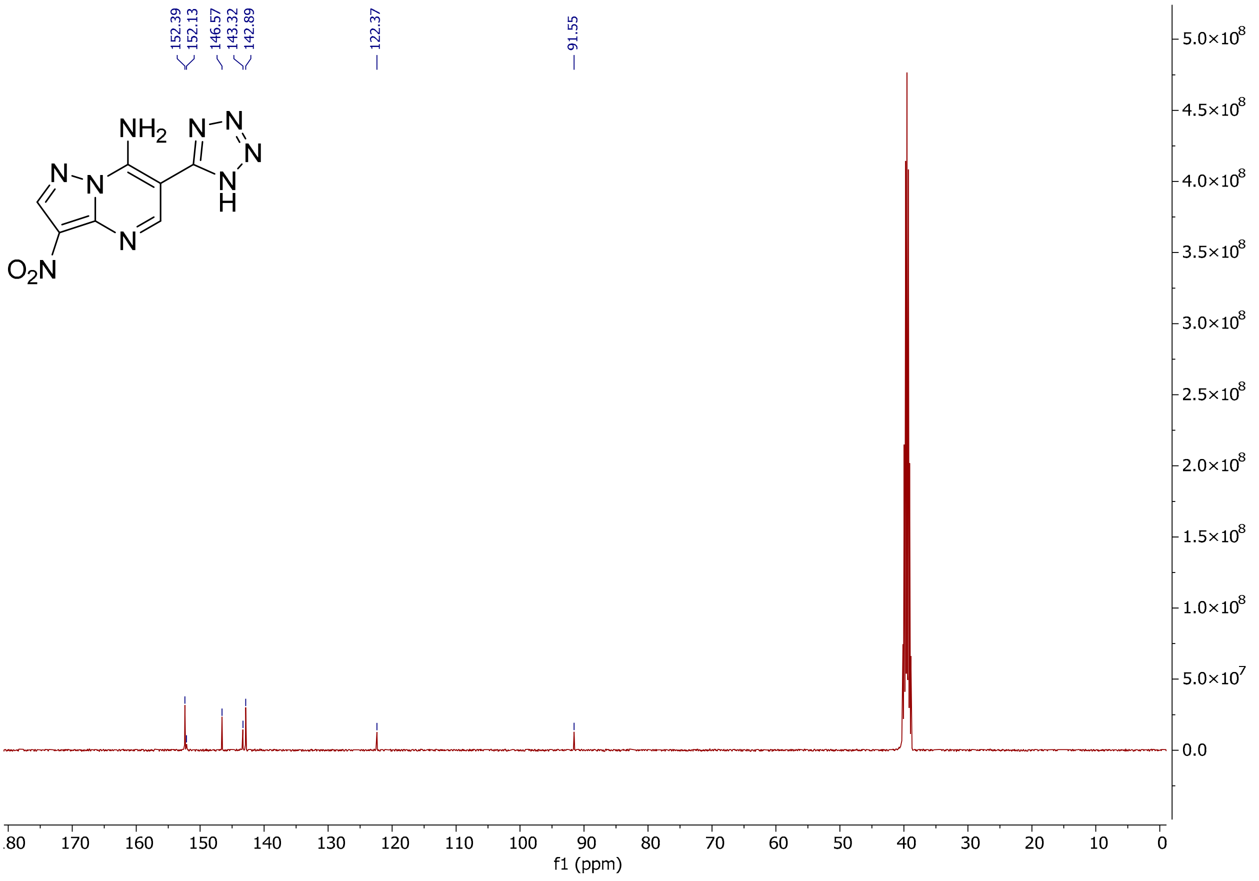

Figure S15. ^{1}H NMR (400 MHz, DMSO-d_6) and ^{13}C NMR (100 MHz, DMSO-d_6) spectra of **1H**

3-nitro-6-(1H-tetrazol-5-yl)-7-aminopyrazolo[1,5-a]pyrimidine (1H)

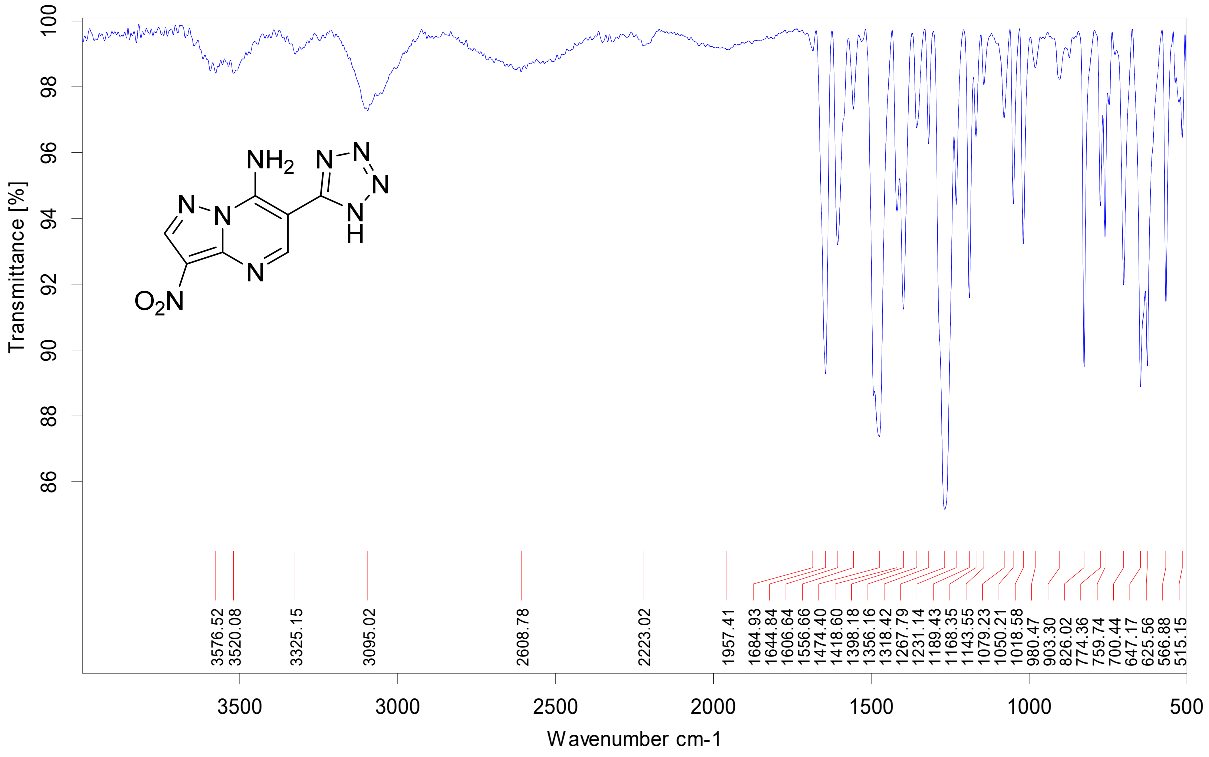

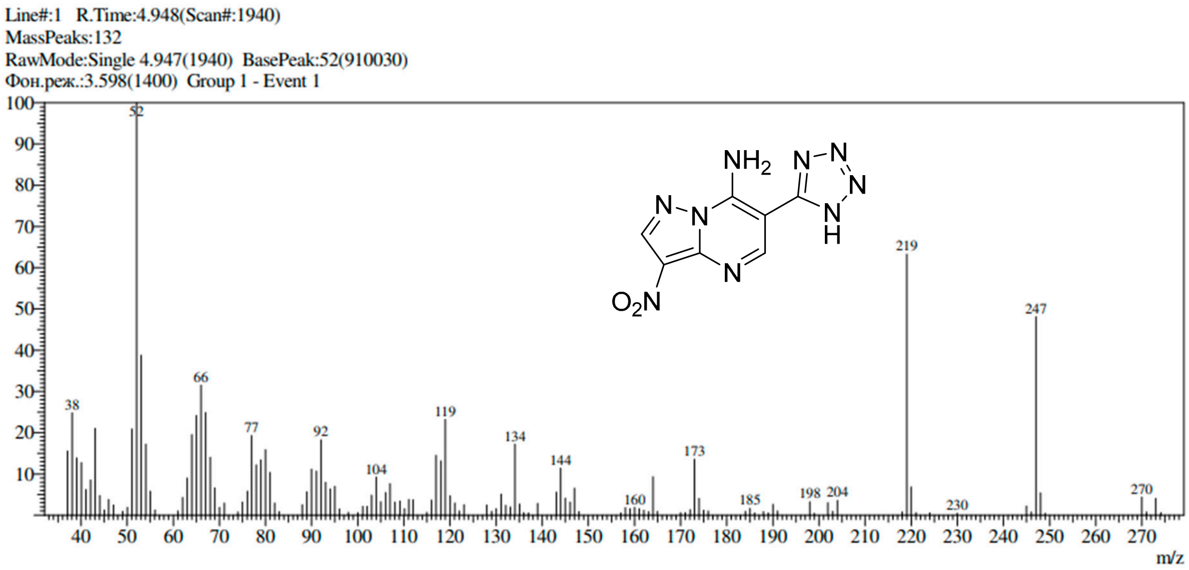

Figure S16. IR and MS (EI, 70 eV) spectra of **1H**

3-phenyl-6-(1H-tetrazol-5-yl)pyrazolo[1,5-a]pyrimidin-7-amine (2i)

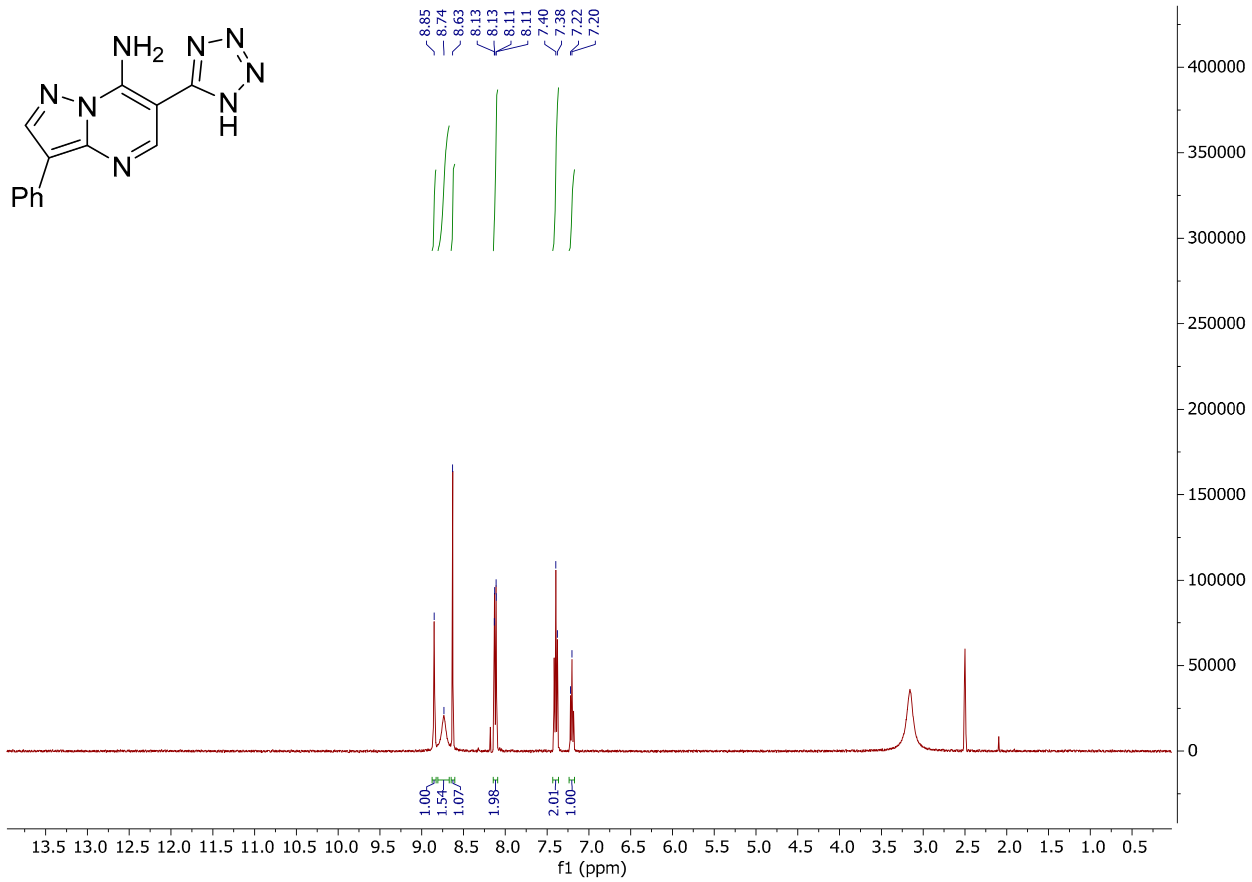

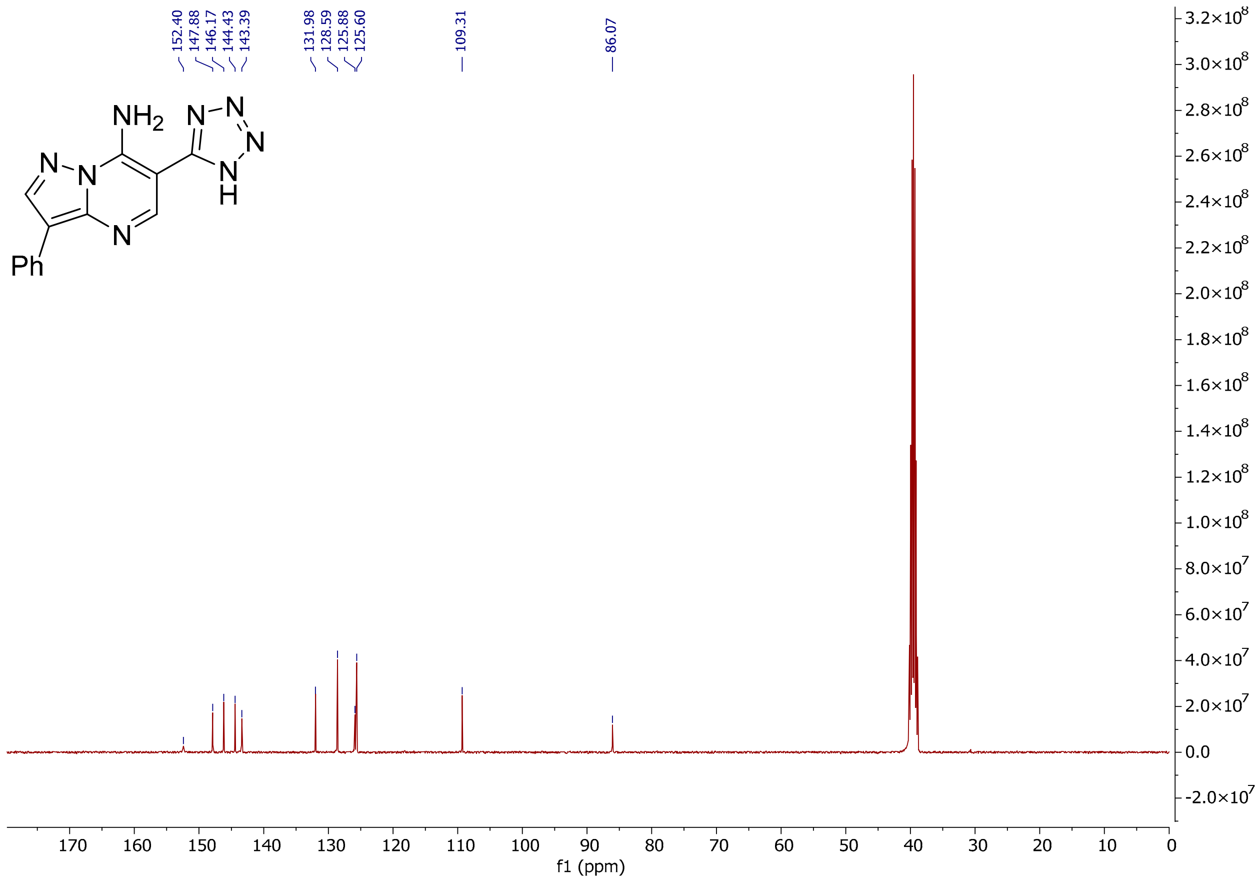

Figure S17. ^{1}H NMR (400 MHz, DMSO-d_6) and ^{13}C NMR (100 MHz, DMSO-d_6) spectra of **2i**

3-phenyl-6-(1H-tetrazol-5-yl)pyrazolo[1,5-a]pyrimidin-7-amine (2i)

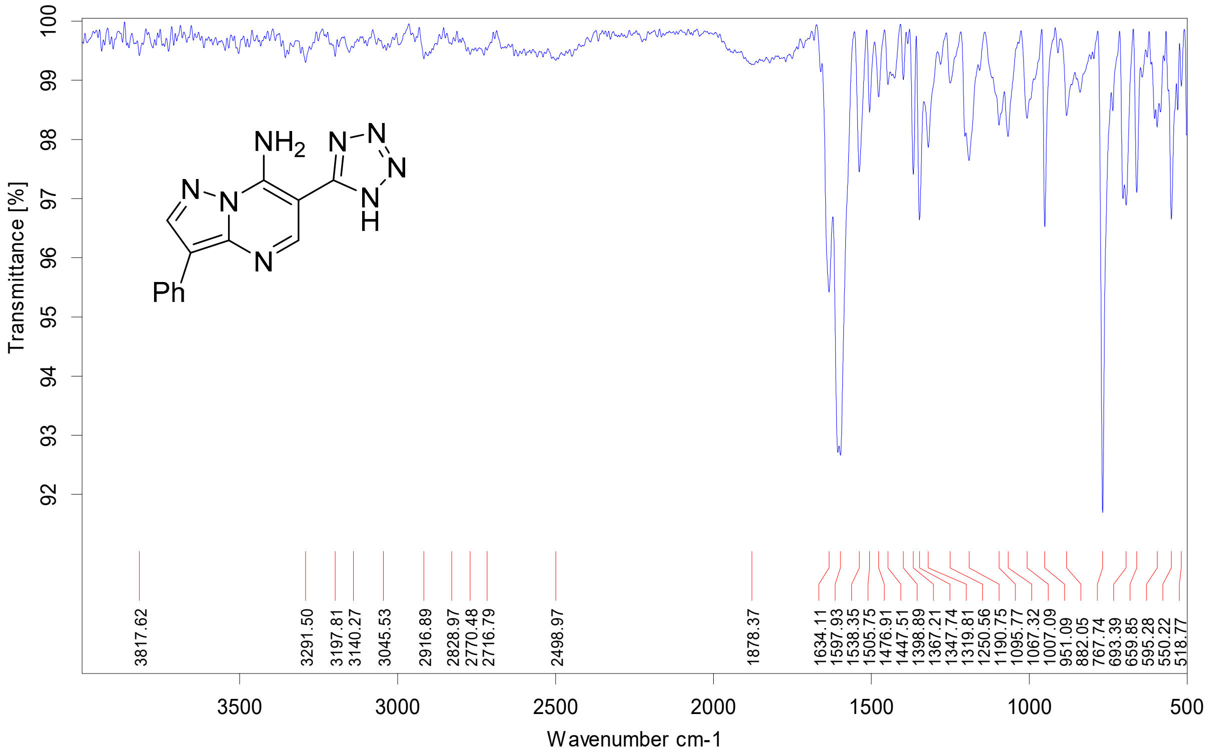

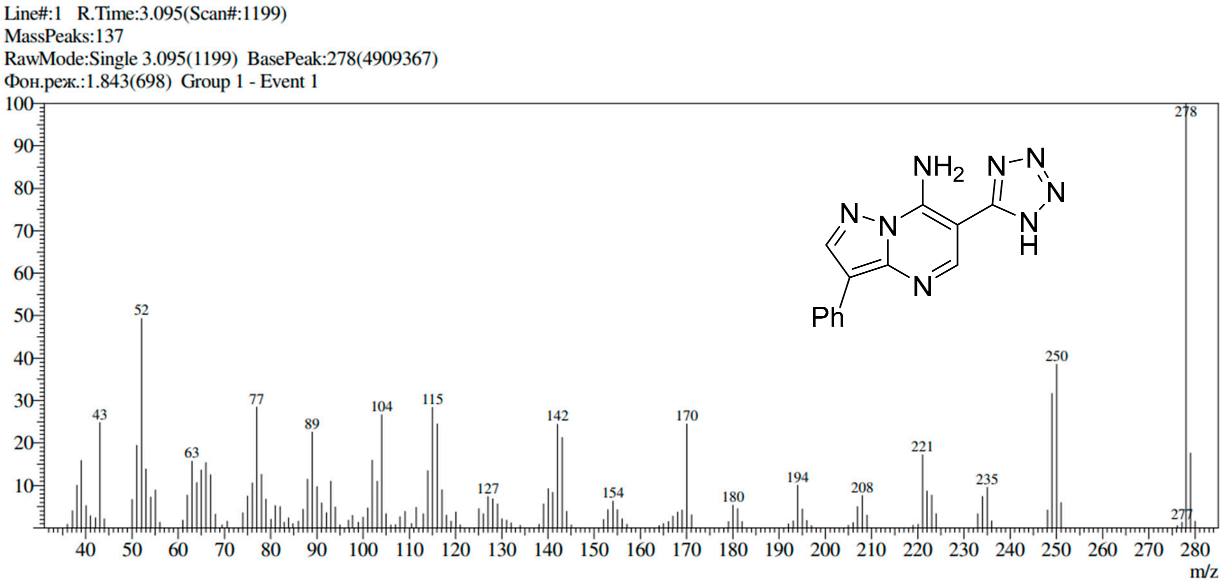

Figure S18. IR and MS (EI, 70 eV) spectra of **2i**

2-(methylthio)-3-ethoxycarbonly-6-(1H-tetrazol-5-yl)-7-aminopyrazolo[1,5-a]pyrimidine (2j)

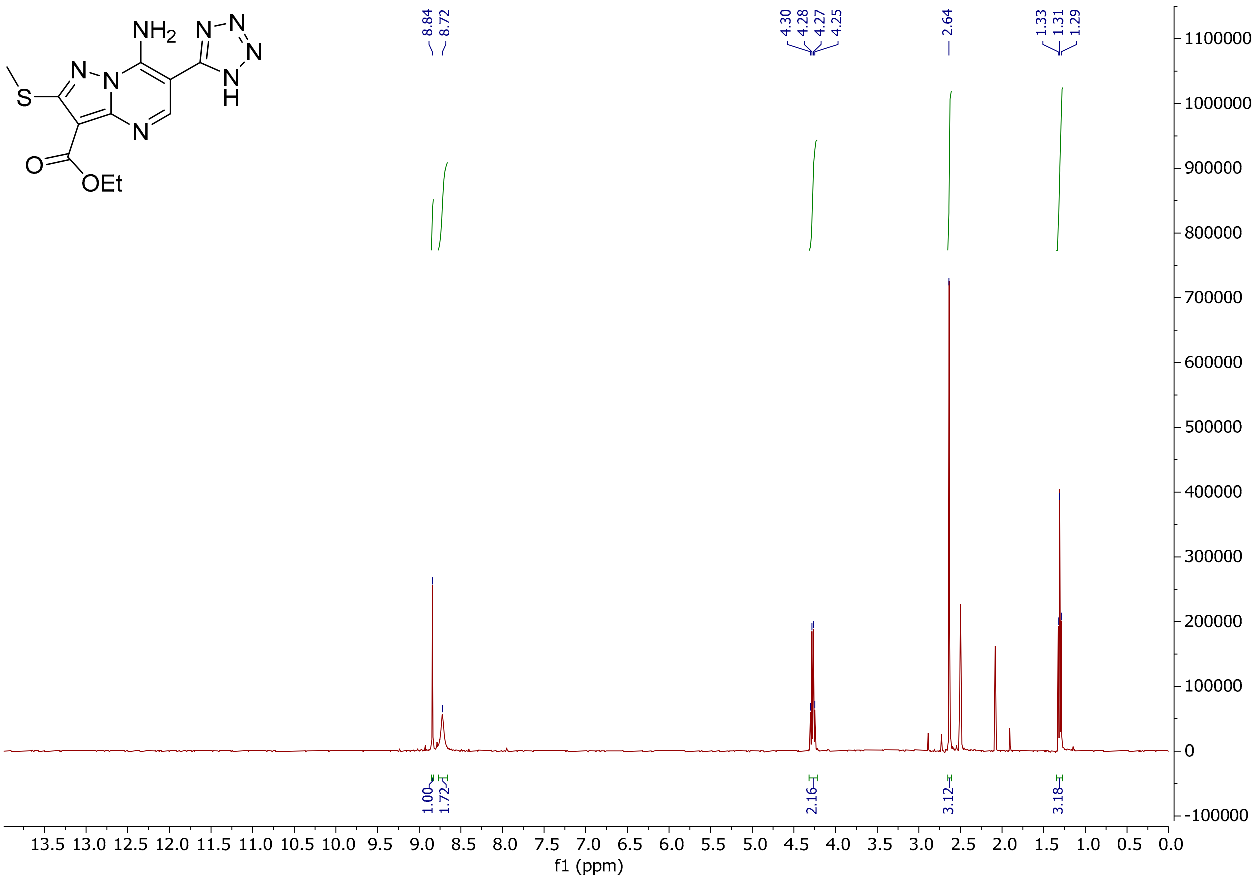

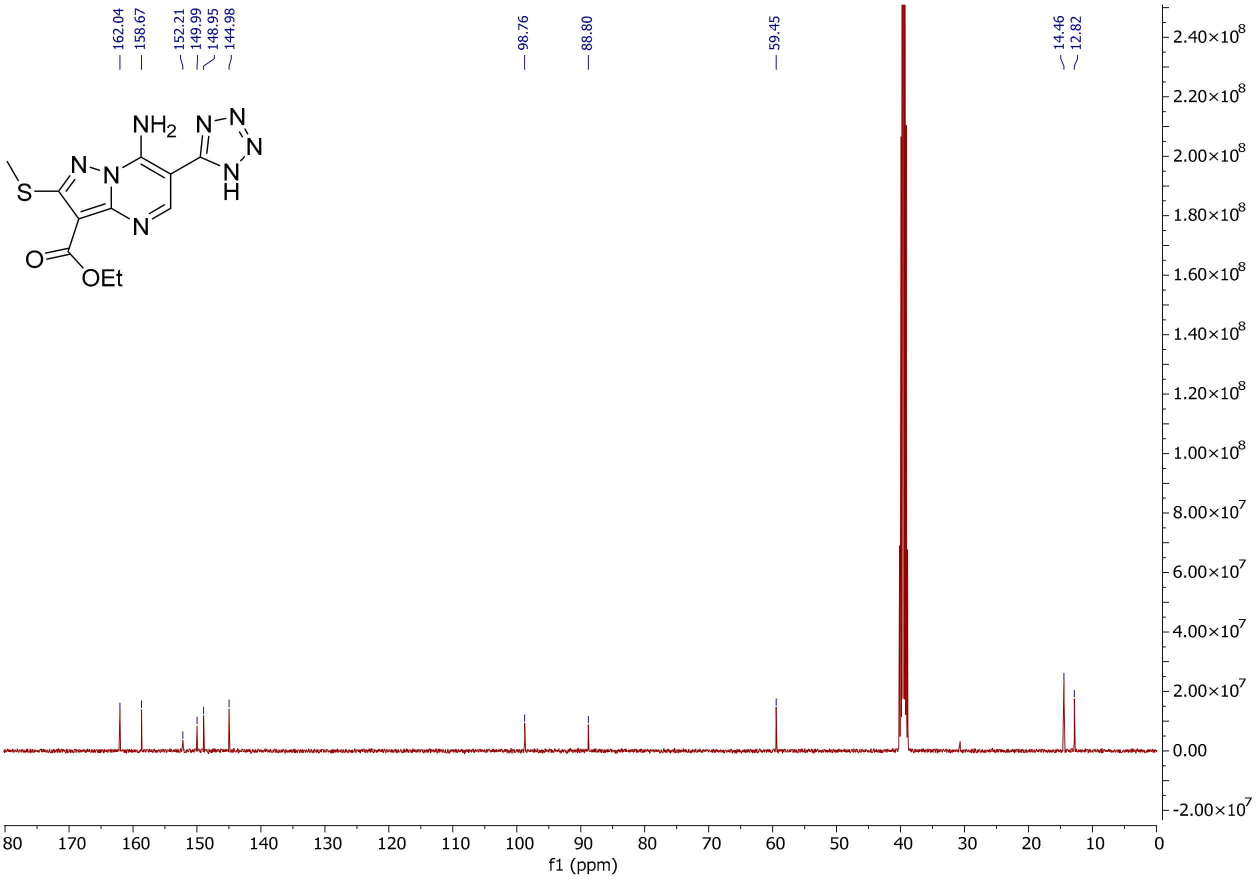

Figure S19. ^{1}H NMR (400 MHz, DMSO-d_6) and ^{13}C NMR (100 MHz, DMSO-d_6) spectra of **2j**

2-(methylthio)-3-ethoxycarbonly-6-(1H-tetrazol-5-yl)-7-aminopyrazolo[1,5-a]pyrimidine (2j)

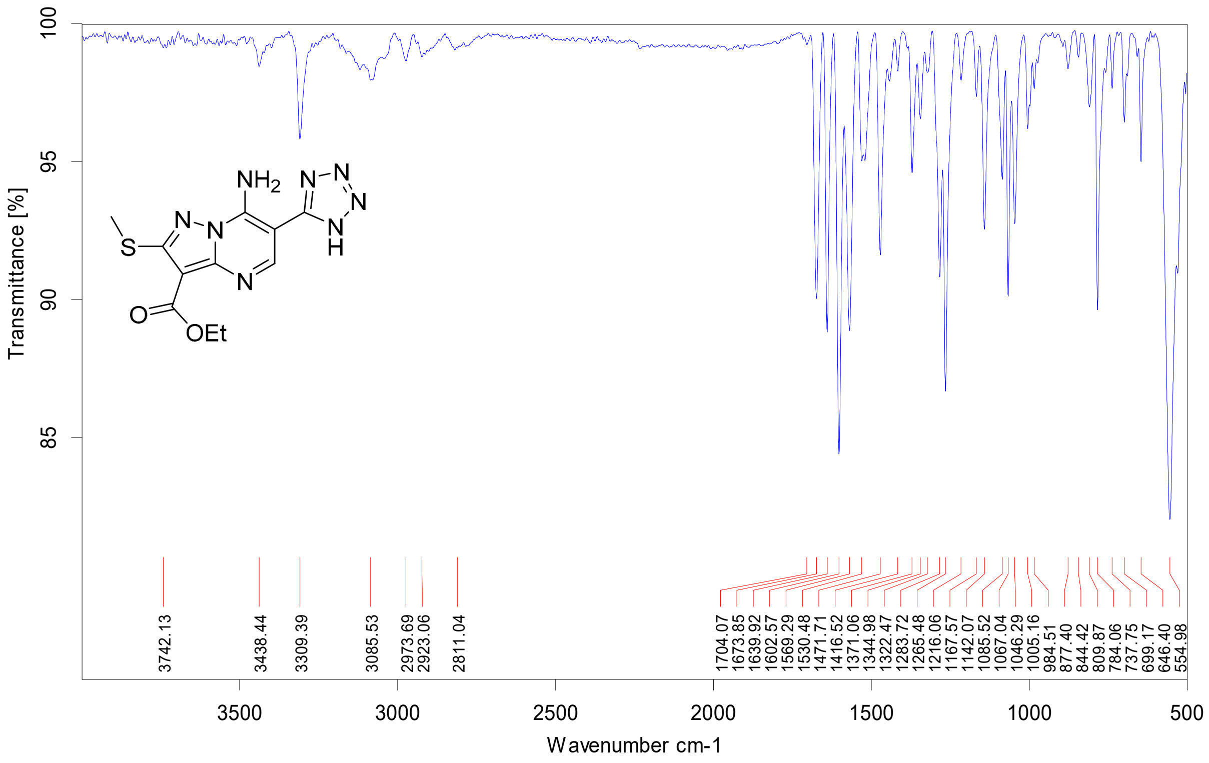

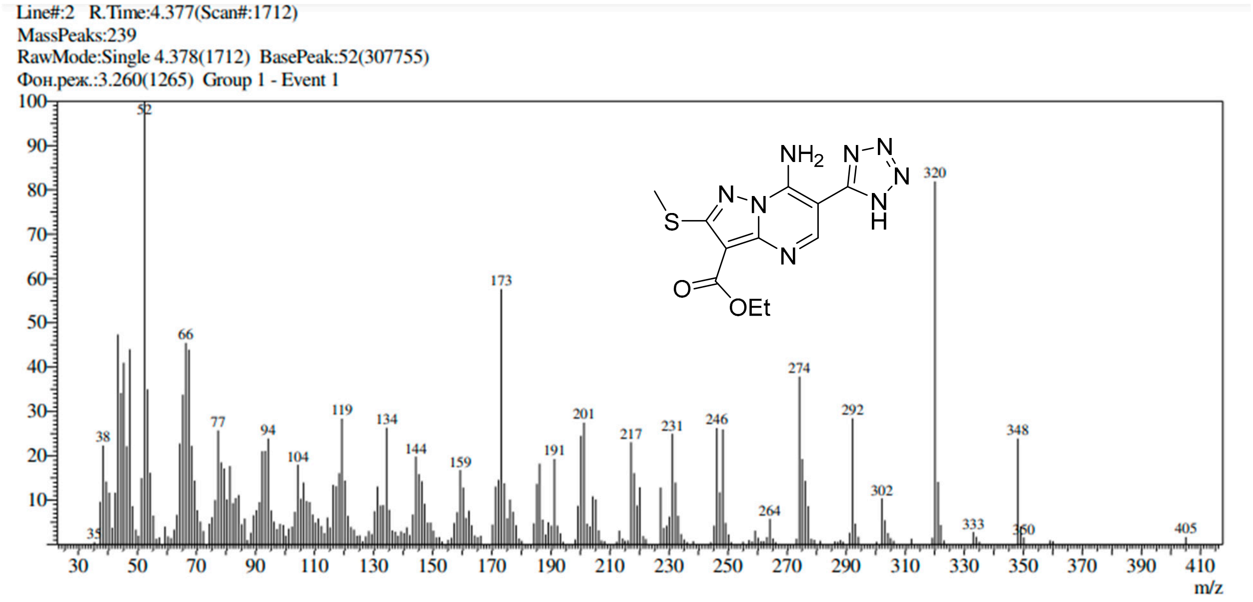

Figure S20. IR and MS (EI, 70 eV) spectra of **2j**

2-(methylthio)-3-carbonitrile-6-(1H-tetrazol-5-yl)-7-aminopyrazolo[1,5-a]pyrimidine (2k)

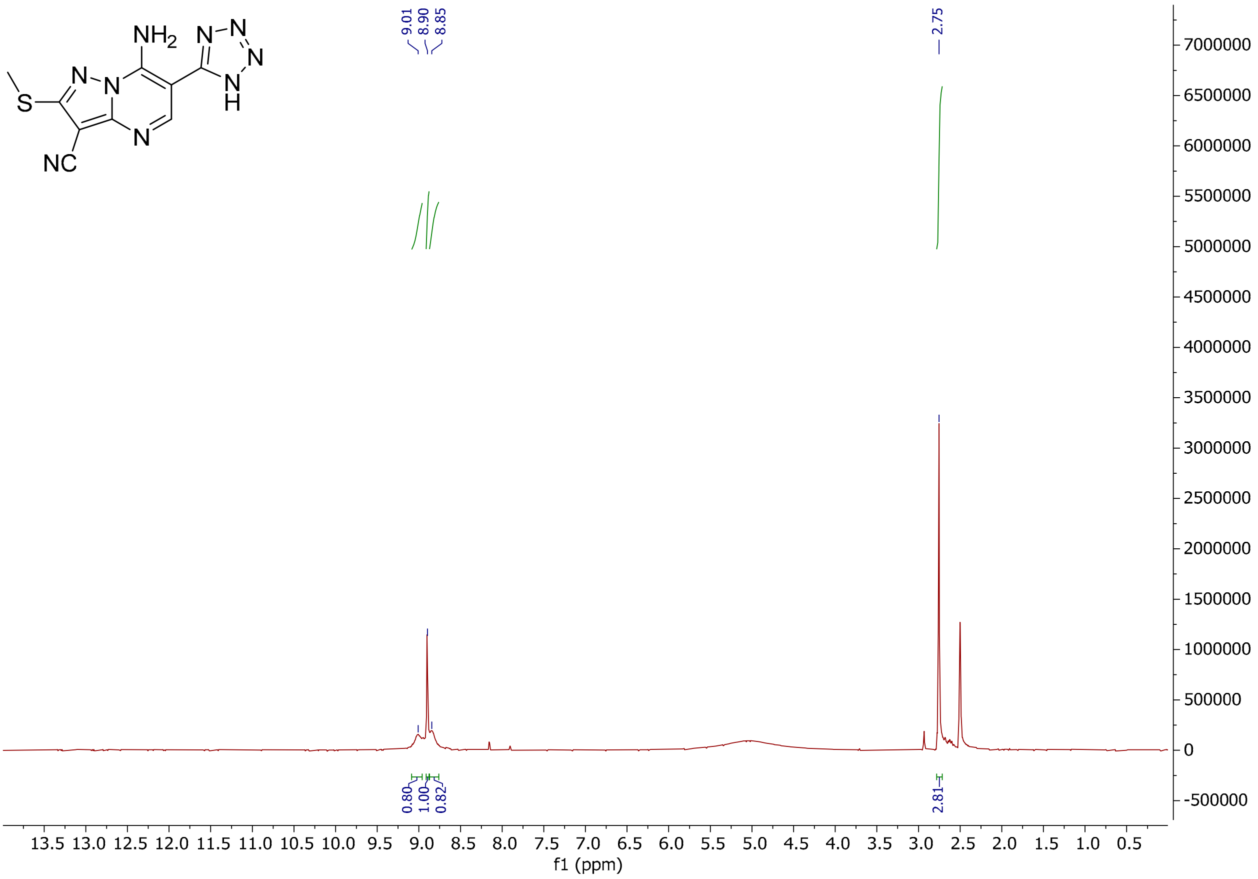

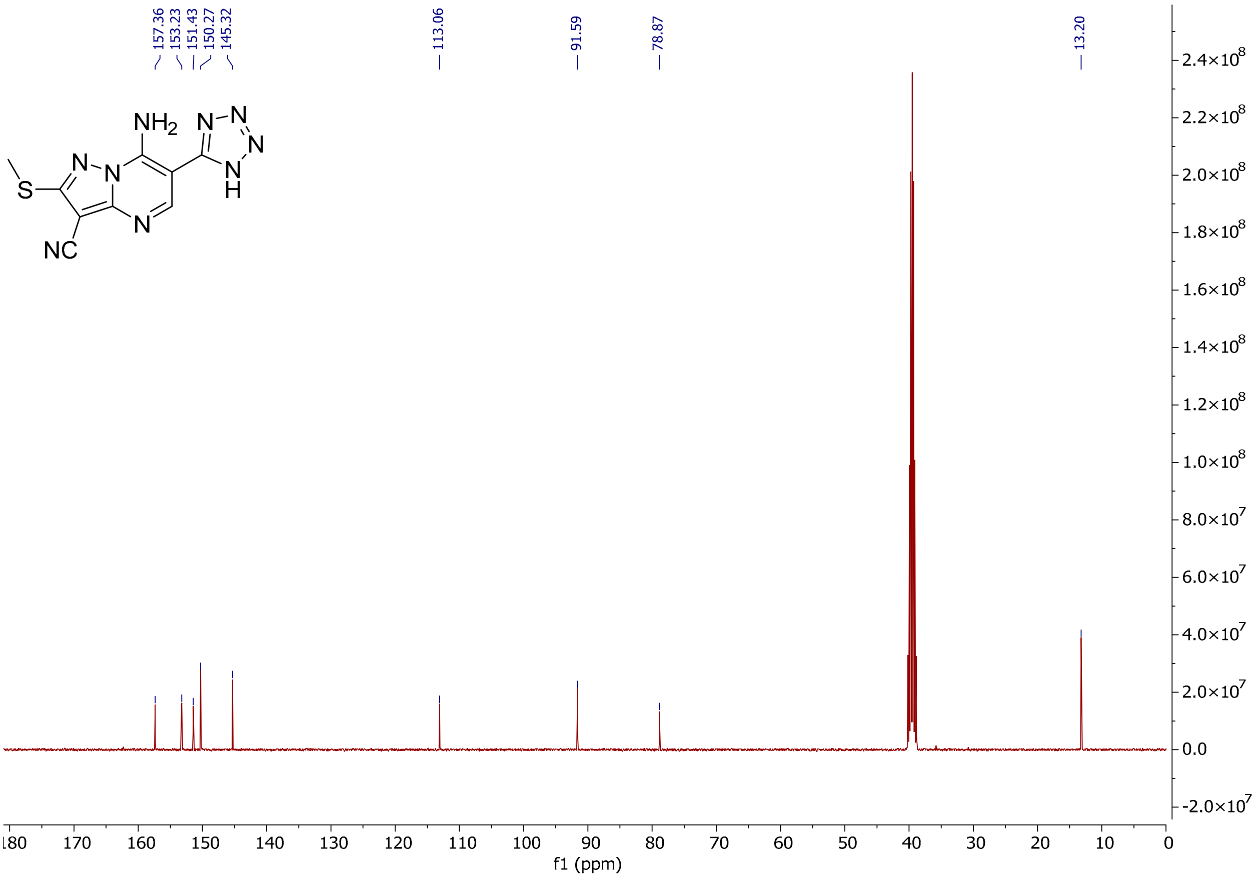

Figure S21. ^{1}H NMR (400 MHz, DMSO-d_6) and ^{13}C NMR (100 MHz, DMSO-d_6) spectra of **2k**

2-(methylthio)-3-carbonitrile-6-(1H-tetrazol-5-yl)-7-aminopyrazolo[1,5-a]pyrimidine (2k)

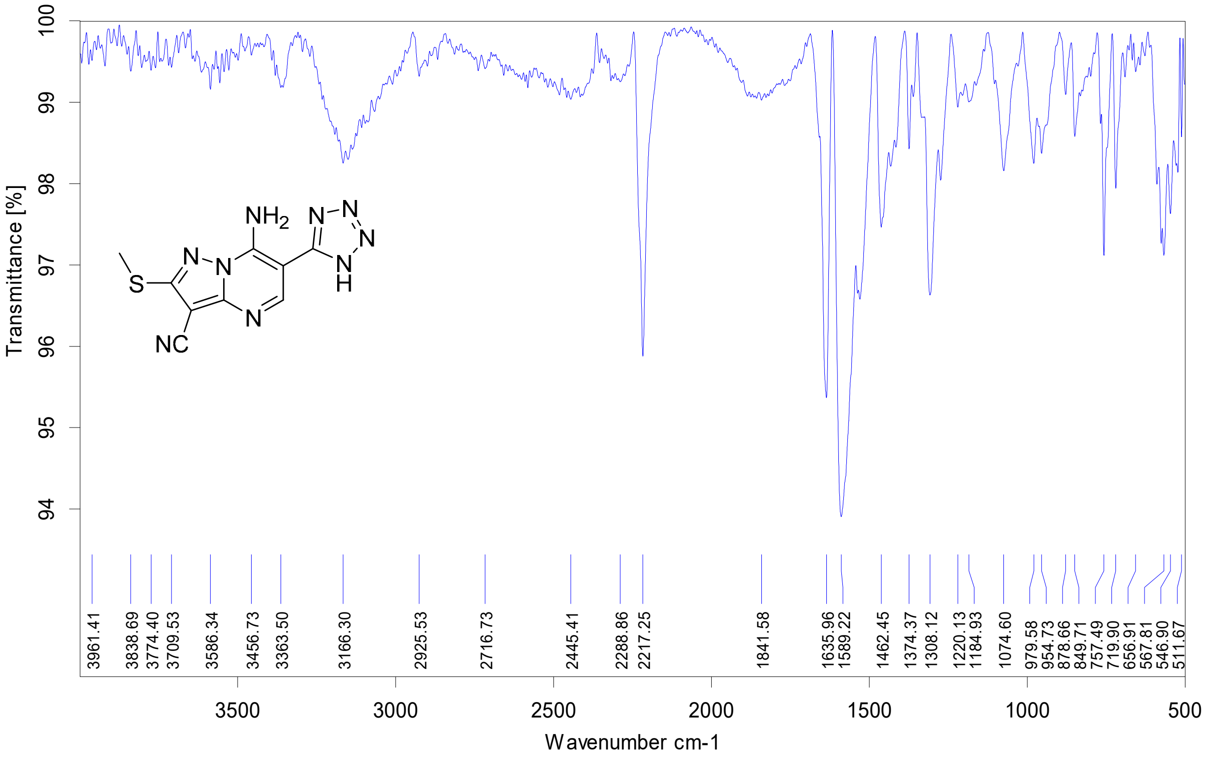

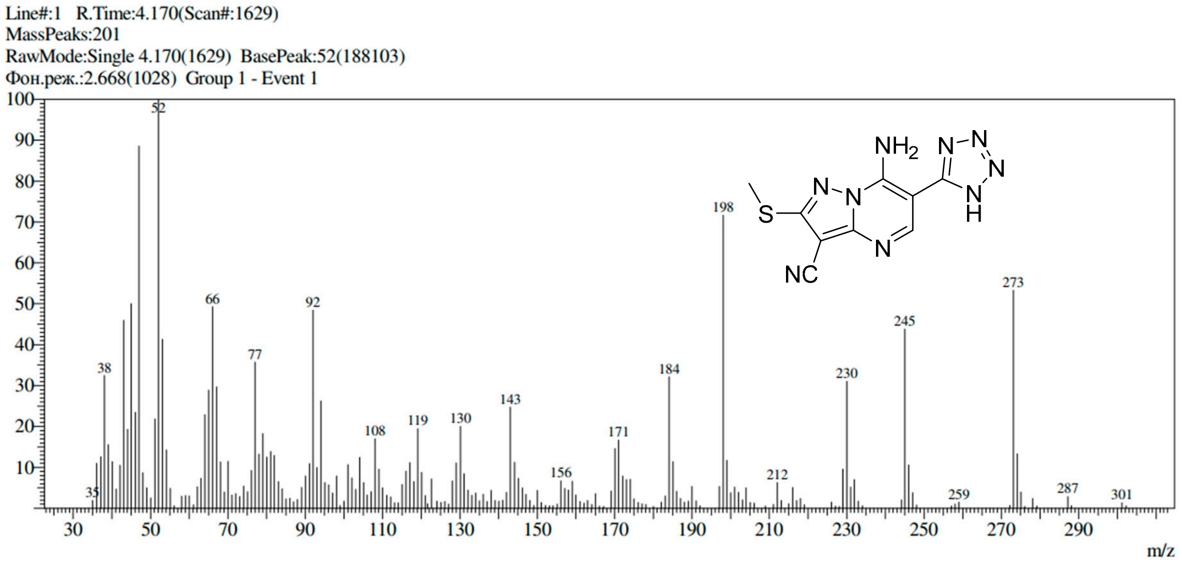

Figure S22. IR and MS (EI, 70 eV) spectra of **2k**

6-(1H-tetrazol-5-yl)-7-amino-[1,2,4]triazolo[1,5-a]pyrimidine (10a)

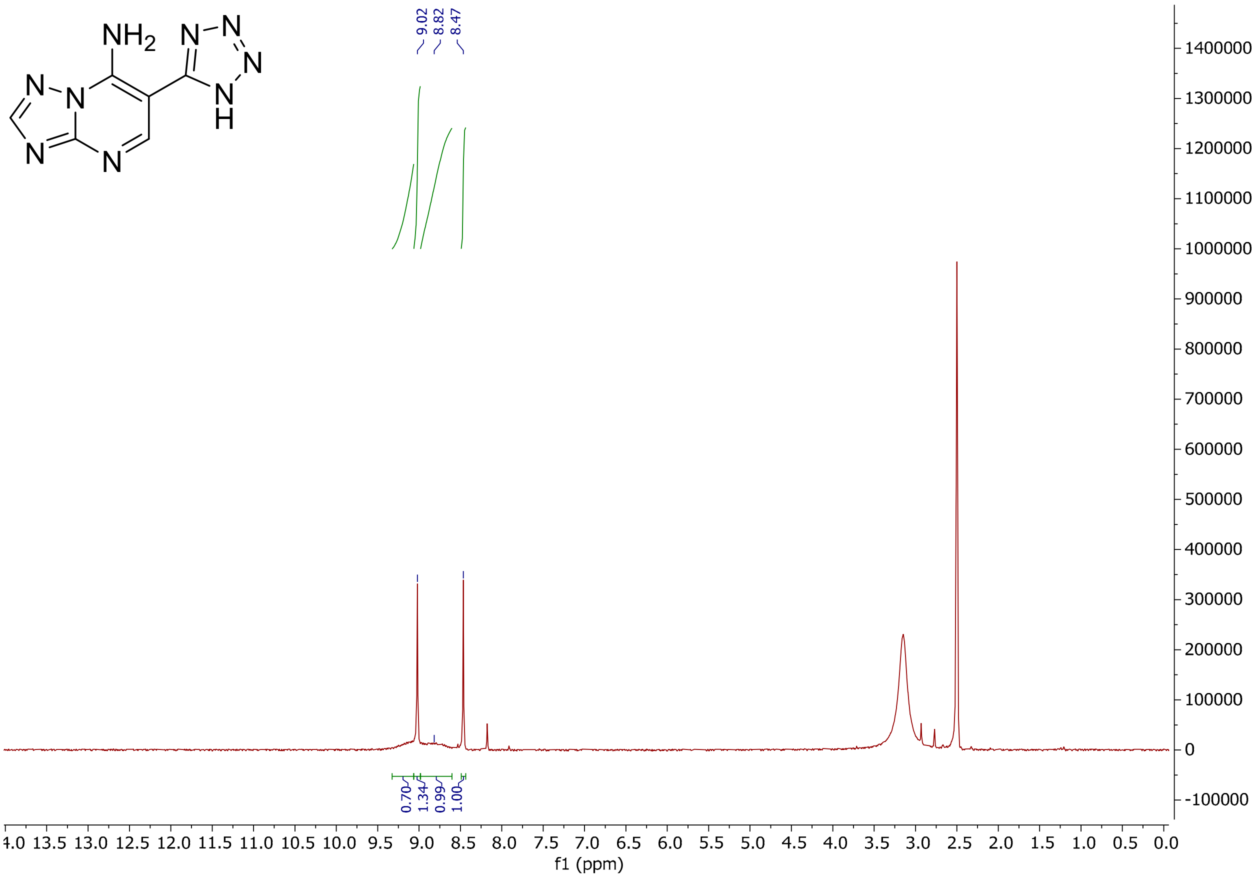

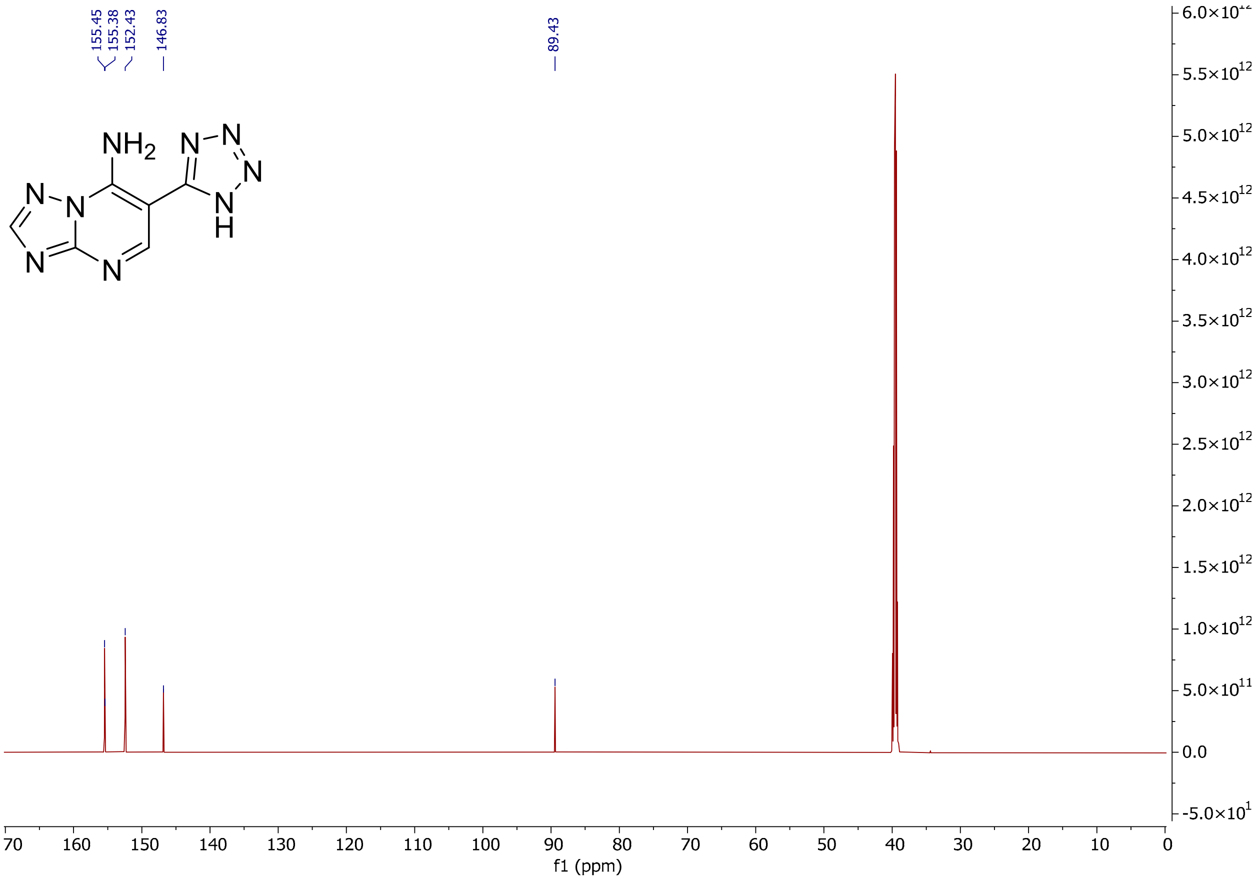

Figure S23. ^{1}H NMR (400 MHz, DMSO-d_6) and ^{13}C NMR (100 MHz, DMSO-d_6) spectra of **10a**

6-(1H-tetrazol-5-yl)-7-amino-[1,2,4]triazolo[1,5-a]pyrimidine (10a)

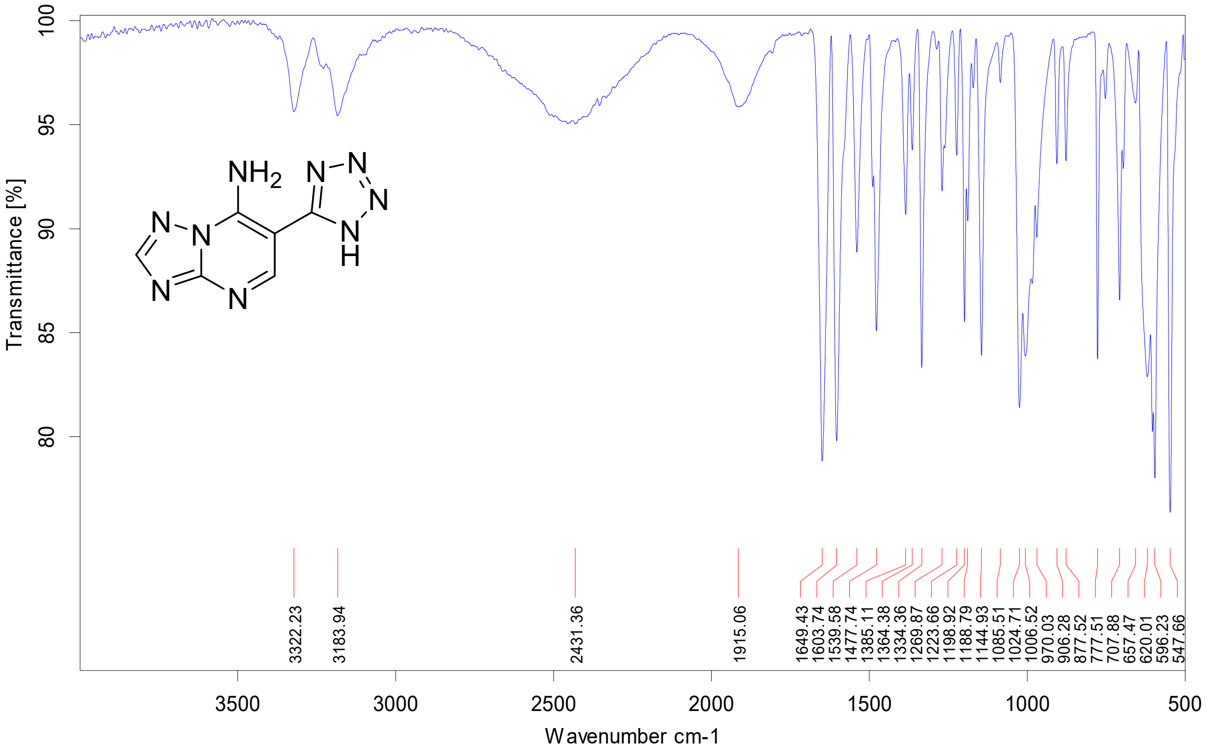

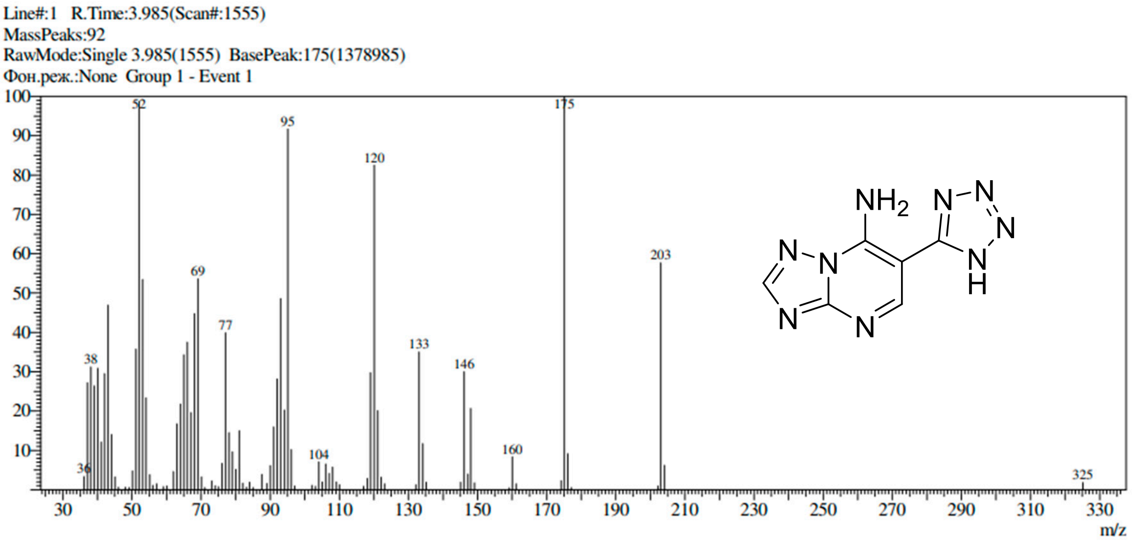

Figure S24. IR and MS (EI, 70 eV) spectra of **10a**

2-methyl-6-(1H-tetrazol-5-yl)-7-amino-[1,2,4]triazolo[1,5-a]pyrimidine (10b)

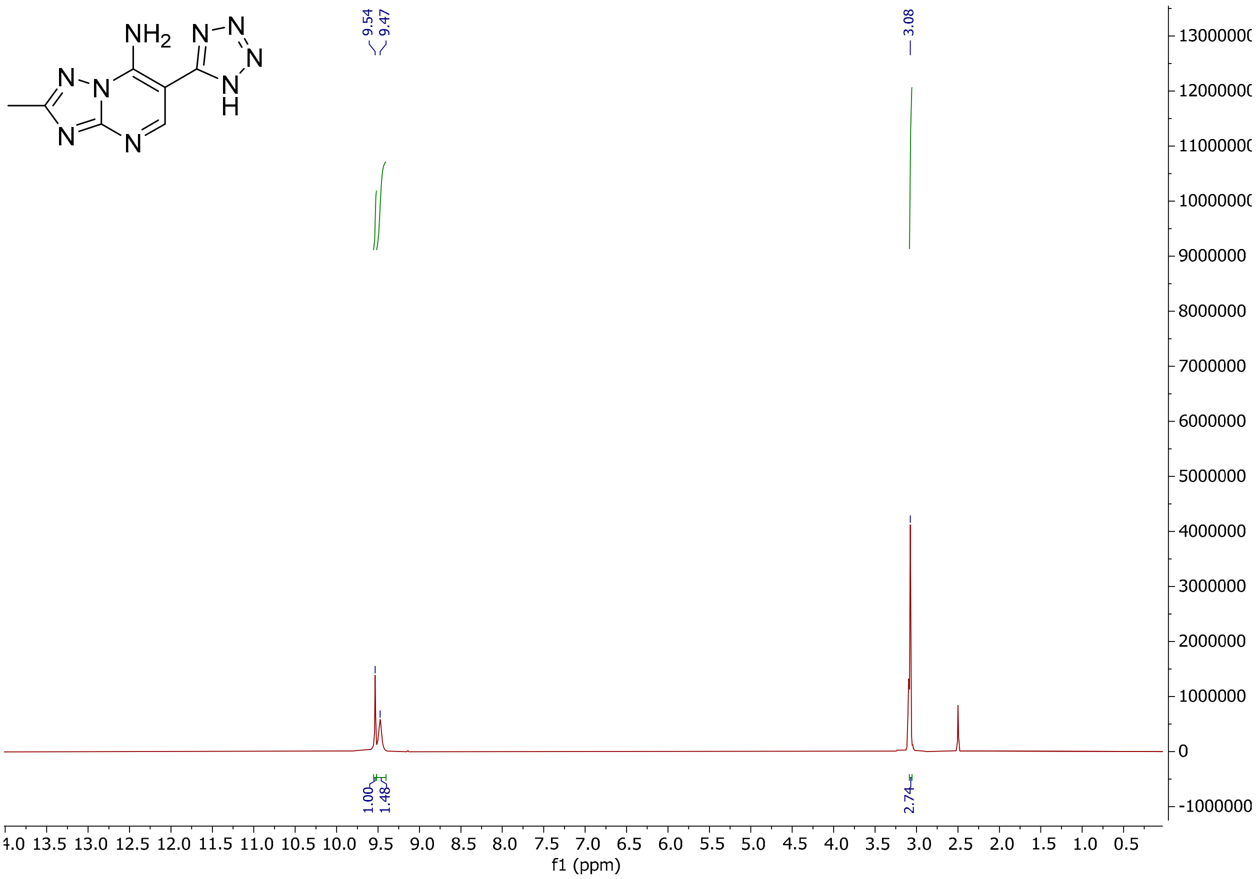

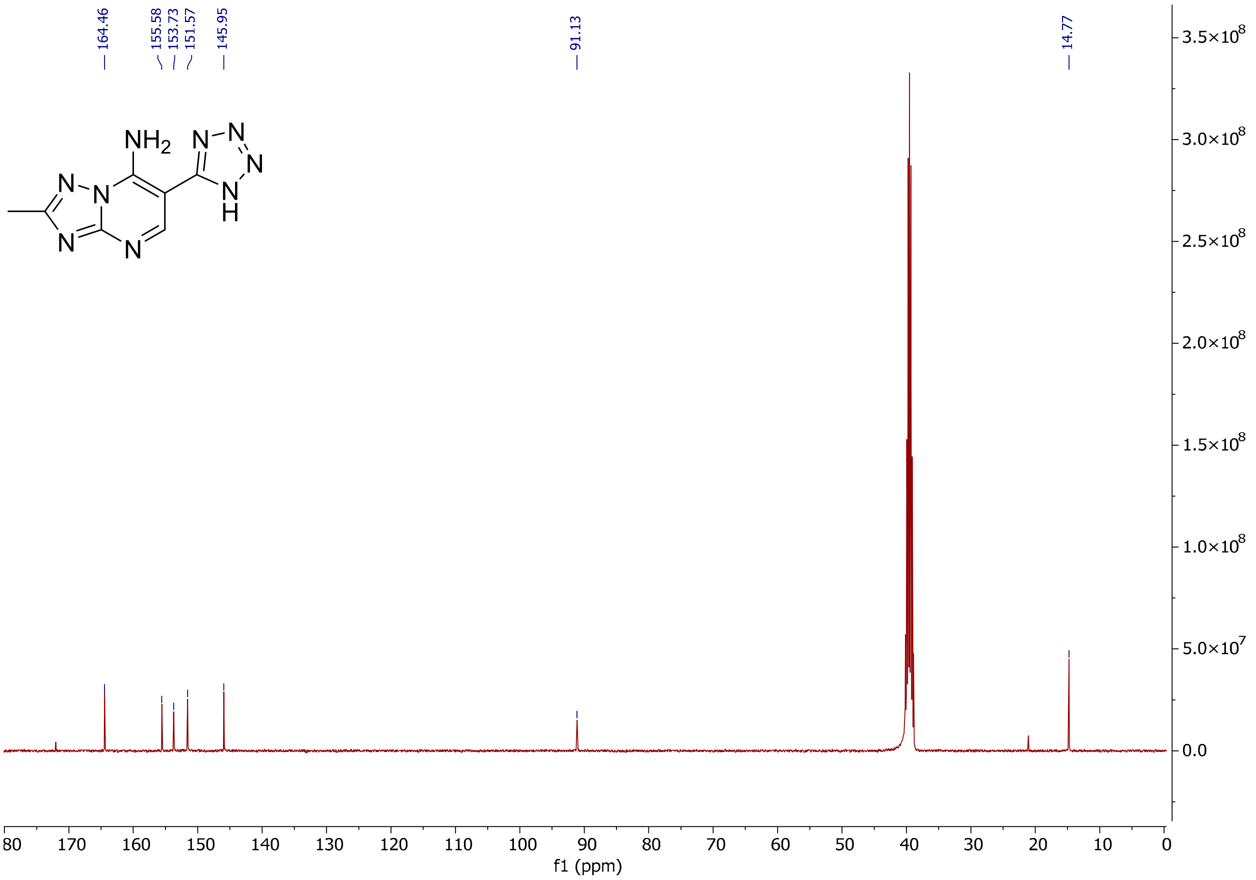

Figure S25. ^{1}H NMR (400 MHz, DMSO-d_6) and ^{13}C NMR (100 MHz, DMSO-d_6) spectra of **10b**

2-methyl-6-(1H-tetrazol-5-yl)-7-amino-[1,2,4]triazolo[1,5-a]pyrimidine (10b)

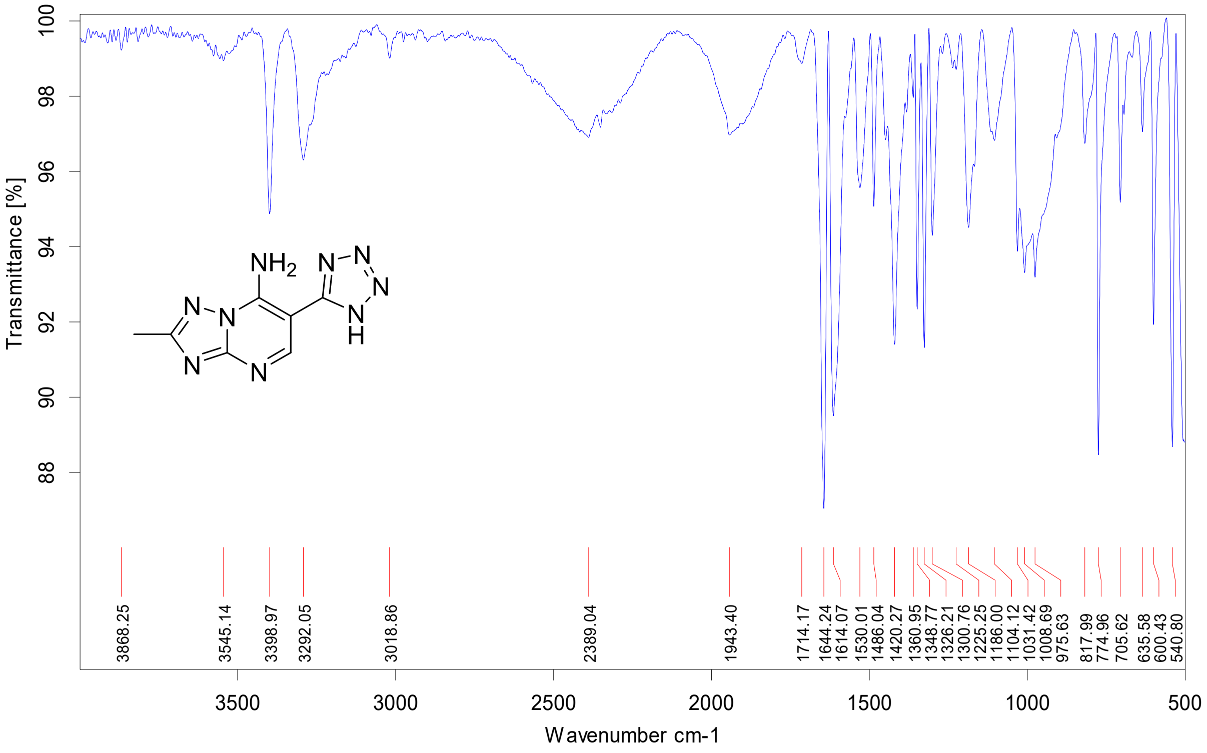

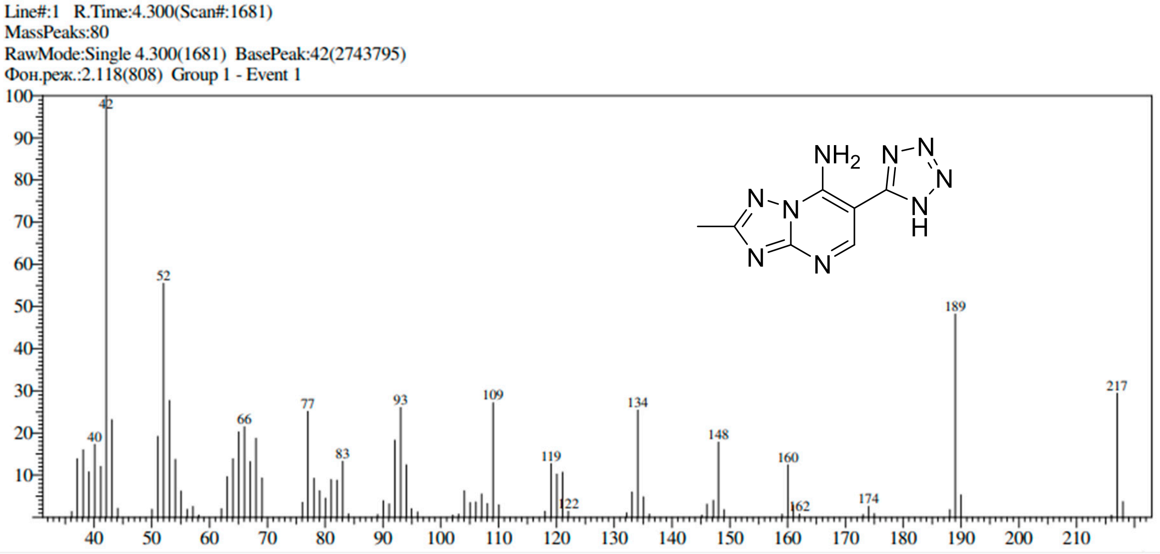

Figure S26. IR and MS (EI, 70 eV) spectra of **10b**

2-(methylthio)-6-(1H-tetrazol-5-yl)-7-amino-[1,2,4]triazolo[1,5-a]pyrimidine (10c)

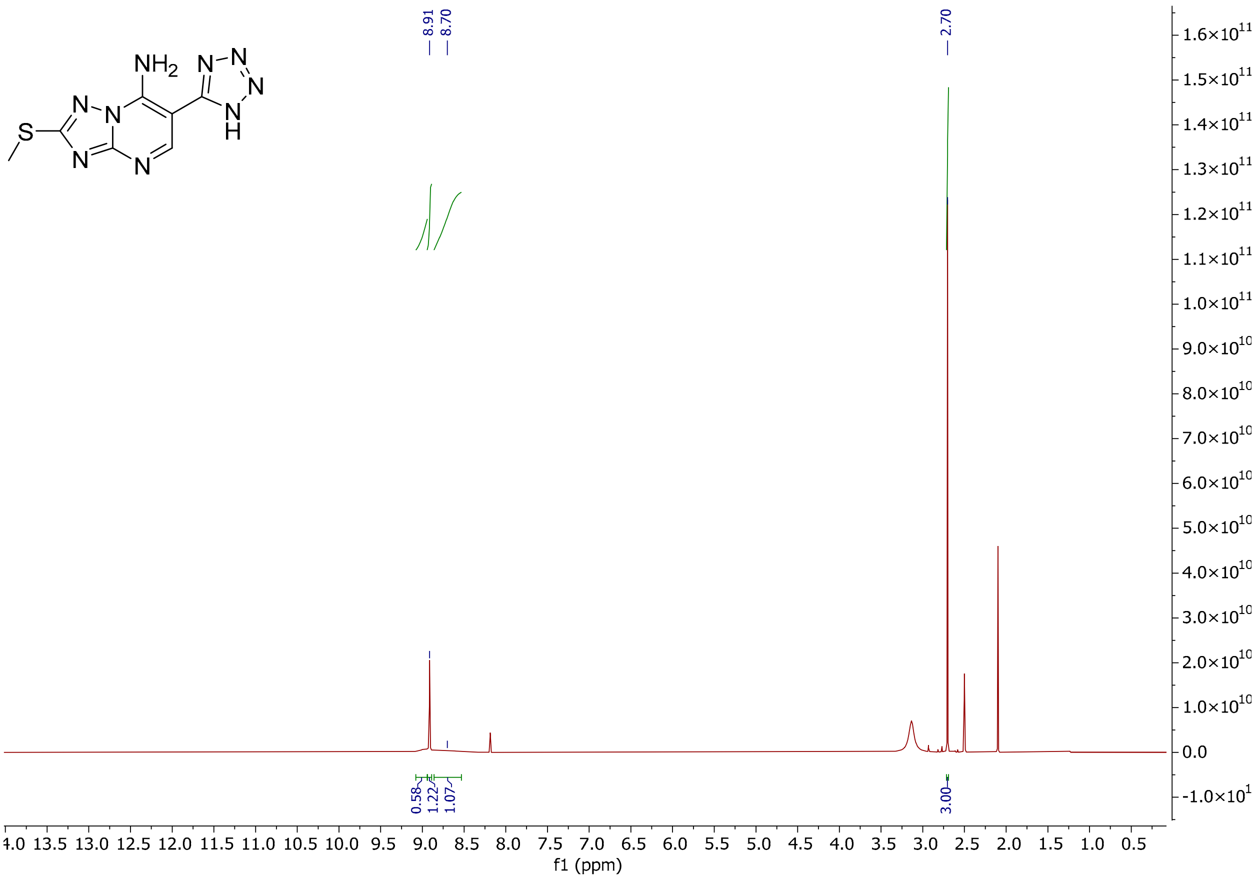

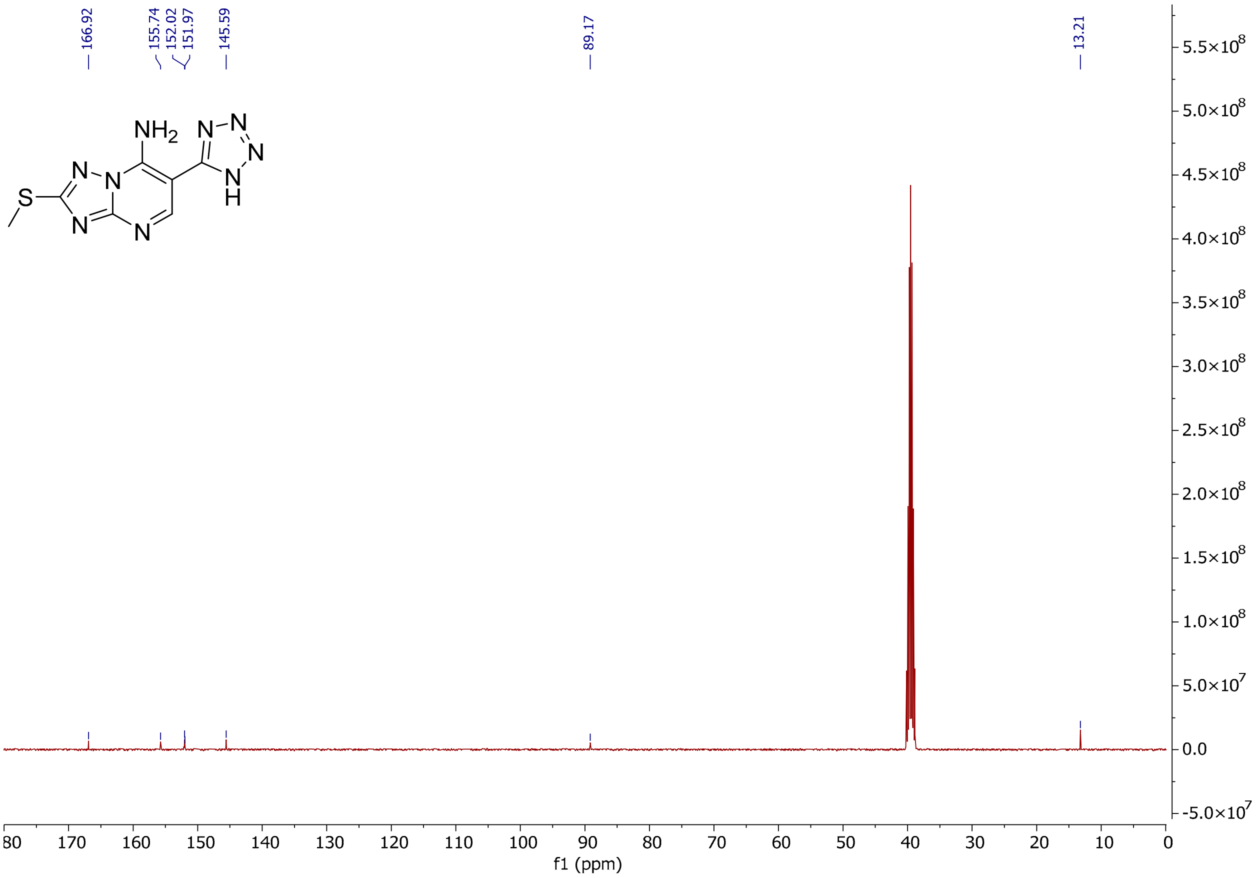

Figure S27. ^{1}H NMR (600 MHz, DMSO-d_6) and ^{13}C NMR (100 MHz, DMSO-d_6) spectra of **10c**

2-(methylthio)-6-(1H-tetrazol-5-yl)-7-amino-[1,2,4]triazolo[1,5-a]pyrimidine (10c)

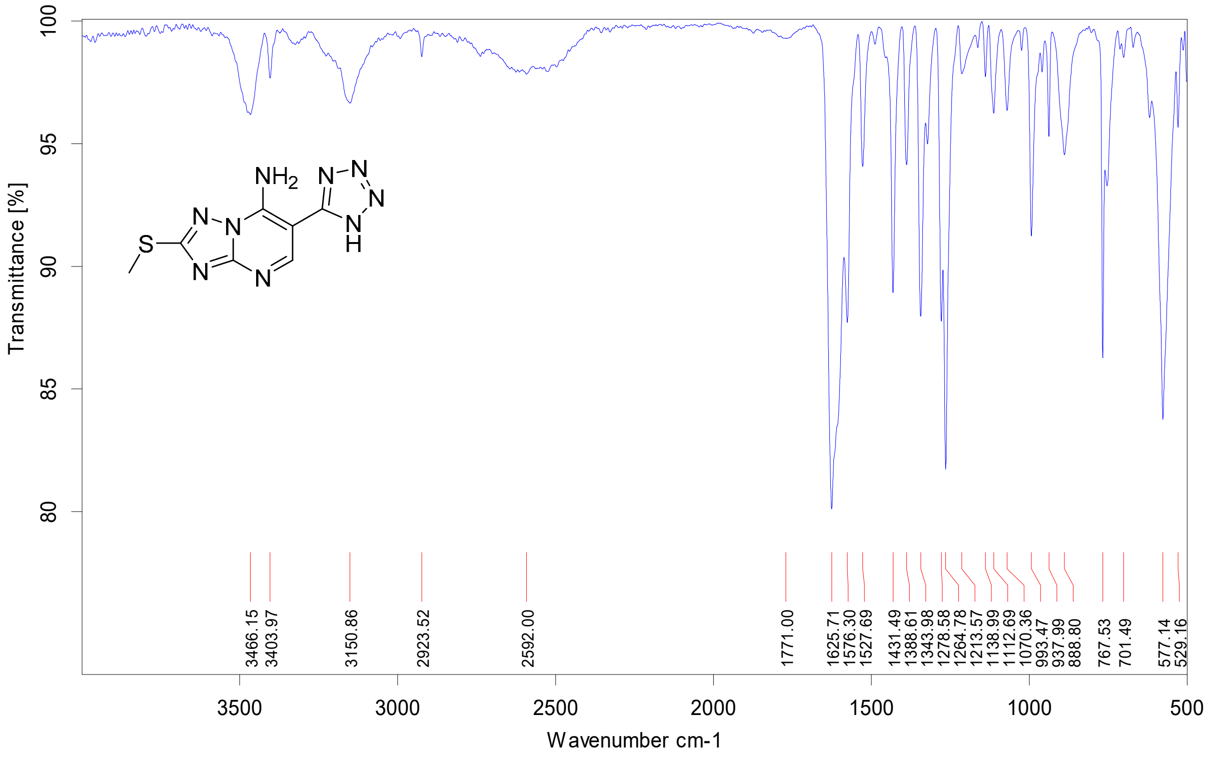

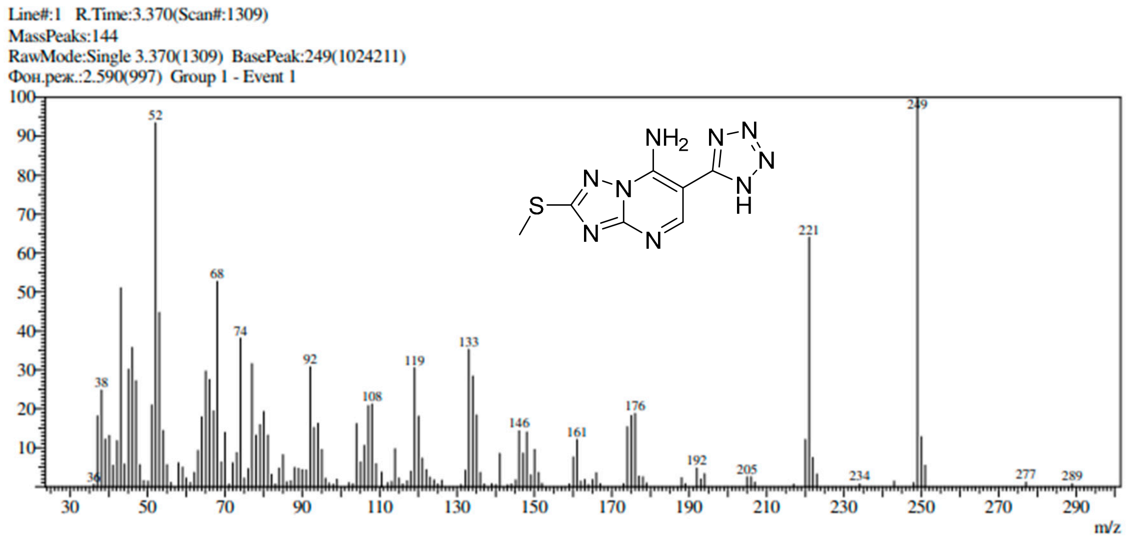

Figure S28. IR and MS (EI, 70 eV) spectra of **10c**

2-(benzylthio)-6-(1H-tetrazol-5-yl)-7-amino-[1,2,4]triazolo[1,5-a]pyrimidine (10d)

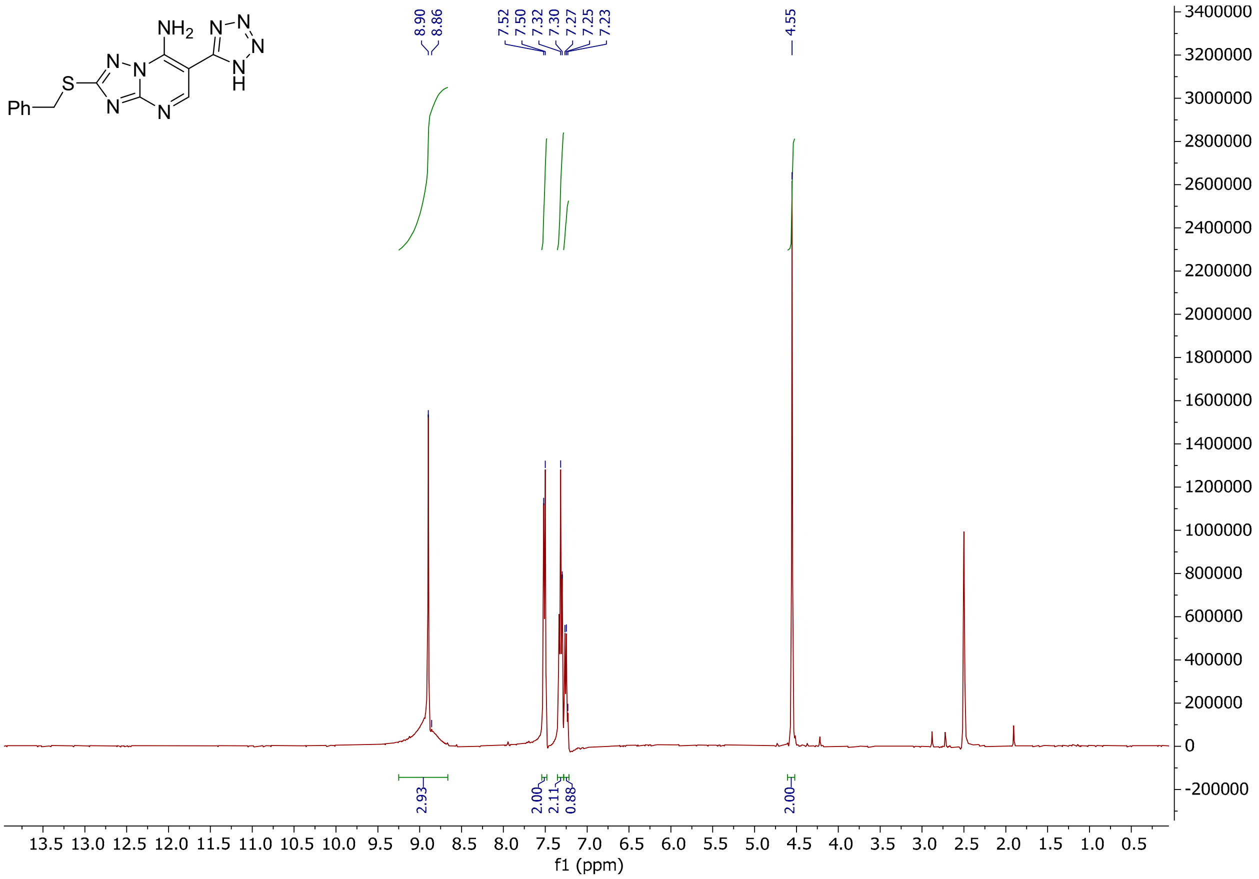

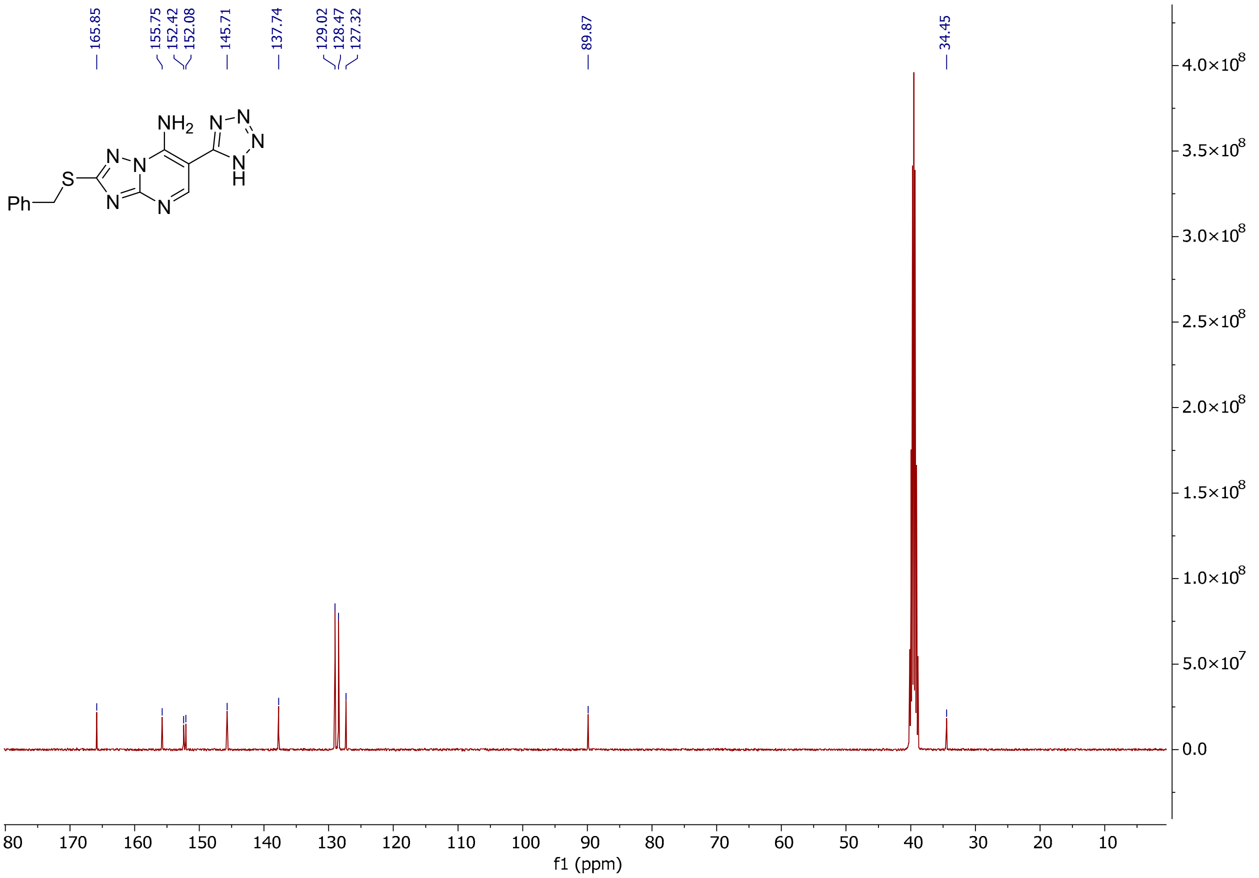

Figure S29. ^{1}H NMR (400 MHz, DMSO-d_6) and ^{13}C NMR (100 MHz, DMSO-d_6) spectra of **10d**

2-(benzylthio)-6-(1H-tetrazol-5-yl)-7-amino-[1,2,4]triazolo[1,5-a]pyrimidine (10d)

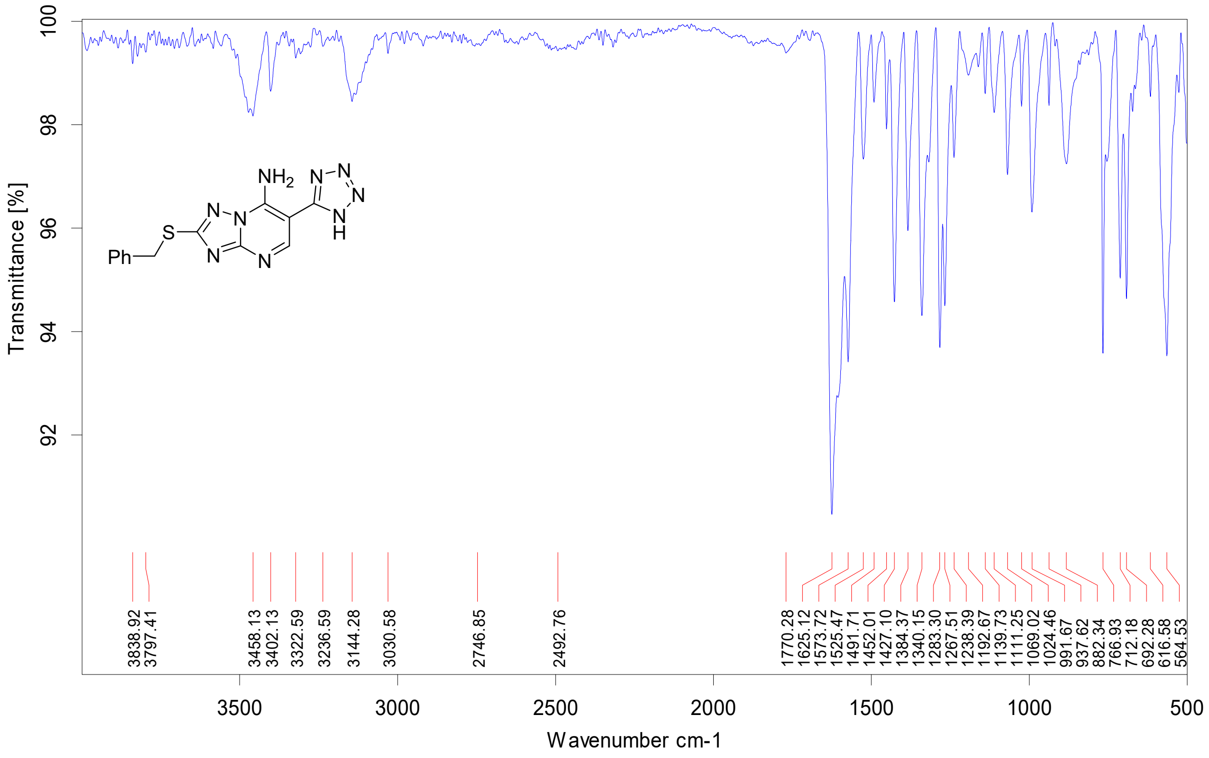

Line#:2 R.Time:4.968(Scan#:1948)
MassPeaks:93
RawMode:Single 4.968(1948) BasePeak:91(4897467)
Фон.реж.:None Group 1 - Event 1

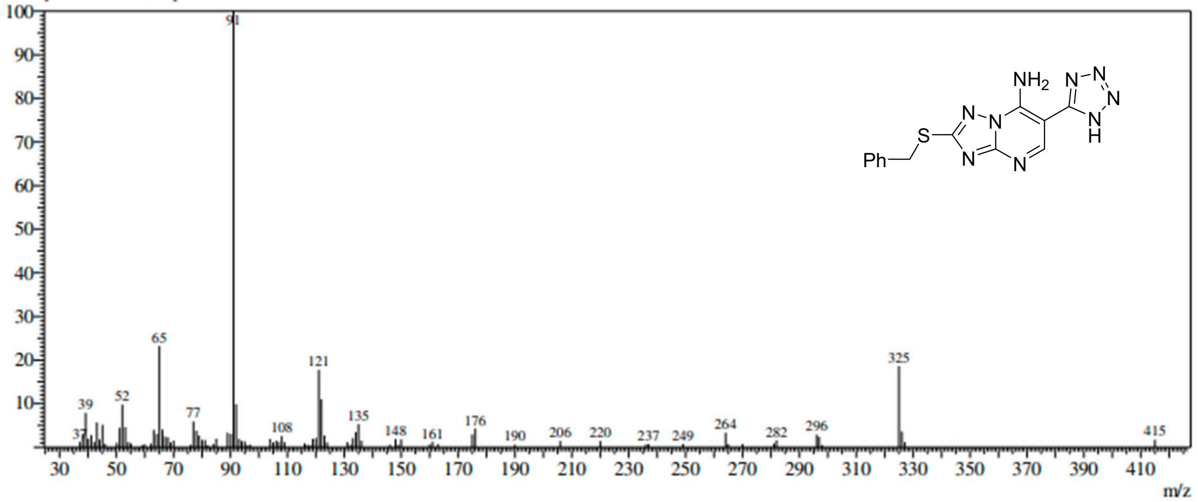

Figure S30. IR and MS (EI, 70 eV) spectra of **10d**

6-(1H-tetrazol-5-yl)-2-(trifluoromethyl)-7-amino-[1,2,4]triazolo[1,5-a]pyrimidine (10e)

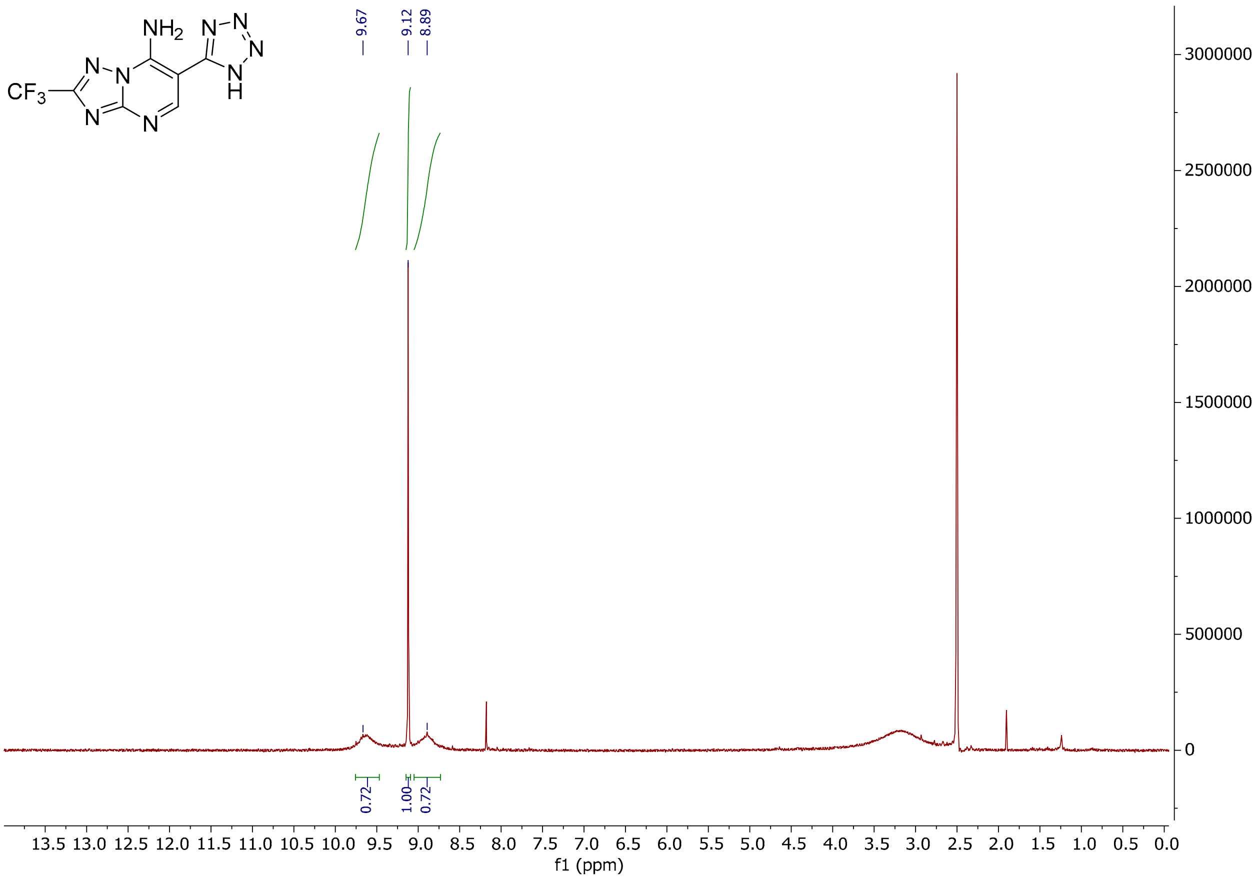

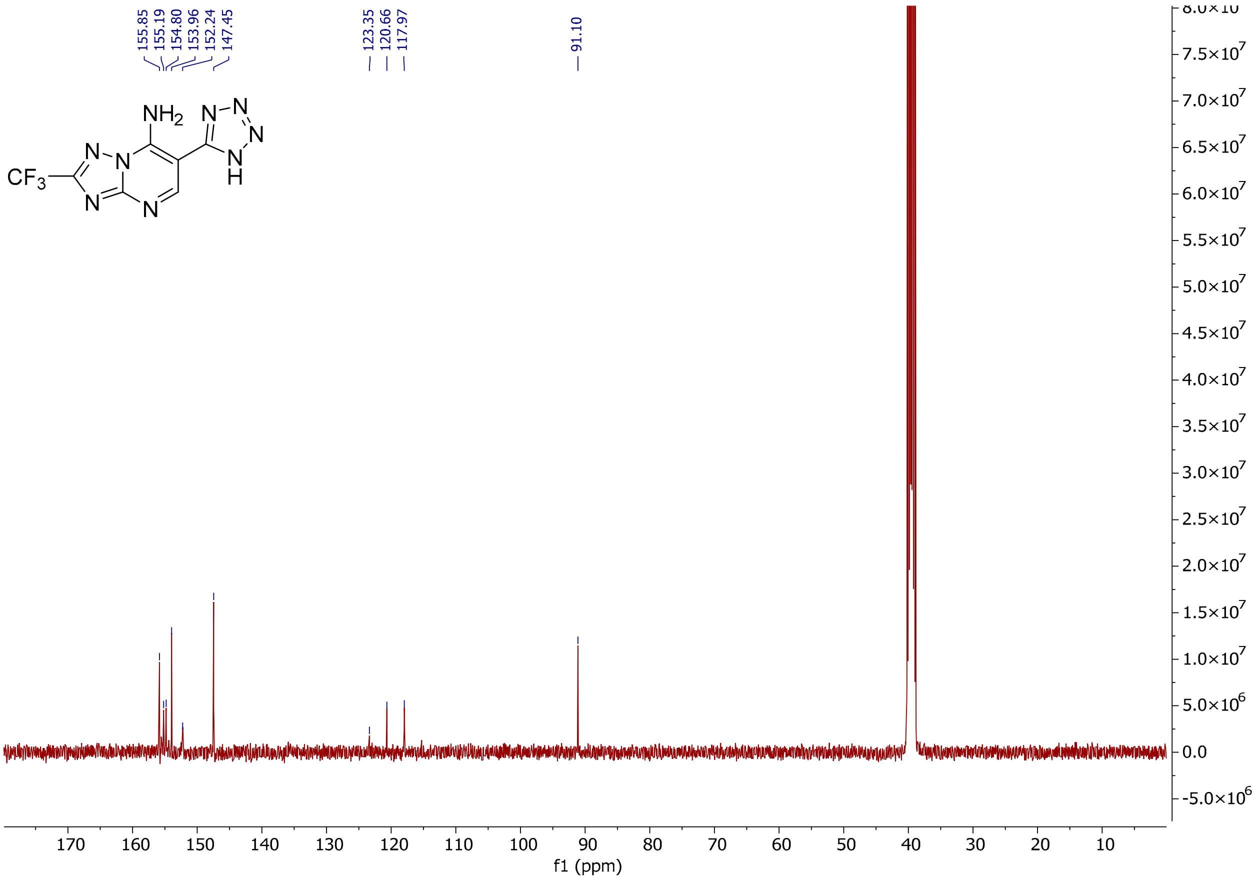

Figure S31. ^{1}H NMR (400 MHz, DMSO-d_6) and ^{13}C NMR (100 MHz, DMSO-d_6) spectra of **10e**

6-(1H-tetrazol-5-yl)-2-(trifluoromethyl)-7-amino-[1,2,4]triazolo[1,5-a]pyrimidine (10e)

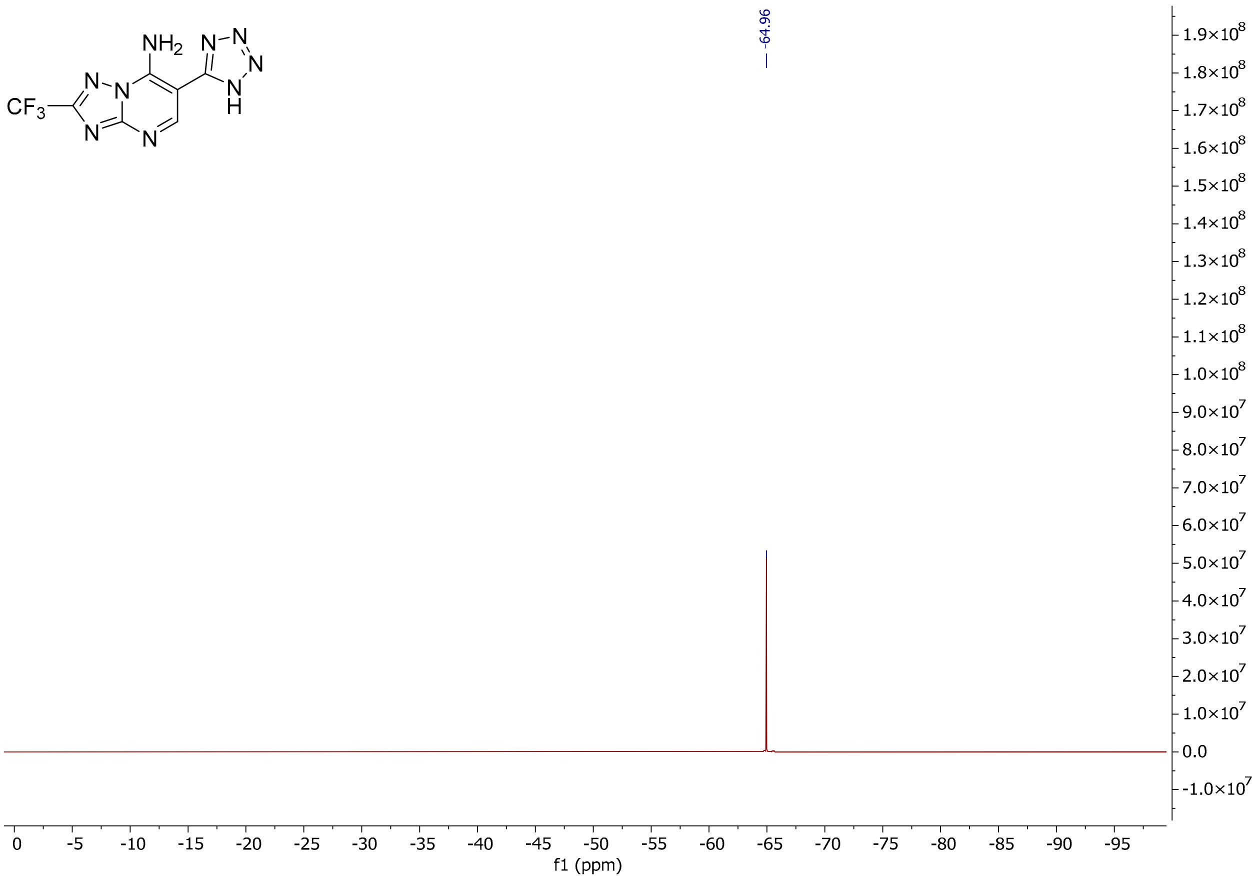

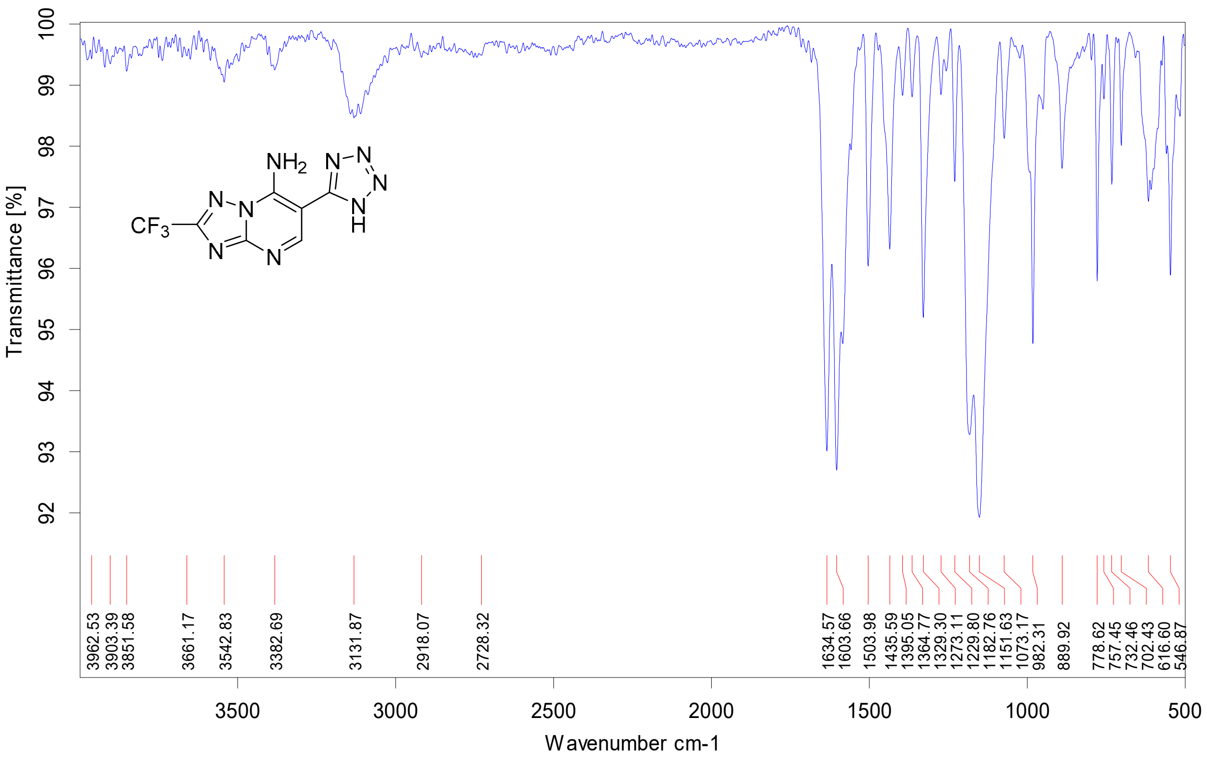

Figure S32. ^{19}F NMR (376 MHz, DMSO-d_6) and IR spectra of **10e**

6-(1H-tetrazol-5-yl)-2-(trifluoromethyl)-7-amino-[1,2,4]triazolo[1,5-a]pyrimidine (10e)

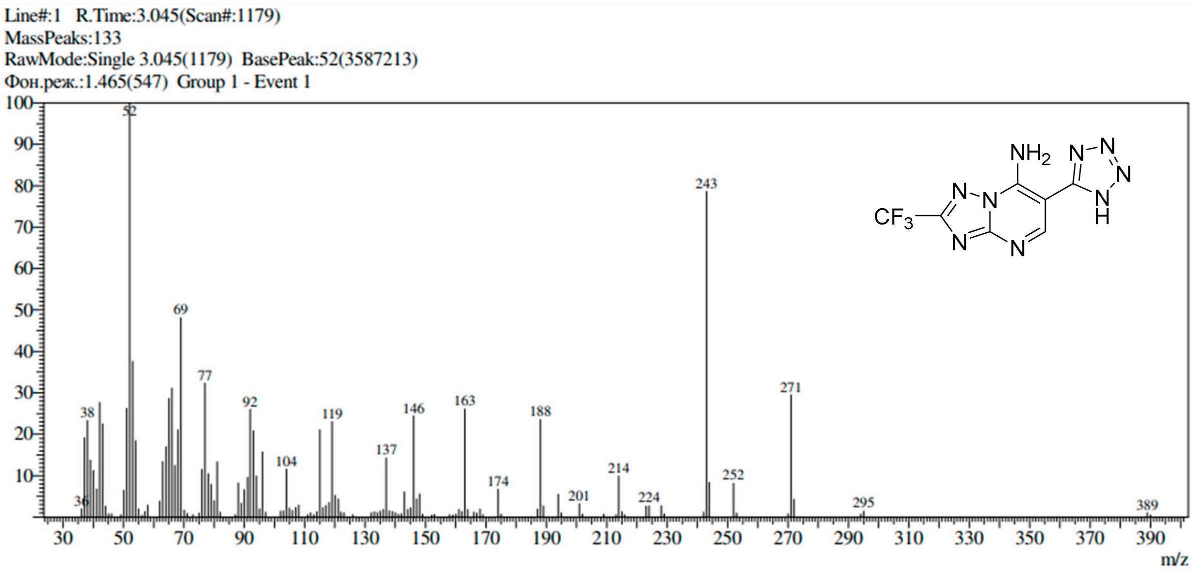

Figure S33. MS (EI, 70 eV) spectra of **10e**

2-ethoxycarbonyl-6-(1H-tetrazol-5-yl)-7-amino-[1,2,4]triazolo[1,5-a]pyrimidine (10f)

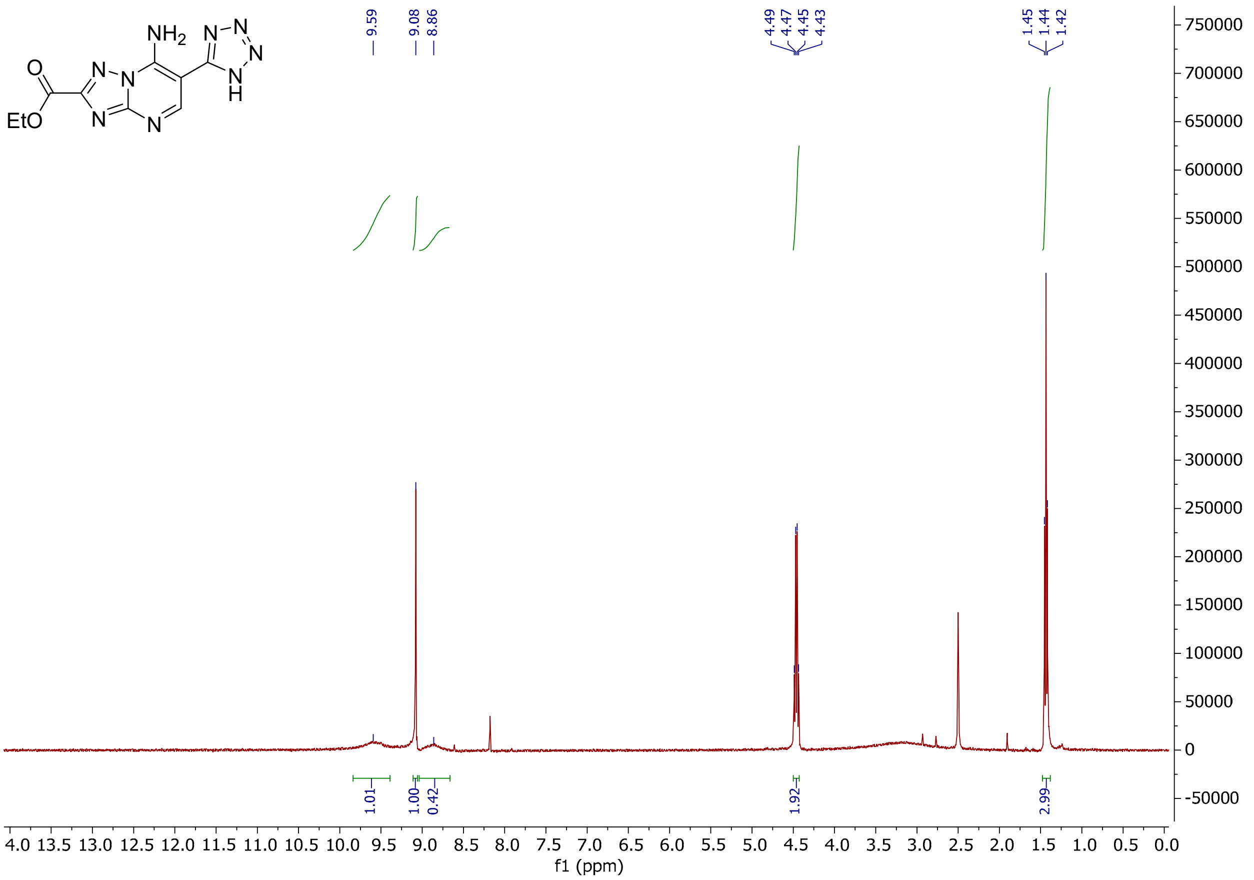

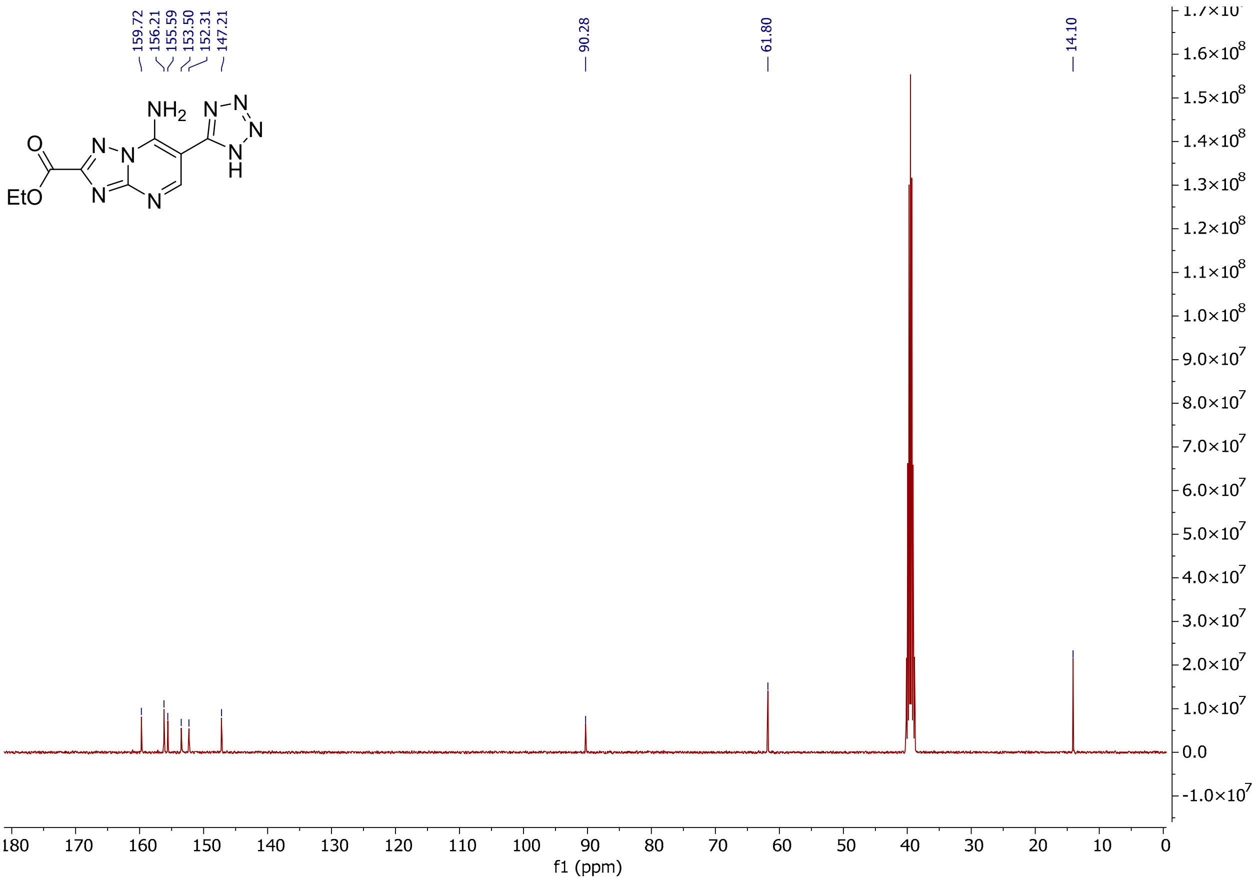

Figure S34. ^{1}H NMR (400 MHz, DMSO-d_6) and ^{13}C NMR (100 MHz, DMSO-d_6) spectra of **10f**

2-ethoxycarbonyl-6-(1H-tetrazol-5-yl)-7-amino-[1,2,4]triazolo[1,5-a]pyrimidine (10f)

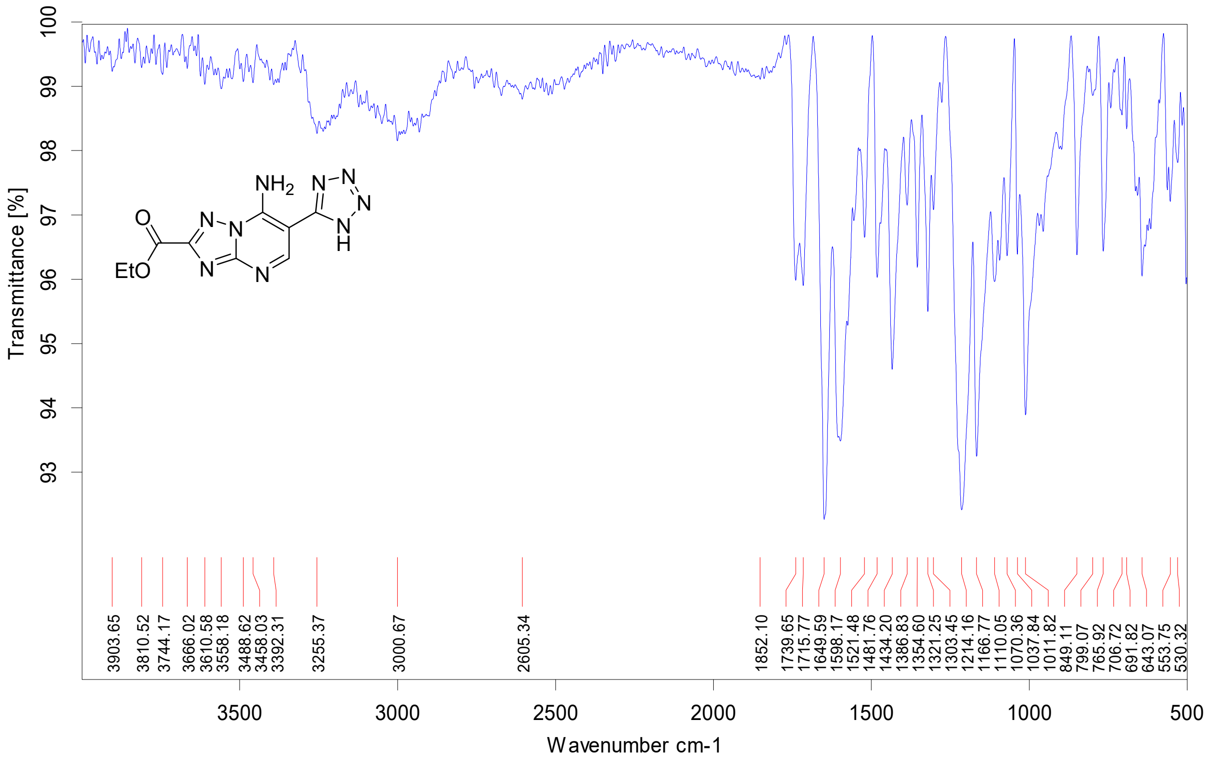

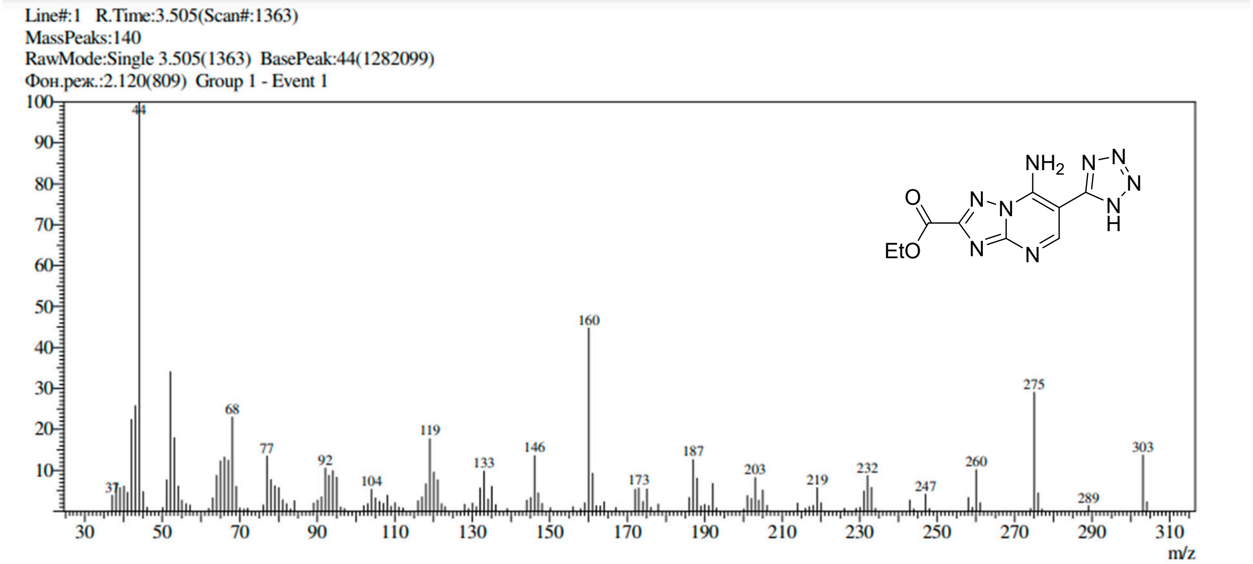

Figure S35. IR and MS (EI, 70 eV) spectra of **10f**

2-phenyl-6-(1H-tetrazol-5-yl)-7-amino-[1,2,4]triazolo[1,5-a]pyrimidine (10g)

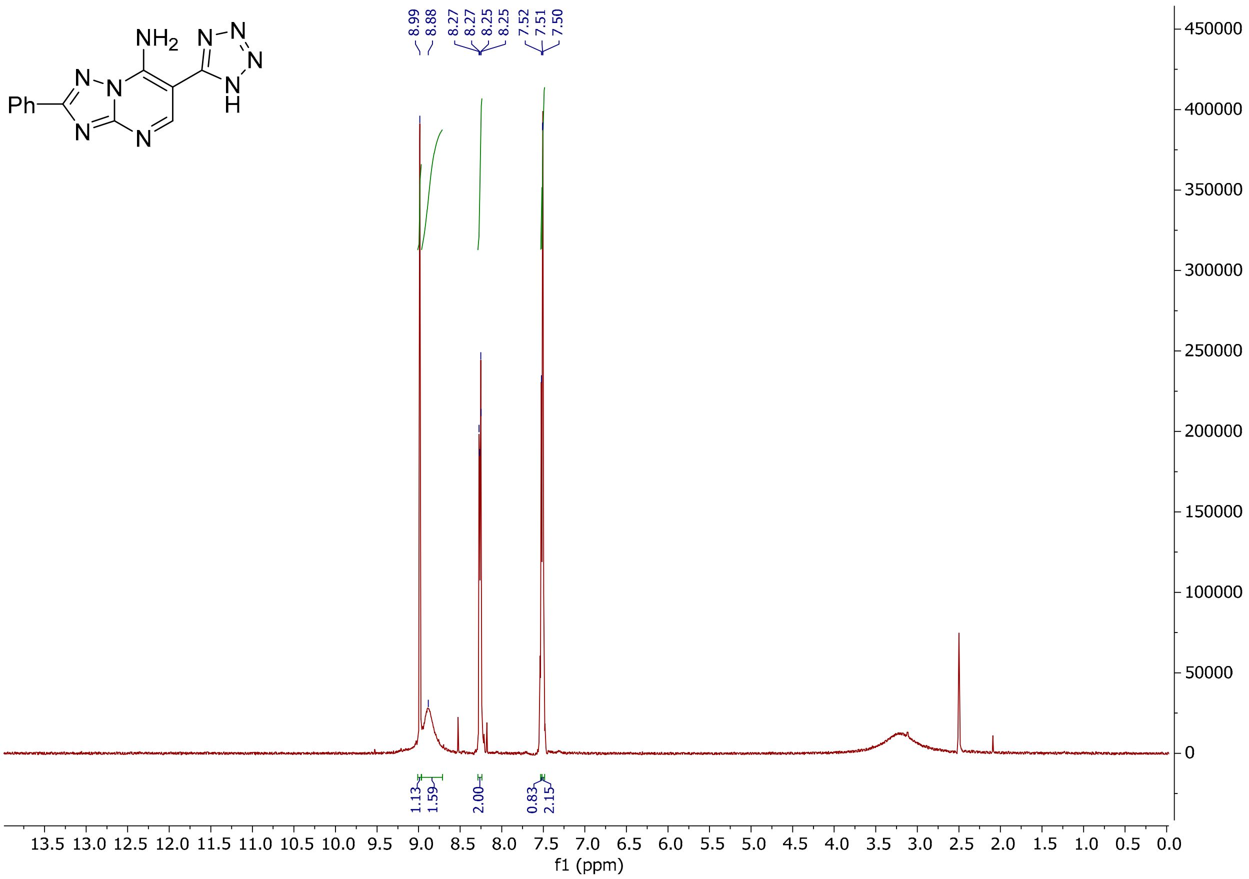

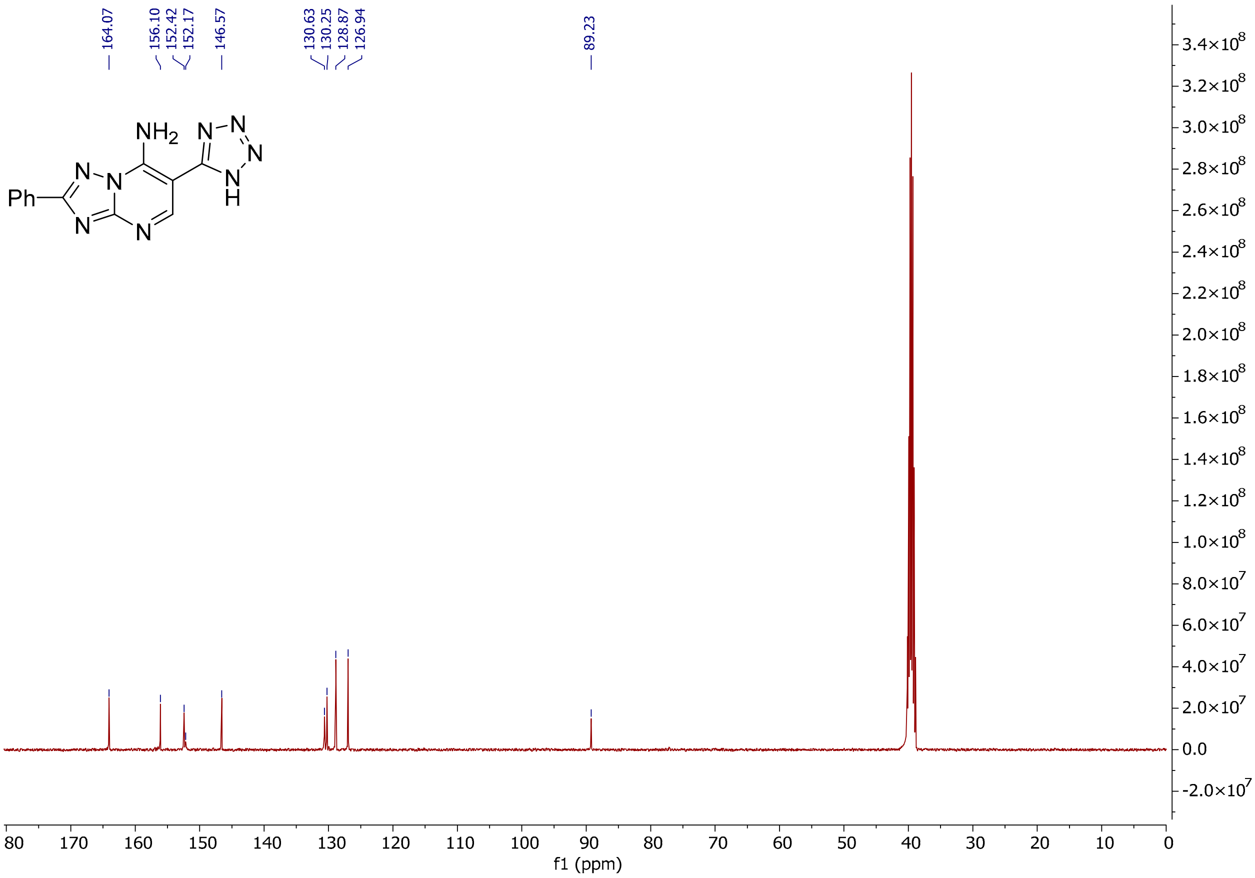

Figure S36. ^{1}H NMR (400 MHz, DMSO-d_6) and ^{13}C NMR (100 MHz, DMSO-d_6) spectra of **10g**

2-phenyl-6-(1H-tetrazol-5-yl)-7-amino-[1,2,4]triazolo[1,5-a]pyrimidine (10g)

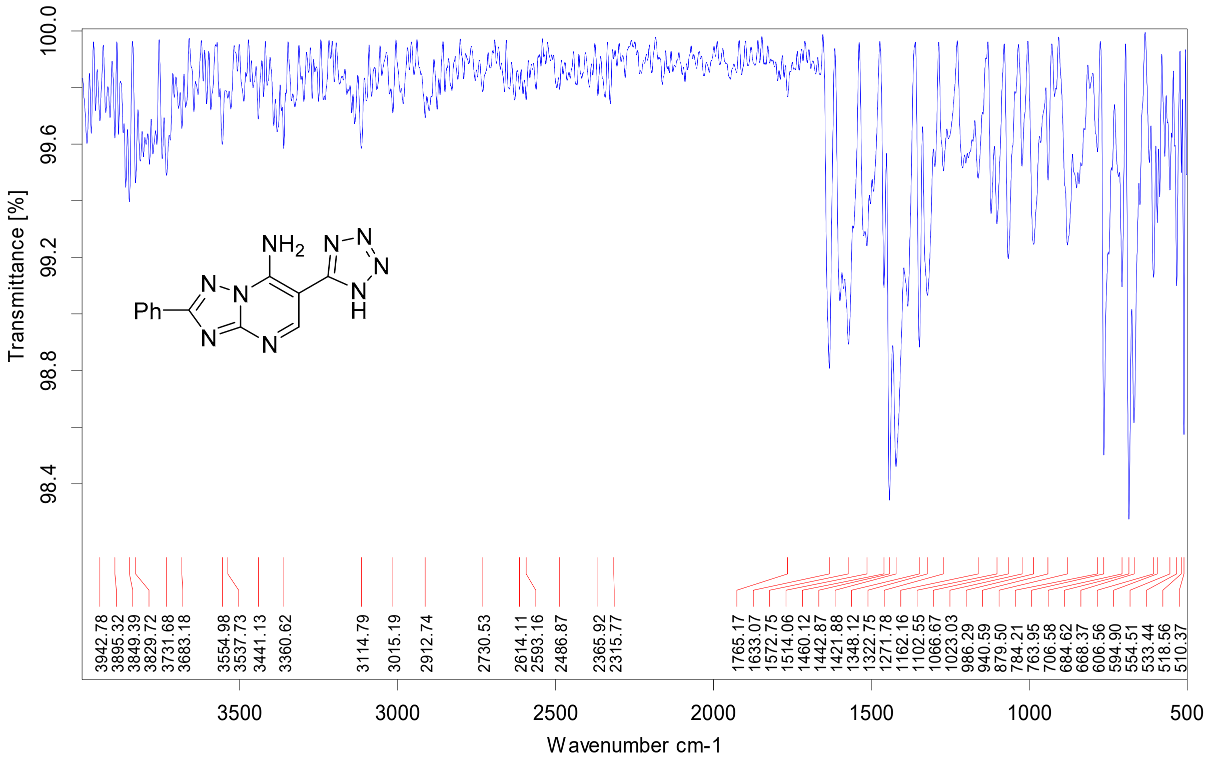

C:\IR-spec\KOrCh\2022\7\TS-213.0000 TS-213	27/05/2003

Page 1/1

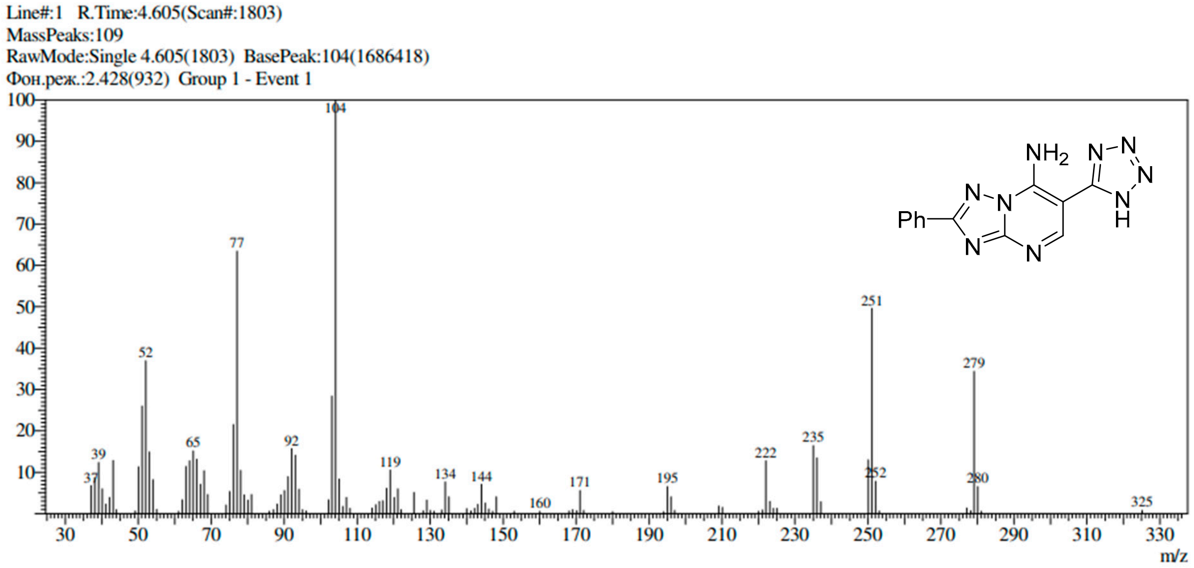

Figure S37. IR and MS (EI, 70 eV) spectra of **10g**

2-(furan-2-yl)-6-(1H-tetrazol-5-yl)-7-amino-[1,2,4]triazolo[1,5-a]pyrimidine (10h)

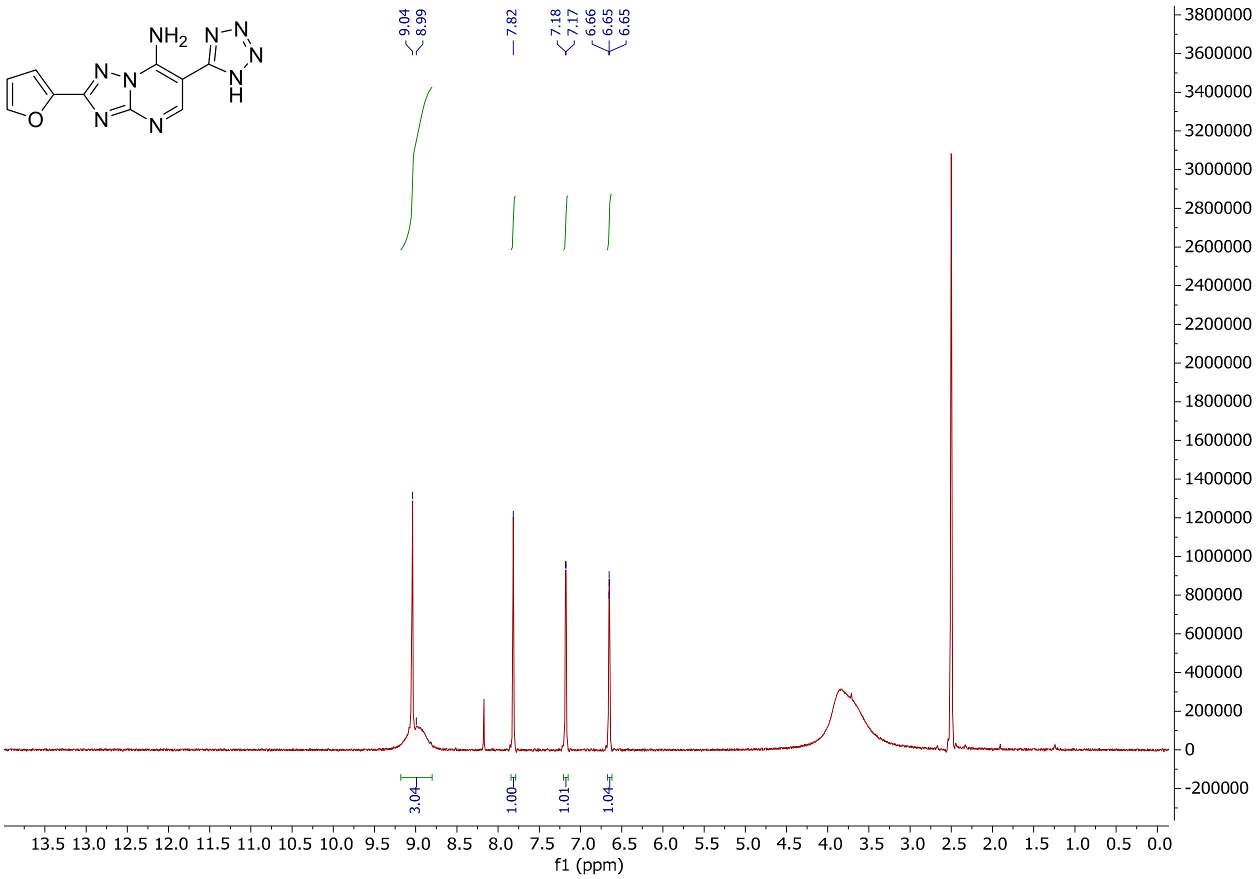

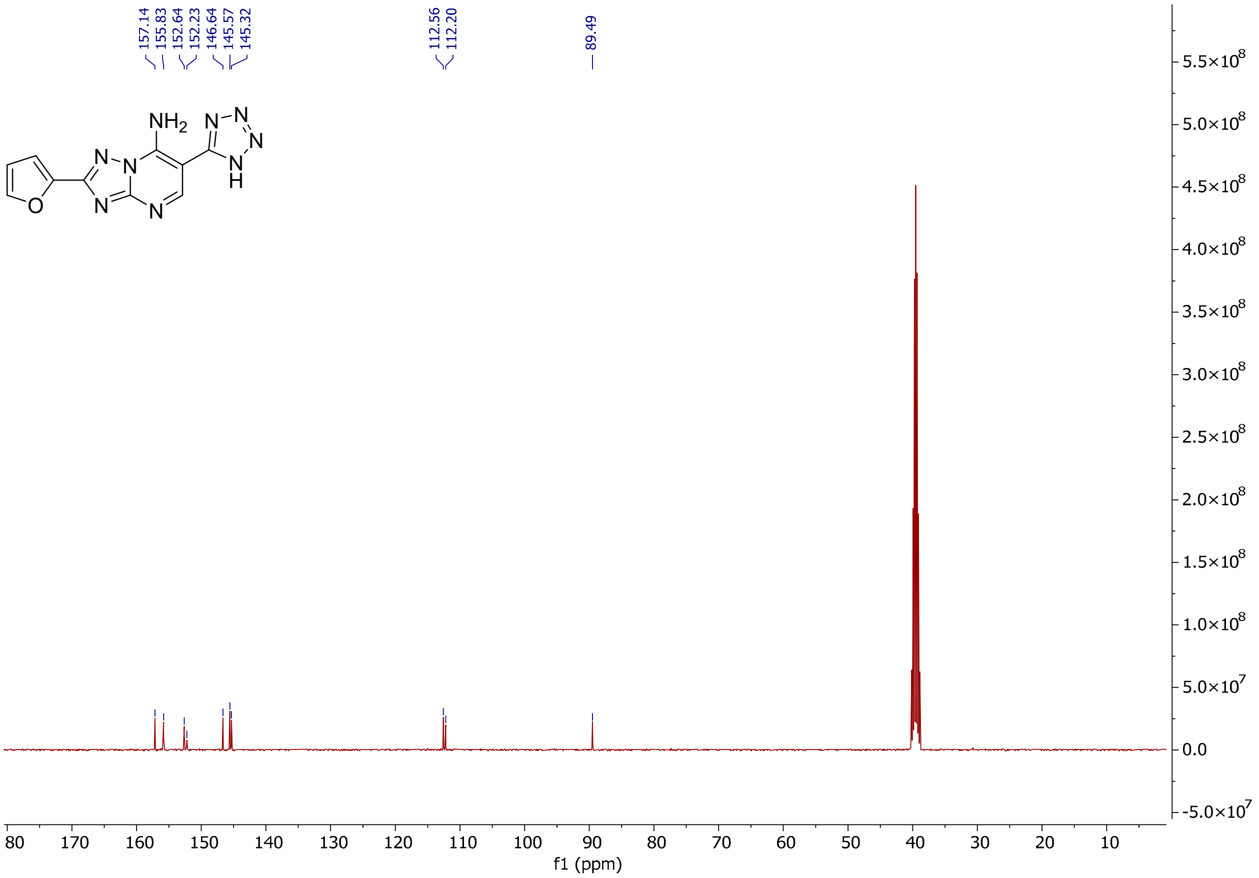

Figure S38. ^{1}H NMR (400 MHz, DMSO-d_6) and ^{13}C NMR (100 MHz, DMSO-d_6) spectra of **10h**

2-(furan-2-yl)-6-(1H-tetrazol-5-yl)-7-amino-[1,2,4]triazolo[1,5-a]pyrimidine (10h)

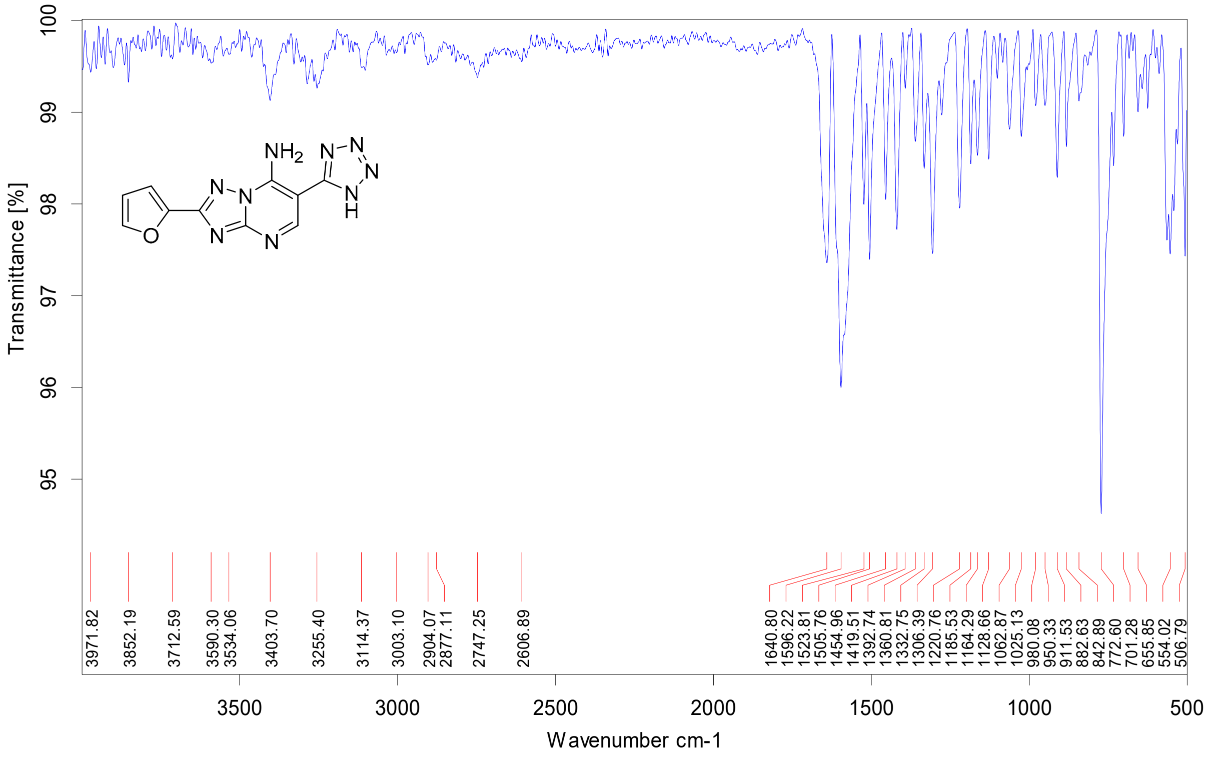

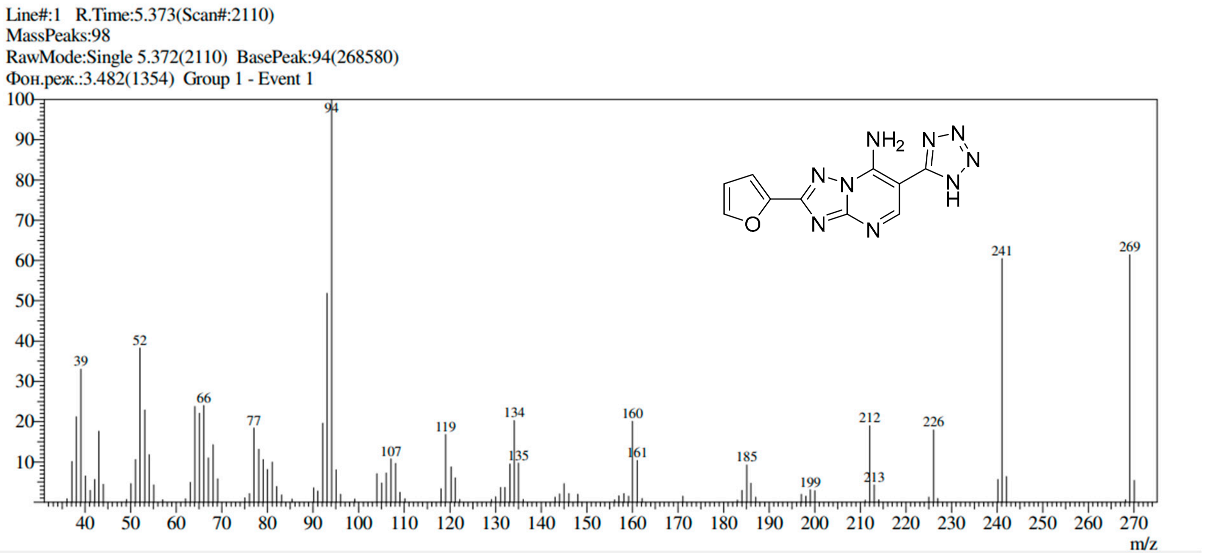

Figure S39. IR and MS (EI, 70 eV) spectra of **10h**

2-(thiophen-2-yl)-6-(1H-tetrazol-5-yl)-7-amino-[1,2,4]triazolo[1,5-a]pyrimidine (10i)

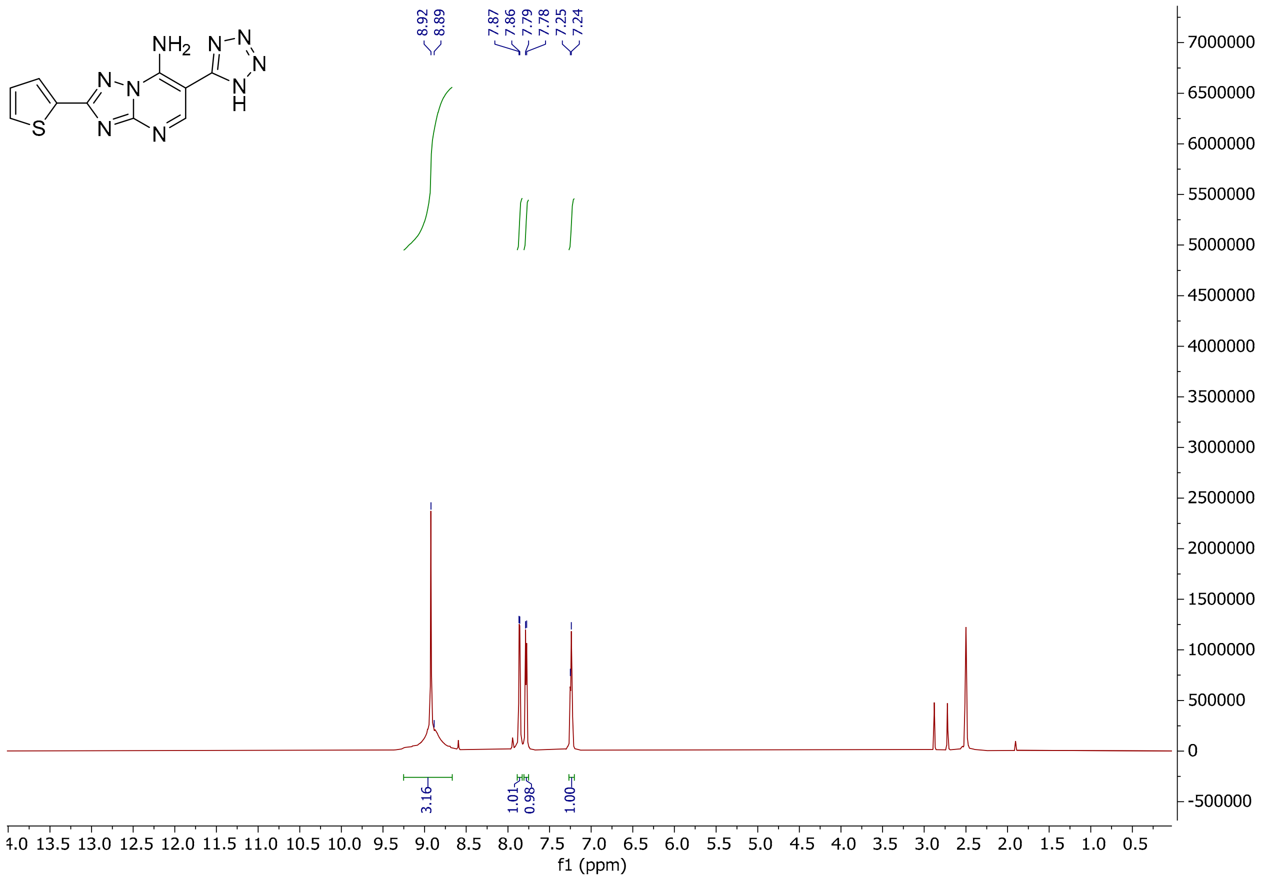

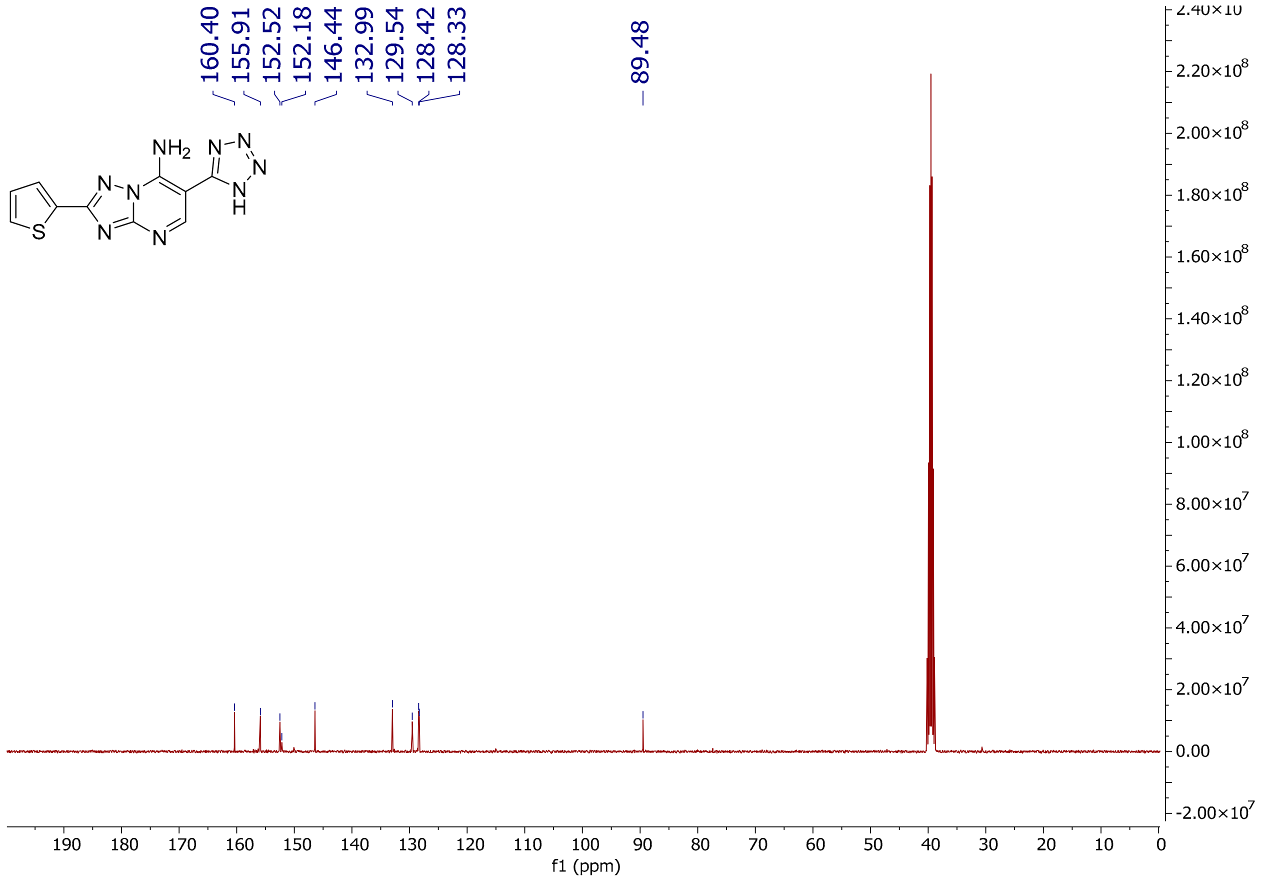

Figure S40. ^{1}H NMR (400 MHz, DMSO-d_6) and ^{13}C NMR (100 MHz, DMSO-d_6) spectra of **10i**

2-(thiophen-2-yl)-6-(1H-tetrazol-5-yl)-7-amino-[1,2,4]triazolo[1,5-a]pyrimidine (10i)

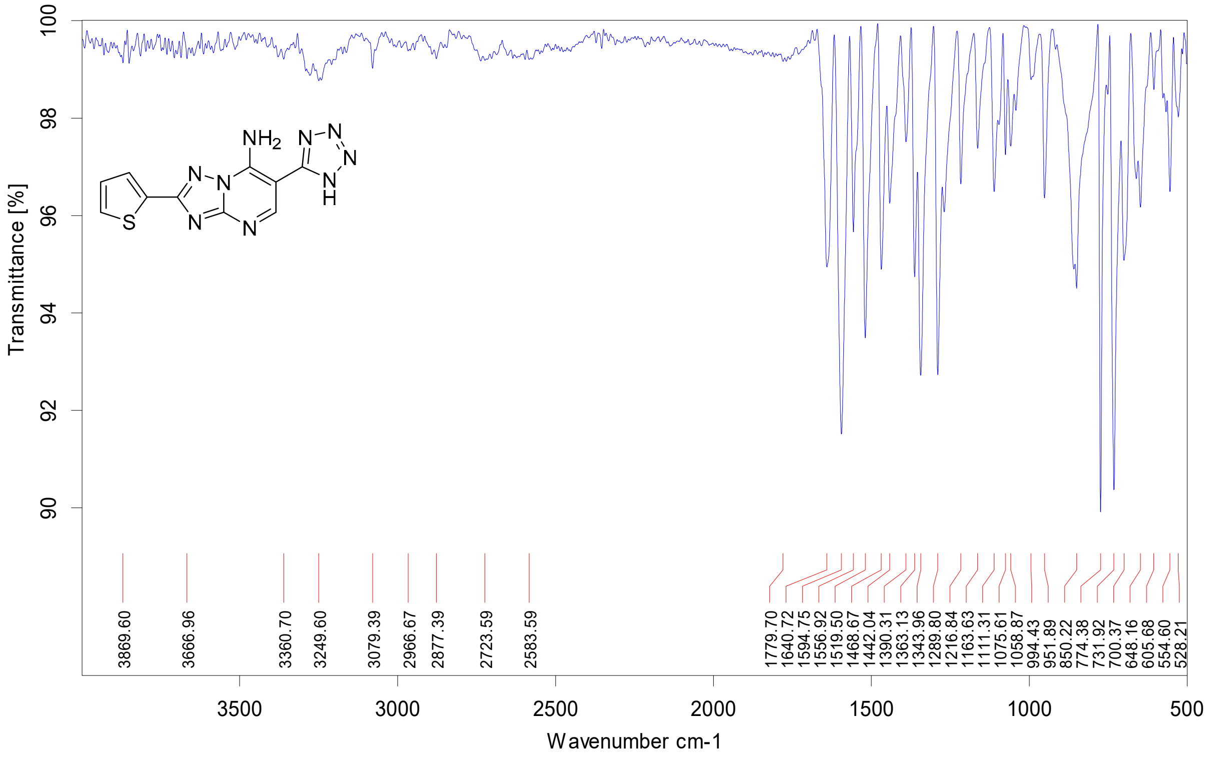

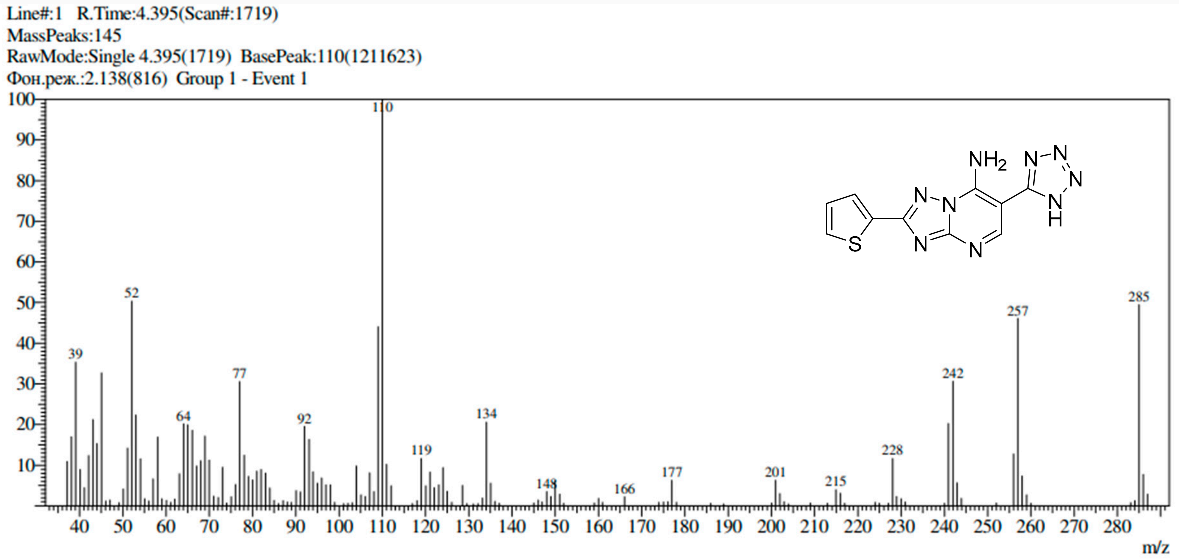

Figure S41. IR and MS (EI, 70 eV) spectra of **10i**

2-(pyridin-3-yl)-6-(1H-tetrazol-5-yl)-7-amino-[1,2,4]triazolo[1,5-a]pyrimidine (10j)

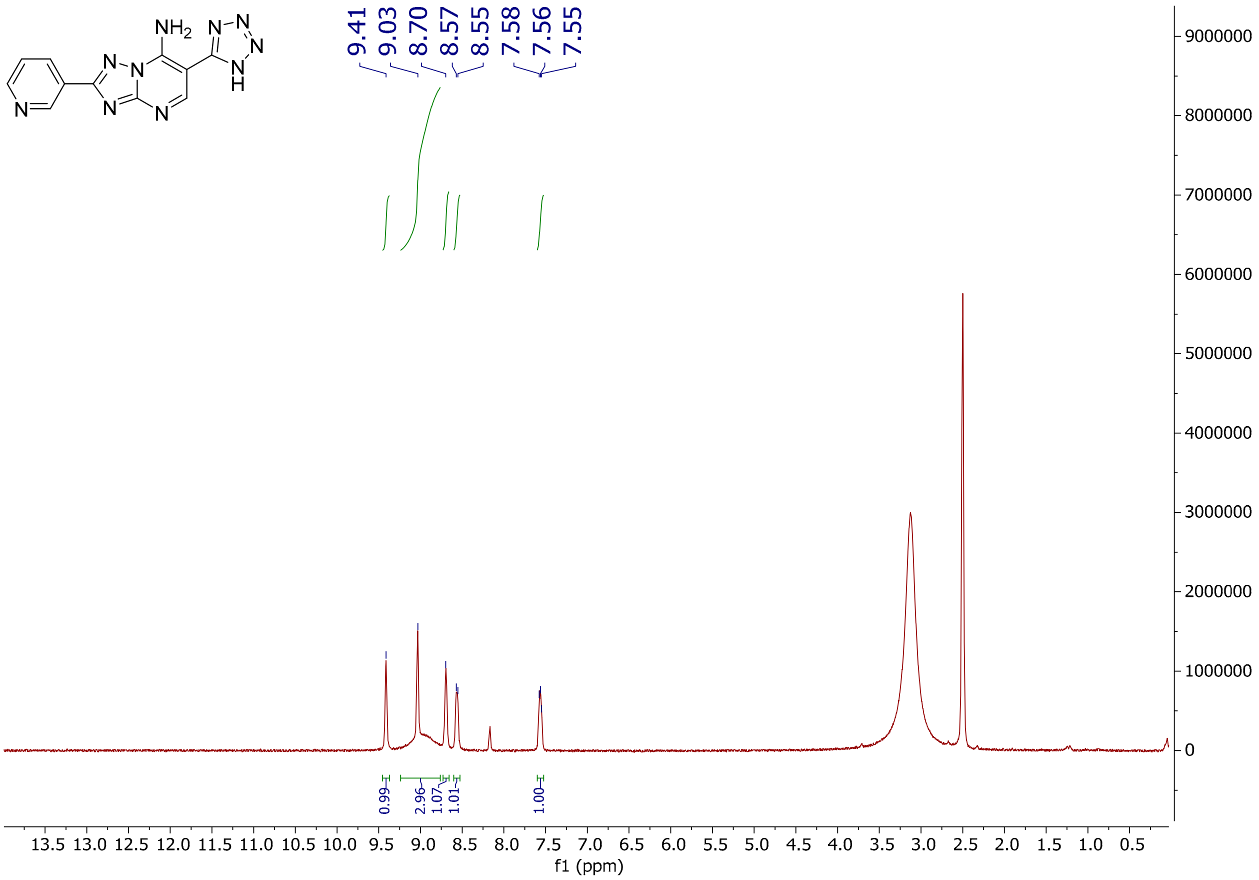

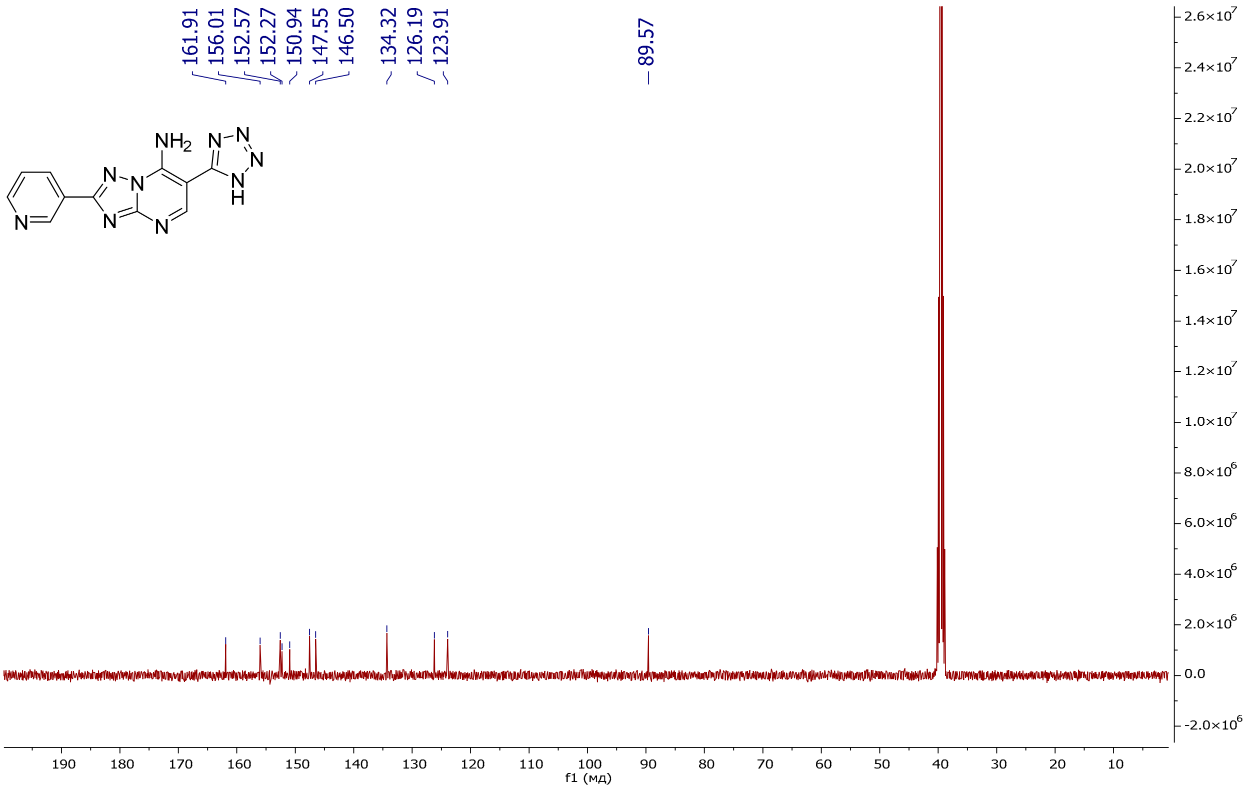

Figure S42. ^{1}H NMR (400 MHz, DMSO-d_6) and ^{13}C NMR (100 MHz, DMSO-d_6) spectra of **10j**

2-(pyridin-3-yl)-6-(1H-tetrazol-5-yl)-7-amino-[1,2,4]triazolo[1,5-a]pyrimidine (10j)

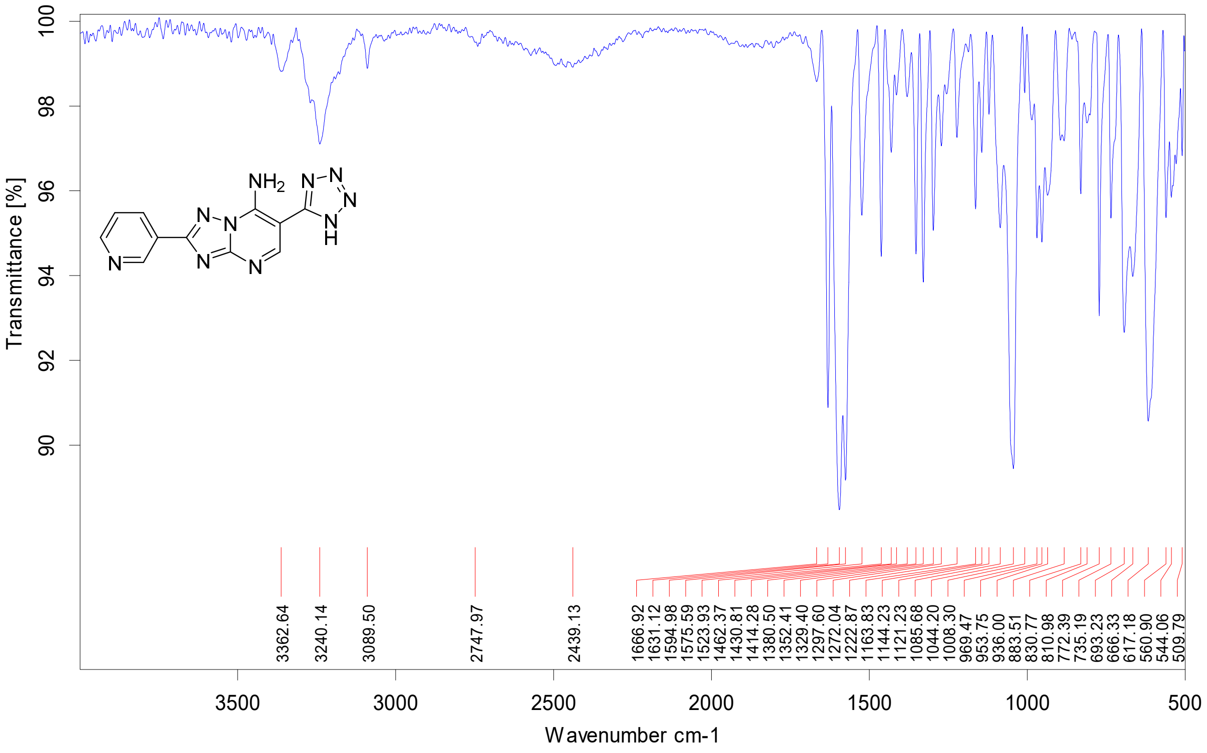

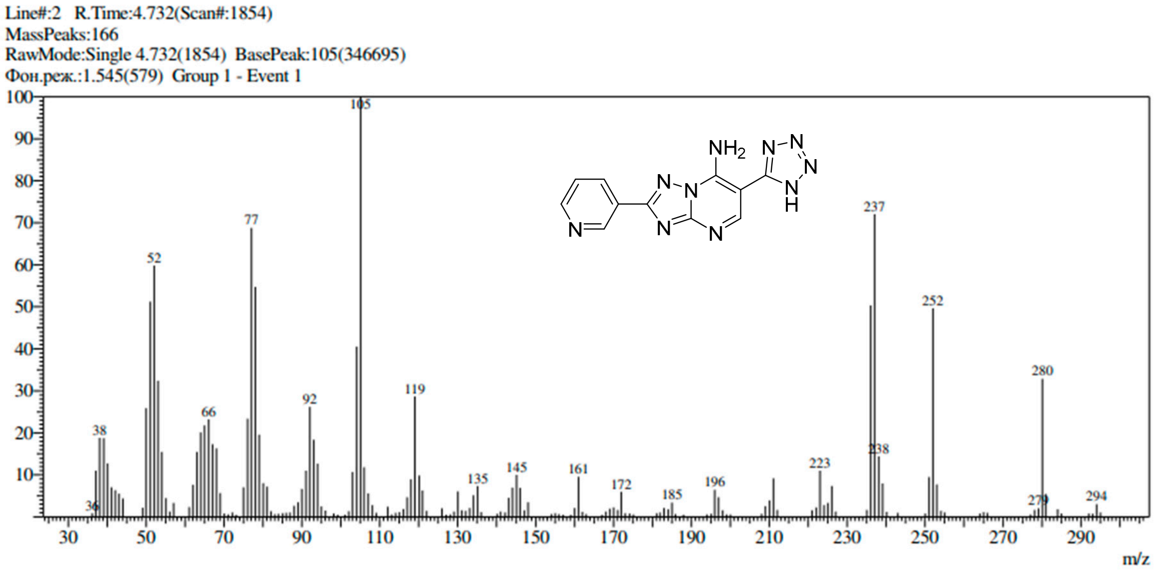

Figure S43. IR and MS (EI, 70 eV) spectra of **10j**

6-(1H-tetrazol-5-yl)-2,7-diamino-[1,2,4]triazolo[1,5-a]pyrimidine (10k)

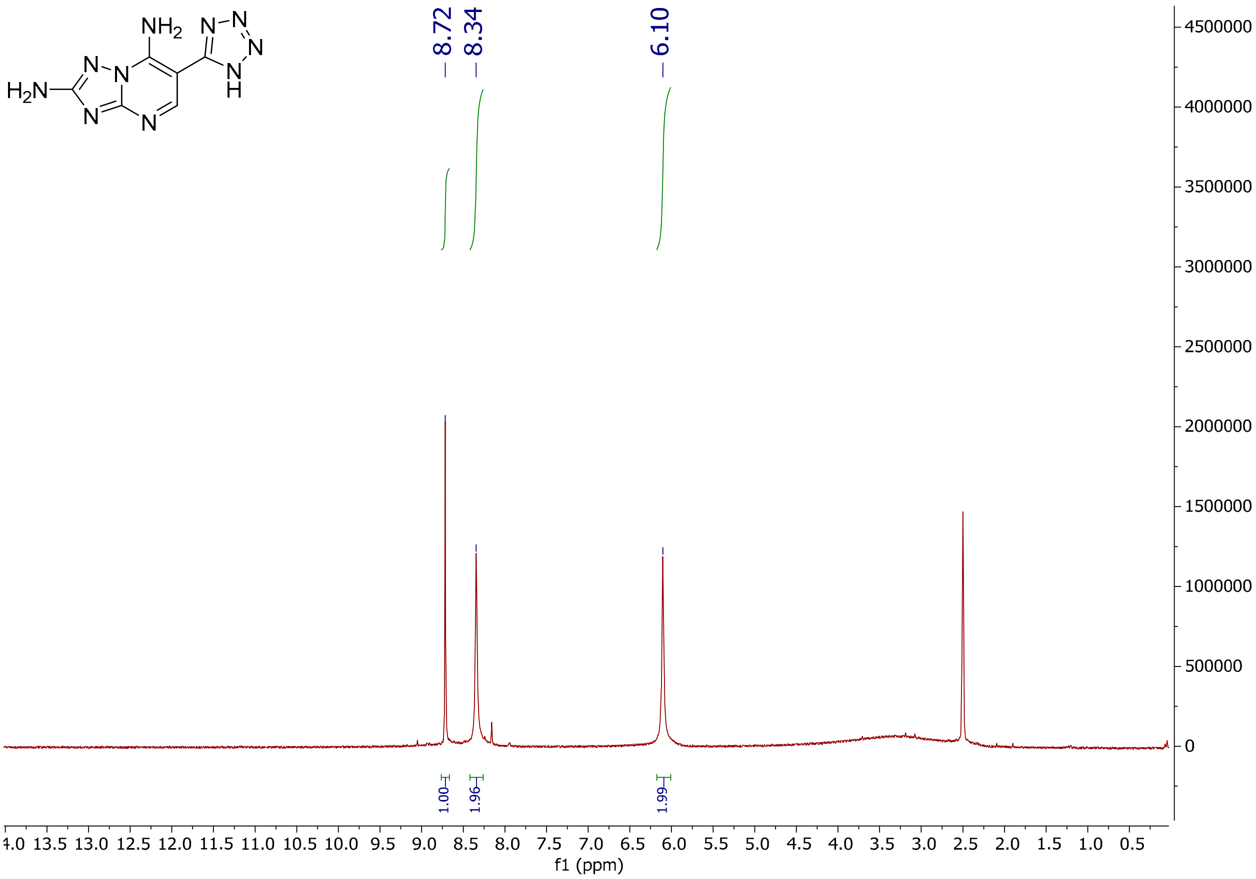

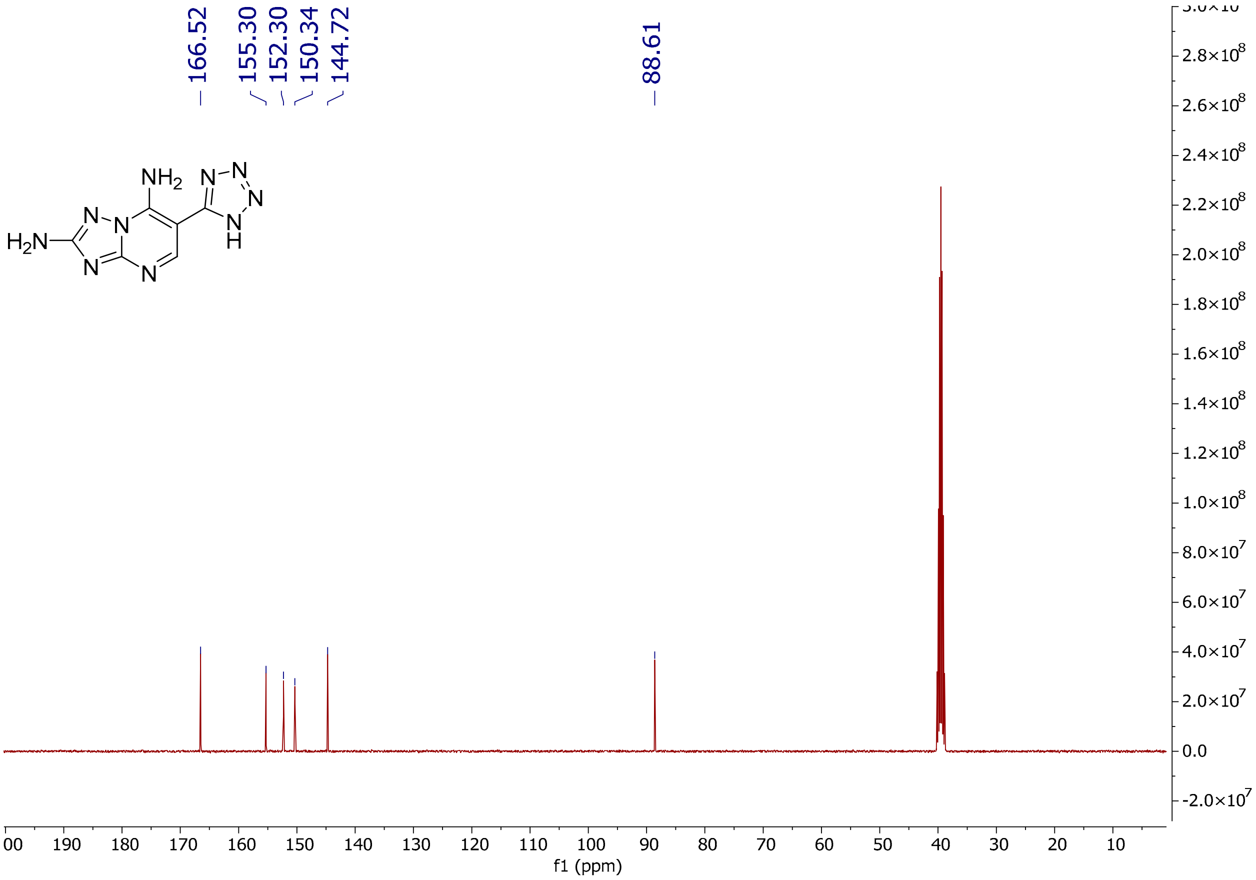

Figure S44. ^{1}H NMR (400 MHz, DMSO-d_6) and ^{13}C NMR (100 MHz, DMSO-d_6) spectra of **10k**

6-(1H-tetrazol-5-yl)-2,7-diamino-[1,2,4]triazolo[1,5-a]pyrimidine (10k)

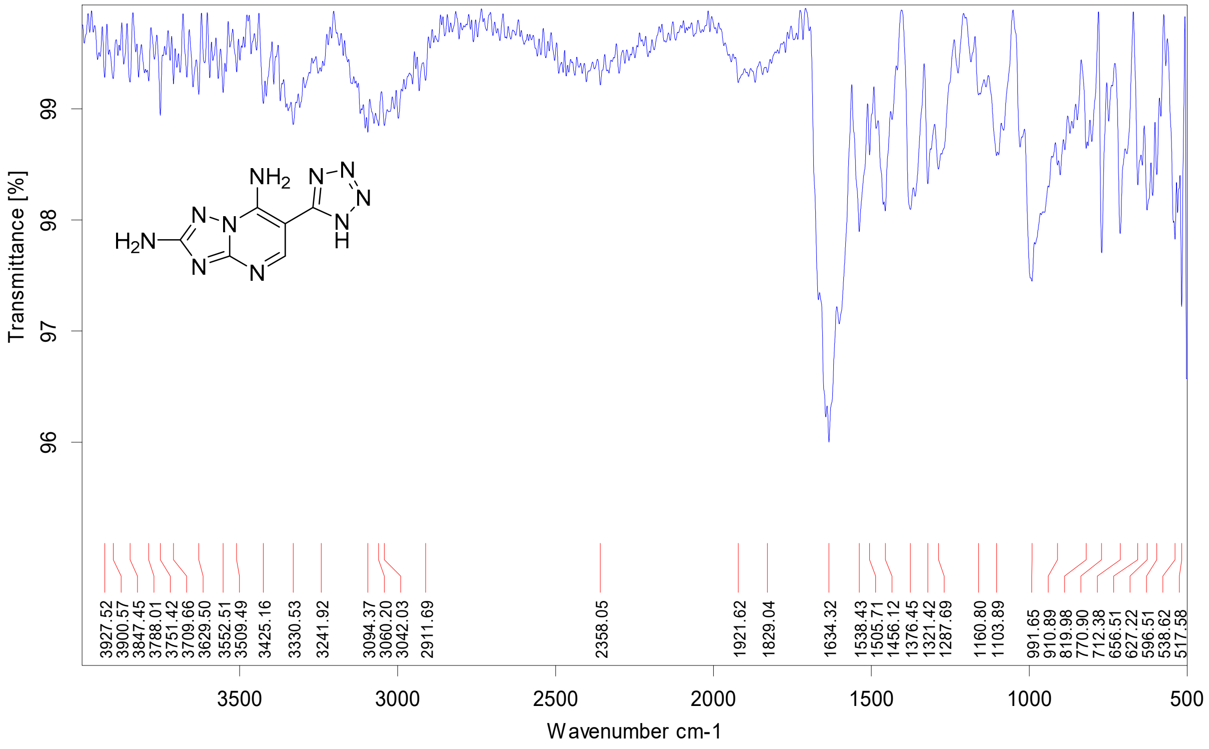

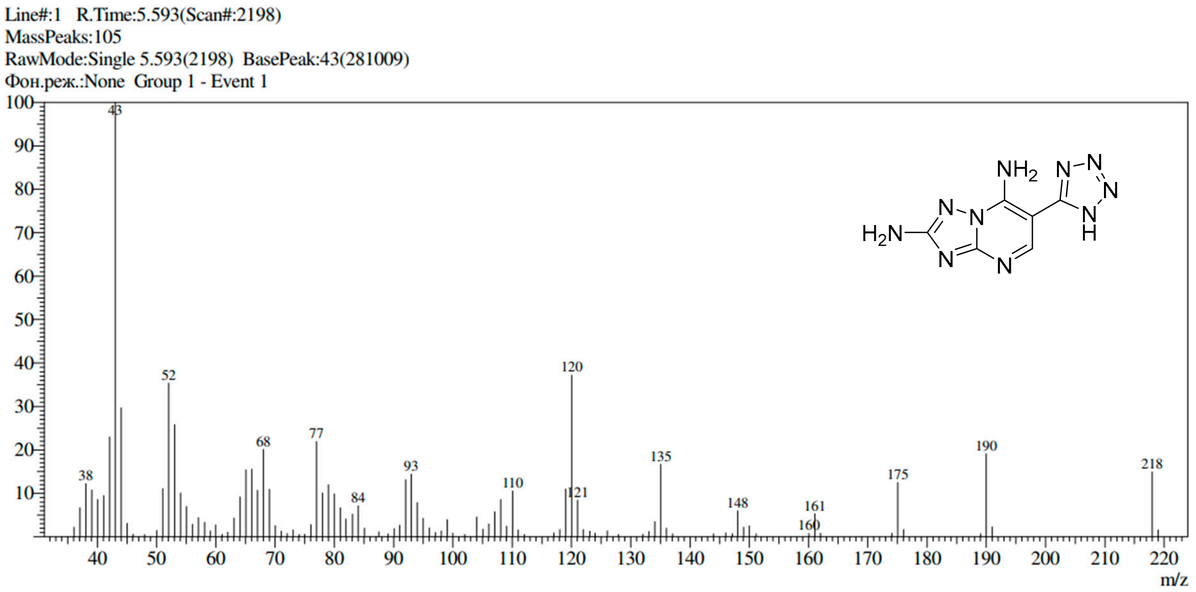

Figure S45. IR and MS (EI, 70 eV) spectra of **10k**

Sodium 5-(7-aminopyrazolo[1,5-a]pyrimidin-6-yl)tetrazol-1-ide (3a)

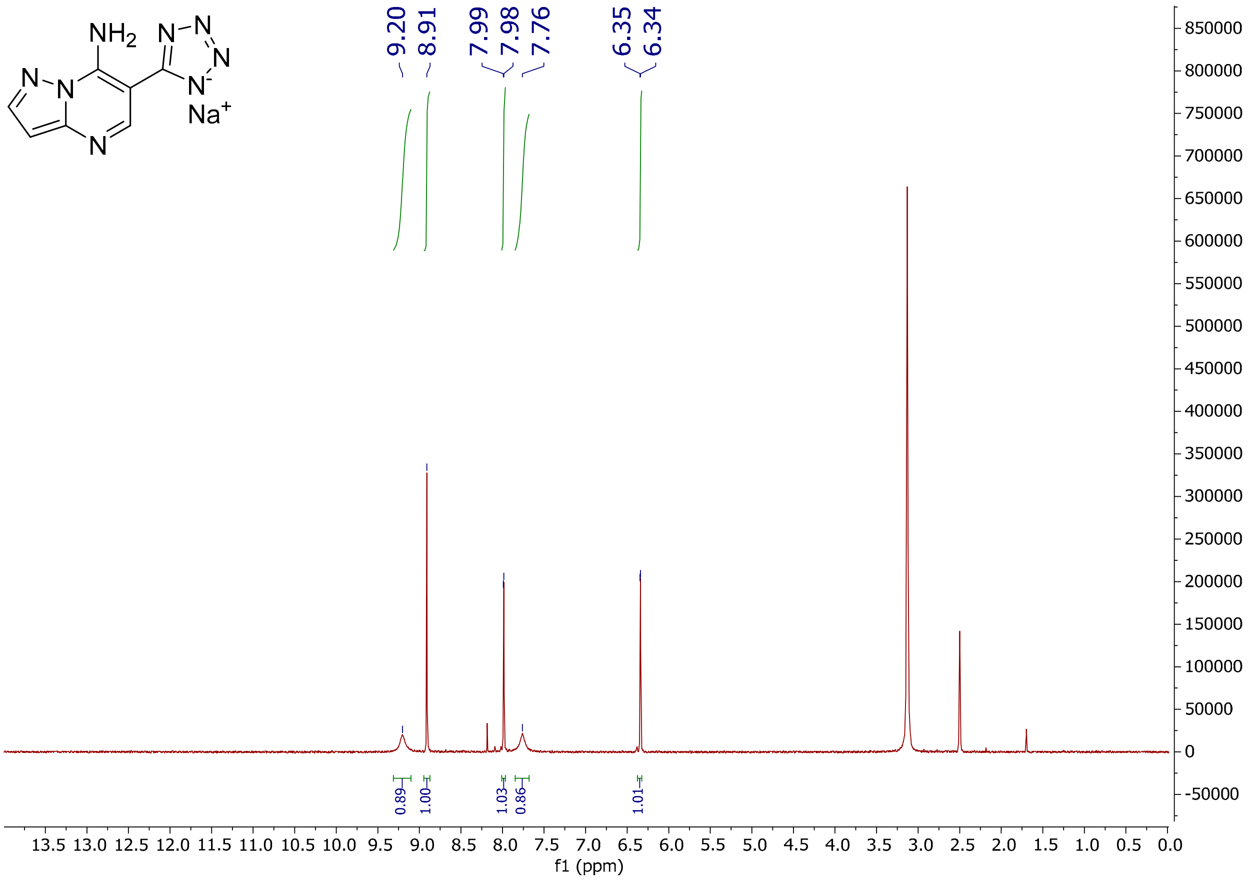

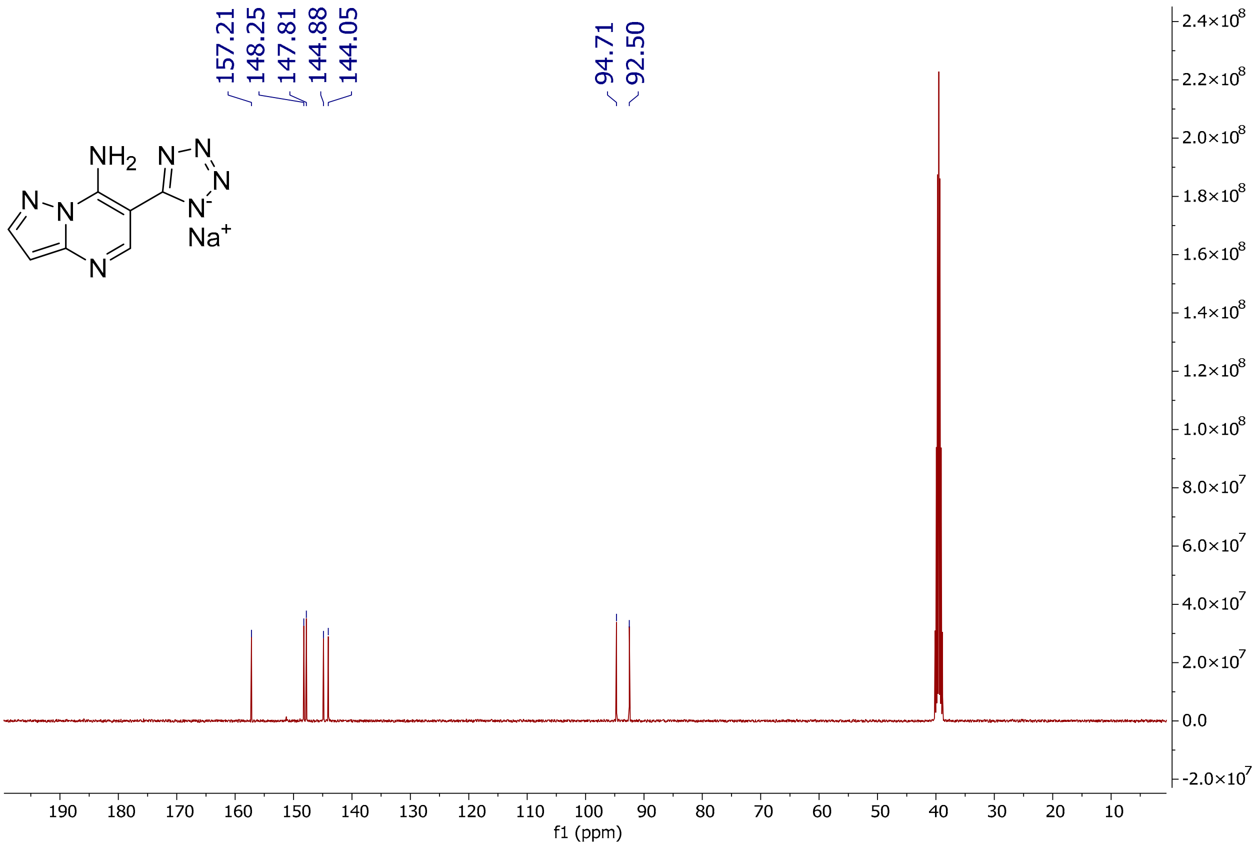

Figure S46. ^{1}H NMR (400 MHz, DMSO-d_6) and ^{13}C NMR (100 MHz, DMSO-d_6) spectra of **3a**

Sodium 5-(7-aminopyrazolo[1,5-a]pyrimidin-6-yl)tetrazol-1-ide (3a)

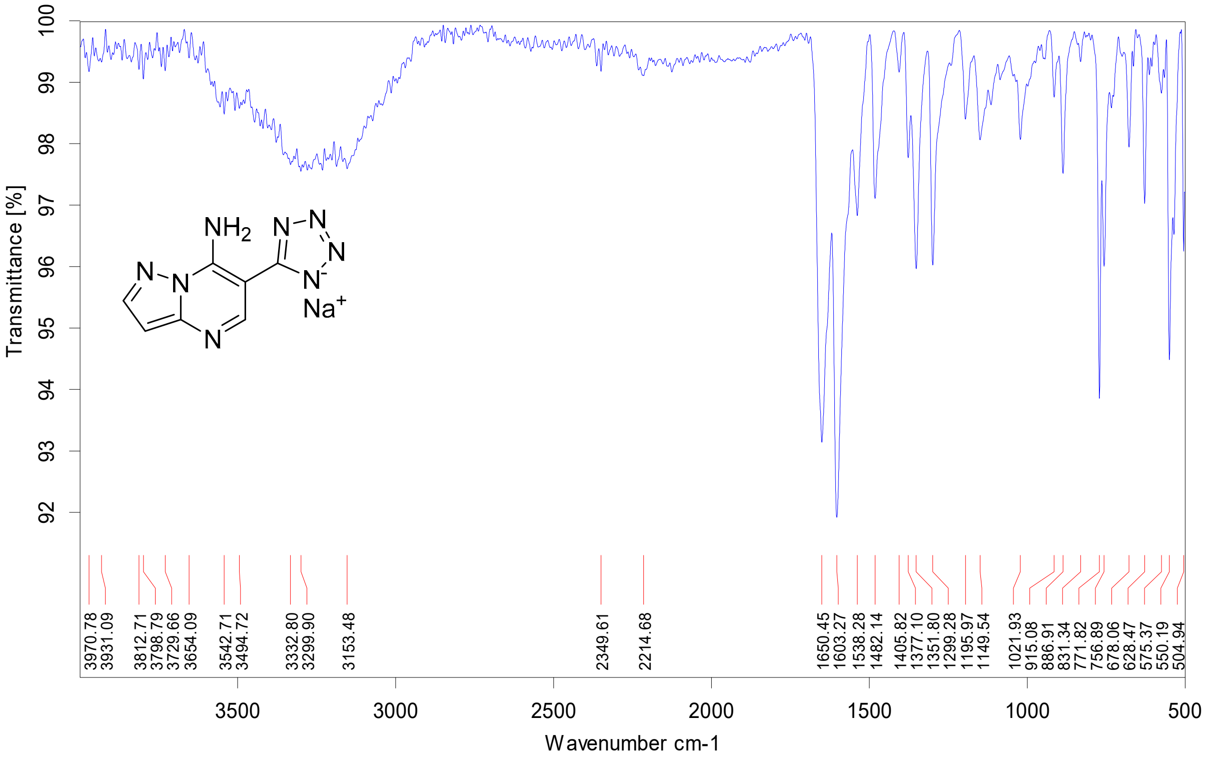

Figure S47. IR spectra of **3a**

Sodium 5-(7-amino-2-methylpyrazolo[1,5-a]pyrimidin-6-yl)tetrazol-1-ide (3b)

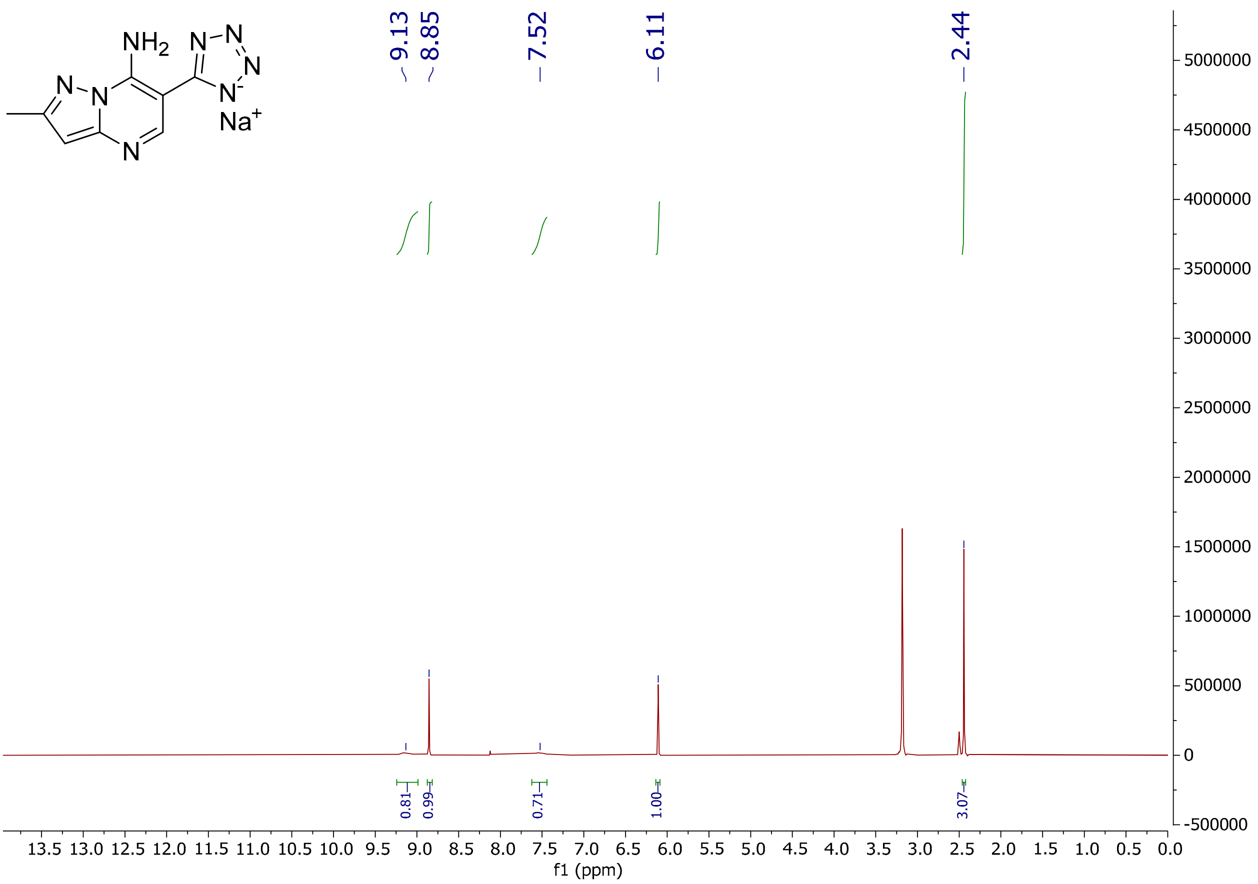

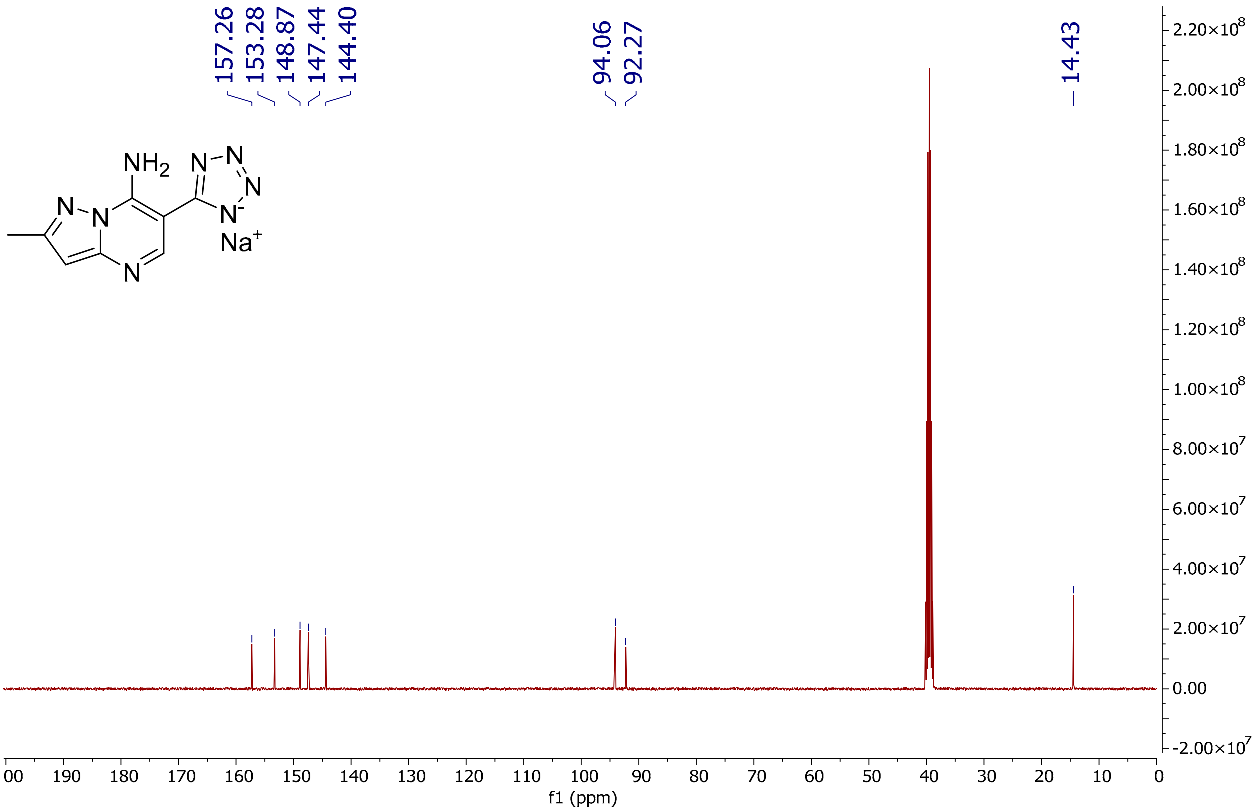

Figure S48. ^{1}H NMR (400 MHz, DMSO-d_6) and ^{13}C NMR (100 MHz, DMSO-d_6) spectra of **3b**

Sodium 5-(7-amino-2-methylpyrazolo[1,5-a]pyrimidin-6-yl)tetrazol-1-ide (3b)

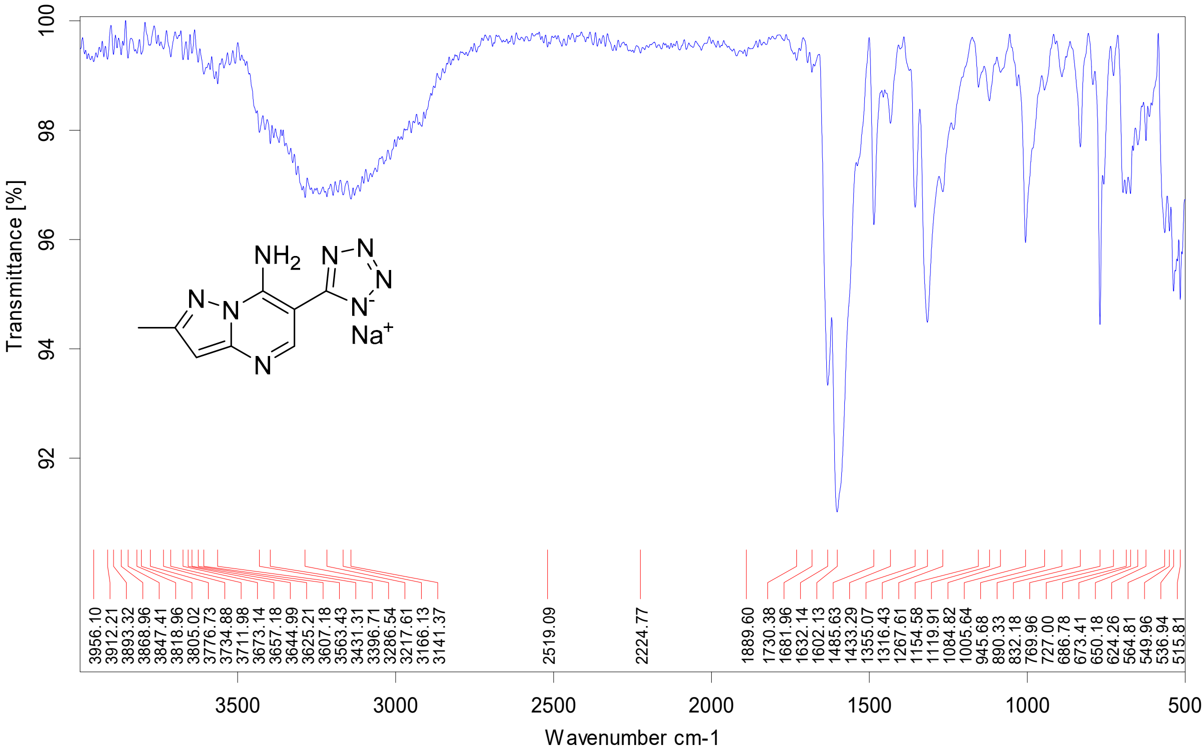

Figure S49. IR spectra of **3b**

Sodium 5-(7-amino-2-(methylthio)pyrazolo[1,5-a]pyrimidin-6-yl)tetrazol-1-ide (3c)

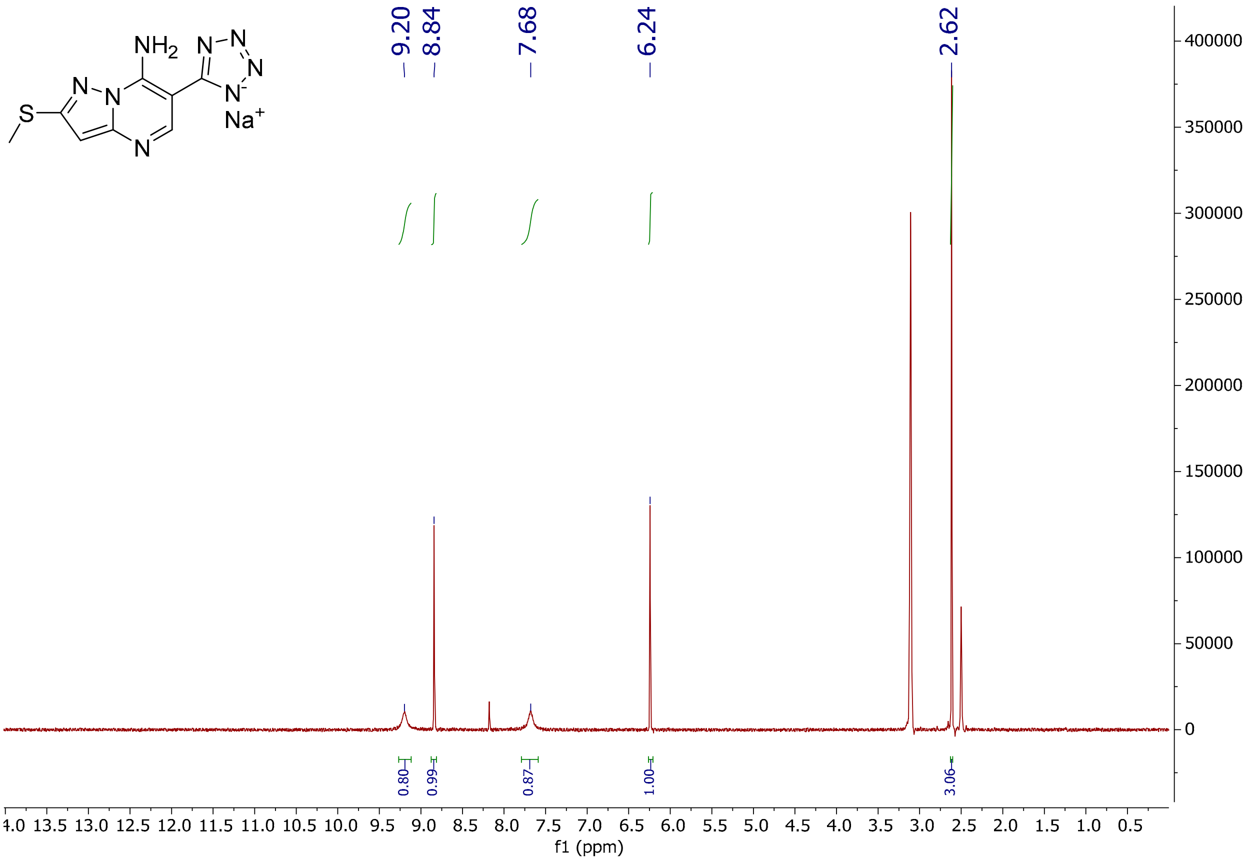

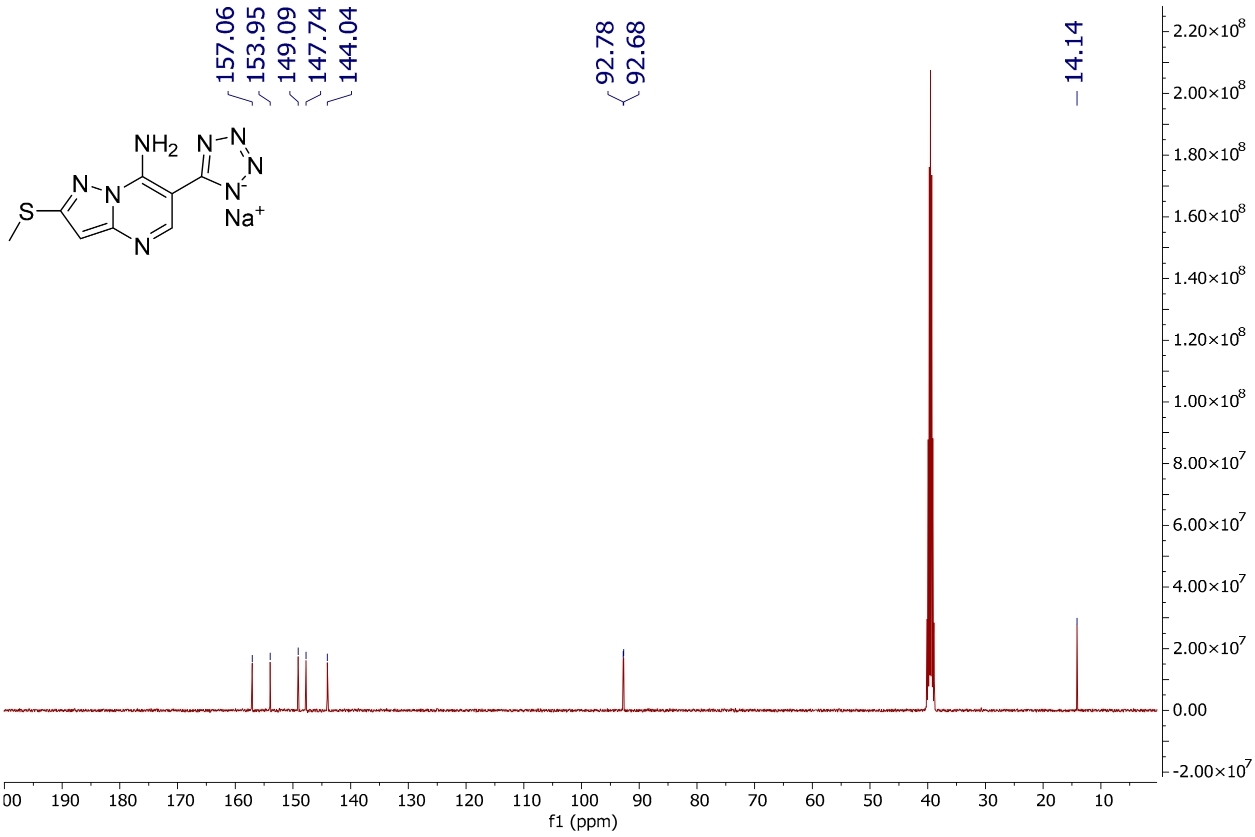

Figure S50. ^{1}H NMR (400 MHz, DMSO-d_6) and ^{13}C NMR (100 MHz, DMSO-d_6) spectra of **3c**

Sodium 5-(7-amino-2-(methylthio)pyrazolo[1,5-a]pyrimidin-6-yl)tetrazol-1-ide (3c)

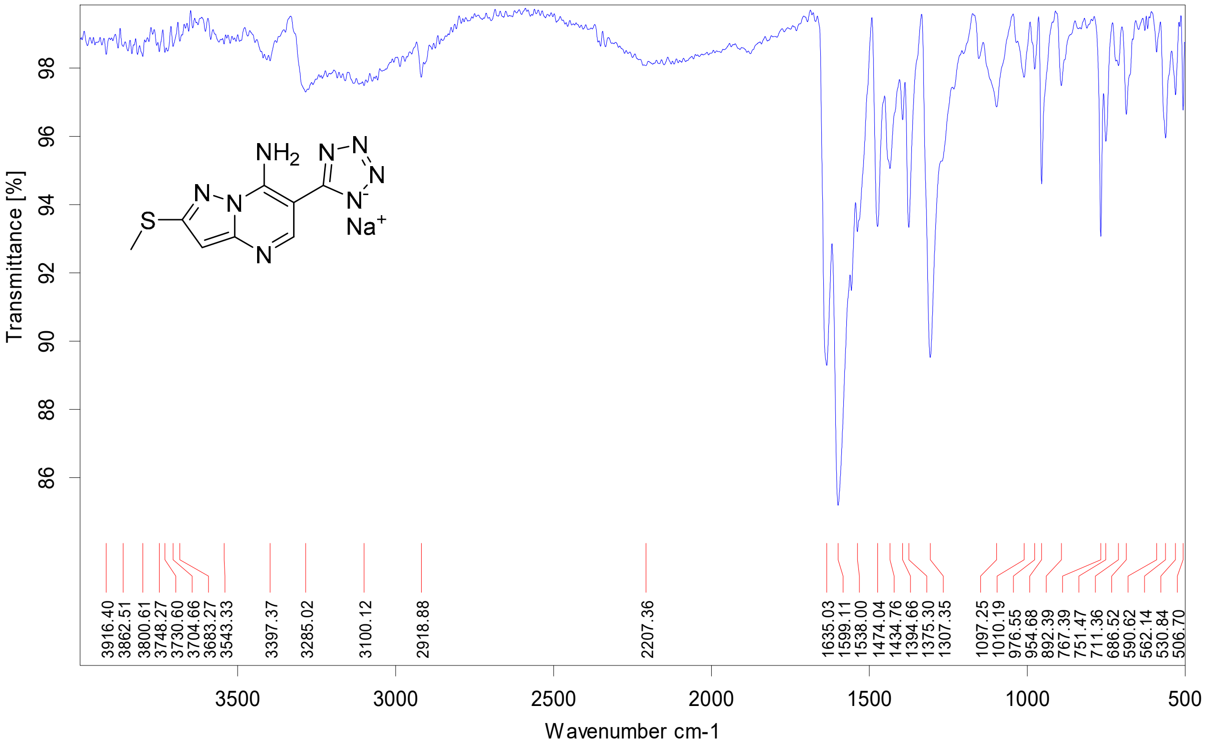

Figure S51. IR spectra of **3c**

Sodium 5-(7-amino-3-cyanopyrazolo[1,5-a]pyrimidin-6-yl)tetrazol-1-ide (3f)

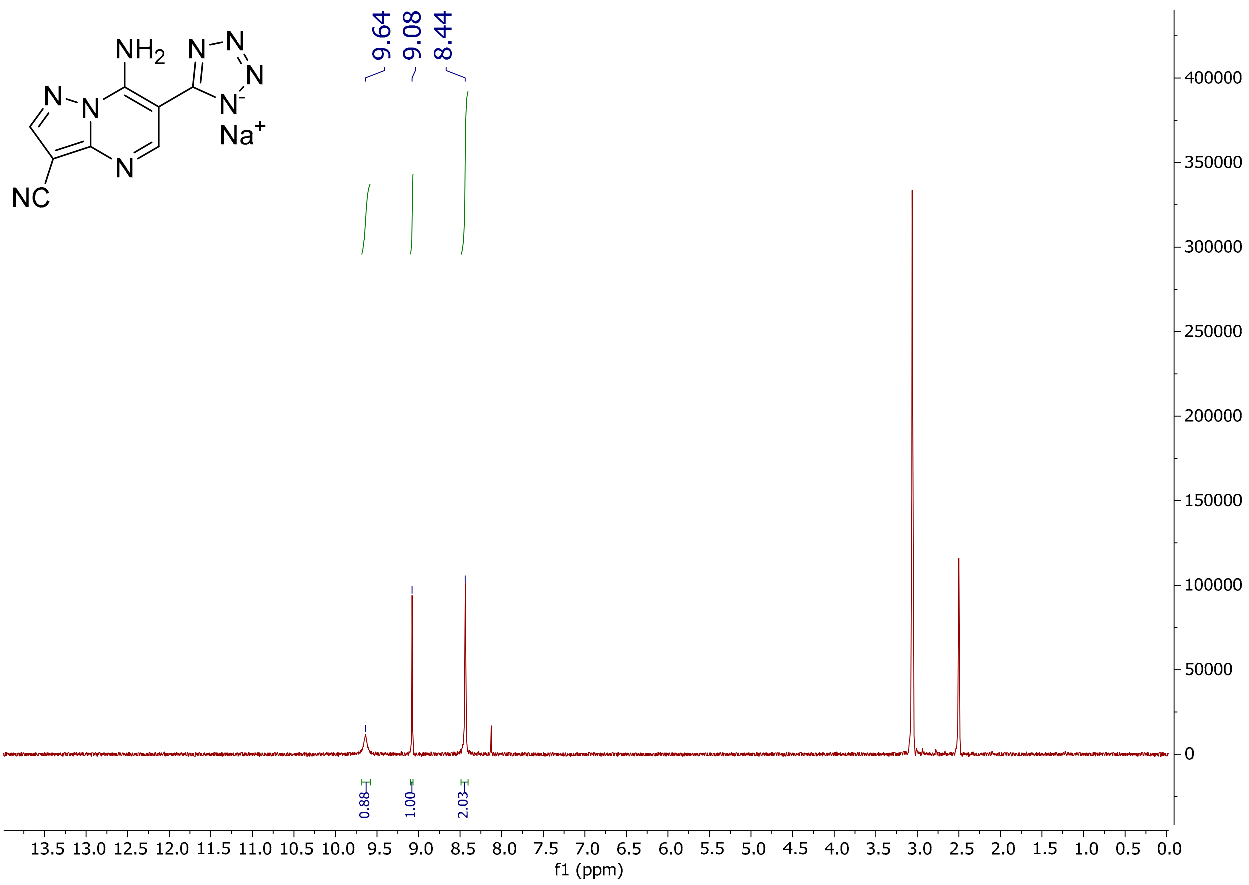

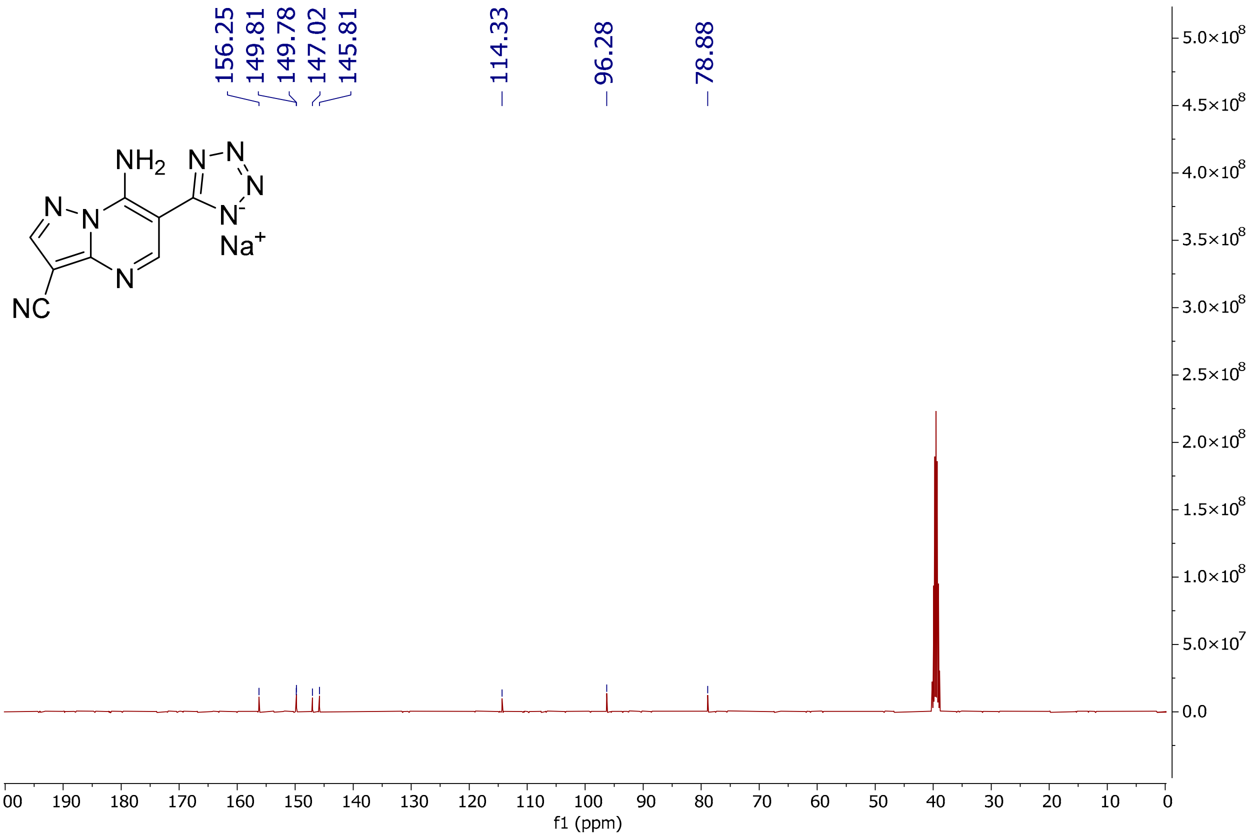

Figure S52. ^{1}H NMR (400 MHz, DMSO-d_6) and ^{13}C NMR (100 MHz, DMSO-d_6) spectra of **3f**

Sodium 5-(7-amino-3-cyanopyrazolo[1,5-a]pyrimidin-6-yl)tetrazol-1-ide (3f)

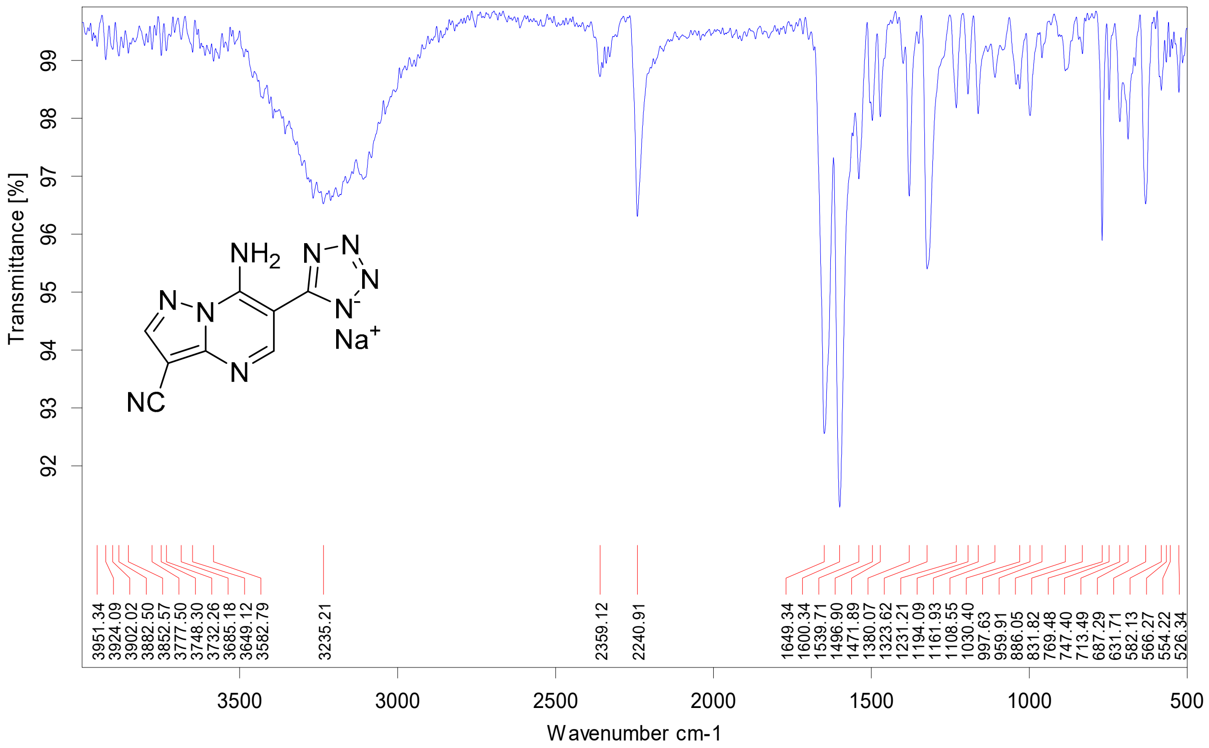

Figure S53. IR spectra of **3f**

Sodium 5-(7-amino-3-(ethoxycarbonyl)pyrazolo[1,5-a]pyrimidin-6-yl)tetrazol-1-ide (3g)

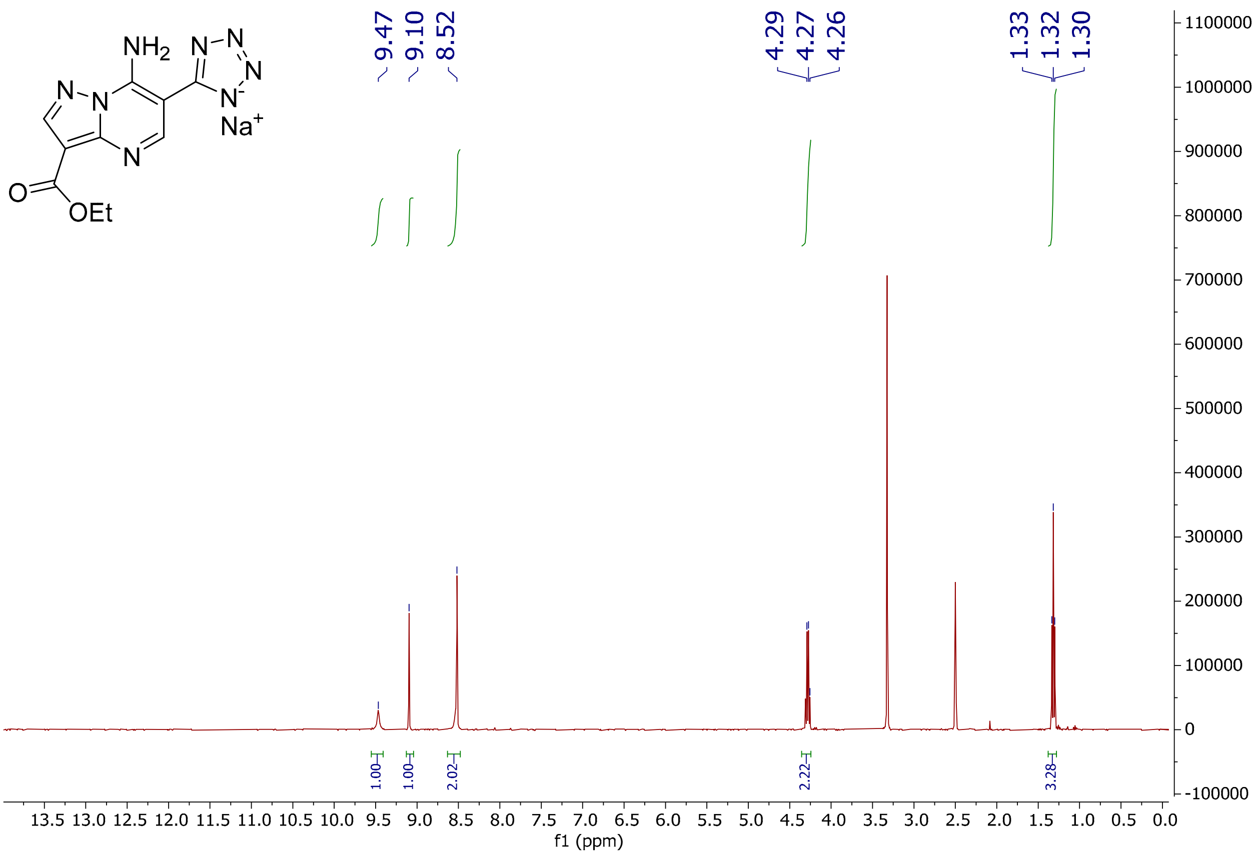

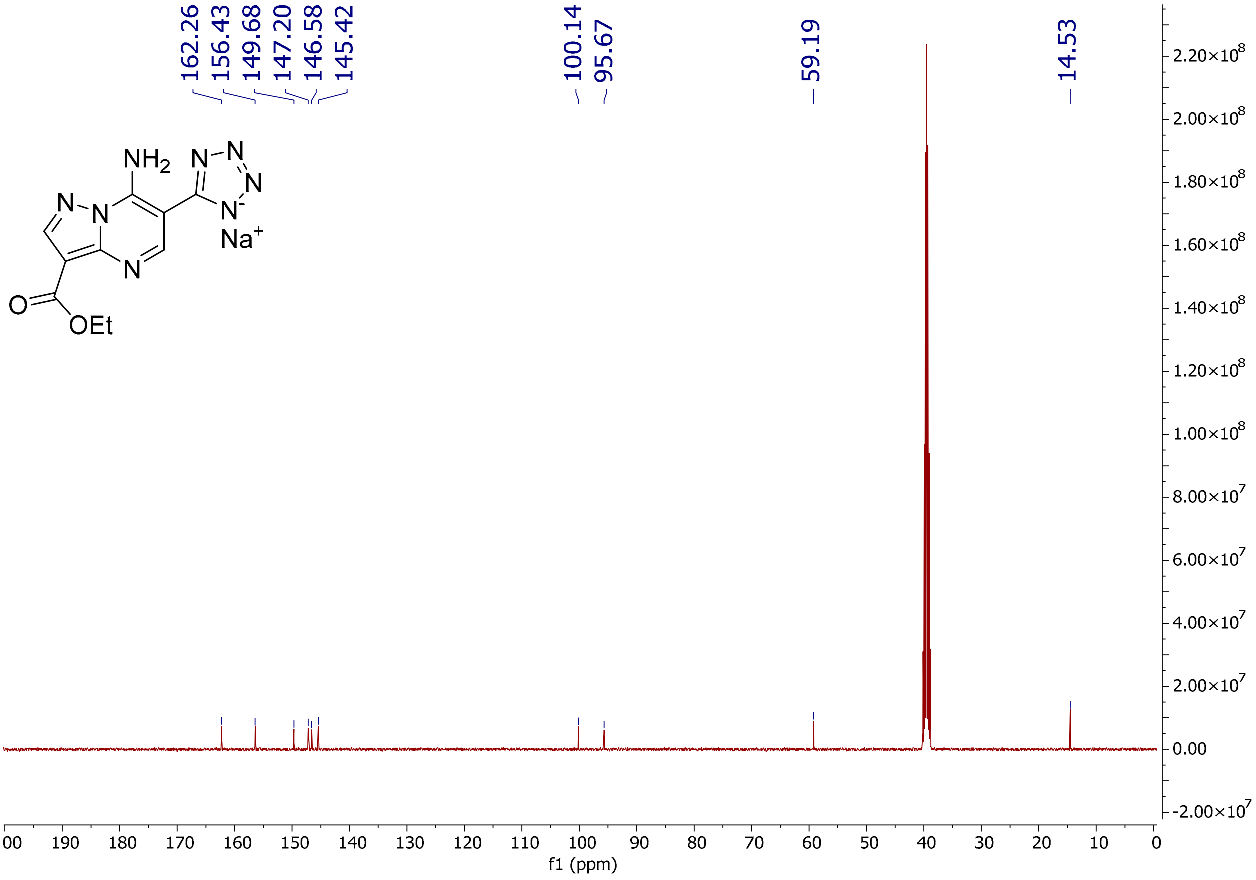

Figure S54. ^{1}H NMR (400 MHz, DMSO-d_6) and ^{13}C NMR (100 MHz, DMSO-d_6) spectra of **3g**

Sodium 5-(7-amino-3-(ethoxycarbonyl)pyrazolo[1,5-a]pyrimidin-6-yl)tetrazol-1-ide (3g)

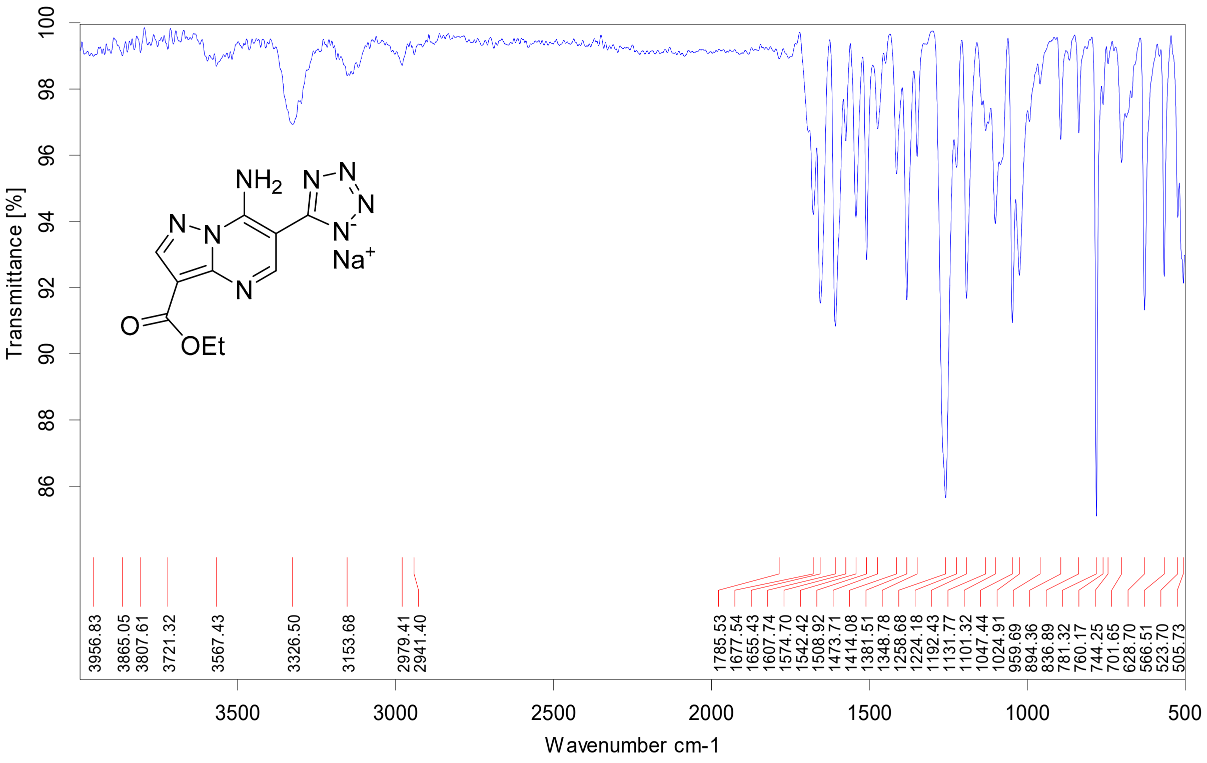

Figure S55. IR spectra of **3g**

Sodium 5-(7-amino-3-nitropyrazolo[1,5-a]pyrimidin-6-yl)tetrazol-1-ide (3h)

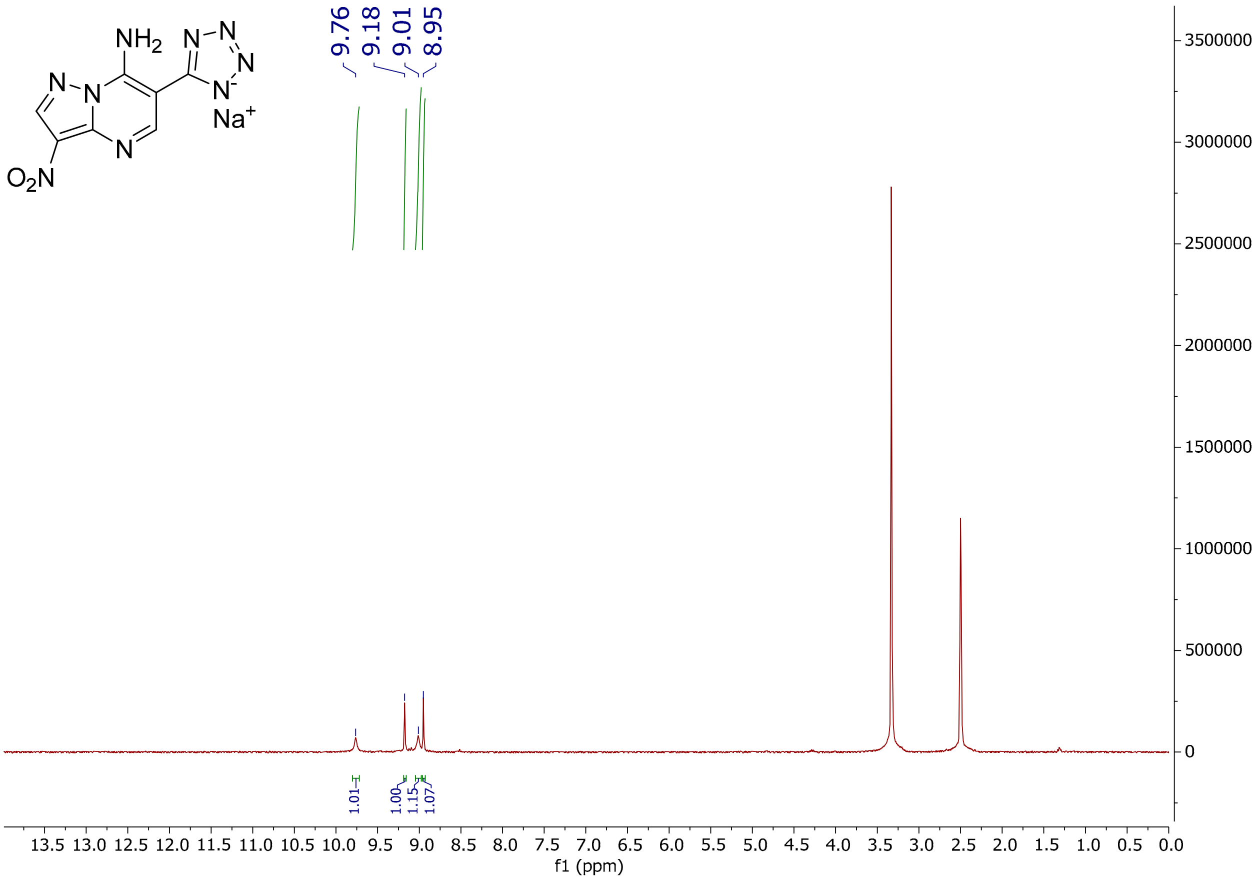

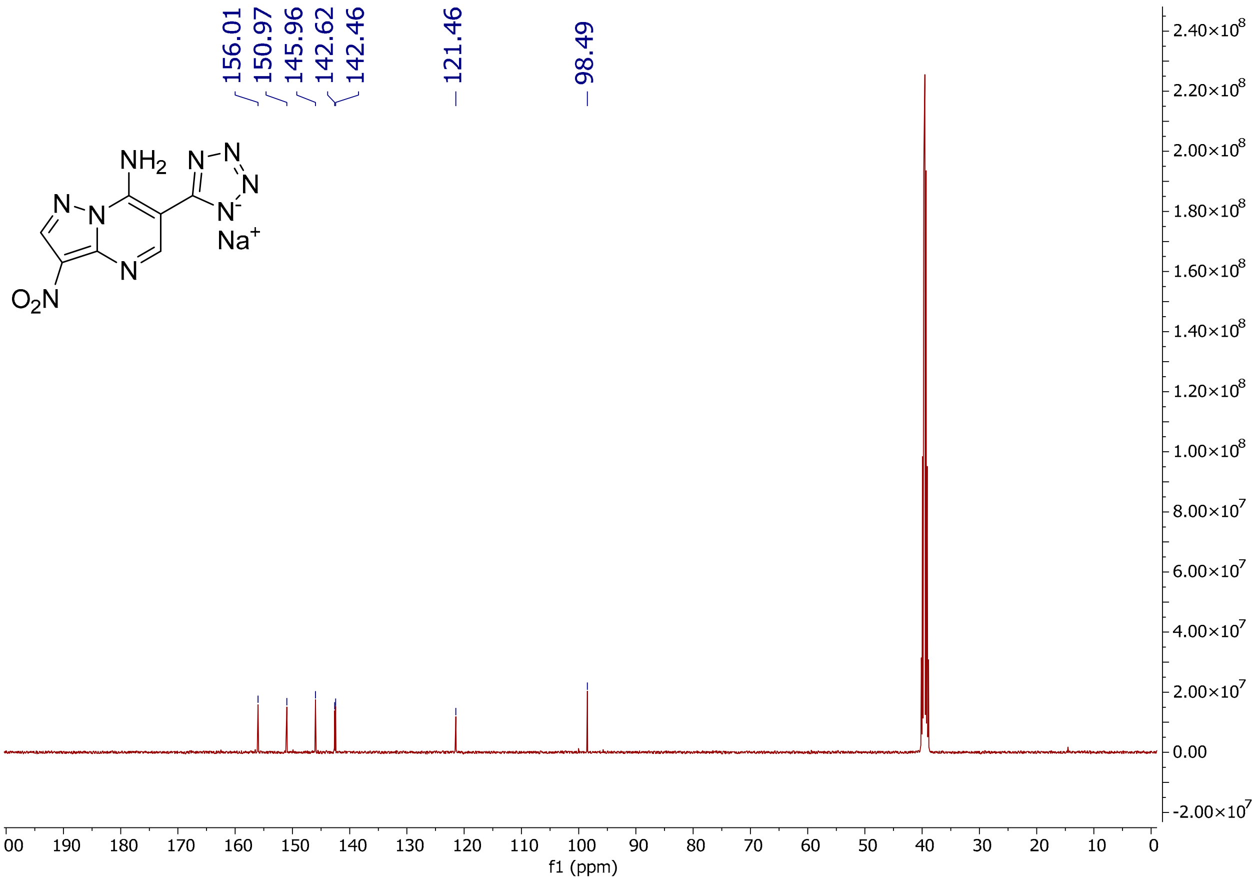

Figure S56. ^{1}H NMR (400 MHz, DMSO-d_6) and ^{13}C NMR (100 MHz, DMSO-d_6) spectra of **3h**

Sodium 5-(7-amino-3-nitropyrazolo[1,5-a]pyrimidin-6-yl)tetrazol-1-ide (3h)

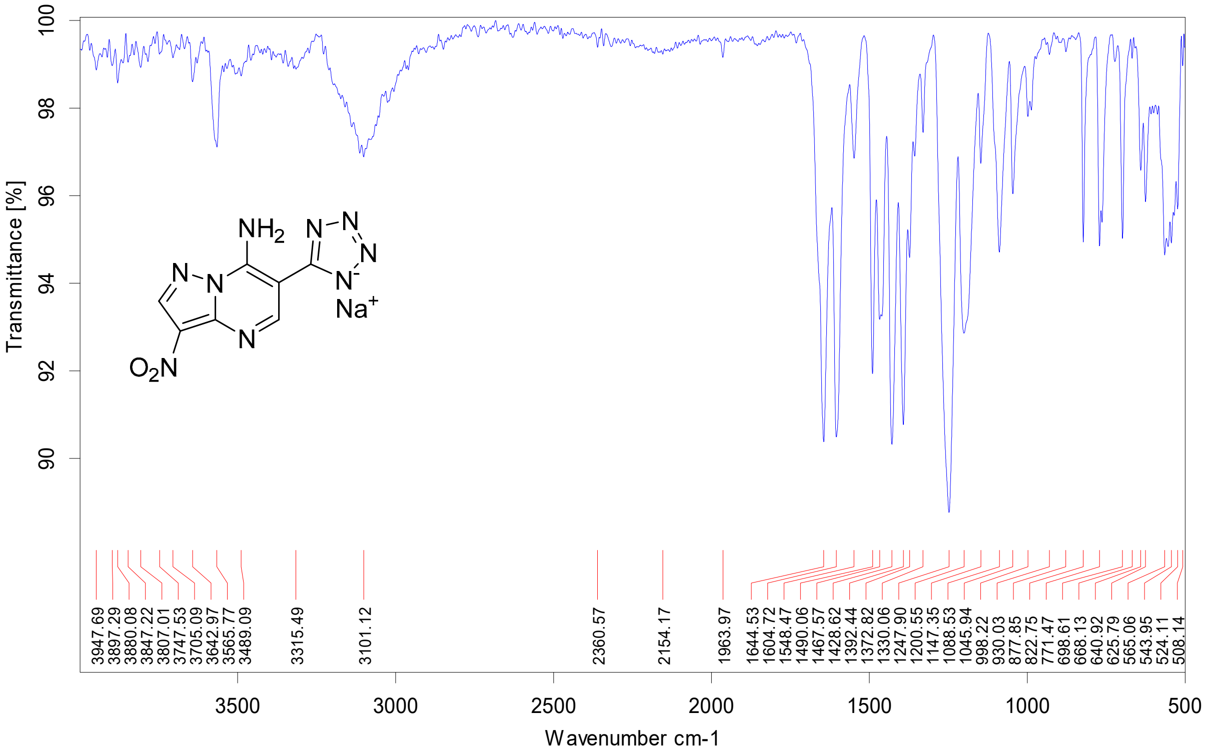

Figure S57. IR spectra of **3h**

Sodium 5-(7-amino-3-phenylpyrazolo[1,5-a]pyrimidin-6-yl)tetrazol-1-ide (3i)

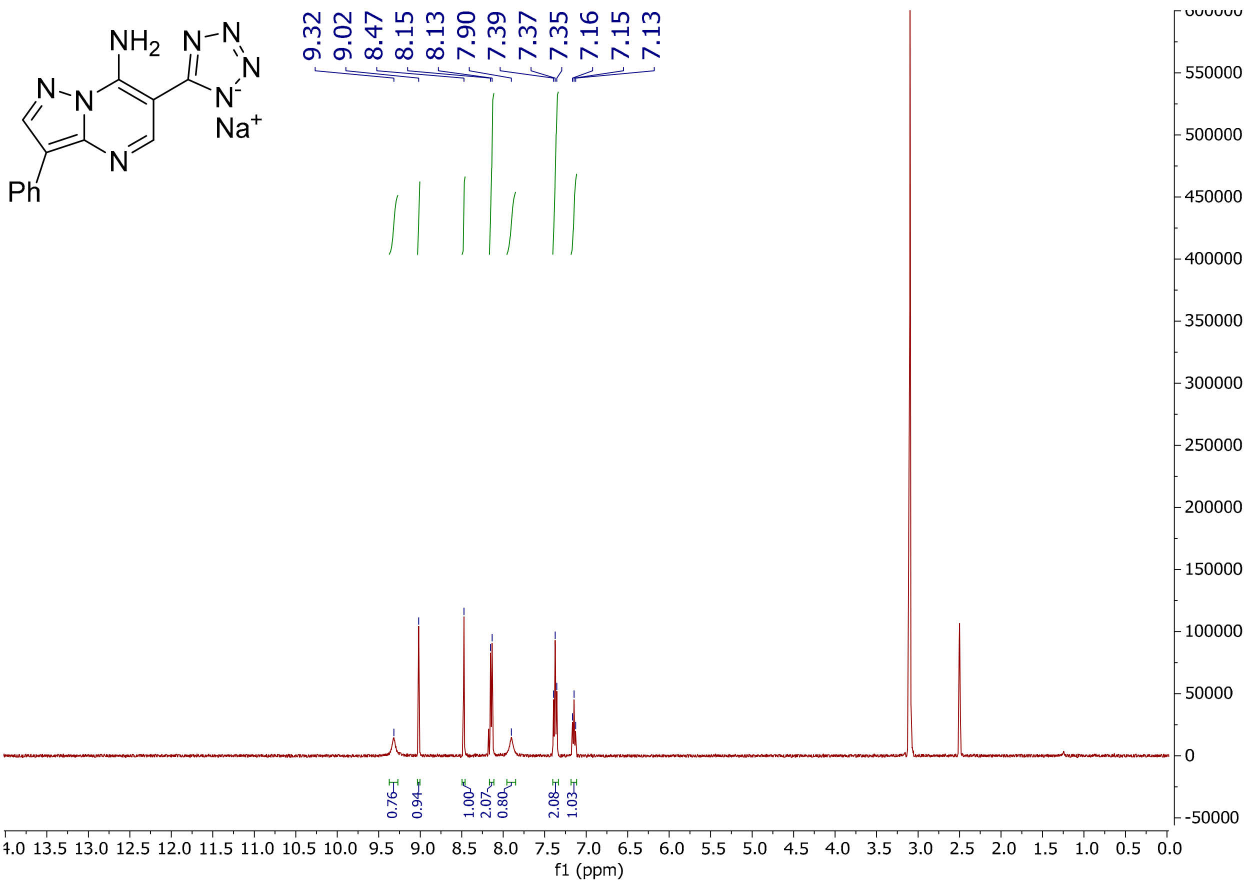

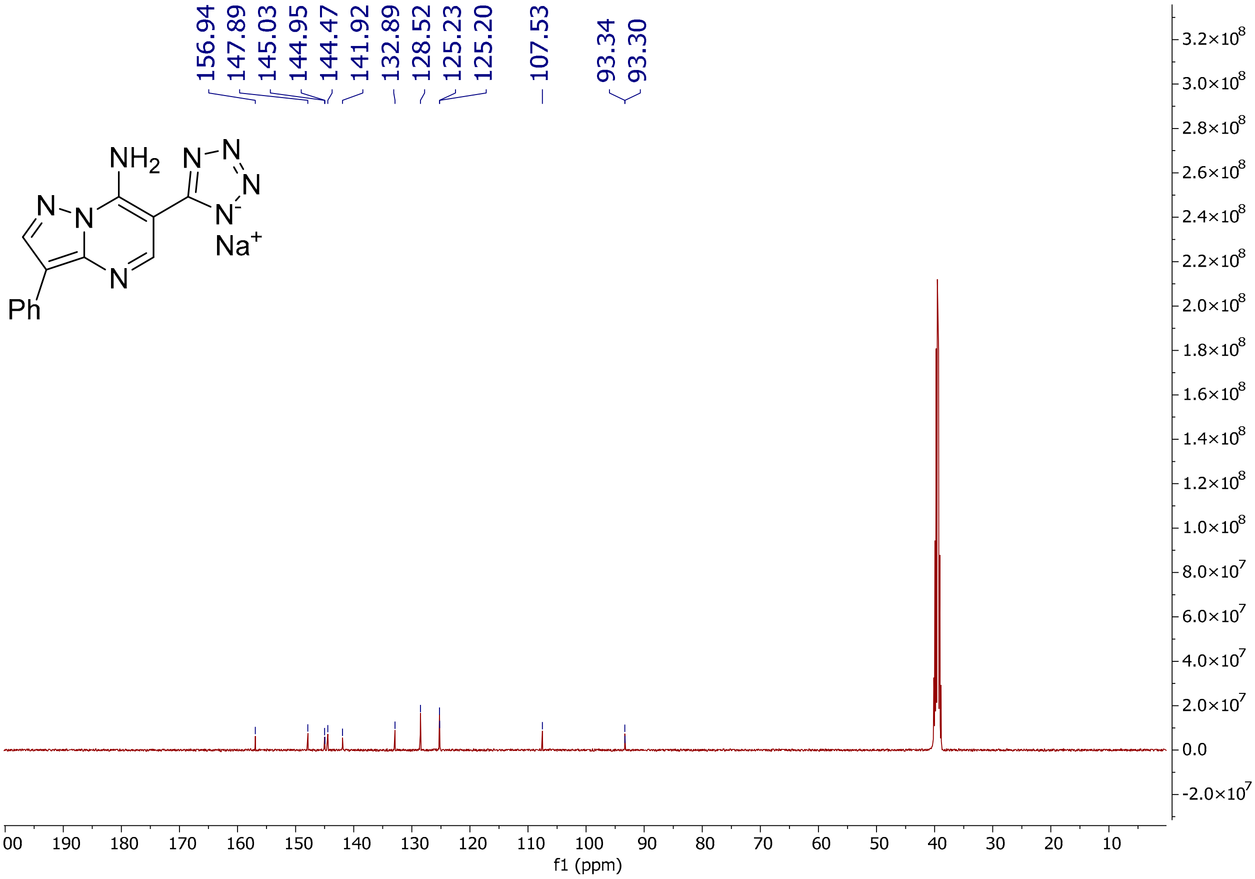

Figure S58. ^{1}H NMR (400 MHz, DMSO-d_6) and ^{13}C NMR (100 MHz, DMSO-d_6) spectra of **3i**

Sodium 5-(7-amino-3-phenylpyrazolo[1,5-a]pyrimidin-6-yl)tetrazol-1-ide (3i)

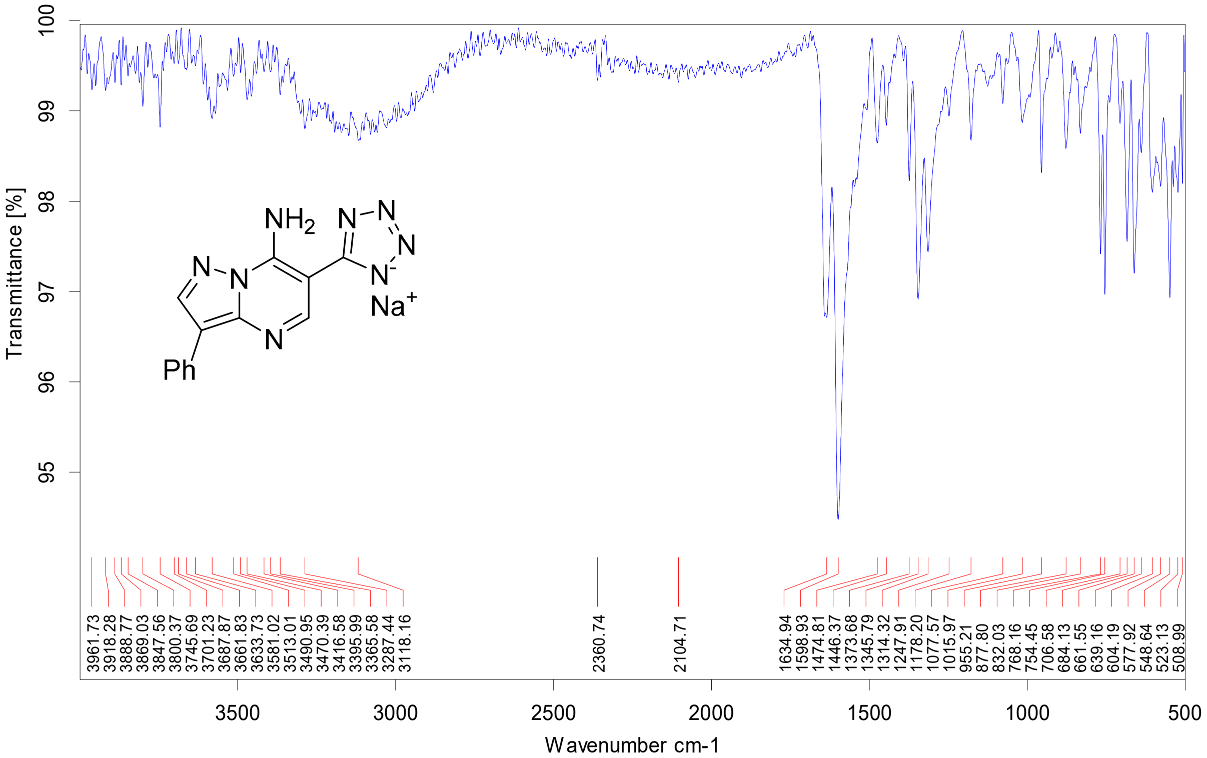

Figure S59. IR spectra of **3i**

Sodium 5-(7-amino-3-(ethoxycarbonyl)-2-(methylthio)pyrazolo[1,5-a]pyrimidin-6-yl)tetrazol-1-ide (3j)

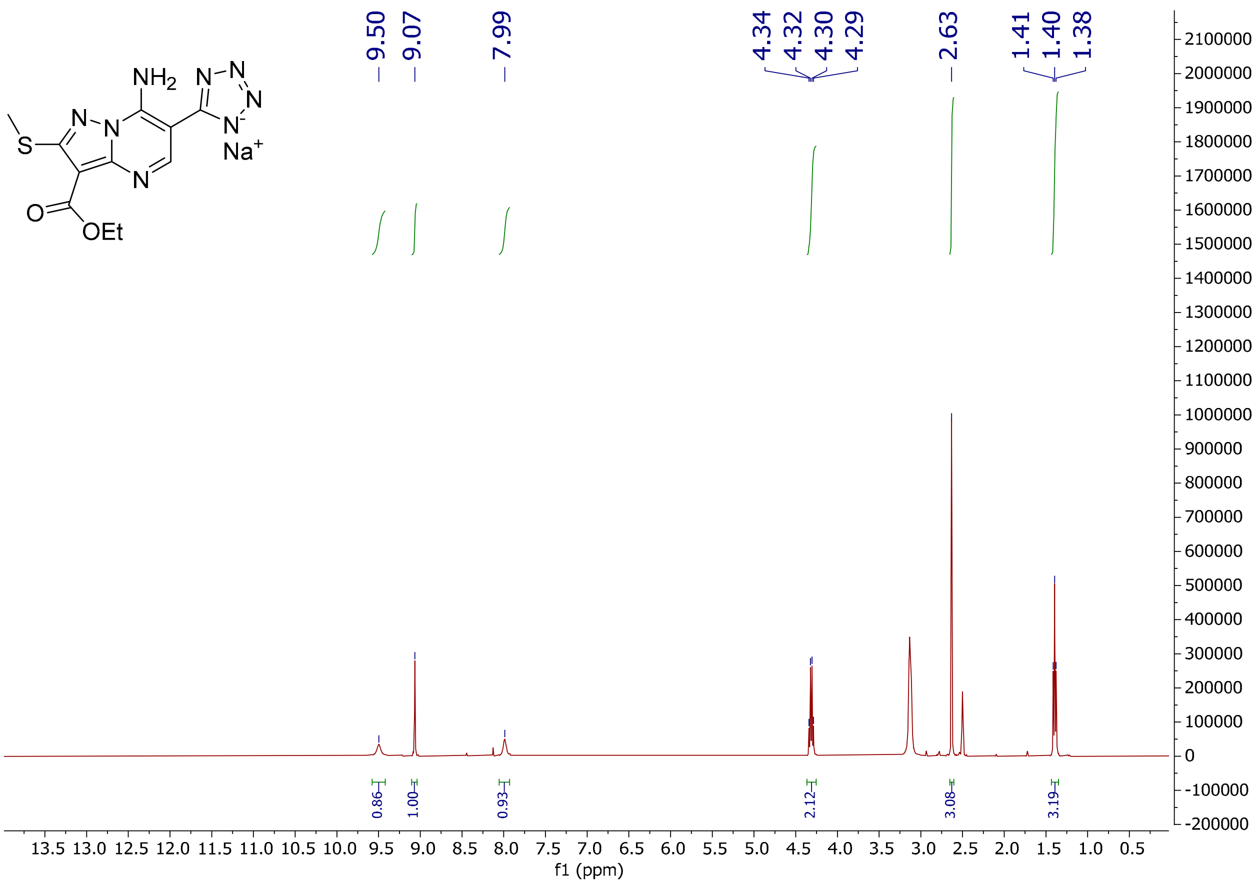

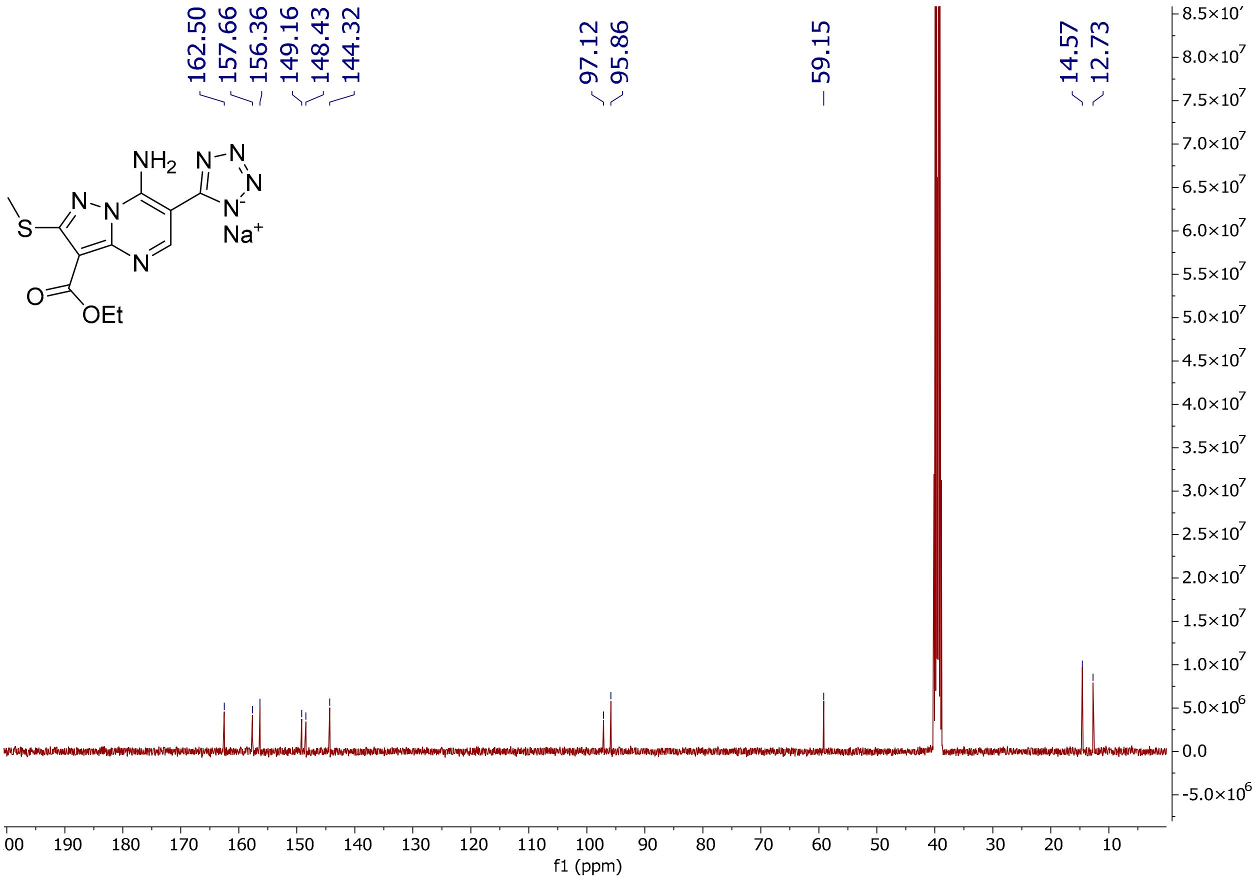

Figure S60. ^{1}H NMR (400 MHz, DMSO-d_6) and ^{13}C NMR (100 MHz, DMSO-d_6) spectra of **3j**

Sodium 5-(7-amino-3-(ethoxycarbonyl)-2-(methylthio)pyrazolo[1,5-a]pyrimidin-6-yl)tetrazol-1-ide (3j)

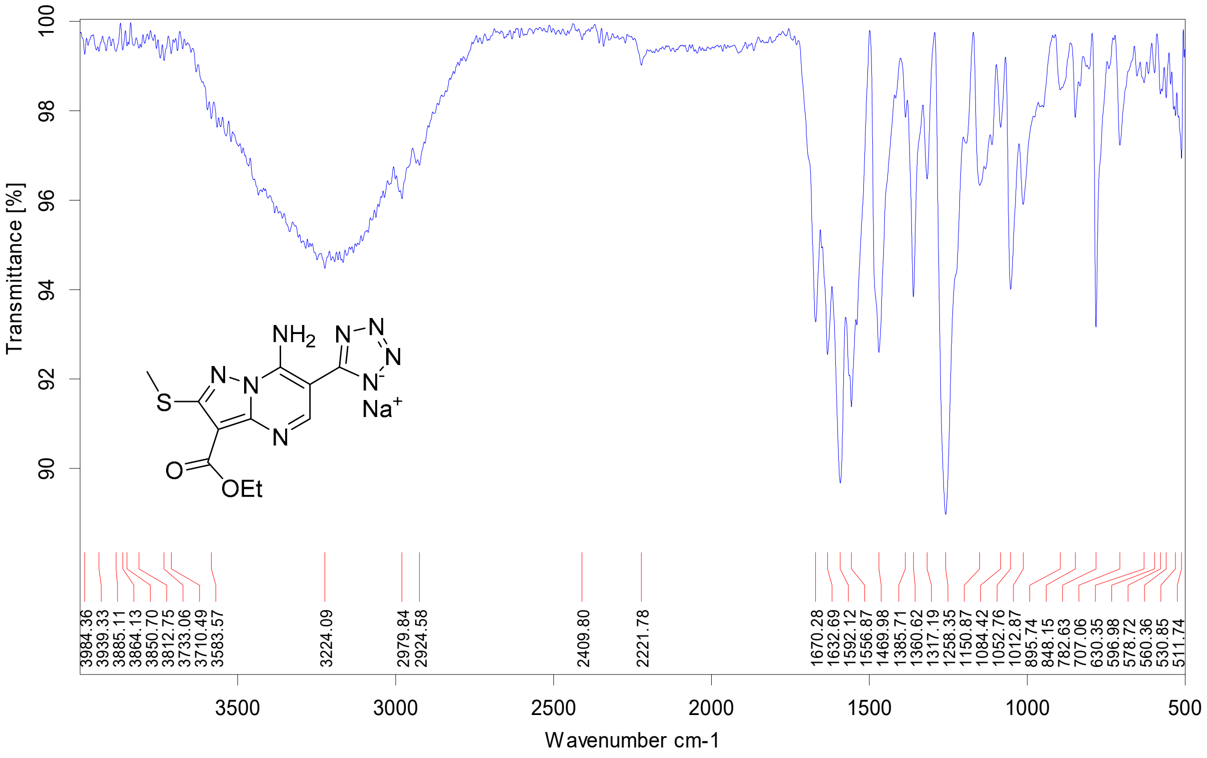

Figure S61. IR spectra of **3j**

Sodium 5-(7-amino-3-cyano-2-(methylthio)pyrazolo[1,5-a]pyrimidin-6-yl)tetrazol-1-ide 1 (3k)

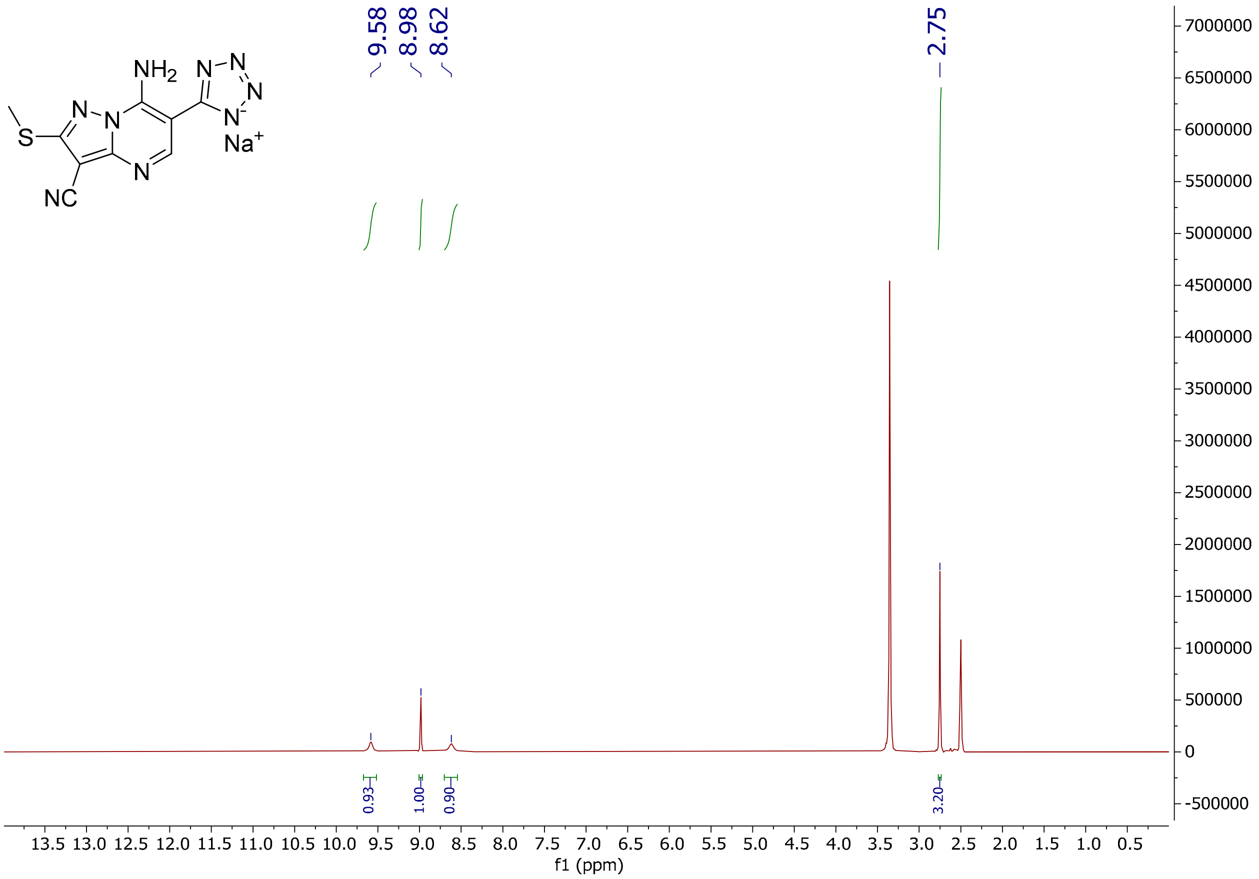

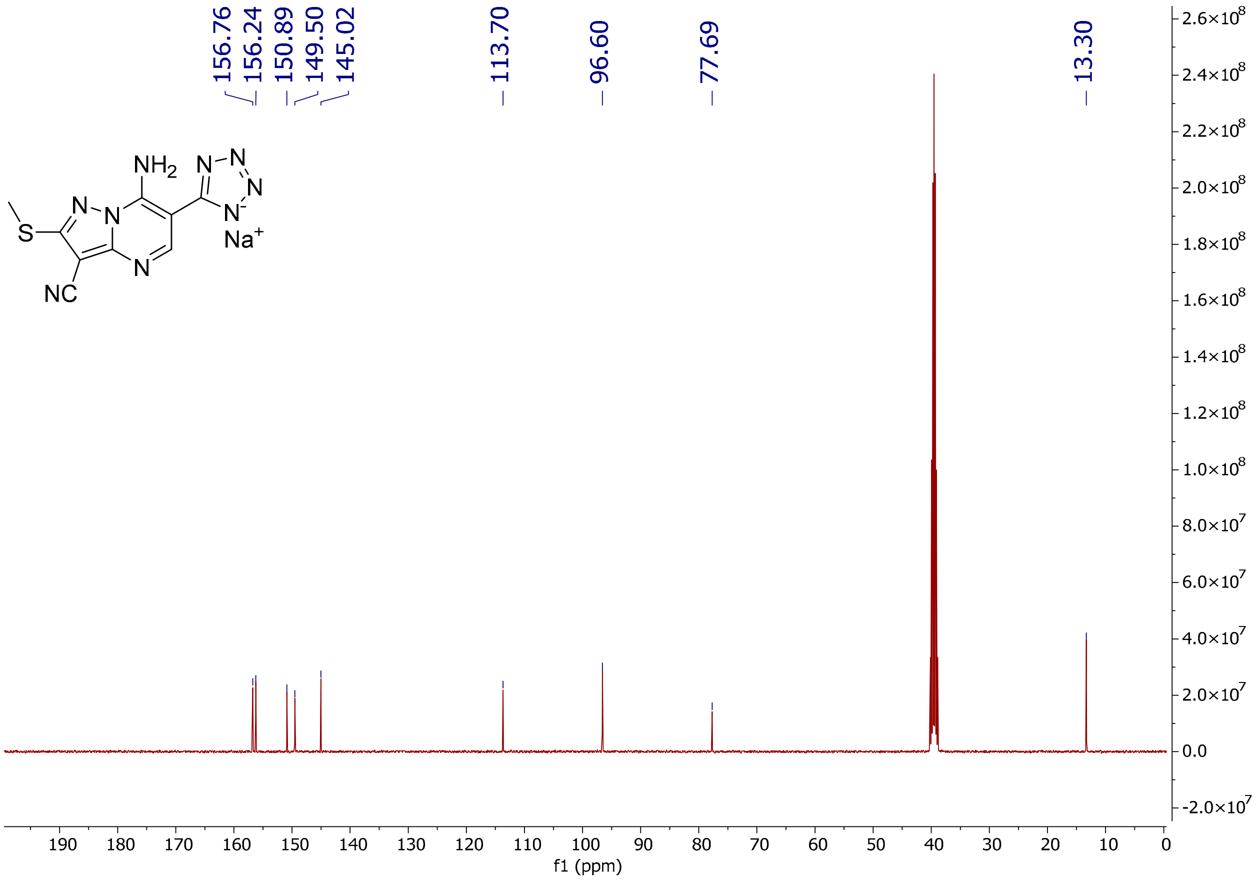

Figure S62. ^{1}H NMR (400 MHz, DMSO-d_6) and ^{13}C NMR (100 MHz, DMSO-d_6) spectra of **3k**

Sodium 5-(7-amino-3-cyano-2-(methylthio)pyrazolo[1,5-a]pyrimidin-6-yl)tetrazol-1-ide 1 (3k)

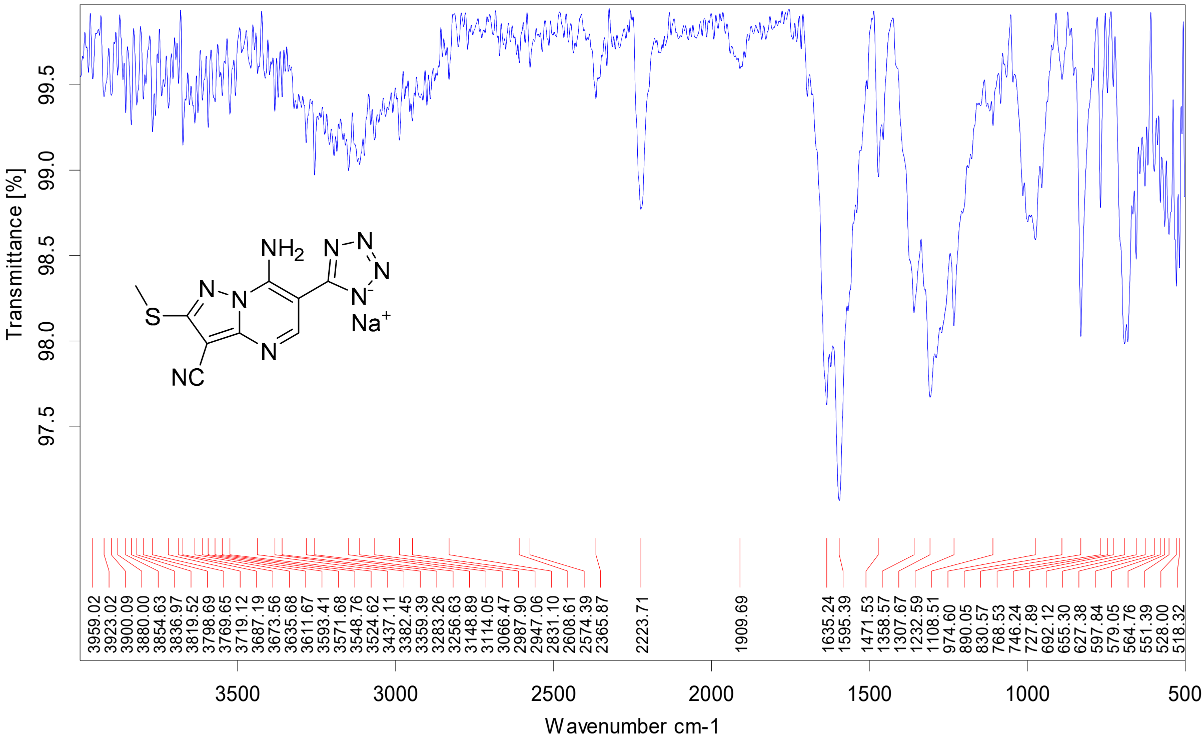

Figure S63. IR spectra of **3k**

Sodium 5-(7-amino-[1,2,4]triazolo[1,5-a]pyrimidin-6-yl)tetrazol-1-ide (11a)

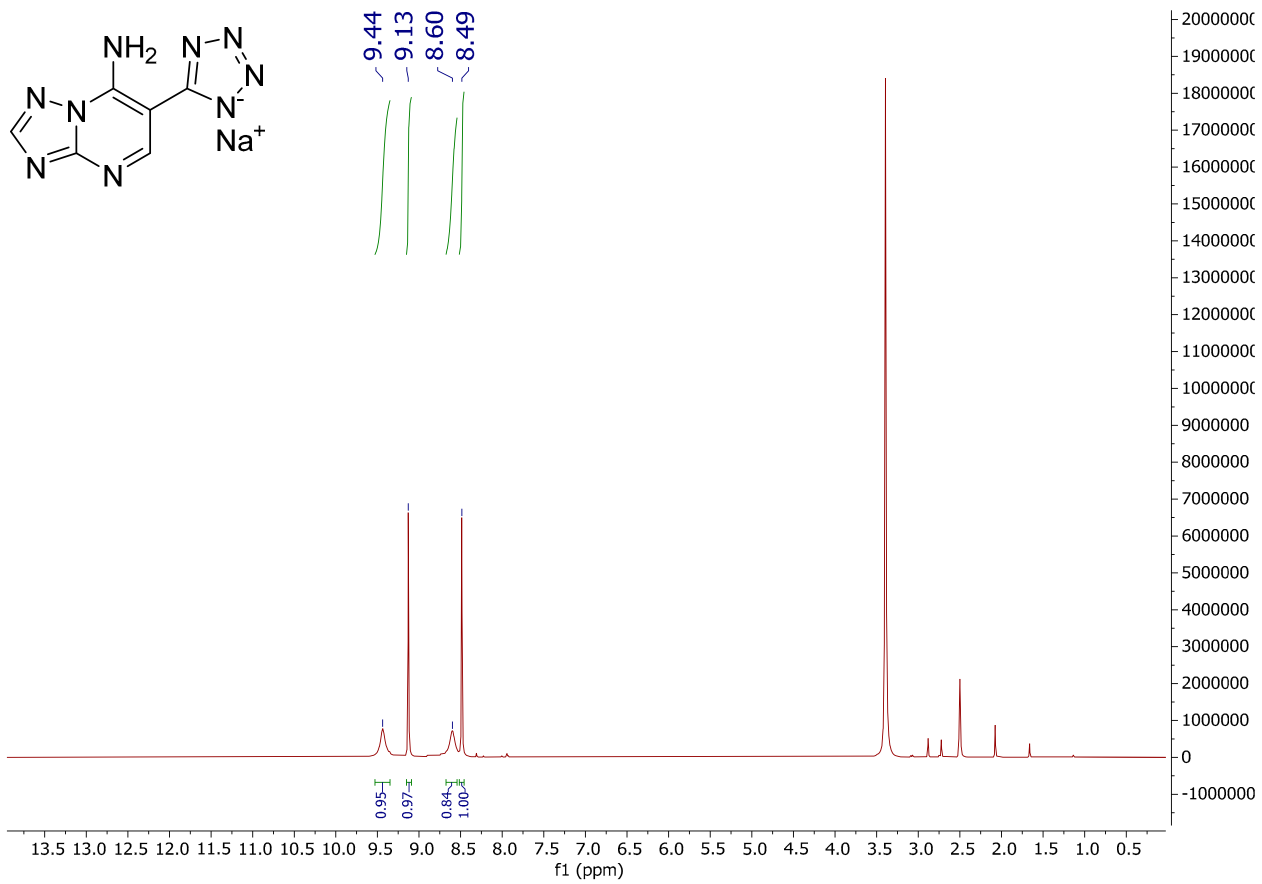

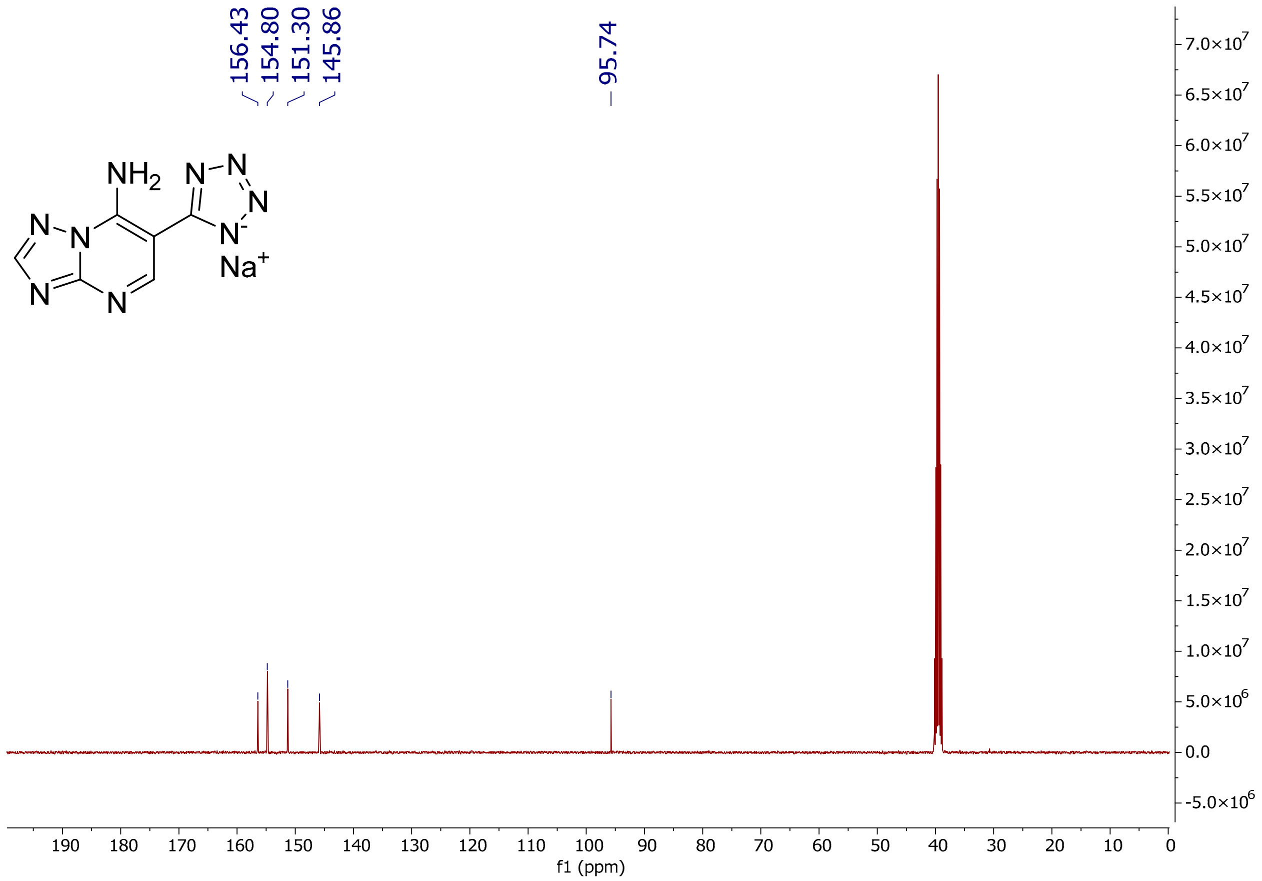

Figure S64. ^{1}H NMR (400 MHz, DMSO-d_6) and ^{13}C NMR (100 MHz, DMSO-d_6) spectra of **11a**

Sodium 5-(7-amino-[1,2,4]triazolo[1,5-a]pyrimidin-6-yl)tetrazol-1-ide (11a)

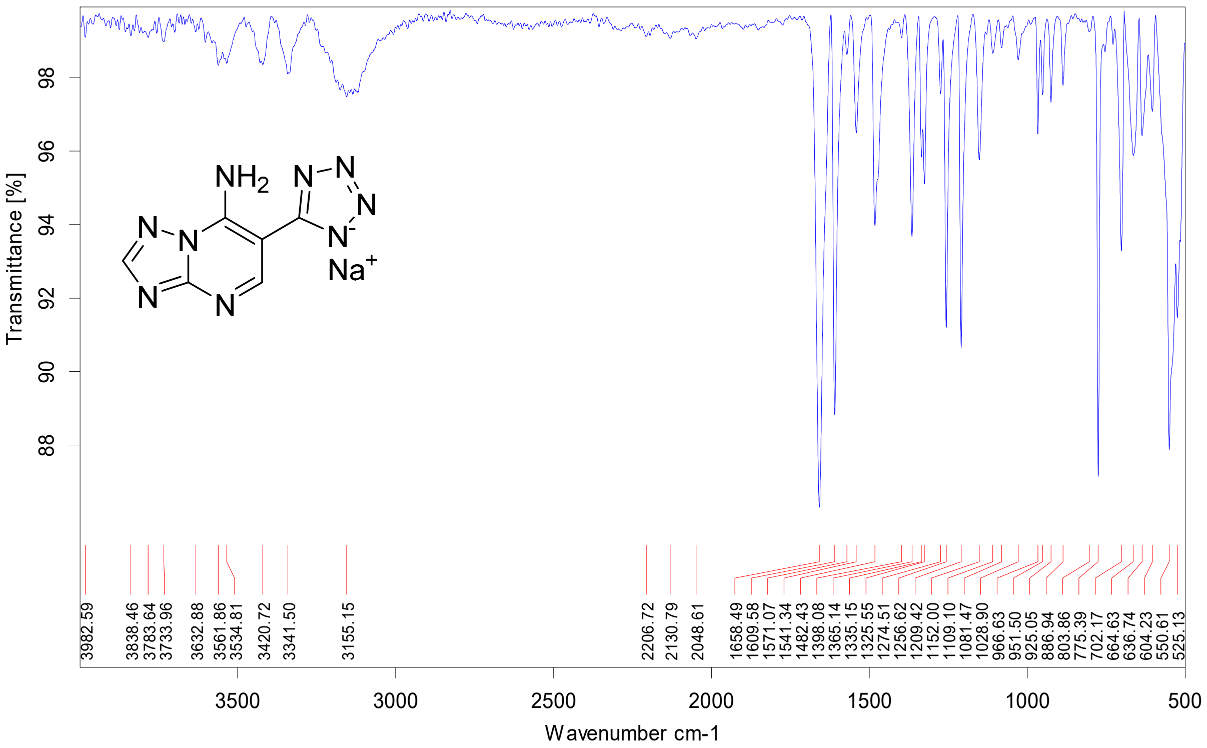

Figure S65. IR spectra of **11a**

Sodium 5-(7-amino-2-methyl-[1,2,4]triazolo[1,5-a]pyrimidin-6-yl)tetrazol-1-ide (11b)

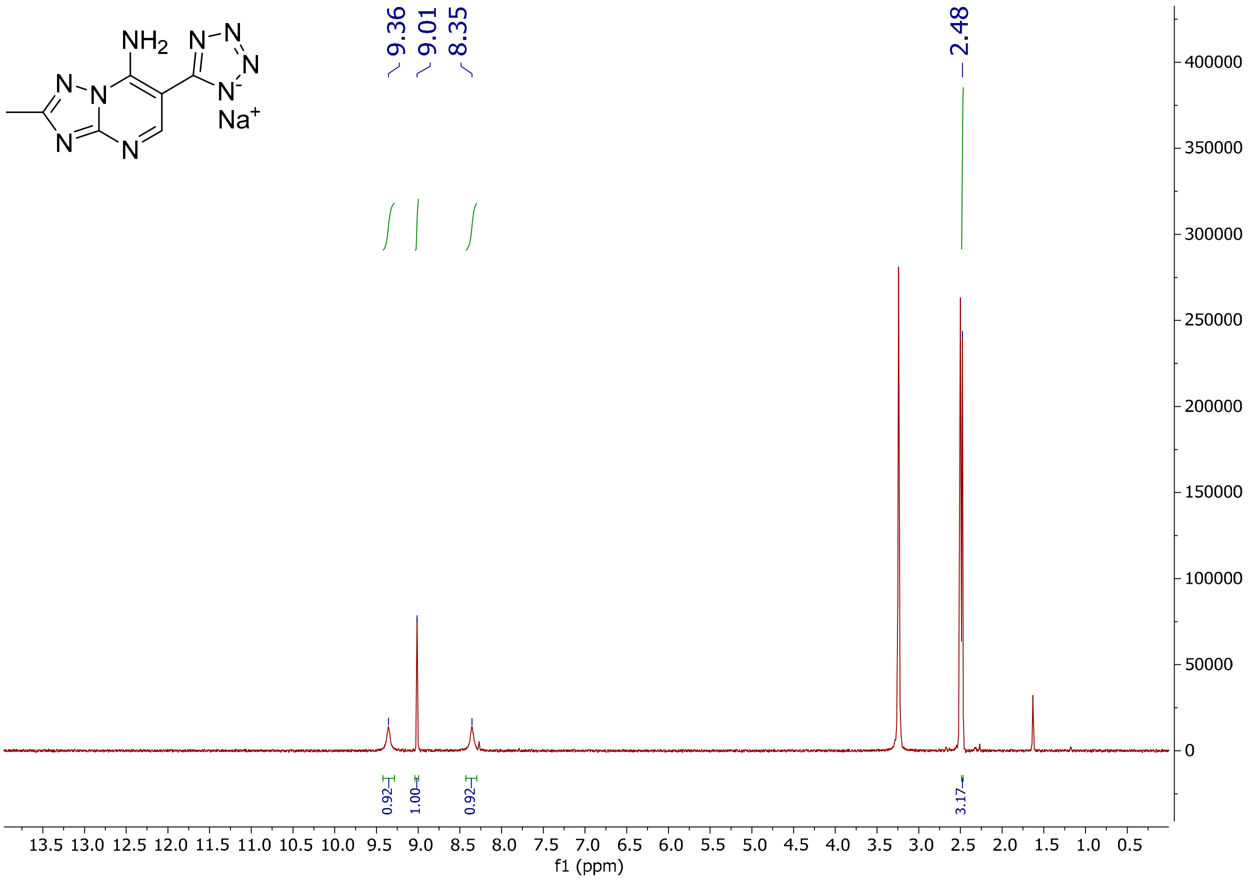

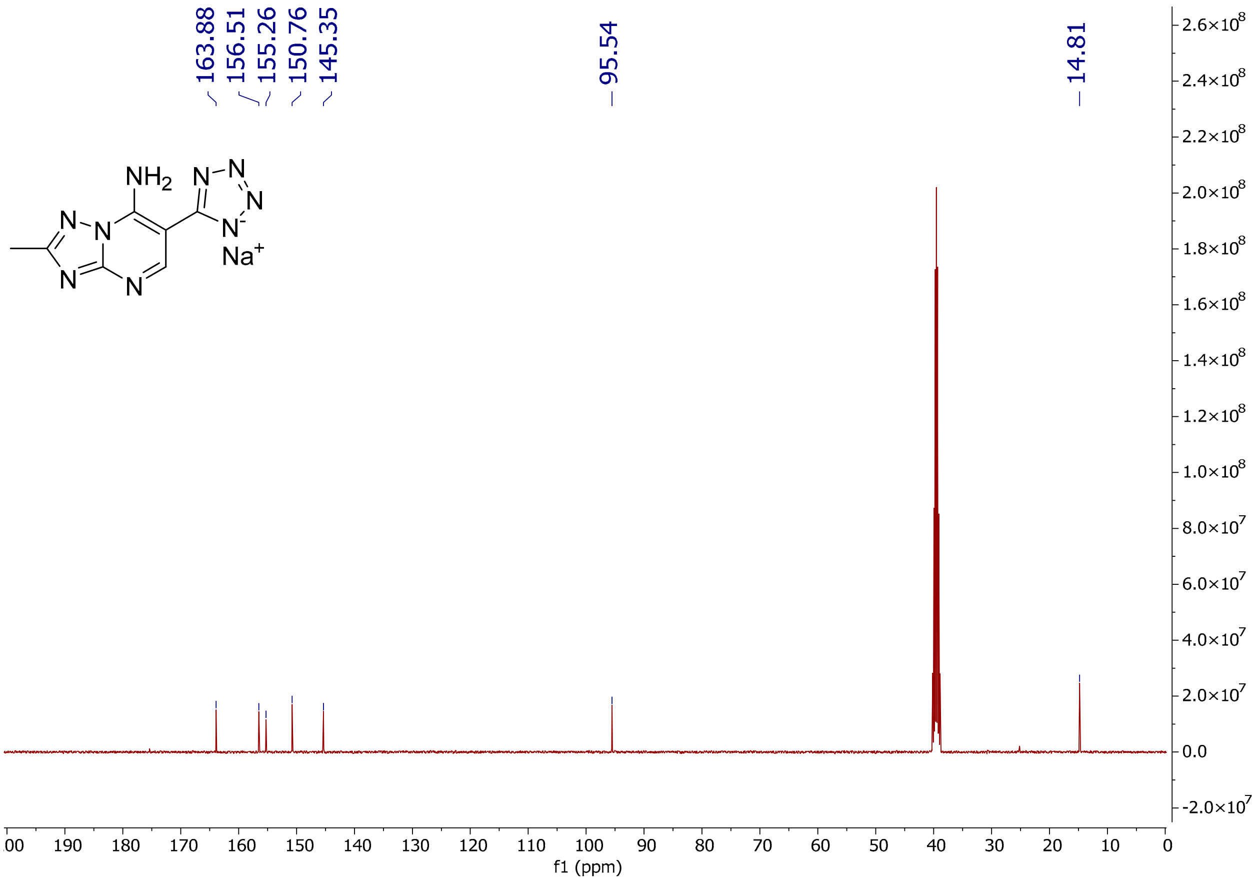

Figure S66. ^{1}H NMR (400 MHz, DMSO-d_6) and ^{13}C NMR (100 MHz, DMSO-d_6) spectra of **11b**

Sodium 5-(7-amino-2-methyl-[1,2,4]triazolo[1,5-a]pyrimidin-6-yl)tetrazol-1-ide (11b)

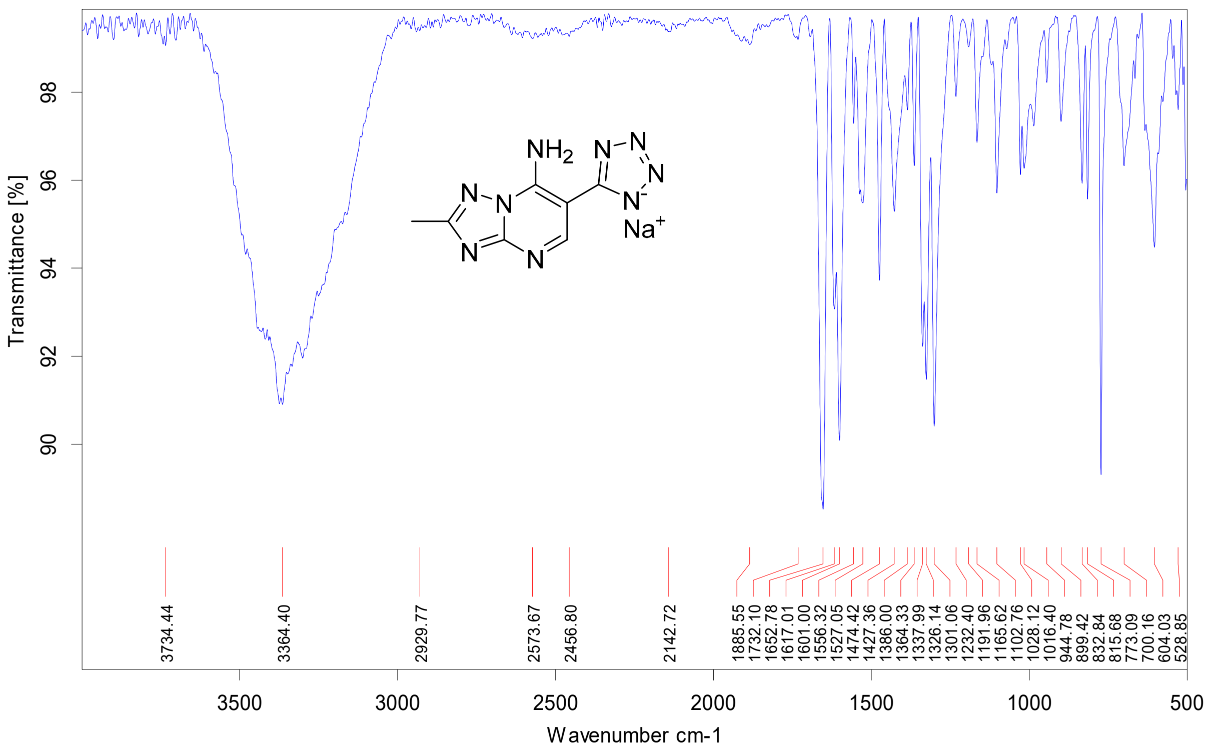

Figure S67. IR spectra of **11b**

Sodium 5-(7-amino-2-(methylthio)-[1,2,4]triazolo[1,5-a]pyrimidin-6-yl)tetrazol-1-ide (11c)

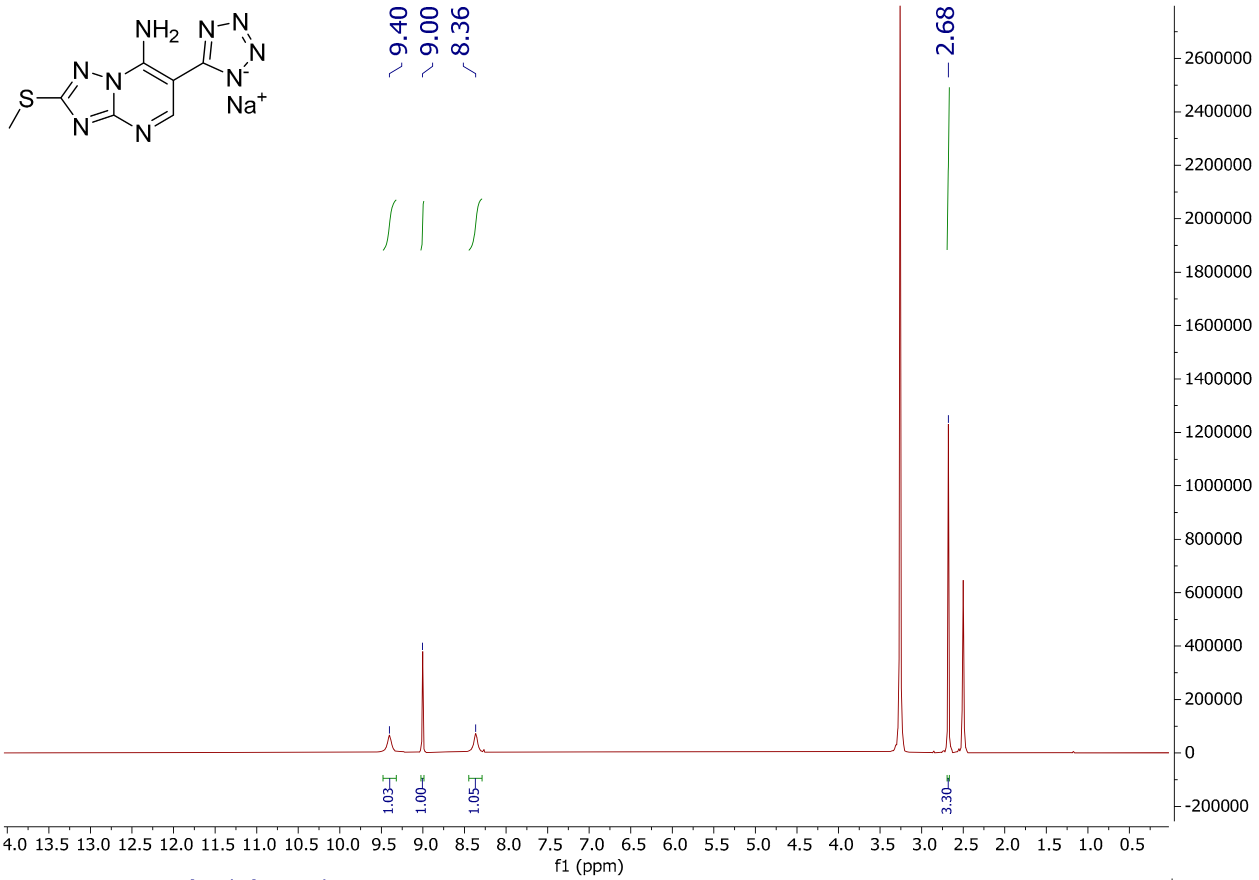

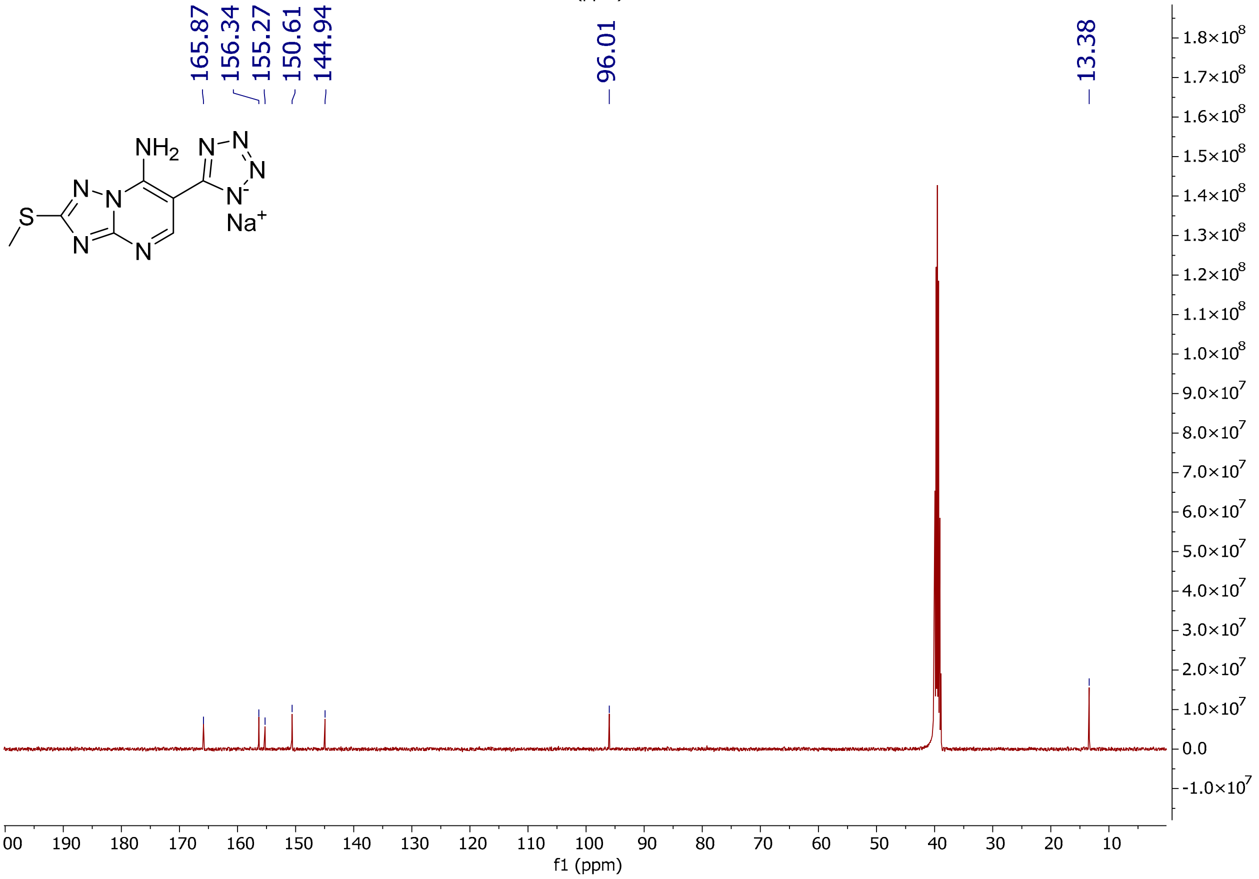

Figure S68. ^{1}H NMR (400 MHz, DMSO-d_6) and ^{13}C NMR (100 MHz, DMSO-d_6) spectra of **11c**

Sodium 5-(7-amino-2-(methylthio)-[1,2,4]triazolo[1,5-a]pyrimidin-6-yl)tetrazol-1-ide (11c)

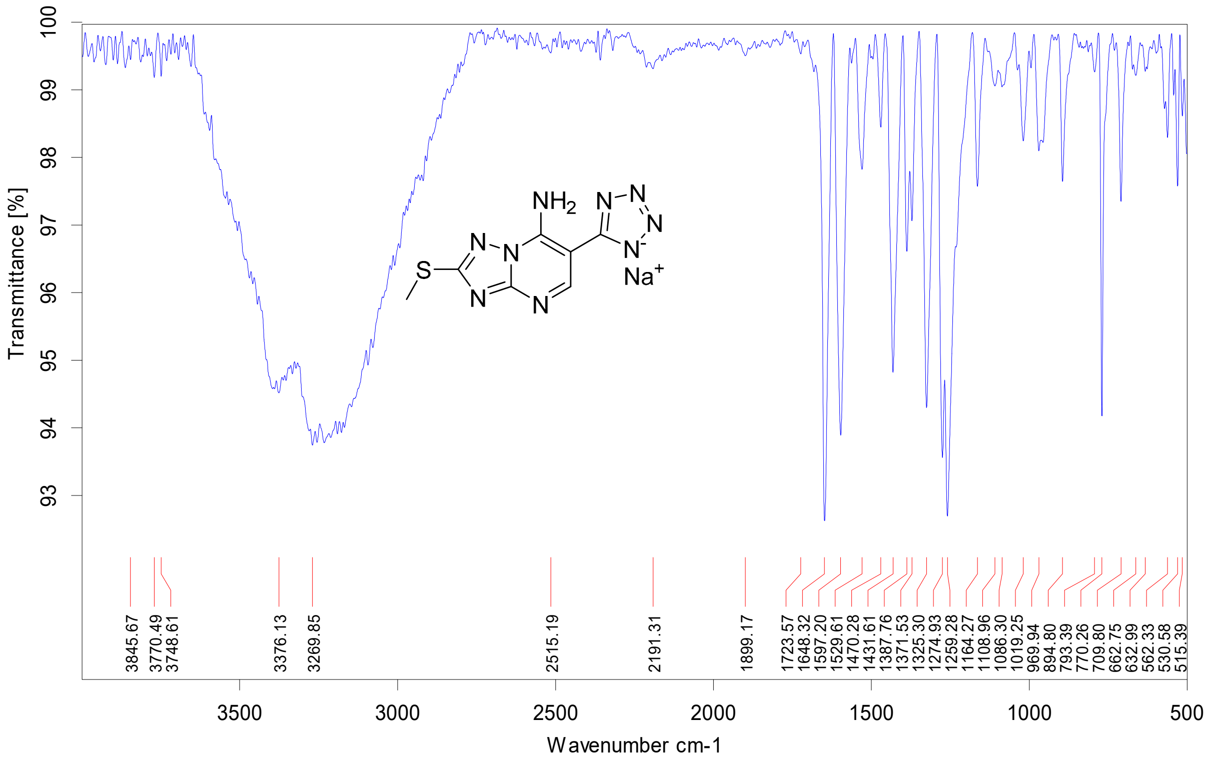

Figure S69. IR spectra of **11c**

Sodium 5-(7-amino-2-(benzylthio)-[1,2,4]triazolo[1,5-a]pyrimidin-6-yl)tetrazol-1-ide (11d)

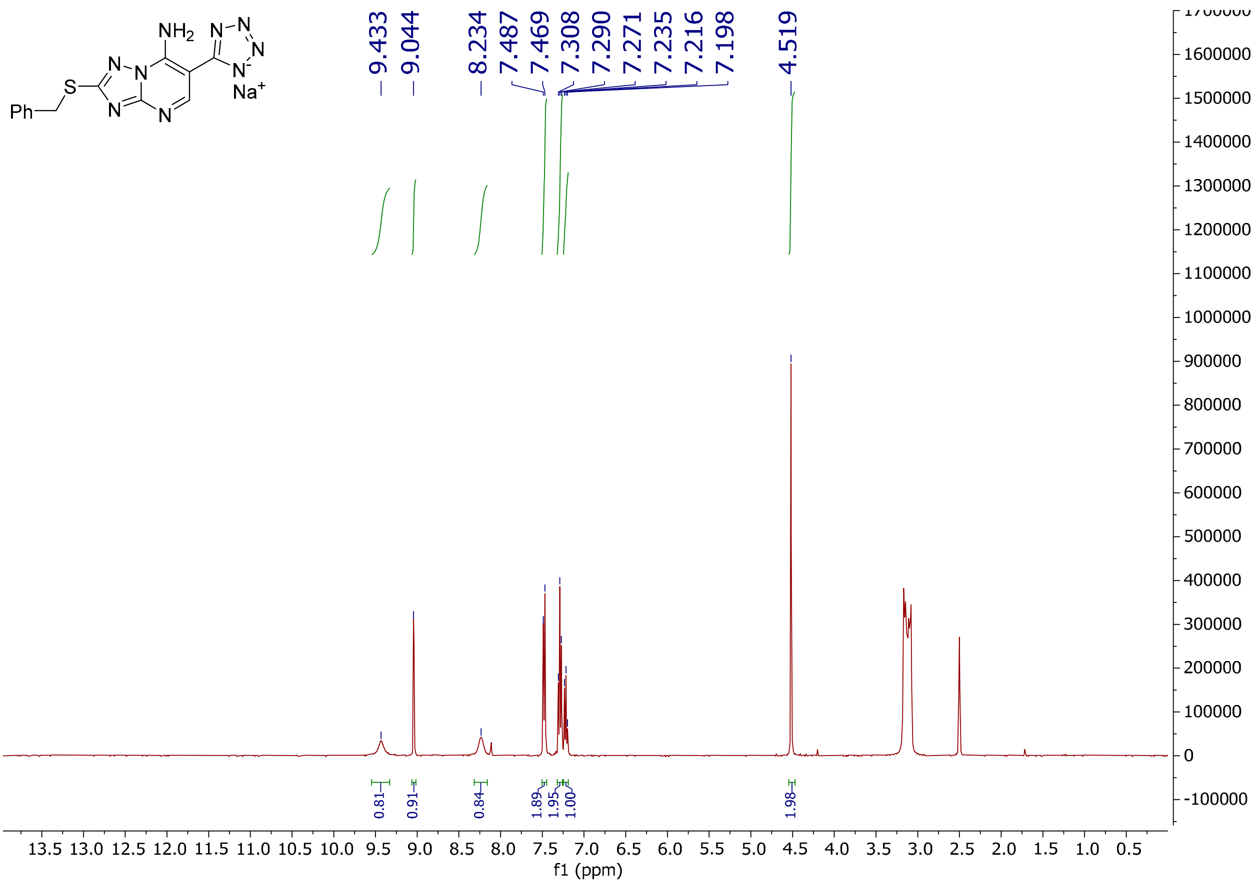

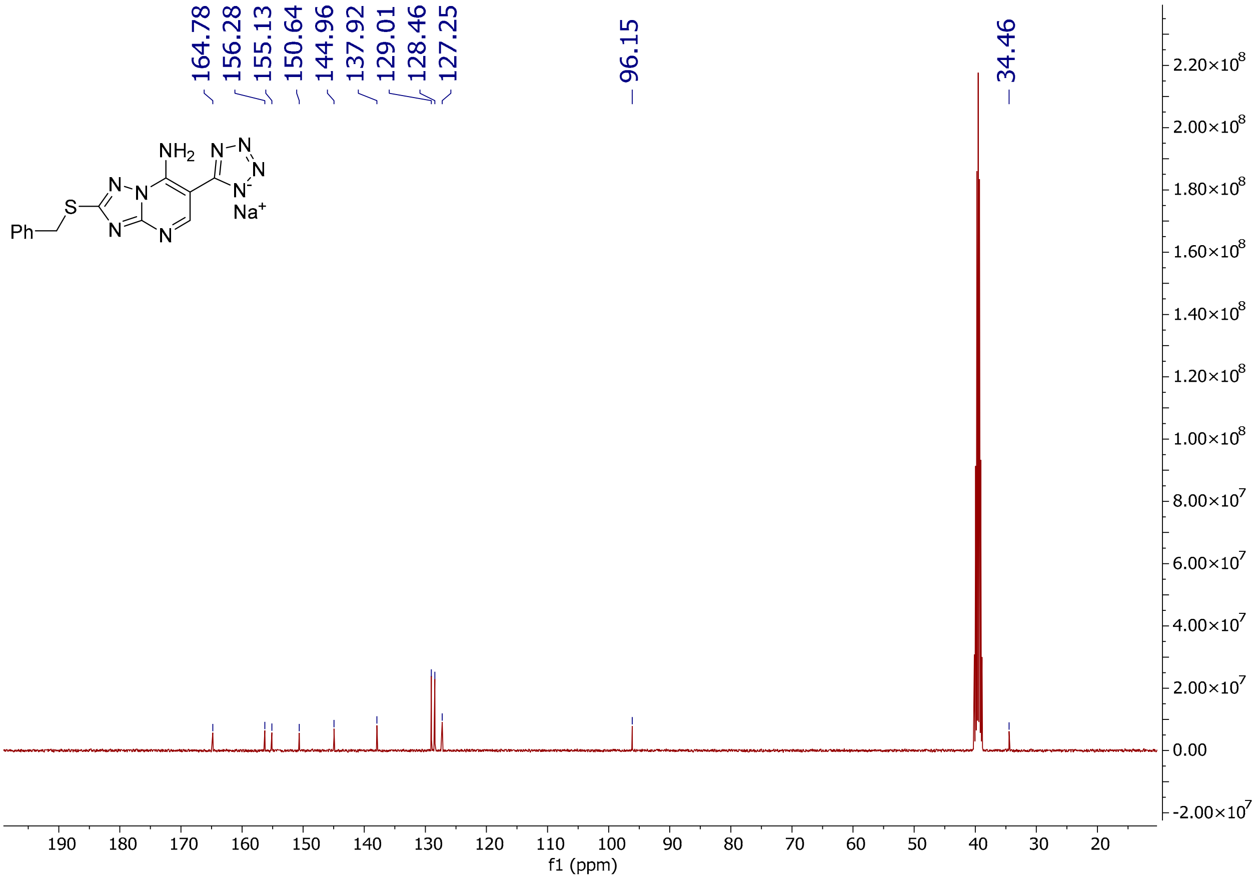

Figure S70. ^{1}H NMR (400 MHz, DMSO-d_6) and ^{13}C NMR (100 MHz, DMSO-d_6) spectra of **11d**

Sodium 5-(7-amino-2-(benzylthio)-[1,2,4]triazolo[1,5-a]pyrimidin-6-yl)tetrazol-1-ide (11d)

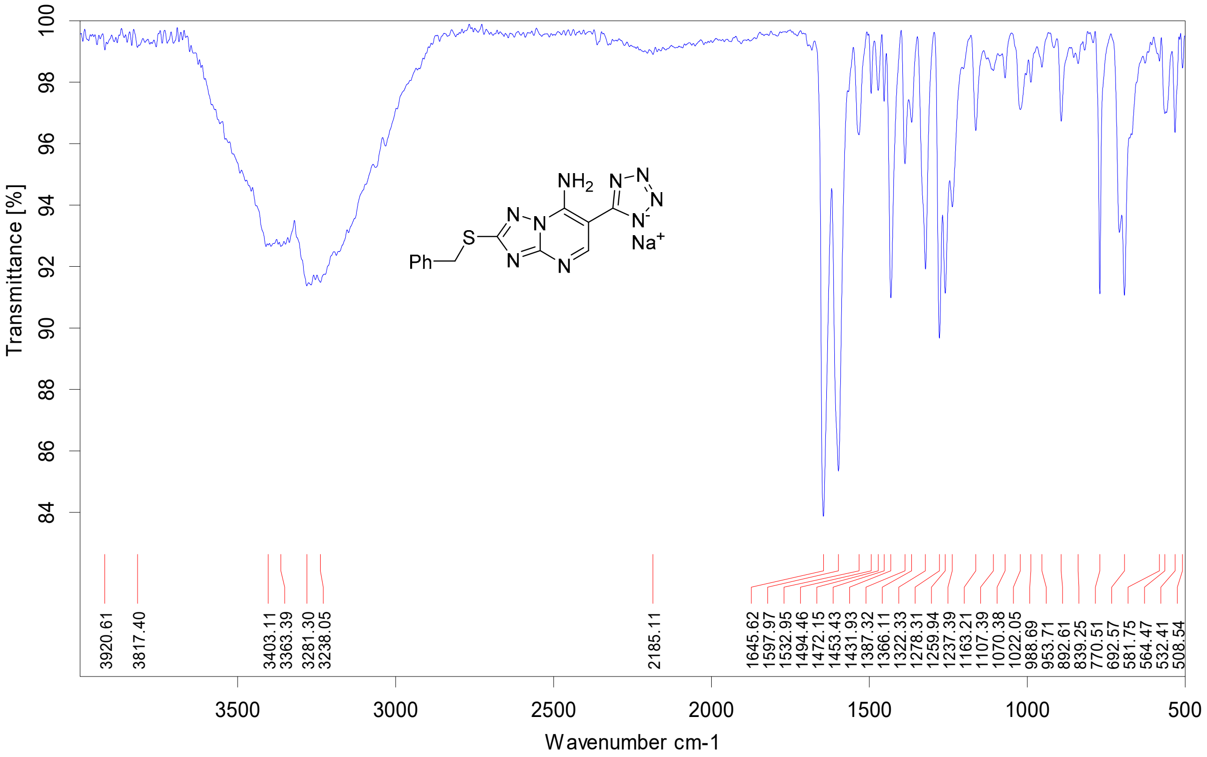

Figure S71. IR spectra of **11d**

Sodium 5-(7-amino-2-(trifluoromethyl)-[1,2,4]triazolo[1,5-a]pyrimidin-6-yl)tetrazol-1-ide (11e)

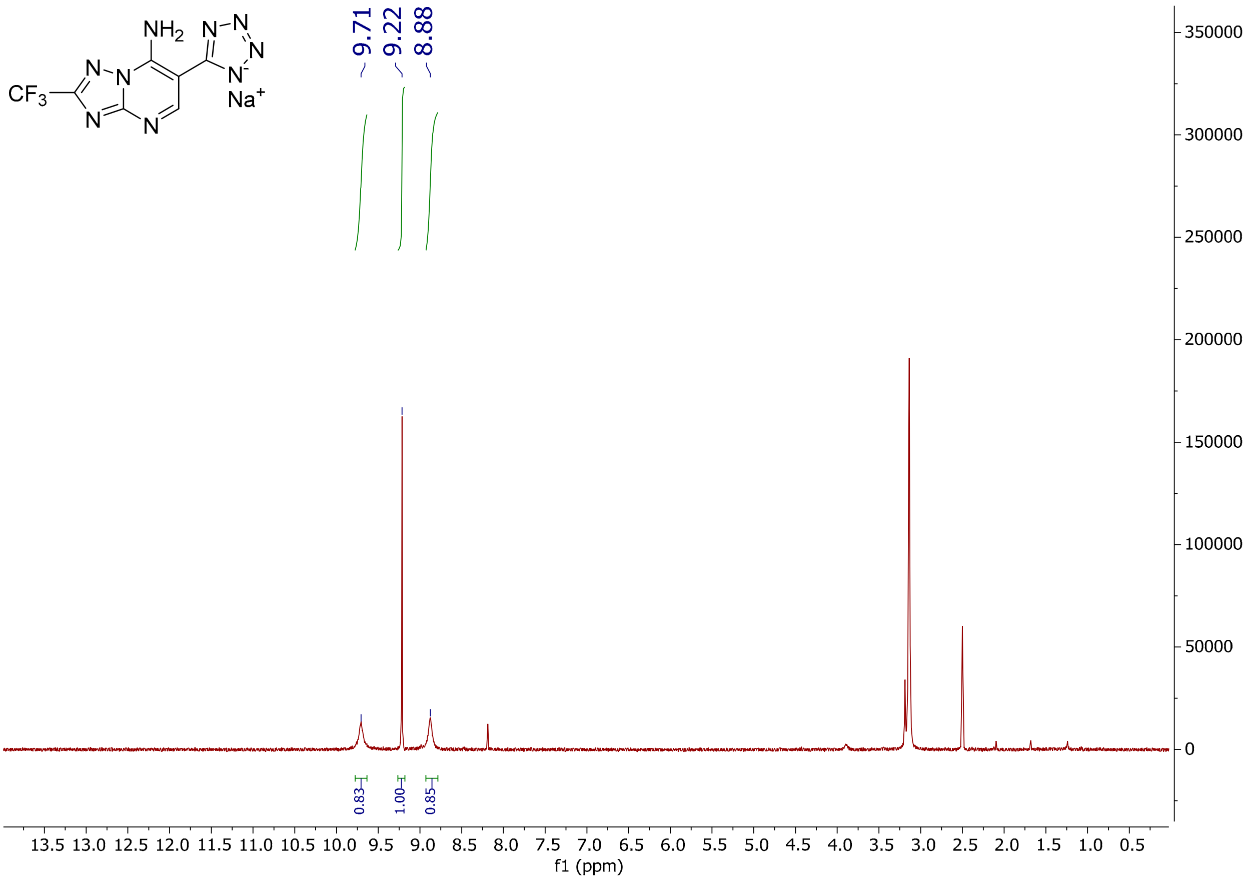

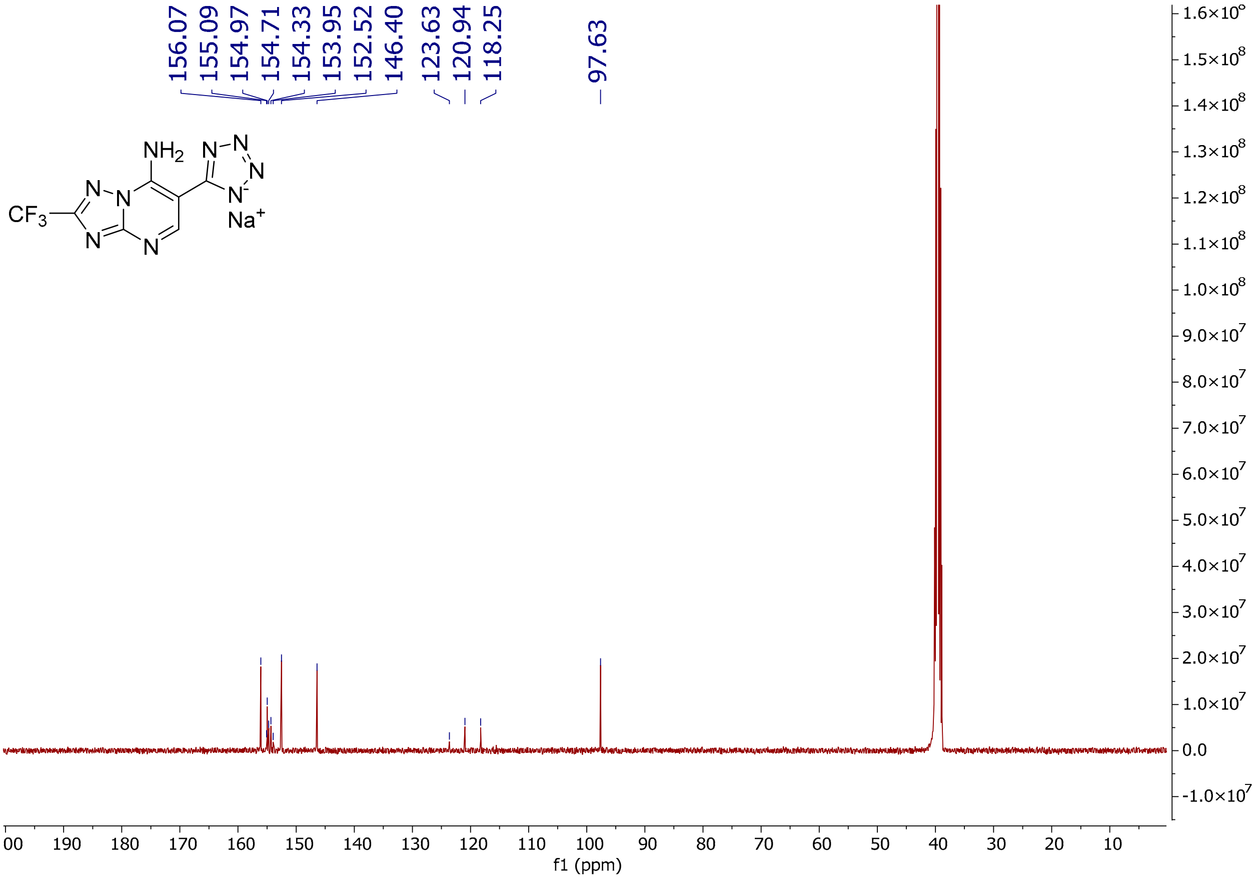

Figure S72. ^{1}H NMR (400 MHz, DMSO-d_6) and ^{13}C NMR (100 MHz, DMSO-d_6) spectra of **11e**

Sodium 5-(7-amino-2-(trifluoromethyl)-[1,2,4]triazolo[1,5-a]pyrimidin-6-yl)tetrazol-1-ide (11e)

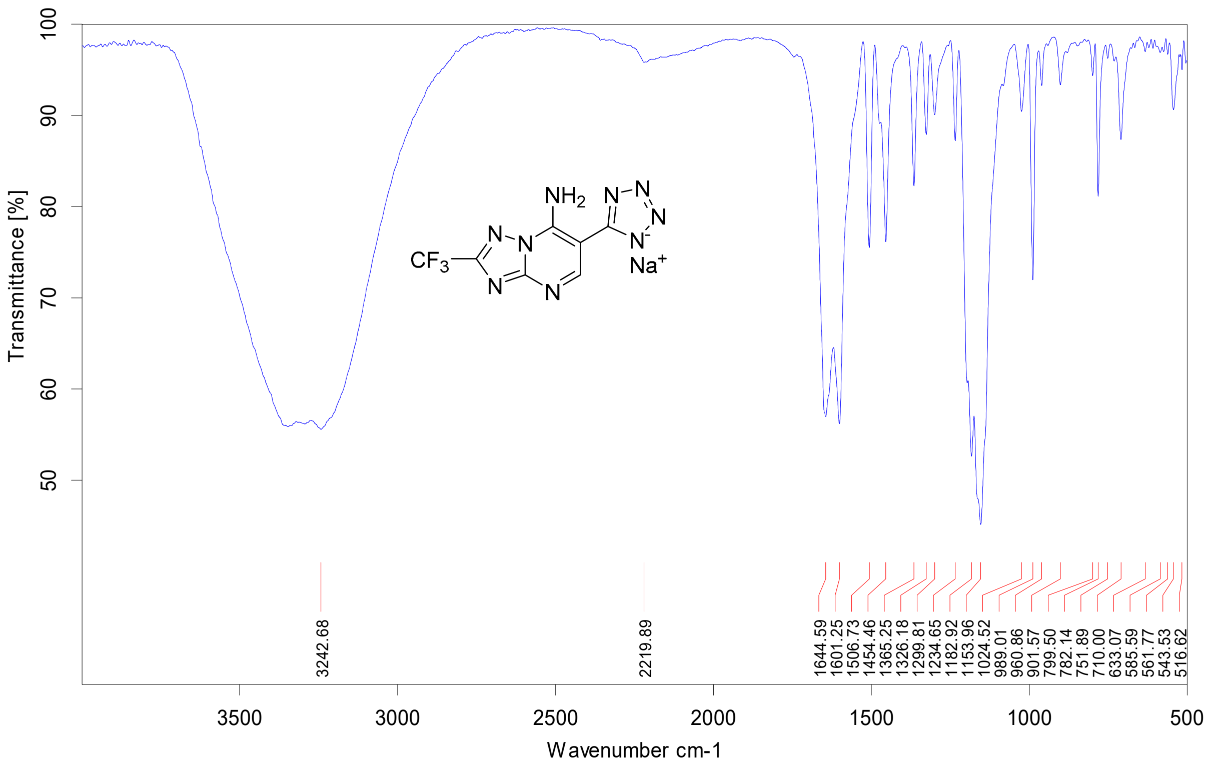

Figure S73. IR spectra of **11e**

Sodium 5-(7-amino-2-phenyl-[1,2,4]triazolo[1,5-a]pyrimidin-6-yl)tetrazol-1-ide (11g)

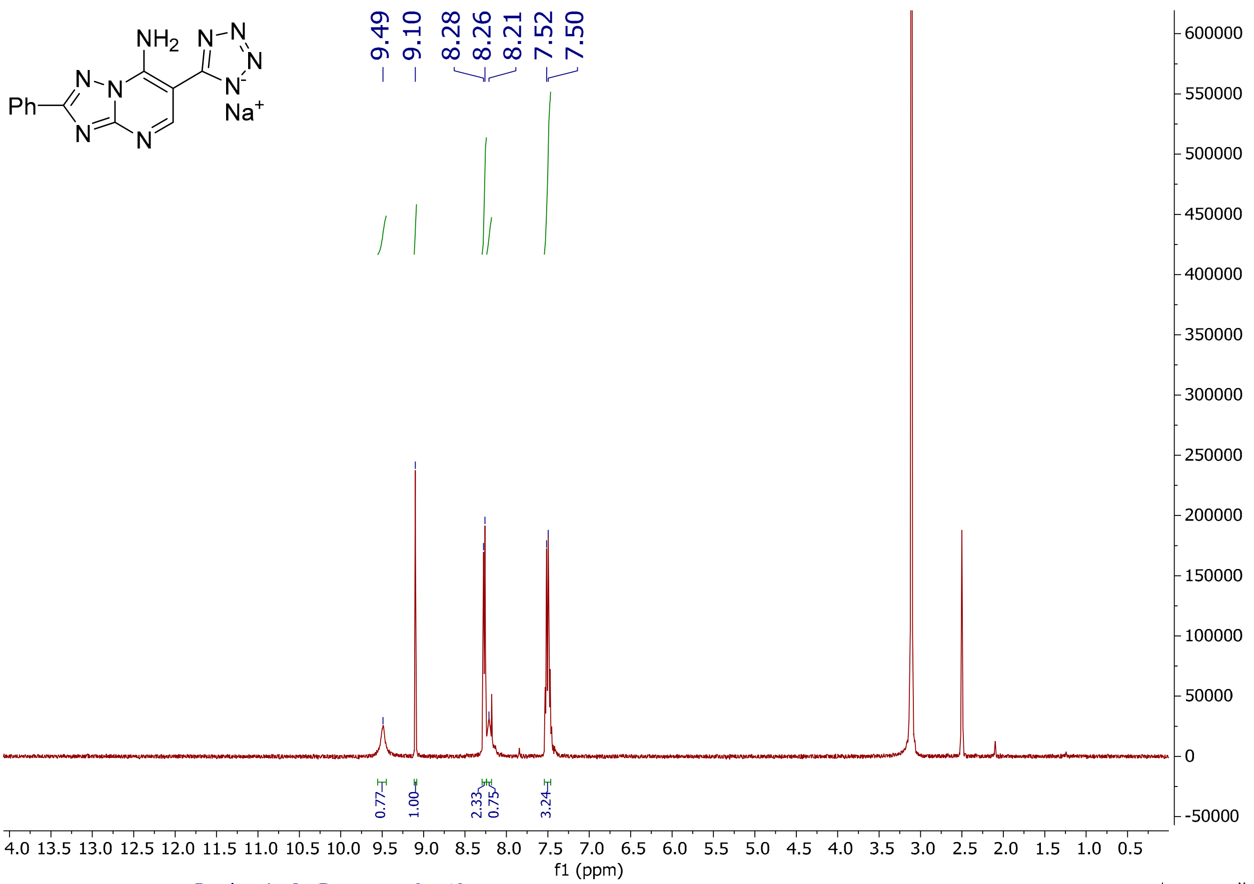

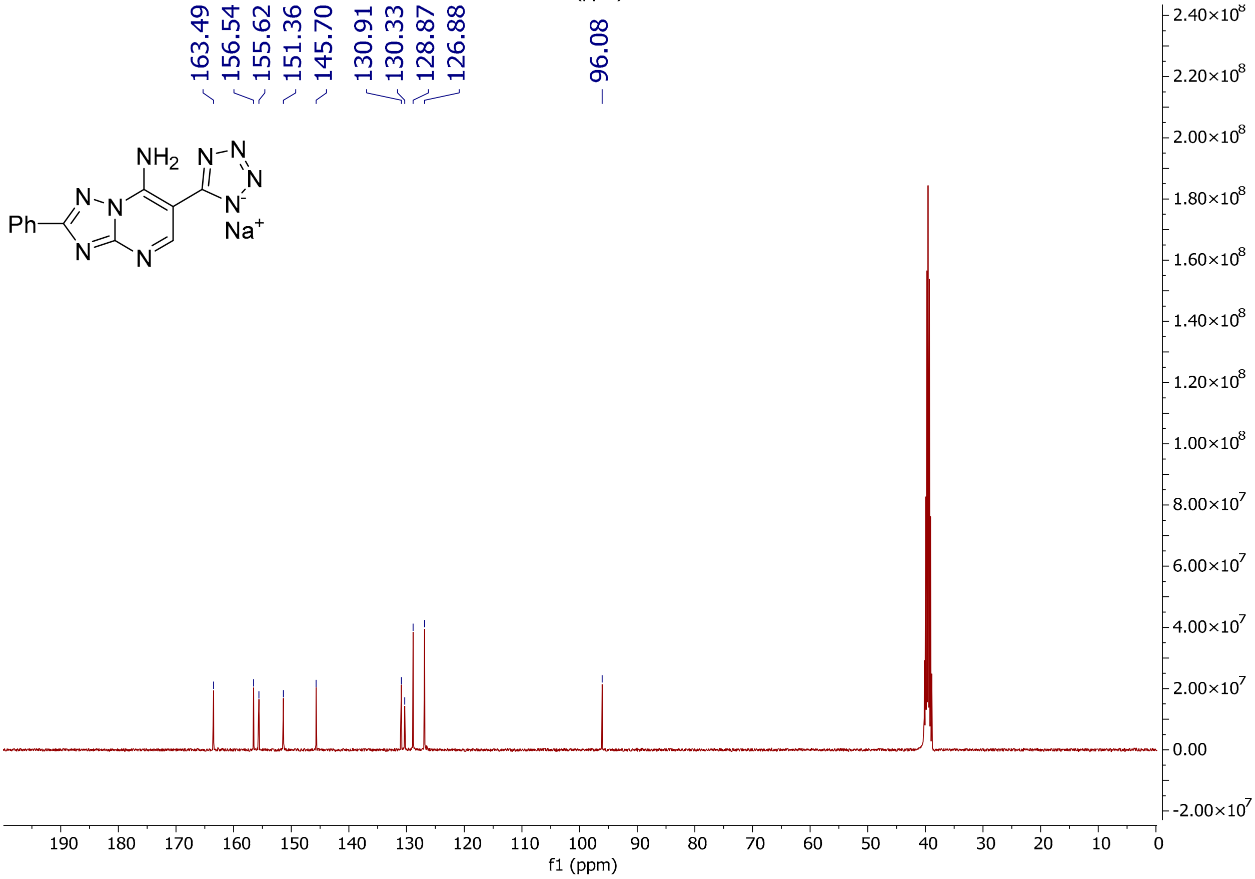

Figure S74. ^{1}H NMR (400 MHz, DMSO-d_6) and ^{13}C NMR (100 MHz, DMSO-d_6) spectra of **11g**

Sodium 5-(7-amino-2-phenyl-[1,2,4]triazolo[1,5-a]pyrimidin-6-yl)tetrazol-1-ide (11g)

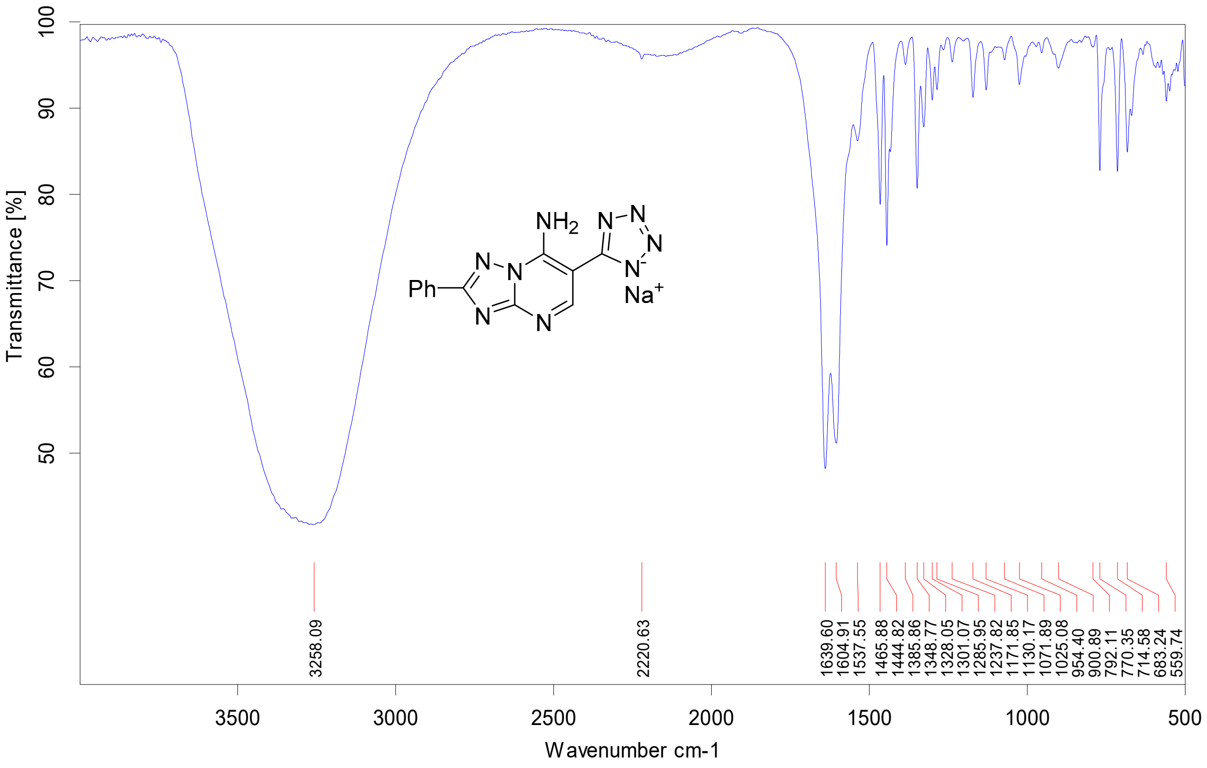

Figure S75. IR spectra of **11g**

Sodium 5-(7-amino-2-(furan-2-yl)-[1,2,4]triazolo[1,5-a]pyrimidin-6-yl)tetrazol-1-ide (11h)

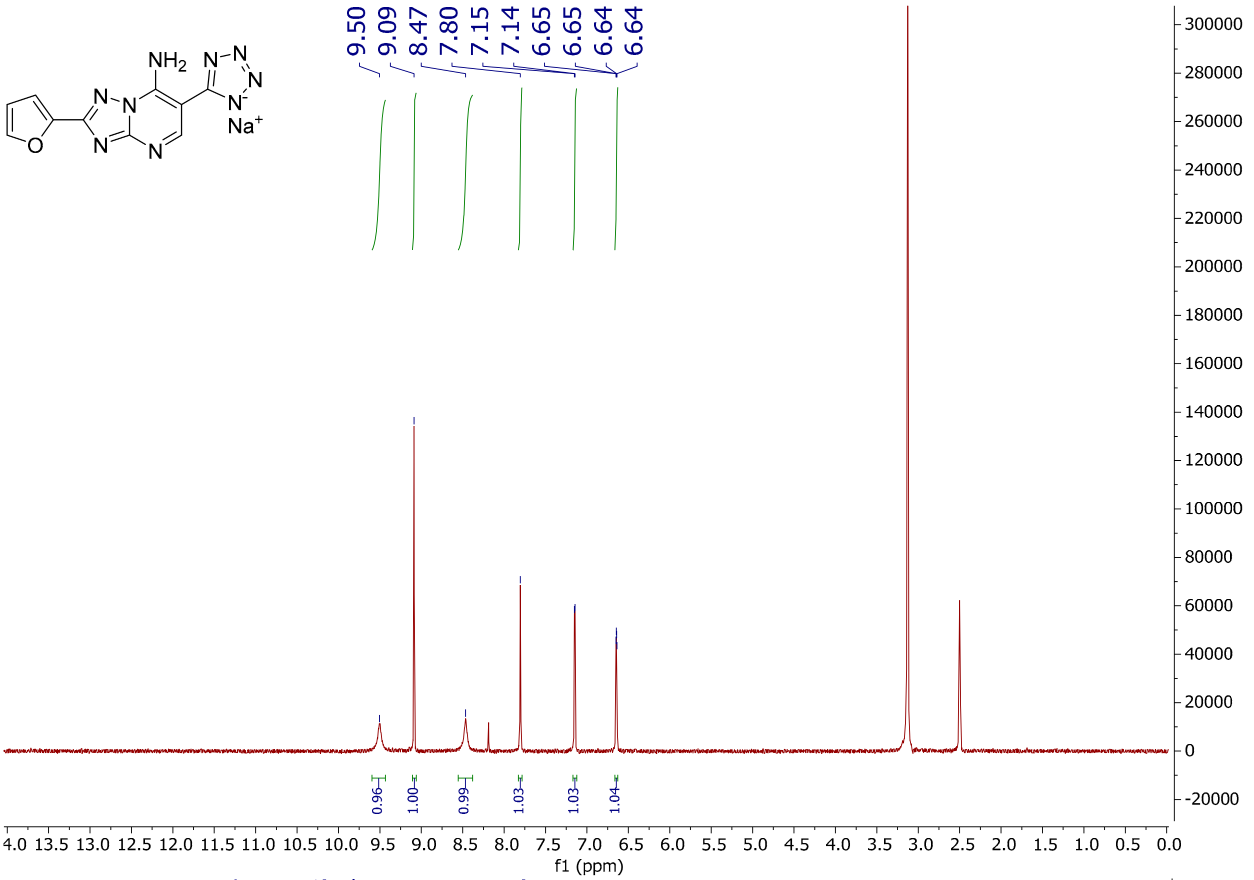

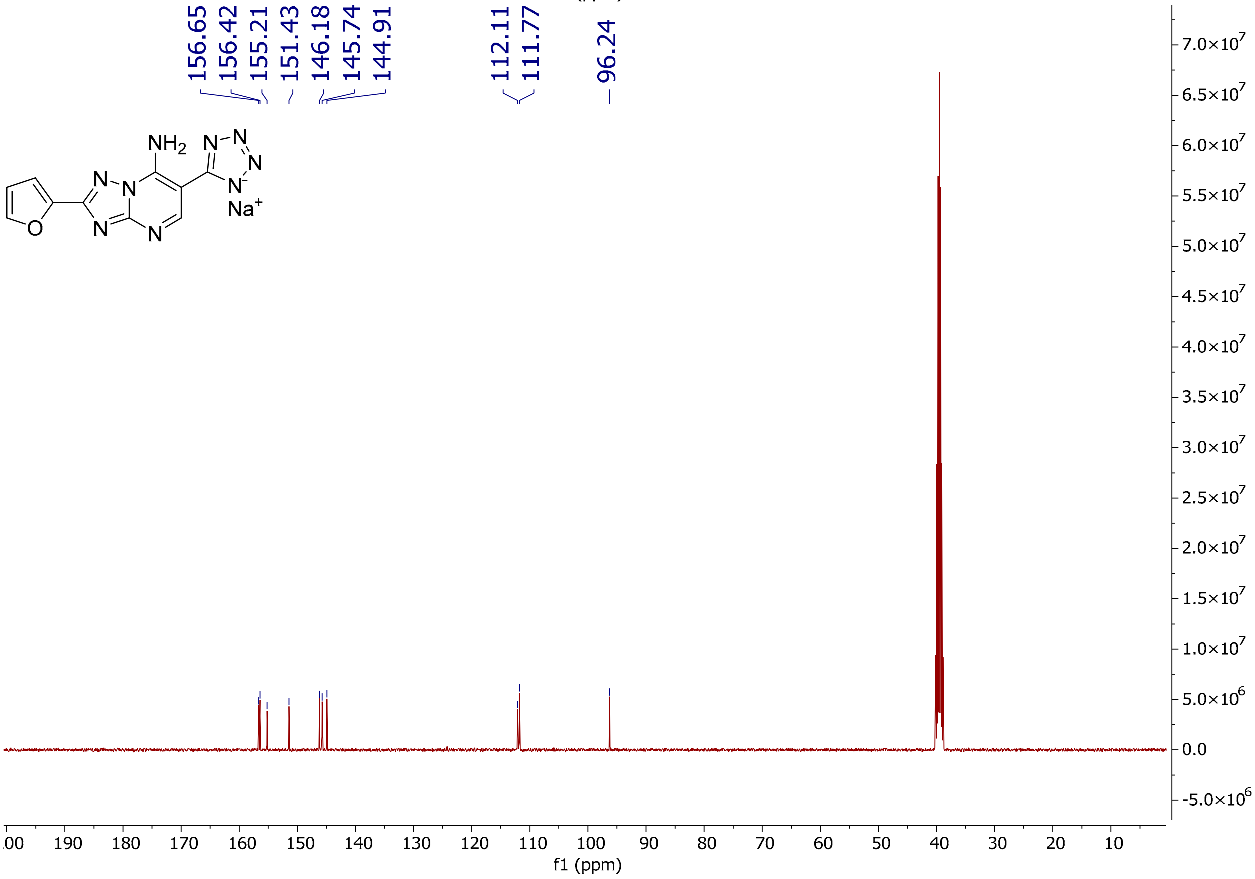

Figure S76. ^{1}H NMR (400 MHz, DMSO-d_6) and ^{13}C NMR (100 MHz, DMSO-d_6) spectra of **11h**

Sodium 5-(7-amino-2-(furan-2-yl)-[1,2,4]triazolo[1,5-a]pyrimidin-6-yl)tetrazol-1-ide (11h)

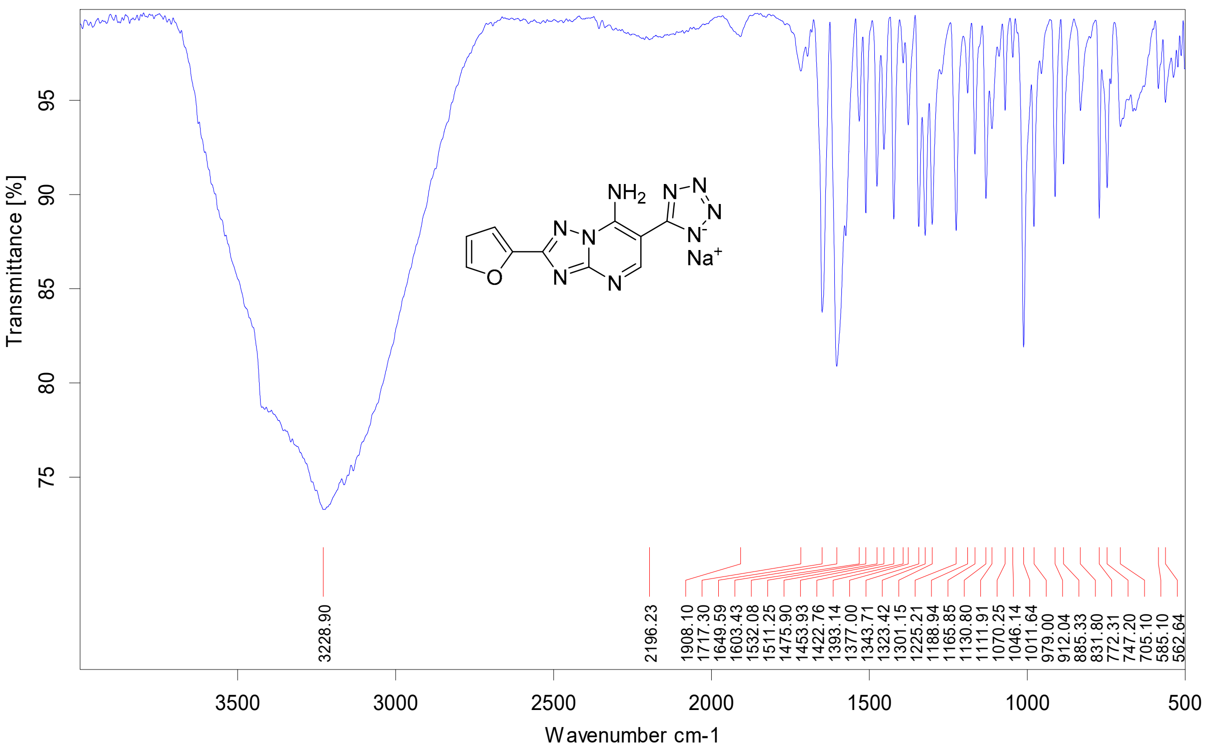

Figure S77. IR spectra of **11h**

Sodium 5-(7-amino-2-(pyridin-3-yl)-[1,2,4]triazolo[1,5-a]pyrimidin-6-yl)tetrazol-1-ide (11j)

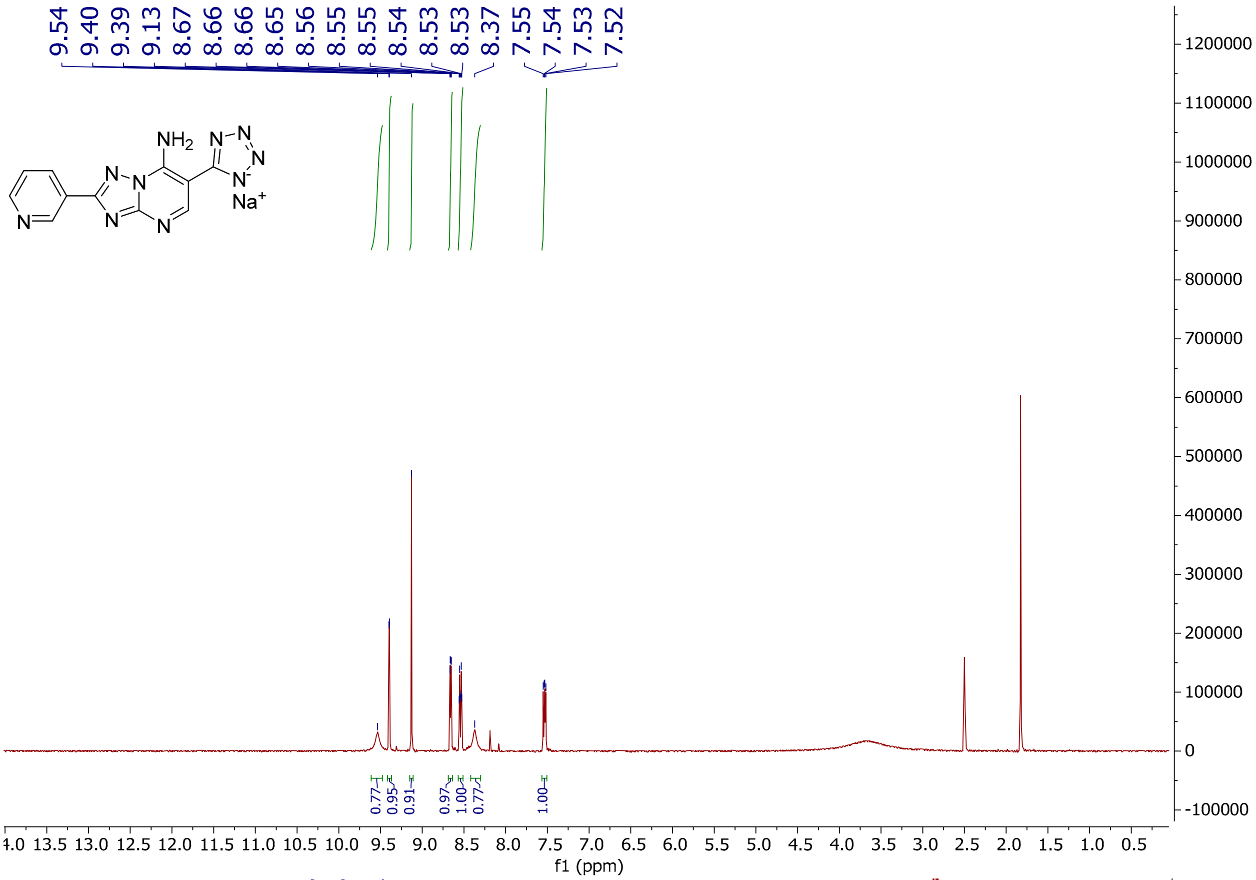

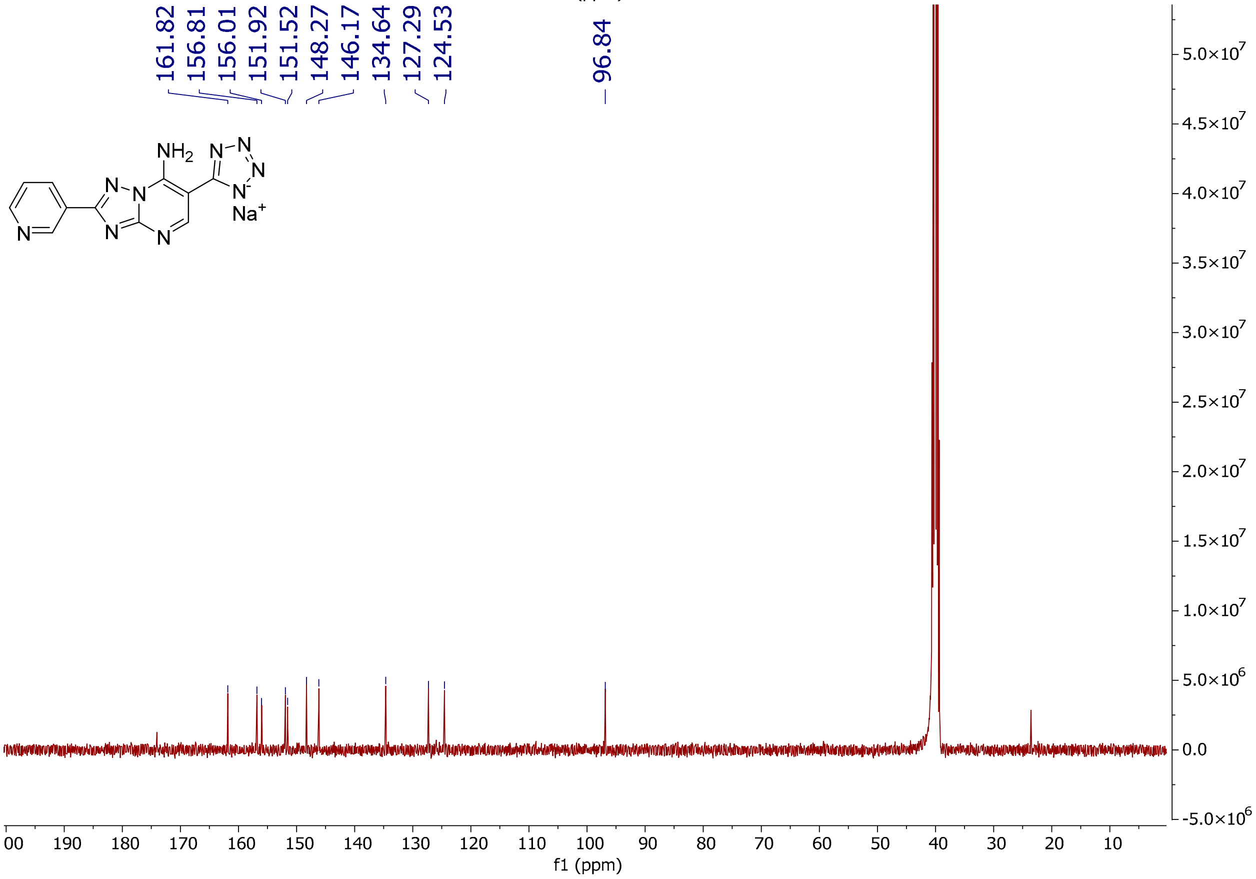

Figure S78. ^{1}H NMR (400 MHz, DMSO-d_6) and ^{13}C NMR (100 MHz, DMSO-d_6) spectra of **11j**

Sodium 5-(7-amino-2-(pyridin-3-yl)-[1,2,4]triazolo[1,5-a]pyrimidin-6-yl)tetrazol-1-ide (11j)

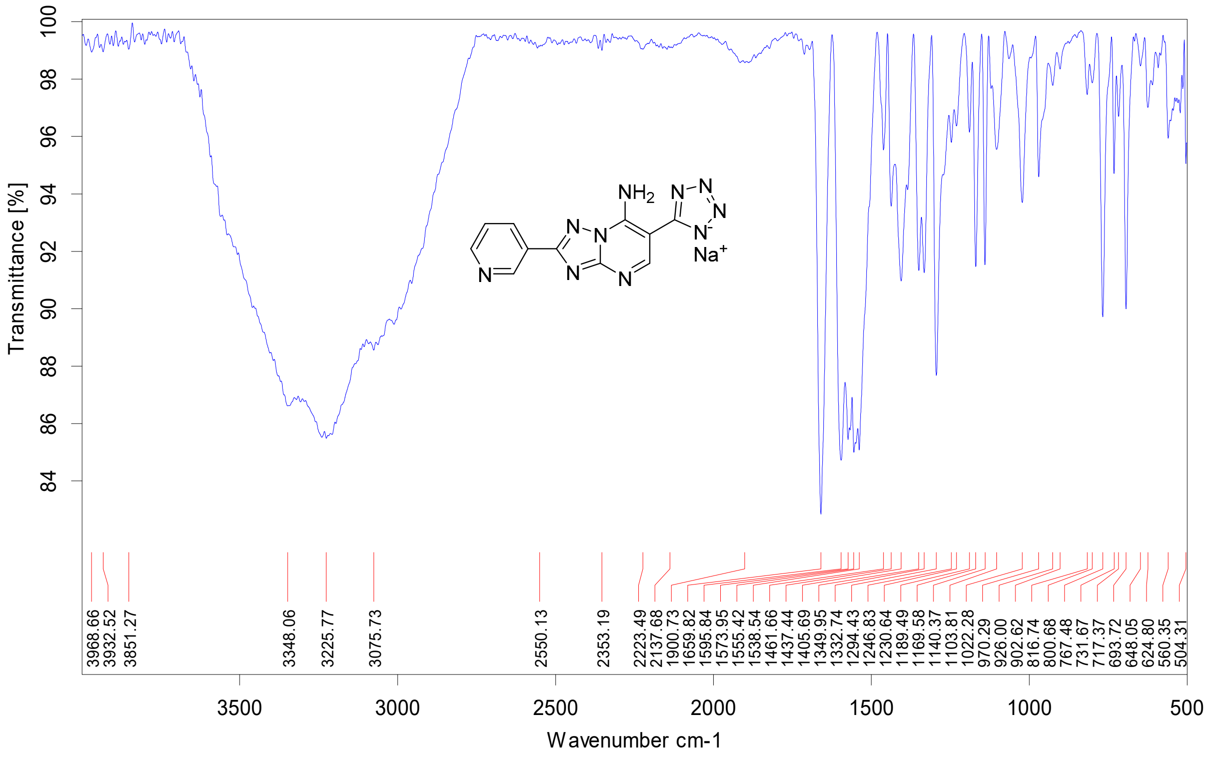

Figure S79. IR spectra of **11j**
